# Gut Microbiome Diversity, Functional Potential, and Ecological Relevance of *Hottentotta tamulus*

**DOI:** 10.1002/mbo3.70373

**Published:** 2026-09-01

**Authors:** Khajid Ullah Khan, Muhammad Tariq Zahid, Ghulam Mustafa, Rahul S. Tanpure, Hafiz Muhammad Tahir, Ramesh Kumar, Deok‐Won Kim, Hyun‐Kyung Park, Byong‐Hun Jeon

**Affiliations:** ^1^ Department of Zoology Government College University Lahore Pakistan; ^2^ Department of Earth Resources & Environmental Engineering Hanyang University Seoul Republic of Korea; ^3^ Department of Pediatrics Hanyang University College of Medicine Seoul Republic of Korea

**Keywords:** *Bacillus*, endosymbionts, functional prediction, gut microbiome, *Hottentotta tamulus*

## Abstract

Scorpions are ancient arachnids of medical and ecological importance; they prey on insects and other arthropods while serving as prey to birds and reptiles. In this study, we reported for the first time on the characterization of the gut intestinal microbiome of *Hottentotta tamulus* native to northeastern Pakistan. The scorpions were identified on a morphological basis and the Cytochrome c oxidase subunit 1 gene sequence, while the gut microbiome was characterized through full‐length 16S rRNA (V1‐V9) Nanopore sequencing. The gut microbiota exhibited low to moderate alpha diversity with Chao1 and Shannon indices of 126 ± 90.54 and 0.85 ± 0.21, respectively. The intestinal microbial community was dominated by the phyla Firmicutes (79.48%–90.43%), followed by Proteobacteria (9.46%–20.48%), whereas Actinobacteriota (0.03%–0.11%) and Bacteroidota (0.00%–0.01%) were present at very low relative abundance. The functional profiling identified 23 notable pathways involved in energy metabolism, biomolecule synthesis, the biodegradation of various xenobiotics, and nucleotide metabolism, highlighting the role of the gut microbiome in metabolic homeostasis. The dominance of *Bacillus* and *Mycoplasma* in gut microbial communities may enhance host adaptation to low‐resource environments.

## Graphical abstract

Long‐read 16S sequencing uncovers the gut microbiome of *H. tamulus*. A highly conserved and low‐diversity bacterial community dominates the gut fauna. *Bacillus* and *Mycoplasma* lineages emerge as predominant candidate endosymbionts. Exploratory profiles reveal potential sex‐specific trends in microbial abundance. Functional models predict metabolic pathways that may support host homeostasis.

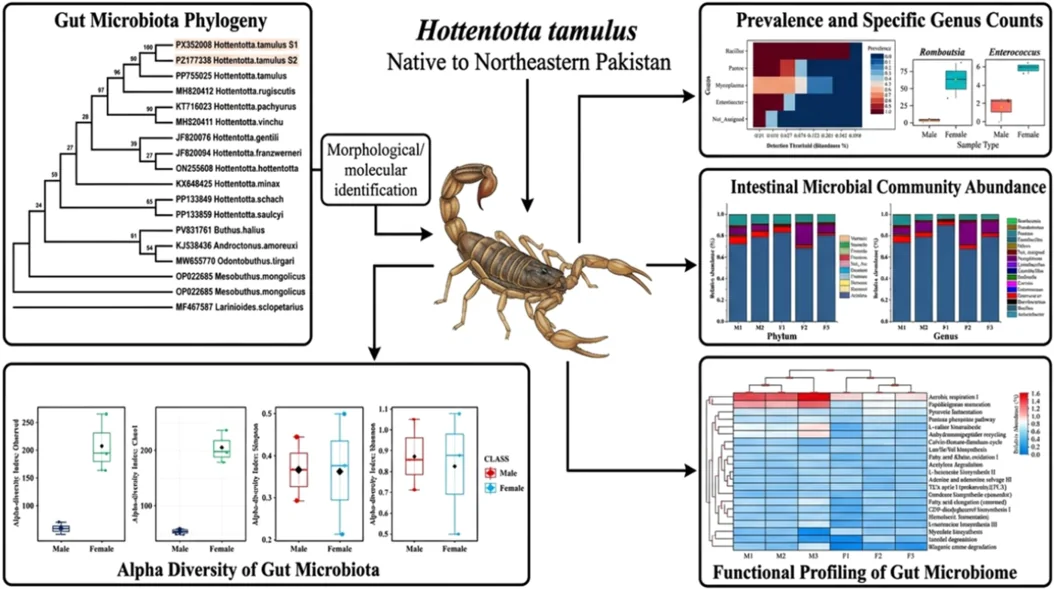

**Abbreviations**
ONT: Oxford Nanopore Technologies
OUT: Operational Taxonomic Unit

## 1 Introduction

Scorpions (Order Scorpions; Phylum Arthropoda) are perhaps the most unique and ecologically important group of arachnids. There are over 2800 known species of scorpions worldwide. Most scorpions are active at night and can be found in many habitats, including deserts, forests, mountainous terrain, and rocky outcroppings (Nasirian 2025). The diversity and distribution of these invertebrates are influenced by environmental variables, such as precipitation, humidity, temperature, soil characteristics, and vegetational cover (Ureta et al. 2020). Because scorpions are primary invertebrate predators that consume numerous small insects and other arthropods, they play an important role as food sources for a variety of animals, including reptiles, birds, and other venomous animals (Sh et al. 2021).

Venom from scorpions has proven to be a major public health problem in South America, North Africa, the Middle East, and parts of South Asia (Abroug et al. 2020; Ahsan et al. 2025). The family Buthidae includes several genera, including *Hottentotta*, which contains medically important species. Species in this family are distributed across Asia and Africa (Mabunda et al. 2024). The Indian red scorpion (*Hottentotta tamulus*, formerly known as *Mesobuthus tamulus*) is widely recognized as one of the most medically important scorpion species, distributed throughout the Indian subcontinent, spanning across India, Pakistan, Nepal, and Sri Lanka. This species holds great significance both as a public health hazard and as an asset in biomedical research (Das et al. 2021; Suranse et al. 2019). The venom of *Hottentotta* usually causes local pain and swelling. Severe systemic reactions are rare but may occur in some individuals (Dehghani et al. 2025; Sanaei‐Zadeh et al. 2017). For centuries, scorpions have been used in traditional medicine (Ortiz et al. 2015). Recent research has identified the bioactive compounds present in scorpion venom that could potentially treat serious illnesses such as cancer, which can provide opportunities for future development of drugs and biomedical applications (Das et al. 2021; Nosouhian et al. 2024; Pashmforoosh and Baradaran 2022; Suranse et al. 2019).

The gut microbiota plays a significant role in maintaining host health through aiding in digestion, metabolism, and immune function. It also supports the balance or homeostasis of the entire body (Alvarenga et al. 2024; Asnicar et al. 2021). In addition to assisting in nutrient absorption and providing energy by breaking down undigested materials via fermentation into short‐chain fatty acids, the microbiome produces vitamins and a wide array of bioactive molecules that have multiple functional effects (Ankrah and Douglas 2018; Fritz et al. 2024). These bioactive molecules influence the level of inflammation and host metabolism through the regulation of short‐chain fatty acid production, thus relating the activity of gut microbes to whole‐body physiological systems (Nogal et al. 2021). Many different bacteria contribute to digestion by producing enzymes that break down starches and fats. Examples include *Bacillus* spp., *Enterococcus* spp., and *Pseudomonas* spp. Some bacteria also produce antibiotics and anti‐proliferative compounds in insects, such as moths and silkworms (Gandotra et al. 2018; Gharsallah et al. 2019; Liang et al. 2018; Liang et al. 2019; Shin et al. 2019). Recently, Atkinson et al. (2024) were able to isolate microbiota from the mesosoma and metasoma of the giant sand scorpion, *Smeringurus mesaensis*. Mesosoma accounts for 55% percent of those antibiotic‐producing strains. Advances in DNA sequencing technologies have provided extensive insights into the functions of the gut microbiome. This indicates how gut microbes affect host metabolic pathways, particularly lipid metabolism (Li et al. 2022).

Scorpions are venomous predatory arthropods of immense scientific significance due to their evolutionary adaptations and the therapeutic potential of their venom bioactive molecules. Despite the extensive research on their ecological role, venom chemistry, and therapeutic applications, little is known about the composition and functional dynamics of gut microflora. Gut microbiota mediate a critical role in host‐environmental interaction; the primary objective of this study was to provide a comprehensive, descriptive analysis of the gut bacterial microbiome of *H. tamulus* collected from an unexplored area of Deva Vatala National Park in Pakistan. We also studied the predicted functional and metabolic potential of gut microbiota using PICRUSt2. Additionally, we explored sex‐related variations in the gut microbial communities of male and female scorpions. Specimen identification was confirmed using a morphological key and COI gene barcoding, while gut bacterial microflora was characterized using Oxford Nanopore Technologies long‐read 16S rRNA sequencing.

## 2 Materials and Methods

### 2.1 Scorpion Sampling

The authors obtained scorpion samples at the time of year when they are most active (June to July) from rocky field sites surrounding the Deva Vatala National Park in Pakistan (32.8949° N, 74.2981° E) using two identical sampling procedures (diurnal and nocturnal). Diurnally, the investigators identified the specimens by digging beneath rock and stone substrates (Khammassi et al. 2024). At night, an ultraviolet light source was employed to detect the specimens, and a pair of forceps was used to collect them. The specimens were then placed into individual ventilation‐assisted clear plastic containers (17 × 9 × 9 cm; length × width × height) utilizing 2–3 cm of soil as a substrate (Tobassum et al. 2018). A total of 126 scorpions (85 females, 41 males) were collected, exhibiting dark brown coloration and characteristic morphological features of *H. tamulus*. In addition to being kept in laboratory settings where their environment was regulated, the scorpions were fed live grasshoppers and misted with water to provide moisture to their habitat.

### 2.2 Morphological Description and Molecular Characterization

The key to identifying the morphology of this specimen lies in the two sets of keys described in detail in Pocock (1900). In addition, we reviewed other important sources related to taxonomy for each species. To specifically look at characteristics specific to individual species, we used a stereomicroscope (Leica, Germany) to observe various parts of the scorpion's morphology. These included looking at the pincer or claw; examining the carapace structure; determining how many segments there were in the metasoma; evaluating the telson's morphology; and assessing the pattern of trichobothria. Additional characteristics that may be useful for making an identification include evaluation of granulation patterns, the presence/development of a keel, and examination of ventral carinae. Evaluating sexual dimorphism occurred when comparing the number of pectinal teeth to metasomal thickness. Total body length, metasomal length, and size of the claws (chela) were measured using a precision caliper (± .001 mm). All our data was cross‐checked with existing information in the taxonomic literature. To confirm the identity of these specimens to the species level, molecular analysis of the COI gene was completed. Molecular analysis of the COI gene provides greater discrimination than morphological identification alone. DNA was extracted from the muscle tissue of the scorpion's tail using a Thermo Scientific Genomic DNA Purification kit (Thermo Fisher Scientific, Waltham, MA, USA) according to the manufacturer's protocol. The COI gene was amplified via PCR using universal primers LCO1490 and HCO2198 (Khammassi et al. 2024). The resulting amplicons were then subjected to Sanger sequencing. The sequences were compared with those in GenBank (NCBI database) for both identification and phylogenetic analysis. A phylogenetic tree was created in MEGA11 using homologous sequences from GenBank, with *Larinioides sclopetarius* (Araneae: Araneidae) as the outgroup.

### 2.3 Metataxonomic Analysis of Scorpion Gut Microbiome

#### 2.3.1 Gut Dna Extraction

Three adult male and three adult female scorpions from the collected specimens were dissected immediately upon arrival in the laboratory. They were surface sterilized with 70% ethanol swabs and dissected aseptically in a laminar airflow cabinet. Intestinal materials were directly collected from the intestine, suspended in autoclaved distilled water, transferred into sterile microfuge tubes, and stored at −20°C until further processing. Total genomic DNA from the gut microbiota was extracted using a ZymoBIOMICS DNA Miniprep Kit (Cat. # D4300, ZymoBIOMICS, USA) according to the manufacturer's instructions. Briefly, the samples were transferred into Zymo BashingBeads lysis tubes, mixed with lysis buffer, and homogenized using a bead homogenizer (Bead Ruptor Elite, OMNI International, Kennesaw, GA, USA) at 6 m/s for 3 min, with a 5 min interval every 60 s (Cha et al. 2023; Hwang et al. 2026).

#### 2.3.2 ONT MinION Library Preparation

Genomic DNA was processed for full‐length *16S rRNA* gene sequencing of the bacterial communities using an SQK‐16S024 kit (ONT, Oxford, UK). The V1‐V9 hypervariable regions were amplified using barcoded universal primers (27 F/1492 R) in a MiniAmp Plus thermal cycler (Thermo Fisher Scientific, Cat# A37835). A negative PCR control containing nuclease‐free water without a DNA template was processed in parallel with the gut samples. The PCR program comprised an initial denaturation at 95°C for 30 s, followed by 25 cycles of denaturation at 98°C for 10 s, annealing at 55°C for 30 s, and extension at 65°C for 90 s, and ended with a final extension at 65°C for 5 min (Cha et al. 2023; Hwang et al. 2026). The amplicons were purified using Agencourt AMPure XP magnetic beads (Beckman Colter, USA) and quantified using a Qubit 4 Fluorometer (Thermo Fisher Scientific, Cat# Q33238). Purified libraries (100 fmol in 10 μL) were loaded onto a MinION Flow Cell (R9.4.1, FLO‐MIN‐106D) and sequenced for up to 48 h in super‐accurate basecalling mode. After sequencing, the flow cell was cleaned with a Flow Cell Wash Kit (EXP‐WSH004) according to the manufacturer's instructions (Seo et al. 2026).

### 2.4 Bioinformatics Analysis of Metataxonomic Data

ONT *16S rRNA* sequencing data were generated via MinKNOW software (v23.04.05, ONT) and processed using the QIIME2 (v2024.2) pipeline(Minich et al. 2025). After quality control and dereplication, reads were clustered *de novo* into operational taxonomic units (OTUs) at an 85% identity threshold using VSEARCH. Oxford Nanopore Technologies (ONT) sequencing is known to have higher base‐calling error rates (5%–10%) compared with short‐read technologies such as Illumina platforms (Zhang et al. 2023). To account for these sequencing errors and avoid excessive loss of valid reads during alignment, an identity threshold (85%) was applied during taxonomic classification. Similar relaxed thresholds have been used in several nanopore‐based microbial community studies to improve read retention during long‐read alignment (Cha et al. 2023; Curren et al. 2019; Guta et al. 2025). The taxonomic classification was performed against the SILVA *16S rRNA* reference database with a query coverage of 0.8 (Cha et al. 2023). Taxa that occurred predominantly in the negative controls (Table S1) and had relative abundance equal to or greater than that in the biological samples were considered potential contaminants and removed from the final feature table before downstream analyses. In addition, alpha diversity metrics (observed species, Chao1, Shannon & Simpson) and phylogenetic relationships were obtained by generating a rooted tree using QIIME2 (Cha et al. 2023). Additionally, the predicted functional capabilities of the gut microbiome were estimated to utilize PICRUSt2, with MetaCyc‐based metabolic pathway abundances inferred from the 16S rRNA gene profile (Douglas et al. 2020; Kim et al. 2025; Ling et al. 2022; Tyagi et al. 2021).

## 3 Results

### 3.1 Morphological and Taxonomic Characterization

**Figure 1:**
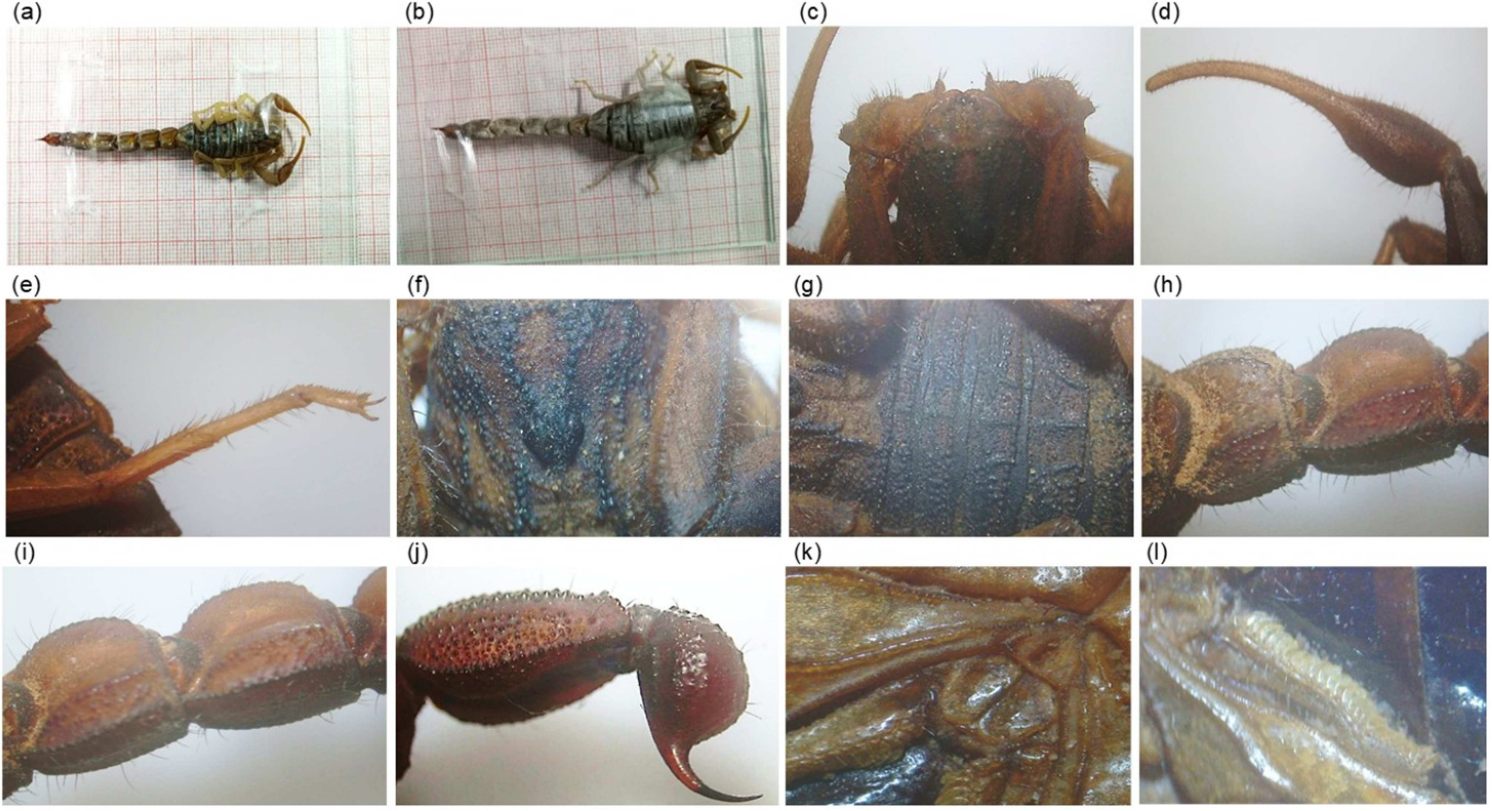
Morphological and taxonomic features of *Hottentotta tamulus*. (a) Adult male of Hottentotta tamulus (b) Adult female specimen (c) chelicerae (d) pedipalp (e) claw tarsus and tibia (f) median eyes (g) mesosomal tergites I–V (h) metasomal segments I–II (i) metasomal III–IV (j) segment V with telson (k) Genital operculum and triangular shaped sternum special character of Bothidae family (l) pectinal teeth.

A total of six scorpions (three females and three males) with all specimens displaying the typical black‐brown coloration and distinctive characteristics were used for morphological characterization. The pedipalp was densely covered with fine hair, while the femur and patella of the walking legs contained coarse hair that was significantly longer than that on the pedipalps. Both male and female specimen's exhibit a long carapace with defined median and lateral carinae, and distinctly separated granules adjacent to their median eyes. The mesosoma was dark in color, and the carinae on the terga were patterned similarly to those on the carapace. The metasoma was clearly segmented with pronounced carinae and sparsely hairy; the first segment of the metasoma was wider than long, while the second segment was longer than wide. The third and fourth segments had small granules, while the final segment had pronounced lateral granulation. The telson vesicle was large, reddish, and bulbous with a curved aculeus that had darkened tips. On the ventral side, the specimen had a triangular‐shaped sternum, which is a special character of the Bothidae family; the right and left pectinal counts were 26 in each (Figure 1).

Clear evidence of sexual dimorphism among our specimens is also obvious, with females being much thicker than males, particularly when comparing the relative widths of body parts, whereas males appeared thin overall compared to females. On the ventral surface, a distinct triangular sternum and genital opercula were clearly visible (Karataş and Gharkheloo 2006; Kovařík and Lowe 2021; Kularatne et al. 2015; Lira et al. 2018; Monod et al. 2017; Ranawana et al. 2013). Comparative morphometric measurements of male and female scorpions are shown in Table 1.

**Table 1:** Morphological measurements of adult male (*n* = 3) and female (*n* = 3) *H. tamulus* (mean ± standard deviation).

Morphological character	Male	Female	Significance
Carapace	10.23 ± 0.24 mm	9.10 ± 0.19 mm	**
Mesosoma	14.61 ± 0.62 mm	20.99 ± 0.53 mm	**
Metasoma	39.18 ± 0.45 mm	42.67 ± 0.90 mm	**
Chela	13.33 ± 0.43 mm	13.11 ± 0.27 mm	NS
Patella	9.64 ± 0.32 mm	8.19 ± 0.26 mm	**
Femur	8.43 ± 0.33 mm	7.21 ± 0.25 mm	**
Dactylus	8.99 ± 0.22 mm	9.23 ± 0.29 mm	NS
Right pectinal count	30.33 ± 0.58	26.00 ± 1.00	**
Left pectinal count	31.67 ± 0.58	26.33 ± 0.58	**
Right pectinal comb	6.20 ± 0.20 mm	5.45 ± 0.13 mm	**
Left pectinal comb	7.40 ± 0.20 mm	6.09 ± 0.14 mm	**
Total length	62.94 ± 2.06 mm	72.83 ± 1.82 mm	**

*Note:* (Values are mean ± standard deviation; *p < 0.05, **p < 0.01, not significant: NS).

### 3.2 DNA Barcode Analysis of Scorpions

**Figure 2:**
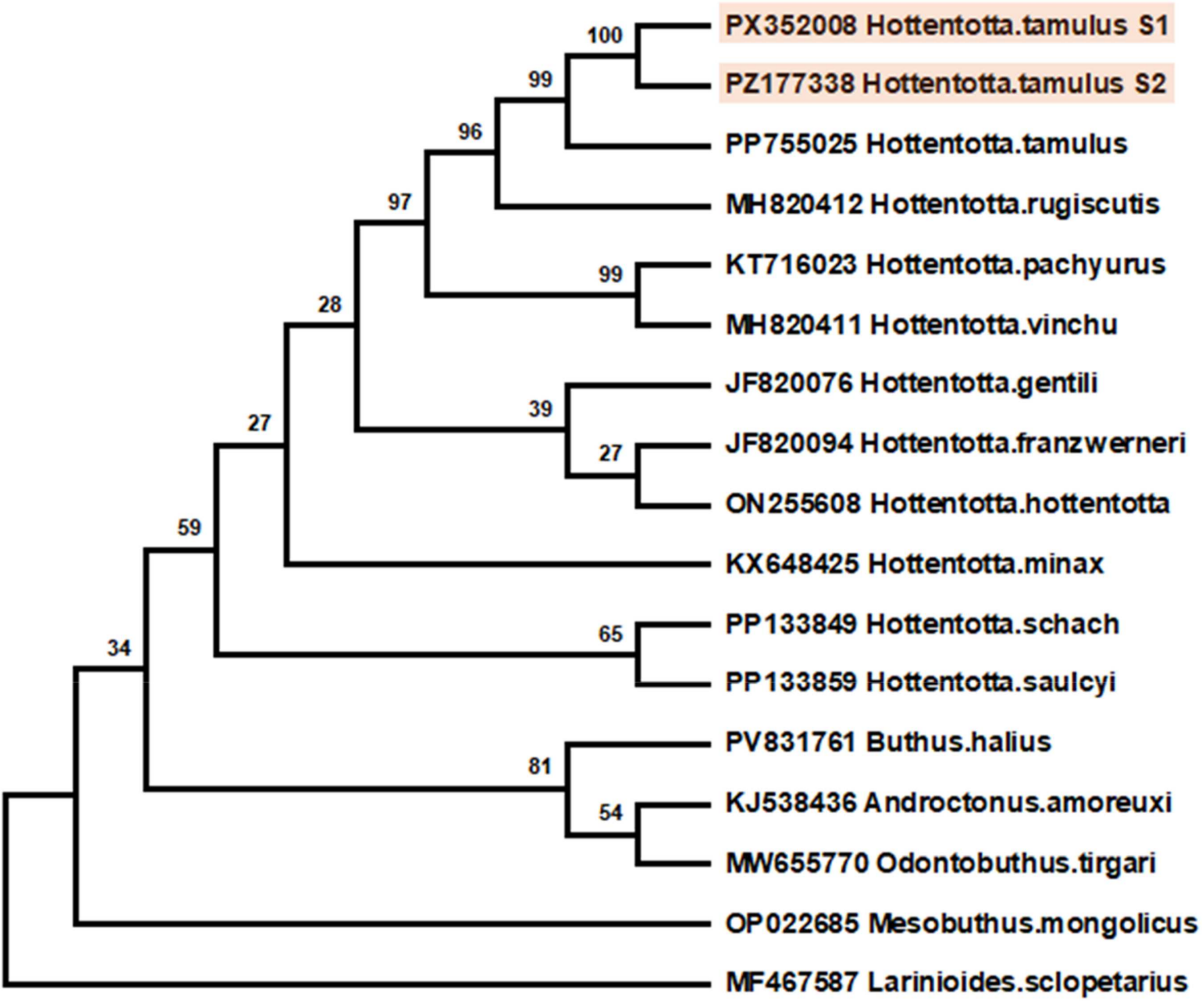
Evolutionary relationships were inferred using the Maximum Likelihood method under the Tamura‐Nei model. Initial trees were generated using the Neighbor‐Joining and BioNJ algorithms (Tamura‐Nei distances); the topology with the highest log‐likelihood was selected. The analysis included 18 nucleotide sequences comprising 675 positions and was performed using MEGA11 (Tamura et al. 2021).

PCR amplification of the mitochondrial *COI* barcode region from two collected scorpions yielded amplicons of approximately 650 bp. BLAST analysis against the NCBI database revealed that the amplicons had nucleotide identities of 96.95% and 96.81% with *H. tamulus* (accession no. PP755025). In silico translation of the nucleotide sequences using invertebrate mitochondrial codons revealed no premature stop codons, confirming the authenticity of the sequences. Phylogenetic analysis using MEGA 11 (Figure 2) further clustered both sequences with *H. tamulus*, verifying appropriate identification.

### 3.3 Gut Microbiome Analysis

**Figure 3:**
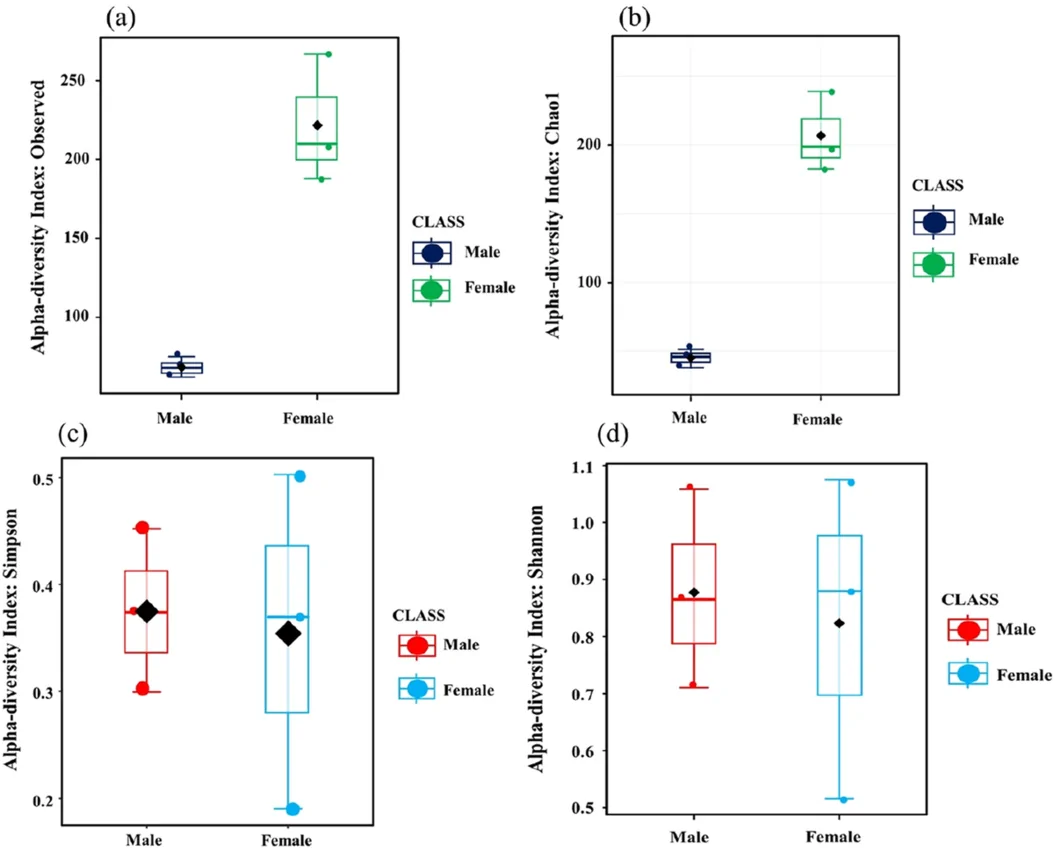
Relative abundance of different bacteria in the gut microbiome of *H. tamulus*. Microbial diversity and richness, represented by Observed (a), Chao1 (b), Simpson (c) and Shannon (d) indices.

The long‐read amplicon sequencing yielded 355,264 high‐quality sequences (approximately 71,052 reads per sample) with a mean length of ~1390 bp. For community profiling, samples were normalized to an average of 20,900 reads/sample, resulting in 154 OTUs at an 85% similarity threshold. Alpha diversity indices, including Observed, Chao1, Simpson, and Shannon indices, revealed moderate taxonomic richness and diversity of the gut bacteria, along with variability among individuals (Figure 3). Female scorpions exhibited markedly higher Observed and Chao1 richness than males, indicating that female scorpions harbor a more diverse microbial community (Figure 3a,b). Moreover, Simpson and Shannon diversity indices consistently indicate that female scorpions possess more diverse and evenly distributed microbial communities, whereas male scorpions display lower diversity and greater dominance of select taxa (Figure 3c,d).

**Figure 4:**
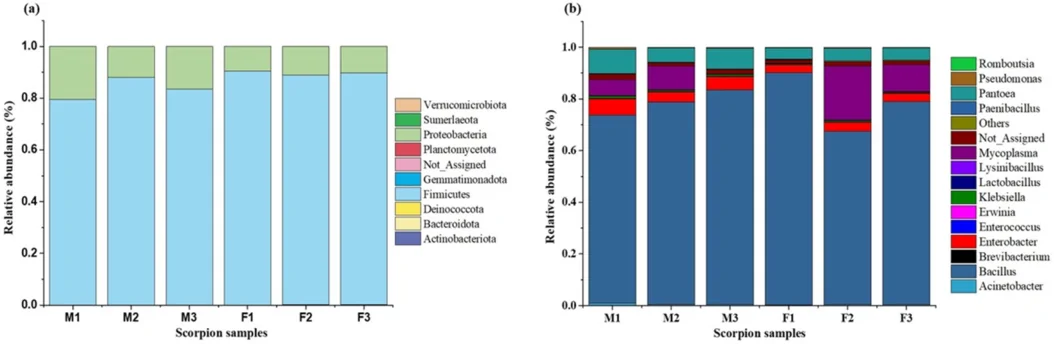
Relative abundance of different bacteria in the gut microbiome of *H. tamulus*. Bar plots illustrate taxonomic distributions at the phylum (a), and genus (b) levels. Individual scorpion samples are labeled on x‐axis (M1‐M3, males; F1‐F3, females) Each color represents a distinct taxon; the height of each block indicates its relative abundance.

The dominant bacteria among the scorpions examined belonged to three of the major taxonomic categories or “Phyla” ‐ specifically Firmicutes (79.48%–90.43%), followed by Proteobacteria (9.46%–20.48%), whereas Actinobacteriota (0.03%–0.11%) and Bacteroidota (0.00%–0.01%) were present at very low relative abundance (Figure 4a). These three groups made up approximately 93% of the 16S rRNA gene sequences obtained from all the scorpion specimens. Other minor phyla present at lower abundances were Clostridiota (formerly Bacteroidetes), Acinetomycota, and Bacteroidota (Bolanos et al. 2019; Elmnasri et al. 2018). Proteobacteria, with subgroups beta‐proteobacteria and gamma‐proteobacteria (e.g., *Acinetobacter* spp. and *Sphingomonas* spp.), represent highly variable populations that respond to environmental conditions in many ways.

**Figure 5:**
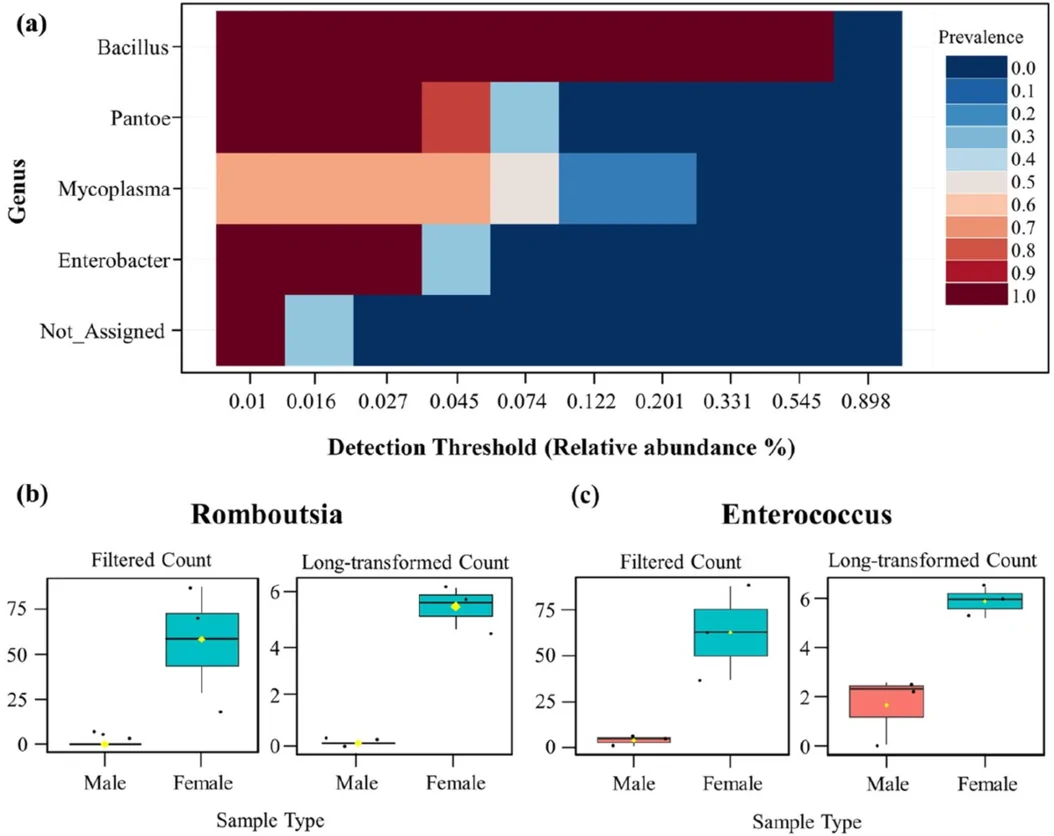
Detection‐threshold‐dependent prevalence of major bacterial genera in the gut microbiota of *H. tamulus* and sex‐associated differences in Enterococcus and Romboutsia abundance. (a) Prevalence heatmap of dominant genera across relative‐abundance thresholds. (b) Boxplots showing filtered and log‐transformed counts of Enterococcus by sex. (c) Boxplots showing filtered and log‐transformed counts of Romboutsia by sex.

When we examine the bacteria at the class level, Bacilli represented ~73.0% of the classes, while gamma‐proteobacteria represented ~13.0% and mollicutes (~7.0%). Some of the orders that contributed significantly to these values include bacillales, enterobacterales, mycoplasmatales, pseudomonadales, and lactobacillales. When we consider the genus‐level diversity of our microbiome dataset, four genera were clearly dominant (*Bacillus*, *Mycoplasma*, *Pantoea*, and *Enterobacter*) and together comprised ~84.1% of all the 16S rRNA genes recovered from our scorpion sample (Figure 4b). While we observed no sexual dimorphism in terms of Firmicutes composition (the relative abundance of Firmicutes was in females = 89.6%; males = 83.6%), there was evidence for sex‐related niche partitioning/host‐associated selective pressures related to the relative abundance of Mycoplasma in female samples (average relative abundance = 10.5%) versus males (average relative abundance = 5.3%). Detection‐threshold‐dependent prevalence of major bacterial genera in the gut microbiota of *H. tamulus* and sex‐associated differences in Enterococcus and Romboutsia abundance. (a) Prevalence heatmap of dominant genera across relative‐abundance thresholds. (b) Boxplots showing filtered and log‐transformed counts of Enterococcus by sex. (c) Boxplots showing filtered and log‐transformed counts of Romboutsia by sex (Figure 5).

**Figure 6:**
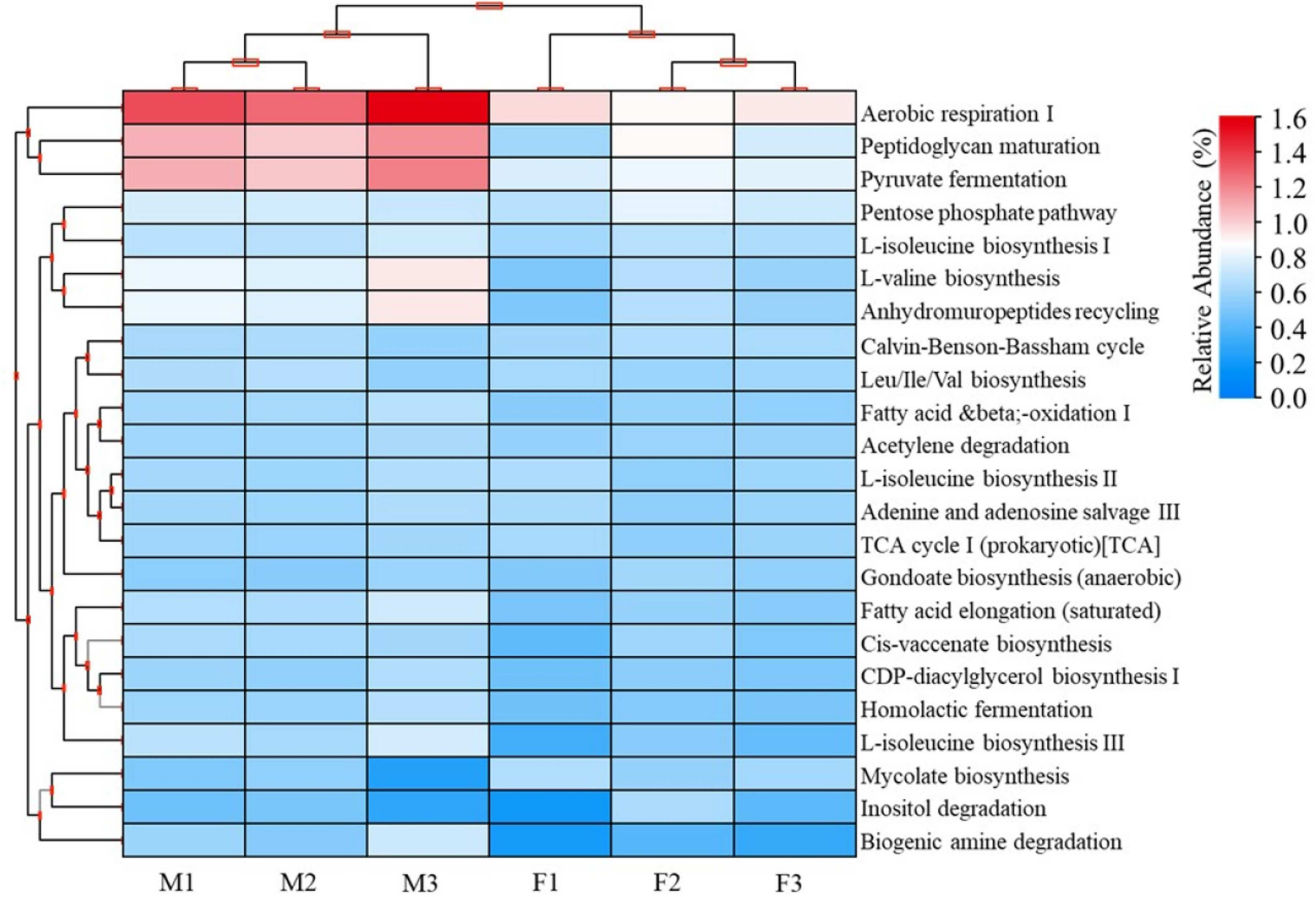
Predicted functional potential of the H. tamulus gut microbiota. The heatmap shows the relative abundance of key bacterial pathways across sample groups (M1–M3 and F1–F3), highlighting clustered variation in metabolic and physiological functions.

Functional prediction analysis was performed using PICRUSt2 based on 16S rRNA sequencing data. It must be emphasized that functional predictions derived from PICRUSt2 are based on 16S rRNA gene profiles and do not represent direct measurements of gene expression or metabolic activity. Therefore, the predictions should be interpreted as indicative rather than definitive and require validation through metagenomic or transcriptomic analyses. A total of 23 enriched MetaCyc pathways were potentially identified within the scorpion gut microbiome. These pathways include energy metabolism, amino acid and lipid biosynthesis, nucleotide metabolism, and the biodegradation of various xenobiotics (Figure 6). The predicted functional profiles were predominantly associated with metabolism, replication, and transcription. Male samples showed enrichment in energy metabolism and biosynthetic pathways, whereas female samples showed greater abundance of lipid metabolism and fatty acid biosynthesis pathways, suggesting potential sex‐related trends that may contribute to functional variation, though they remain hypothesis‐generating due to sample‐size constraints.

## 4 Discussion

The morphological characteristics typically used to identify *Hottentotta tamulus*, such as its black and brown coloration, highly developed and granulate carinae, and terga. A metasoma with the first segment wider than long and characterized by granules, also with sparse hair on the lateral sides of each segment. Furthermore, we found a bulbous, reddish‐colored telson, which contains a curved aculeus. On the ventral side, it contains a triangular‐shaped sternum, and the right and left pectinal teeth were 26 in each (Figure 1). All these characteristics have been described previously as common characteristics for *Hottentotta saulcyi*, which also describe *H. saulcyi* with a very hairy pedipalp, and sparsely hairy metasoma and legs (Karataş and Gharkheloo 2006).

In addition, the sex‐based distinctions can be explained by biological principles. Females of *H. tamulus* are generally wider and thicker than males. Males tend to have a thinner build overall and may display some minor difference in pedipalpal length or metasomal width. This suggests that there is a relationship between body size and sex. External morphology remains an efficient means of identifying scorpion species before any subsequent ecological or microbiome study of these organisms (Kularatne et al. 2015; Monod et al. 2017). Sexual dimorphism has been documented in comparative taxonomic studies of other members of the genus *Tityus pusillus*, another member of the Bothidae family (Lira et al. 2018).

Ranawana et al. (2013) also reported *H. tamulus* with a body length of 50–90 mm from the northern dry zone of Sri Lanka. This species exhibits significant sexual dimorphism in pectinal teeth count, metasoma size, and granules on the movable fingers of the pedipalps. *H. polystictus* is smaller in comparison, with males and females measuring 45 and 55 mm, respectively, and possessing pectinal tooth counts of 22–24 and 18–20, respectively. *H. haudensis* is the smallest in the genus *Hottentotta*, with males measuring 27–31 mm and females measuring 31–33.5 mm; they are characterized by inferior pectinal tooth counts of 16–19 and 14–16, respectively (Kovařík and Lowe 2021).

The application of 650 bp of mitochondrial COI is noteworthy for an accurate phylogenetic analysis within the genus *Hottentotta*, especially when compared with the short sequences (200–300 bp) used for degraded museum specimens (Goodman et al. 2021). The observed nucleotide identities of 96.81% and 96.95% with *H. tamulus* confirmed species‐level identification (Figure 2). The reference sequence utilized (PP755025) belongs to a specimen commercially obtained from an exotic insect market in China, with its precise native location within Subcontent undocumented (Xu et al. 2025). Therefore, approximately 3% genetic divergence strongly indicates geographical isolation and regional variation between our collection and the reference strain within this lineage. Similar patterns of divergence have been observed in other buthid and scorpionid species. For instance, *Scorpio maurus*, once considered monotypic, was reclassified as a species complex following a multi‐locus generic analysis (Talal et al. 2015). High intraspecific variability and deeply divergent lineages are commonly found in scorpion populations isolated through geographical barriers, as in the case of *Alpiscorpius* lineages in the European Alps (Štundlová et al. 2019).

In gut microbiome analysis, long‐read amplicon sequencing yielded approximately 71,052 reads per sample with a mean read length of ~1390 bp. This sequencing volume should be able to capture the vast majority of OTU's; therefore, it is unlikely that increased sampling volume will significantly affect the observed diversity (Ling et al. 2022; Tyagi et al. 2021). The presence of Firmicutes in both the GI tract and gonad indicates that it is likely to be part of a persistent core microbiome that has significant impacts on the physiological processes of the host organism, possibly through the production of various organic acids and/or bacteriocin production. Both of these products can help control the proliferation of pathogenic organisms and also contribute to maintaining a homeostatic balance within the microbial ecosystem (Elmnasri et al. 2018). Members of the Phyla Firmicutes, Proteobacteria, and Tenericutes have also been reported in the gastrointestinal tract and reproductive organs of the various scorpion species, and these members included *Enterococcus*, *Lactobacillus*, *Bacillus, Methylophilus*, and *Mycoplasma* (Bolanos et al. 2019; Elmnasri et al. 2018). More recent studies have shown that proteobacteria may account for > 50% of the bacterial population found in both the telson/venom apparatus of scorpions (Murdoch et al. 2026; Shimwell et al. 2023), indicating that this group may play roles in nutrient cycling, substrate decomposition, etc. Conversely, while female samples had lower amounts of Proteobacteria (relative abundance ~10.3%) than did male samples (relative abundance ~16.3%), these differences were largely due to differences in the levels of each of several different Proteobacteria, including *Pantoea, Enterobacter, Klebsiella, Acinetobacter*, and *Pseudomonas*. Bolaños et al. (2016) used culture‐based techniques to identify some of the same bacterial types found in our study of *Vaejovis smithi* and *Centruroides limpidus* (*Mycoplasma* and *Pseudomonas*); further, they noted that *Mycoplasma* appears to be consistently found in multiple species of scorpions in species‐specific lineages. Further support for this observation comes from Elmnasri et al. (2018), who similarly reported that the majority of microbes in *A. australis* consisted of Firmicutes and Proteobacteria, with Mollicutes being an additional abundant component. Recently, Rampanti et al. (2025) identified *Bacillus* as the most abundant genus in the black scorpion (*Heterometrus longimanus*), mirroring the dominance of *Bacillus* spp. in *H. tamulus*.

The microbial profile of *H. tamulus* was similar to that of other arachnids and insects. *Pardosa* spiders exhibit a similar gut microbial profile, having *Bacillus cereus* and *Enterobacter hormaechei* as the dominant species in their microbiome (Wu et al. 2021). Furthermore, common endosymbiotic genera, such as *Acinetobacter*, *Pseudomonas*, and *Bacillus*, have been reported across various spider families (Kumar et al. 2020; Tyagi et al. 2021) and lepidopterans (Shao et al. 2024). These findings suggest a conserved pattern of gut microbes across diverse predatory arthropods.

At the genus level, *Bacillus, Pantoea, Mycoplasma*, and *Enterobacter* constituted the core microbiota (Figure 5a). *Mycoplasma*s appear to be an indigenous and important component of the scorpion gut microflora. Bolanos et al. (2019) reported *Mycoplasma* in 15 morphospecies of scorpions belonging to the genera *Centruroides*, *Mesomexovis*, *Vaejovis*, and *Diplocentrus* in Mexico; they noted that the lineages appeared to be species‐specific. This suggests a phylogenetic and long‐term evolutionary relationship between scorpions and *Mycoplasma* symbionts. This role of *Mycoplasma* has also been extended to venomous taxa, including several snake families that harbor *Mycoplasma* and account for up to 40% of their respiratory microbiota (Faulhaber et al. 2025).

*Bacillus* was a major constituent of the gut microflora of *H. tamulus* at levels of 17%–30%, and it probably plays an important role as a key endosymbiont by providing support for antioxidant defense systems, maintaining gut barriers and producing vitamins and amino acids *(*Xie et al. 2023 *)*. These results are similar to those previously reported for *Heterometrus longimanus*, where counts were estimated at up to 4 log10 CFU/g (Rampanti et al. 2025), therefore demonstrating that this genus has a large representation within both *Heterometrus longimanus* and *H. tamulus*. While endosymbionts provide significant benefits to their host's fitness, they can also potentially impact their distribution patterns. The presence of *Enterococcus* and *Romboutsia* in the gut of female scorpions was higher when compared to male scorpion gut samples, showing a clear sex‐specific enrichment of each of these genera (Figure 5b,c). Given that *Enterococcus* and *Romboutsia* are intestinal fermenters adapted to nutrient‐rich environments and involved in carbohydrate and bile acid metabolism, their female‐biased abundance suggests that female‐specific physiological or dietary conditions (e.g., reproductive energy demands) may create a more favorable niche for these taxa and could influence host energy harvest and metabolism.

While our predictive PICRUSt2 models highlight various metabolic pathways, directly linking these profiles to active environmental tolerance extends beyond the scope of our current descriptive data (Figure 6). Li et al. (2023) suggested that comparative analysis of microbial distribution and functional potential can provide insights into ecological and evolutionary forces behind host‐microbe associations (Li et al. 2023). In other arthropods, exposure to drought and elevated temperatures, the microbiome has been shown to influence the expression of heat stress‐related genes and modulate host physiological responses to environmental stressors (Chen et al. 2024; Jaramillo and Castañeda 2021). Although shifts in the abundance of Bacteroidetes, Firmicutes, and Proteobacteria correlate with stress response pathways in model invertebrates (Chen et al. 2024), whether *H. tamulus* gut microbiota actively underpins host adaptation to resource‐limited or arid environments remains a compelling hypothesis that requires future empirical testing through targeted survival or environment‐controlled assay. Comprehensive microbiome analyses under targeted or controlled conditions are important to unravel niche specialization, dietary adaptation, and physiological plasticity in animals.

Because the specific transmission routes of these symbionts were not empirically investigated in the present study, the mechanism of microbial acquisition in *H. tamulus* remains entirely speculative. It is a plausible hypothesis for future research that extensive brood care in scorpions may facilitate the social transmission of endosymbiotic bacteria. Specifically, when mothers offer enzymatically treated prey to their offspring. Such a pattern of maternal inheritance has been recorded in other arachnids, such as brown widow spiders, suggesting a potential role for the gut microbiota in the global spread of host species (Mowery et al. 2024).

The alpha diversity indices indicated that male scorpions display lower diversity and greater dominance of select taxa. These results were consistent with those of previous studies on scorpion microbiomes, which reported limited bacterial diversity and recurring dominance by specific phyla. Doniger et al. (2020) also reported on the gut microbiota of the desert scorpion, *Scorpio maurus*, albeit at a lower sequencing depth (approximately 1976 reads per sample). Additionally, *H. tamulus* showed greater microbiome diversity than select social spiders, which display a low mean Shannon index of 0.66 ± 0.71 (standard deviation), well below levels typical of other arthropods (Busck et al. 2022). For example, Liang et al. identified over 2200 bacterial OTUs in unfertilized egg samples and over 26,000 in soil samples (Liang et al. 2019). The moderate level of bacterial richness found in scorpions is significantly greater than that found in spiders, while spiders have a highly specialized and very limited fauna (Mowery et al. 2024; Rampanti et al. 2025; Xie et al. 2023). Sex‐specific variations are also an important factor influencing gut microbiota composition of arachnids. For example, multiple tick studies (e.g., *Dermacentor* spp. and *Amblyomma americanum*) report that male ticks have significantly greater bacterial richness and distinct community composition than females (Briggs et al. 2025; Zhang et al. 2019).

Research on arthropods, including ticks, has shown that females frequently possess more stable and functionally diverse microbial communities, attributable to reproductive investment and extended lifespan, whereas males typically display greater variability associated with increased environmental exposure (Kim et al. 2021). In the wolf spider *Pardosa astrigera*, males showed increased microbial diversity in spring, whereas females showed greater diversity in winter, suggesting that seasonal or physiological conditions influence sex‐dependent variations in the microbiome (Li et al. 2024). Functional prediction analysis predicted 23 enriched MetaCyc pathways within the scorpion gut microbiome, potentially indicating energy metabolism, biomolecular biosynthesis, nucleotide metabolism, and xenobiotic biodegradation. These findings aligned with the results of functional analyses of other arthropod microbiomes, including grasshoppers and spiders. For instance, functional analysis of the microbiome of four grasshopper species revealed that primary metabolic and transport functions (including amino acid, carbohydrate, and energy metabolism), transcription, replication, recombination, and repair were most active in the intestine (Ling et al. 2022). Similarly, predicted functional analysis of the gut microbiota of 35 spider species revealed the enhanced metabolism of amino acids, carbohydrates, vitamins, fatty acids, and lipids (Tyagi et al. 2021).

## 5 Conclusion

The current investigation provides the first baseline characterization of the gut microbiota of *H. tamulus* in northeast Pakistan. The descriptive sequencing data revealed a distinct bacterial community dominated by Firmicutes, Proteobacteria, and Tenericutes with *Bacillus, Pantoea, Mycoplasma*, and *Enterobacter* constituting primary genera. The functional predictive profiling suggested that these microbial communities possess potential contributions to both the host's metabolism and physiological processes, including producing energy, creating essential molecules through biosynthesis, and xenobiotic degradation. Ultimately, these baseline findings identify *Bacillus* and *Mycoplasma* as prime candidates for future research using integrated approaches of metagenomics, transcriptomics, and venom‐associated microbiota to fully understand the functionally relevant aspects of the associations between these scorpions and their microbiota.

## 6 Study Limitations and Future Perspectives

While this study provides the first long‐read 16S rRNA baseline for the gut microbiota of *Hottentotta tamulus*, we acknowledge that our sample size (*n* = 6; 3 males and 3 females) limits our capacity to draw definitive, species‐wide conclusions regarding a permanent core microbiome or robust sex‐specific divergence. Consequently, the observed variations between male and female microbial profiles, as well as the inferred functional pathways related to resource‐limited environments, must be interpreted with caution. These findings are intended as preliminary, hypothesis‐generating observations that lay a critical foundation for future, large‐scale biogeographical and tissue‐specific microbiome investigations within this lineage. These preliminary trends require validation through future large‐scale studies with broader geographic sampling.

## Author Contributions

**Khajid Ullah Khan:** conceptualization, methodology, data curation, investigation, validation, formal analysis, writing – original draft. **Muhammad Tariq Zahid:** conceptualization, methodology, data curation, supervision, formal analysis, validation, investigation, visualization, project administration, resources, writing – review and editing. **Ghulam Mustafa:** conceptualization, methodology, software, data curation, investigation, validation, formal analysis, visualization, writing – original draft. **Rahul S. Tanpure:** conceptualization, methodology, data curation, validation, visualization, formal analysis, writing – review and editing. **Hafiz Muhammad Tahir:** conceptualization, methodology, software, data curation, writing – review and editing, validation. **Ramesh Kumar:** conceptualization, methodology, software, data curation, validation, formal analysis, writing – review and editing, visualization. **Deok‐Won Kim:** conceptualization, investigation, validation, visualization, data curation; formal analysis. **Hyun‐Kyung Park:** conceptualization, data curation, methodology, visualization, validation, writing – review and editing. **Byong‐Hun Jeon:** conceptualization, validation, visualization, writing – review and editing, funding acquisition, software, supervision, data curation.

## Ethics Statement

The authors have nothing to report.

## Conflicts of Interest

The authors declare no conflicts of interest.

## Supporting information


Supporting File

## Data Availability

Data sharing not applicable to this article as no datasets were generated or analyzed during the current study. No data was used for the research described in the article.
