# Supplementary material for: Gut Microbiome Diversity, Functional Potential, and Ecological Relevance of Hottentotta tamulus
Source: Microbiologyopen. 2026 Sep 1;15(5):e70373. doi: 10.1002/mbo3.70373 (PMC13535146; doi:10.1002/mbo3.70373)
Supplement: Supplementary file 1 [file MBO3-15-e70373-s001.docx]

**Gut Microbiome Diversity, Functional Potential, and Ecological Relevance of Hottentotta tamulus**

Khajid Ullah Khan^1^, Muhammad Tariq Zahid^1*^, Ghulam Mustafa^2^, Rahul S Tanpure^2^, Hafiz Muhammad Tahir^1^, Ramesh Kumar^2^, Deok-Won Kim^2^, Hyun-Kyung Park^3^, Byong-Hun Jeon^2^*

1 Department of Zoology, Government College University Lahore, Pakistan

2 Department of Earth Resources & Environmental Engineering, Hanyang University, 222-Wangsimni-ro, Seongdong-gu, Seoul 04763, Republic of Korea

3 Department of Pediatrics, Hanyang University College of Medicine, Seoul, Republic of Korea

*Corresponding authors,

Email Ids: [mtariqzahid@gcu.edu.pk](mailto:mtariqzahid@gcu.edu.pk) (M.T. Zahid); [bhjeon@hanyang.ac.kr](mailto:bhjeon@hanyang.ac.kr) (B-H. Jeon)

Table 1S. Taxonomic composition of the negative control sample showing the detected OTUs and their corresponding read counts, as constructed from the BIOM file.

| # Constructed from biom file |  |
| --- | --- |
| #OTU ID | Negative control |
| d__Bacteria;p__Firmicutes;c__Bacilli;o__Lactobacillales;f__Streptococcaceae;g__Streptococcus;s__Streptococcus_anginosus | 132 |
| Unassigned;__;__;__;__;__;__ | 4 |
| d__Bacteria;p__Firmicutes;c__Clostridia;o__Clostridiales;f__Clostridiaceae;g__Clostridium_sensu_stricto_1;__ | 3 |
| d__Bacteria;p__Firmicutes;c__Bacilli;o__Lactobacillales;f__Enterococcaceae;g__Enterococcus;s__Enterococcus_faecalis | 3 |
| d__Bacteria;p__Actinobacteriota;c__Actinobacteria;o__Propionibacteriales;f__Propionibacteriaceae;g__Cutibacterium;s__Cutibacterium_acnes | 3 |
| d__Bacteria;p__Firmicutes;c__Bacilli;o__Lactobacillales;f__Enterococcaceae;g__Enterococcus;s__Enterococcus_faecium | 2 |
| d__Bacteria;p__Firmicutes;c__Bacilli;o__Staphylococcales;f__Staphylococcaceae;g__Staphylococcus;s__Staphylococcus_epidermidis | 2 |
| d__Bacteria;p__Firmicutes;c__Clostridia;o__Lachnospirales;f__Lachnospiraceae;g__Hungatella;__ | 2 |
| d__Bacteria;p__Firmicutes;c__Bacilli;o__Lactobacillales;f__Streptococcaceae;g__Streptococcus;s__Streptococcus_agalactiae | 2 |
| d__Bacteria;p__Proteobacteria;c__Gammaproteobacteria;o__Enterobacterales;f__Enterobacteriaceae;g__Klebsiella;__ | 1 |
| d__Bacteria;p__Firmicutes;c__Bacilli;o__Staphylococcales;f__Staphylococcaceae;g__Staphylococcus;s__Staphylococcus_capitis | 1 |
| d__Bacteria;p__Firmicutes;c__Bacilli;o__Lactobacillales;f__Lactobacillaceae;g__Lactobacillus;s__Lactobacillus_crispatus | 1 |
| d__Bacteria;p__Proteobacteria;c__Gammaproteobacteria;o__Burkholderiales;f__Neisseriaceae;g__uncultured;s__uncultured_bacterium | 1 |
| d__Bacteria;p__Firmicutes;c__Bacilli;o__Lactobacillales;f__Streptococcaceae;g__Streptococcus;s__Streptococcus_salivarius | 0 |
| d__Bacteria;p__Firmicutes;c__Bacilli;o__Lactobacillales;f__Streptococcaceae;g__Streptococcus;__ | 0 |
| d__Bacteria;p__Proteobacteria;c__Gammaproteobacteria;o__Enterobacterales;f__Enterobacteriaceae;g__Klebsiella;s__Klebsiella_aerogenes | 0 |
| d__Bacteria;p__Firmicutes;c__Bacilli;o__Lactobacillales;f__Enterococcaceae;g__Enterococcus;s__Enterococcus_durans | 0 |
| d__Bacteria;p__Firmicutes;c__Clostridia;o__Peptostreptococcales-Tissierellales;f__Peptostreptococcaceae;g__Clostridioides;s__Clostridioides_difficile | 0 |
| d__Bacteria;p__Firmicutes;c__Clostridia;o__Clostridiales;f__Clostridiaceae;g__Clostridium_sensu_stricto_1;s__uncultured_bacterium | 0 |
| d__Bacteria;p__Firmicutes;c__Bacilli;o__Staphylococcales;f__Staphylococcaceae;g__Staphylococcus;s__Staphylococcus_haemolyticus | 0 |
| d__Bacteria;p__Firmicutes;c__Bacilli;o__Lactobacillales;f__Enterococcaceae;g__Enterococcus;__ | 0 |
| d__Bacteria;p__Firmicutes;c__Bacilli;o__Staphylococcales;f__Staphylococcaceae;g__Staphylococcus;__ | 0 |
| d__Bacteria;p__Proteobacteria;c__Gammaproteobacteria;o__Enterobacterales;f__Enterobacteriaceae;g__Klebsiella;s__Klebsiella_pneumoniae | 0 |
| d__Bacteria;p__Firmicutes;c__Bacilli;o__Lactobacillales;f__Lactobacillaceae;g__Lacticaseibacillus;s__Lactobacillus_rhamnosus | 0 |
| d__Bacteria;p__Proteobacteria;c__Gammaproteobacteria;o__Enterobacterales;f__Enterobacteriaceae;__;__ | 0 |
| d__Bacteria;p__Proteobacteria;c__Gammaproteobacteria;o__Enterobacterales;f__Enterobacteriaceae;g__Escherichia-Shigella;s__Escherichia_coli | 0 |
| d__Bacteria;p__Firmicutes;c__Clostridia;o__Peptostreptococcales-Tissierellales;f__Family_XI;g__Finegoldia;s__Finegoldia_magna | 0 |
| d__Bacteria;p__Firmicutes;c__Clostridia;o__Oscillospirales;f__Ruminococcaceae;g__Faecalibacterium;__ | 0 |
| d__Bacteria;p__Firmicutes;c__Clostridia;o__Clostridiales;f__Clostridiaceae;g__Clostridium_sensu_stricto_1;s__Clostridium_butyricum | 0 |
| d__Bacteria;p__Firmicutes;c__Bacilli;o__Lactobacillales;f__Lactobacillaceae;g__Lactobacillus;s__Lactobacillus_gasseri | 0 |
| d__Bacteria;p__Firmicutes;c__Clostridia;o__Clostridiales;f__Clostridiaceae;g__Clostridium_sensu_stricto_1;s__uncultured_organism | 0 |
| d__Bacteria;p__Proteobacteria;c__Gammaproteobacteria;o__Enterobacterales;f__Enterobacteriaceae;g__Escherichia-Shigella;s__uncultured_bacterium | 0 |
| d__Bacteria;p__Proteobacteria;c__Gammaproteobacteria;o__Pseudomonadales;f__Moraxellaceae;g__Enhydrobacter;s__Moraxella_osloensis | 0 |
| d__Bacteria;p__Firmicutes;c__Bacilli;o__Lactobacillales;f__Streptococcaceae;g__Streptococcus;s__Streptococcus_mitis | 0 |
| d__Bacteria;p__Firmicutes;c__Clostridia;o__Oscillospirales;f__Ruminococcaceae;g__Ruminococcus;s__uncultured_bacterium | 0 |
| d__Bacteria;p__Firmicutes;c__Bacilli;o__Lactobacillales;f__Lactobacillaceae;g__Lactobacillus;s__Lactobacillus_jensenii | 0 |
| d__Bacteria;p__Firmicutes;c__Bacilli;o__Lactobacillales;f__Streptococcaceae;g__Streptococcus;s__Streptococcus_pneumoniae | 0 |
| d__Bacteria;p__Firmicutes;c__Clostridia;o__Lachnospirales;f__Lachnospiraceae;g__Hungatella;s__uncultured_organism | 0 |
| d__Bacteria;p__Proteobacteria;c__Gammaproteobacteria;o__Enterobacterales;f__Pasteurellaceae;g__Haemophilus;s__Haemophilus_parainfluenzae | 0 |
| d__Bacteria;p__Firmicutes;c__Bacilli;o__Staphylococcales;f__Staphylococcaceae;g__Staphylococcus;s__uncultured_Staphylococcus | 0 |
| d__Bacteria;p__Firmicutes;c__Bacilli;__;__;__;__ | 0 |
| d__Bacteria;p__Firmicutes;c__Bacilli;o__Lactobacillales;f__Lactobacillaceae;g__Lactobacillus;__ | 0 |
| d__Bacteria;p__Firmicutes;c__Bacilli;o__Erysipelotrichales;f__Erysipelatoclostridiaceae;g__Erysipelatoclostridium;__ | 0 |
| d__Bacteria;p__Firmicutes;c__Bacilli;o__Lactobacillales;f__Lactobacillaceae;g__Lactobacillus;s__uncultured_bacterium | 0 |
| d__Bacteria;p__Firmicutes;c__Clostridia;o__Clostridiales;f__Clostridiaceae;g__Clostridium_sensu_stricto_1;s__Clostridium_perfringens | 0 |
| d__Bacteria;p__Firmicutes;c__Bacilli;o__Lactobacillales;f__Streptococcaceae;g__Streptococcus;s__Streptococcus_sp. | 0 |
| d__Bacteria;p__Firmicutes;c__Clostridia;o__Lachnospirales;f__Lachnospiraceae;g__[Eubacterium]_eligens_group;__ | 0 |
| d__Bacteria;p__Verrucomicrobiota;c__Verrucomicrobiae;o__Verrucomicrobiales;f__Akkermansiaceae;g__Akkermansia;s__uncultured_bacterium | 0 |
| d__Bacteria;p__Firmicutes;c__Clostridia;o__Lachnospirales;f__Lachnospiraceae;g__Lachnospiraceae_UCG-008;s__uncultured_bacterium | 0 |
| d__Bacteria;p__Firmicutes;c__Clostridia;o__Lachnospirales;f__Lachnospiraceae;g__Anaerostipes;s__Anaerostipes_hadrus | 0 |
| d__Bacteria;p__Firmicutes;c__Clostridia;o__Lachnospirales;f__Lachnospiraceae;g__Lachnospiraceae_UCG-004;s__uncultured_bacterium | 0 |
| d__Bacteria;p__Firmicutes;c__Negativicutes;o__Veillonellales-Selenomonadales;f__Veillonellaceae;g__Veillonella;s__Veillonella_parvula | 0 |
| d__Bacteria;p__Firmicutes;c__Bacilli;o__Lactobacillales;f__Lactobacillaceae;g__Limosilactobacillus;s__uncultured_bacterium | 0 |
| d__Bacteria;p__Firmicutes;c__Clostridia;o__Lachnospirales;f__Lachnospiraceae;g__Fusicatenibacter;s__uncultured_bacterium | 0 |
| d__Bacteria;p__Firmicutes;c__Clostridia;o__Lachnospirales;f__Lachnospiraceae;g__Lachnoclostridium;s__uncultured_bacterium | 0 |
| d__Bacteria;p__Firmicutes;c__Bacilli;o__Lactobacillales;f__Enterococcaceae;g__Enterococcus;s__Enterococcus_hirae | 0 |
| d__Bacteria;p__Firmicutes;c__Clostridia;o__Lachnospirales;f__Lachnospiraceae;g__Anaerostipes;__ | 0 |
| d__Bacteria;p__Firmicutes;c__Clostridia;o__Lachnospirales;f__Lachnospiraceae;g__Blautia;s__uncultured_bacterium | 0 |
| d__Bacteria;p__Firmicutes;c__Negativicutes;o__Veillonellales-Selenomonadales;f__Veillonellaceae;g__Veillonella;__ | 0 |
| d__Bacteria;p__Firmicutes;c__Clostridia;o__Lachnospirales;f__Lachnospiraceae;g__[Ruminococcus]_gnavus_group;__ | 0 |
| d__Bacteria;p__Proteobacteria;c__Gammaproteobacteria;o__Enterobacterales;f__Enterobacteriaceae;g__Enterobacter;s__Klebsiella_oxytoca | 0 |
| d__Bacteria;p__Actinobacteriota;c__Actinobacteria;o__Actinomycetales;f__Actinomycetaceae;g__Actinomyces;__ | 0 |
| d__Bacteria;p__Firmicutes;c__Clostridia;o__Oscillospirales;f__Ruminococcaceae;g__Faecalibacterium;s__uncultured_bacterium | 0 |
| d__Bacteria;p__Firmicutes;c__Bacilli;o__Lactobacillales;f__Lactobacillaceae;__;__ | 0 |
| d__Bacteria;p__Firmicutes;c__Clostridia;o__Peptostreptococcales-Tissierellales;f__Family_XI;g__Anaerococcus;__ | 0 |
| d__Bacteria;p__Firmicutes;c__Bacilli;o__Staphylococcales;f__Staphylococcaceae;g__Staphylococcus;s__Staphylococcus_aureus | 0 |
| d__Bacteria;p__Proteobacteria;c__Gammaproteobacteria;o__Enterobacterales;f__Enterobacteriaceae;g__Escherichia-Shigella;s__Citrobacter_koseri | 0 |
| d__Bacteria;p__Proteobacteria;c__Gammaproteobacteria;o__Enterobacterales;f__Enterobacteriaceae;g__Enterobacter;__ | 0 |
| d__Bacteria;p__Proteobacteria;c__Gammaproteobacteria;o__Enterobacterales;f__Enterobacteriaceae;g__Enterobacter;s__Enterobacter_cloacae | 0 |
| d__Bacteria;p__Cyanobacteria;c__Cyanobacteriia;o__Chloroplast;f__Chloroplast;g__Chloroplast;s__uncultured_bacterium | 0 |
| d__Bacteria;p__Proteobacteria;c__Alphaproteobacteria;o__Sphingomonadales;f__Sphingomonadaceae;g__Sphingomonas;__ | 0 |
| d__Bacteria;p__Firmicutes;__;__;__;__;__ | 0 |
| d__Bacteria;p__Firmicutes;c__Bacilli;o__Lactobacillales;f__Enterococcaceae;g__Enterococcus;s__Enterococcus_gilvus | 0 |
| d__Bacteria;p__Firmicutes;c__Bacilli;o__Lactobacillales;f__Streptococcaceae;g__Streptococcus;s__uncultured_bacterium | 0 |
| d__Bacteria;p__Firmicutes;c__Clostridia;o__Peptostreptococcales-Tissierellales;f__Peptostreptococcaceae;g__Romboutsia;s__uncultured_bacterium | 0 |
| d__Bacteria;p__Proteobacteria;c__Alphaproteobacteria;o__Rhizobiales;f__Rhizobiaceae;g__Allorhizobium-Neorhizobium-Pararhizobium-Rhizobium;s__Agrobacterium_radiobacter | 0 |
| d__Bacteria;p__Firmicutes;c__Clostridia;o__Peptostreptococcales-Tissierellales;f__Peptostreptococcaceae;g__Romboutsia;__ | 0 |
| d__Bacteria;p__Proteobacteria;c__Gammaproteobacteria;o__Xanthomonadales;f__Xanthomonadaceae;g__Pseudoxanthomonas;__ | 0 |
| d__Bacteria;p__Proteobacteria;c__Gammaproteobacteria;o__Pseudomonadales;f__Pseudomonadaceae;g__Pseudomonas;s__Pseudomonas_balearica | 0 |
| d__Bacteria;p__Firmicutes;c__Bacilli;o__Alicyclobacillales;f__Alicyclobacillaceae;g__Tumebacillus;s__uncultured_bacterium | 0 |
| d__Bacteria;p__Actinobacteriota;c__Acidimicrobiia;o__Microtrichales;f__uncultured;g__uncultured;s__uncultured_bacterium | 0 |
| d__Bacteria;p__Acidobacteriota;c__Acidobacteriae;o__Acidobacteriales;f__Acidobacteriaceae_(Subgroup_1);g__Acidipila-Silvibacterium;s__uncultured_bacterium | 0 |
| d__Bacteria;p__Proteobacteria;c__Alphaproteobacteria;o__Micropepsales;f__Micropepsaceae;g__uncultured;s__uncultured_bacterium | 0 |
| d__Bacteria;p__Proteobacteria;c__Gammaproteobacteria;o__Xanthomonadales;f__Rhodanobacteraceae;g__Chujaibacter;__ | 0 |
| d__Bacteria;p__Proteobacteria;c__Alphaproteobacteria;o__Rhizobiales;f__Hyphomicrobiaceae;g__Pedomicrobium;s__uncultured_bacterium | 0 |
| d__Bacteria;p__Acidobacteriota;c__Acidobacteriae;o__Acidobacteriales;f__Acidobacteriaceae_(Subgroup_1);g__uncultured;__ | 0 |
| d__Bacteria;p__Proteobacteria;c__Gammaproteobacteria;o__Gammaproteobacteria_Incertae_Sedis;f__Unknown_Family;g__Acidibacter;__ | 0 |
| d__Bacteria;p__Proteobacteria;c__Gammaproteobacteria;o__Burkholderiales;f__Oxalobacteraceae;g__Massilia;__ | 0 |
| d__Bacteria;p__Acidobacteriota;c__Acidobacteriae;o__Bryobacterales;f__Bryobacteraceae;g__Bryobacter;s__uncultured_bacterium | 0 |
| d__Bacteria;p__Actinobacteriota;c__Actinobacteria;o__Micrococcales;f__Microbacteriaceae;g__Humibacter;__ | 0 |
| d__Bacteria;p__Proteobacteria;c__Alphaproteobacteria;o__Caulobacterales;f__Caulobacteraceae;g__Brevundimonas;s__Brevundimonas_sp. | 0 |
| d__Bacteria;p__Firmicutes;c__Bacilli;o__Bacillales;f__Planococcaceae;g__Paenisporosarcina;s__Paenisporosarcina_sp. | 0 |
| d__Bacteria;p__Proteobacteria;c__Alphaproteobacteria;o__Rhizobiales;f__Xanthobacteraceae;g__uncultured;s__uncultured_bacterium | 0 |
| d__Bacteria;p__Proteobacteria;c__Alphaproteobacteria;o__Rhizobiales;f__Xanthobacteraceae;g__Bradyrhizobium;s__Bradyrhizobium_japonicum | 0 |
| d__Bacteria;p__Firmicutes;c__Clostridia;o__Peptostreptococcales-Tissierellales;f__Family_XI;g__Tepidimicrobium;s__uncultured_bacterium | 0 |
| d__Bacteria;p__Proteobacteria;c__Gammaproteobacteria;o__Burkholderiales;f__Oxalobacteraceae;g__Noviherbaspirillum;__ | 0 |
| d__Bacteria;p__Cyanobacteria;c__Sericytochromatia;o__Sericytochromatia;f__Sericytochromatia;g__Sericytochromatia;s__uncultured_bacterium | 0 |
| d__Bacteria;p__Firmicutes;c__Clostridia;o__Lachnospirales;f__Lachnospiraceae;__;__ | 0 |
| d__Bacteria;p__Verrucomicrobiota;c__Verrucomicrobiae;o__Opitutales;f__Opitutaceae;g__Opitutus;__ | 0 |
| d__Bacteria;p__Actinobacteriota;c__Thermoleophilia;o__Gaiellales;f__uncultured;g__uncultured;s__uncultured_bacterium | 0 |
| d__Bacteria;p__Firmicutes;c__Bacilli;o__Paenibacillales;f__Paenibacillaceae;g__Cohnella;__ | 0 |
| d__Bacteria;p__Firmicutes;c__Bacilli;o__Bacillales;f__Bacillaceae;g__Bacillus;__ | 0 |
| d__Bacteria;p__Proteobacteria;c__Alphaproteobacteria;o__Rhizobiales;f__Rhizobiaceae;g__Mesorhizobium;__ | 0 |
| d__Bacteria;p__Proteobacteria;c__Gammaproteobacteria;o__Xanthomonadales;f__Xanthomonadaceae;g__Luteimonas;__ | 0 |
| d__Bacteria;p__Bacteroidota;c__Bacteroidia;o__Chitinophagales;f__Chitinophagaceae;g__Flavihumibacter;__ | 0 |
| d__Bacteria;p__Firmicutes;c__Clostridia;o__Peptostreptococcales-Tissierellales;f__Peptostreptococcaceae;g__Terrisporobacter;s__uncultured_bacterium | 0 |
| d__Bacteria;p__Proteobacteria;c__Gammaproteobacteria;o__Burkholderiales;f__Alcaligenaceae;g__Castellaniella;__ | 0 |
| d__Bacteria;p__Proteobacteria;c__Alphaproteobacteria;o__Rhizobiales;f__Xanthobacteraceae;g__Bradyrhizobium;__ | 0 |
| d__Bacteria;p__Sumerlaeota;c__Sumerlaeia;o__Sumerlaeales;f__Sumerlaeaceae;g__Sumerlaea;s__uncultured_bacterium | 0 |
| d__Bacteria;p__Firmicutes;c__Bacilli;o__Bacillales;f__Bacillaceae;g__Bacillus;s__uncultured_Bacillus | 0 |
| d__Bacteria;p__Firmicutes;c__Bacilli;o__Aneurinibacillales;f__Aneurinibacillaceae;g__Aneurinibacillus;__ | 0 |
| d__Bacteria;p__Proteobacteria;c__Gammaproteobacteria;o__Pseudomonadales;f__Pseudomonadaceae;g__Pseudomonas;s__Pseudomonas_stutzeri | 0 |
| d__Bacteria;p__Proteobacteria;c__Gammaproteobacteria;o__Xanthomonadales;f__Rhodanobacteraceae;g__Dyella;__ | 0 |
| d__Bacteria;p__Proteobacteria;c__Gammaproteobacteria;o__Xanthomonadales;f__Rhodanobacteraceae;g__Rhodanobacter;__ | 0 |
| d__Bacteria;p__Proteobacteria;c__Gammaproteobacteria;o__Burkholderiales;f__Oxalobacteraceae;g__Noviherbaspirillum;s__uncultured_bacterium | 0 |
| d__Bacteria;p__Proteobacteria;c__Alphaproteobacteria;o__Caulobacterales;f__Caulobacteraceae;g__Phenylobacterium;s__uncultured_bacterium | 0 |
| d__Bacteria;p__Firmicutes;c__Clostridia;o__Clostridiales;f__Clostridiaceae;g__Clostridium_sensu_stricto_1;s__Clostridium_saccharoperbutylacetonicum | 0 |
| d__Bacteria;p__Proteobacteria;c__Alphaproteobacteria;o__Rhizobiales;f__Xanthobacteraceae;__;__ | 0 |
| d__Bacteria;p__Bdellovibrionota;c__Bdellovibrionia;o__Bdellovibrionales;f__Bdellovibrionaceae;g__Bdellovibrio;__ | 0 |
| d__Bacteria;p__Bacteroidota;c__Bacteroidia;o__Chitinophagales;f__Chitinophagaceae;g__Parasegetibacter;__ | 0 |
| d__Bacteria;p__Firmicutes;c__Clostridia;o__Lachnospirales;f__Lachnospiraceae;g__Roseburia;__ | 0 |
| d__Bacteria;p__Firmicutes;c__Bacilli;o__Bacillales;f__Bacillaceae;g__Bacillus;s__Bacillus_circulans | 0 |
| d__Bacteria;p__Proteobacteria;c__Gammaproteobacteria;o__Xanthomonadales;f__Xanthomonadaceae;g__Thermomonas;__ | 0 |
| d__Bacteria;p__Proteobacteria;c__Alphaproteobacteria;o__Elsterales;f__uncultured;g__uncultured;__ | 0 |
| d__Bacteria;p__Proteobacteria;c__Gammaproteobacteria;o__Burkholderiales;f__Burkholderiaceae;g__Burkholderia-Caballeronia-Paraburkholderia;__ | 0 |
| d__Bacteria;p__Proteobacteria;c__Alphaproteobacteria;o__Rhizobiales;f__Hyphomicrobiaceae;g__Hyphomicrobium;__ | 0 |
| d__Bacteria;p__Proteobacteria;c__Alphaproteobacteria;o__Caulobacterales;f__Caulobacteraceae;g__Brevundimonas;__ | 0 |
| d__Bacteria;p__Proteobacteria;c__Gammaproteobacteria;o__Burkholderiales;f__Burkholderiaceae;g__Burkholderia-Caballeronia-Paraburkholderia;s__Burkholderia_cenocepacia | 0 |
| d__Bacteria;p__Myxococcota;c__Polyangia;o__Haliangiales;f__Haliangiaceae;g__Haliangium;__ | 0 |
| d__Bacteria;p__Acidobacteriota;c__Acidobacteriae;o__Bryobacterales;f__Bryobacteraceae;g__Bryobacter;__ | 0 |
| d__Bacteria;p__Planctomycetota;c__Planctomycetes;o__Isosphaerales;f__Isosphaeraceae;g__Paludisphaera;__ | 0 |
| d__Bacteria;p__Bacteroidota;c__Bacteroidia;o__Chitinophagales;f__Chitinophagaceae;g__Terrimonas;s__uncultured_bacterium | 0 |
| d__Bacteria;p__Firmicutes;c__Bacilli;o__Alicyclobacillales;f__Alicyclobacillaceae;g__Tumebacillus;__ | 0 |
| d__Bacteria;p__Proteobacteria;c__Gammaproteobacteria;o__Pseudomonadales;f__Pseudomonadaceae;g__Pseudomonas;__ | 0 |
| d__Bacteria;p__Firmicutes;c__Bacilli;o__Paenibacillales;f__Paenibacillaceae;g__Paenibacillus;__ | 0 |
| d__Bacteria;p__Firmicutes;c__Bacilli;o__Bacillales;f__Bacillaceae;g__Bacillus;s__Bacillus_megaterium | 0 |
| d__Bacteria;p__Proteobacteria;c__Alphaproteobacteria;o__Rhizobiales;f__Xanthobacteraceae;g__Pseudolabrys;__ | 0 |
| d__Bacteria;p__Planctomycetota;c__Planctomycetes;o__Isosphaerales;f__Isosphaeraceae;g__Aquisphaera;__ | 0 |
| d__Bacteria;p__Proteobacteria;c__Alphaproteobacteria;o__Rhizobiales;f__Xanthobacteraceae;g__Pseudolabrys;s__uncultured_bacterium | 0 |
| d__Bacteria;p__Firmicutes;c__Clostridia;o__Lachnospirales;f__Lachnospiraceae;g__Herbinix;__ | 0 |
| d__Bacteria;p__Planctomycetota;c__Planctomycetes;o__Isosphaerales;f__Isosphaeraceae;g__uncultured;__ | 0 |
| d__Bacteria;p__Firmicutes;c__Bacilli;o__Alicyclobacillales;f__Alicyclobacillaceae;g__Tumebacillus;s__Tumebacillus_avium | 0 |
| d__Bacteria;p__Proteobacteria;c__Alphaproteobacteria;o__Rhizobiales;f__Rhizobiaceae;g__Pseudaminobacter;__ | 0 |
| d__Bacteria;p__Firmicutes;c__Bacilli;o__Alicyclobacillales;f__Alicyclobacillaceae;g__Tumebacillus;s__uncultured_soil | 0 |
| d__Bacteria;p__Firmicutes;c__Bacilli;o__Paenibacillales;f__Paenibacillaceae;g__Paenibacillus;s__Paenibacillus_barengoltzii | 0 |
| d__Bacteria;p__Proteobacteria;c__Gammaproteobacteria;o__Coxiellales;f__Coxiellaceae;g__Coxiella;s__uncultured_bacterium | 0 |
| d__Bacteria;p__Proteobacteria;c__Gammaproteobacteria;o__Burkholderiales;f__Comamonadaceae;g__Ramlibacter;__ | 0 |
| d__Bacteria;p__Firmicutes;c__Clostridia;o__Peptostreptococcales-Tissierellales;f__Proteiniboraceae;g__Proteiniborus;s__uncultured_bacterium | 0 |
| d__Bacteria;p__Firmicutes;c__Clostridia;o__Peptostreptococcales-Tissierellales;f__Peptostreptococcaceae;g__Romboutsia;s__Romboutsia_ilealis | 0 |
| d__Bacteria;p__Proteobacteria;c__Gammaproteobacteria;o__Burkholderiales;f__Oxalobacteraceae;g__Massilia;s__uncultured_bacterium | 0 |
| d__Bacteria;p__Chloroflexi;c__Chloroflexia;o__Thermomicrobiales;f__JG30-KF-CM45;g__JG30-KF-CM45;s__uncultured_bacterium | 0 |
| d__Bacteria;p__Proteobacteria;c__Alphaproteobacteria;o__Rhizobiales;f__Hyphomicrobiaceae;g__Hyphomicrobium;s__uncultured_bacterium | 0 |
| d__Bacteria;p__Acidobacteriota;c__Acidobacteriae;o__Acidobacteriales;f__uncultured;g__uncultured;__ | 0 |
| d__Bacteria;p__Proteobacteria;c__Alphaproteobacteria;o__Sphingomonadales;f__Sphingomonadaceae;g__Altererythrobacter;s__uncultured_bacterium | 0 |
| d__Bacteria;p__Firmicutes;c__Limnochordia;o__Limnochordales;f__Limnochordaceae;g__Limnochordaceae;__ | 0 |
| d__Bacteria;p__Planctomycetota;c__Planctomycetes;o__Isosphaerales;f__Isosphaeraceae;__;__ | 0 |
| d__Bacteria;p__Proteobacteria;c__Alphaproteobacteria;o__Sphingomonadales;f__Sphingomonadaceae;g__Sphingomonas;s__uncultured_bacterium | 0 |
| d__Bacteria;p__Proteobacteria;c__Gammaproteobacteria;o__Diplorickettsiales;f__Diplorickettsiaceae;g__Aquicella;__ | 0 |
| d__Bacteria;p__Chloroflexi;c__Ktedonobacteria;o__Ktedonobacterales;f__Ktedonobacteraceae;__;__ | 0 |
| d__Bacteria;p__Proteobacteria;c__Gammaproteobacteria;o__Xanthomonadales;f__Xanthomonadaceae;g__Lysobacter;__ | 0 |
| d__Bacteria;p__Actinobacteriota;c__Actinobacteria;o__Propionibacteriales;f__Nocardioidaceae;g__Nocardioides;s__Nocardioides_sp. | 0 |
| d__Bacteria;p__Firmicutes;c__Clostridia;o__Clostridiales;f__Clostridiaceae;g__Clostridium_sensu_stricto_6;__ | 0 |
| d__Bacteria;p__Proteobacteria;c__Alphaproteobacteria;o__Rhizobiales;f__Rhizobiaceae;__;__ | 0 |
| d__Bacteria;p__Actinobacteriota;c__Actinobacteria;o__Propionibacteriales;f__Nocardioidaceae;g__Nocardioides;__ | 0 |
| d__Bacteria;p__Proteobacteria;c__Gammaproteobacteria;o__Xanthomonadales;f__Xanthomonadaceae;g__Thermomonas;s__uncultured_bacterium | 0 |
| d__Bacteria;p__Proteobacteria;c__Gammaproteobacteria;o__Burkholderiales;f__TRA3-20;g__TRA3-20;s__uncultured_bacterium | 0 |
| d__Bacteria;p__Proteobacteria;c__Gammaproteobacteria;o__JG36-TzT-191;f__JG36-TzT-191;g__JG36-TzT-191;__ | 0 |
| d__Bacteria;p__Planctomycetota;c__Planctomycetes;o__Gemmatales;f__Gemmataceae;g__uncultured;__ | 0 |
| d__Bacteria;p__Proteobacteria;c__Gammaproteobacteria;o__Burkholderiales;f__Methylophilaceae;g__Methylophilus;__ | 0 |
| d__Bacteria;p__Proteobacteria;c__Gammaproteobacteria;o__Burkholderiales;f__Rhodocyclaceae;g__Methyloversatilis;__ | 0 |
| d__Bacteria;p__Firmicutes;c__Bacilli;o__Bacillales;f__Bacillaceae;g__Bacillus;s__Bacillus_thermolactis | 0 |
| d__Bacteria;p__Bacteroidota;c__Bacteroidia;o__Chitinophagales;f__Chitinophagaceae;g__Niastella;__ | 0 |
| d__Bacteria;p__Patescibacteria;c__Saccharimonadia;o__Saccharimonadales;f__Saccharimonadales;g__Saccharimonadales;__ | 0 |
| d__Bacteria;p__Patescibacteria;c__Saccharimonadia;o__Saccharimonadales;f__LWQ8;g__LWQ8;__ | 0 |
| d__Bacteria;p__Actinobacteriota;c__Actinobacteria;o__Micrococcales;f__Micrococcaceae;g__Pseudarthrobacter;__ | 0 |
| d__Bacteria;p__Actinobacteriota;c__Actinobacteria;o__Micrococcales;f__Microbacteriaceae;g__Agrococcus;__ | 0 |
| d__Bacteria;p__Acidobacteriota;c__Acidobacteriae;o__Acidobacteriales;f__Acidobacteriaceae_(Subgroup_1);g__Acidipila-Silvibacterium;__ | 0 |
| d__Bacteria;p__Proteobacteria;c__Alphaproteobacteria;o__Rhizobiales;f__Devosiaceae;g__Devosia;__ | 0 |
| d__Bacteria;p__Firmicutes;c__Clostridia;o__Oscillospirales;f__Oscillospiraceae;g__Sporobacter;__ | 0 |
| d__Bacteria;p__Firmicutes;c__Bacilli;o__Bacillales;f__Bacillaceae;g__Bacillus;s__uncultured_bacterium | 0 |
| d__Bacteria;p__Firmicutes;c__Bacilli;o__Thermoactinomycetales;f__Thermoactinomycetaceae;g__Novibacillus;__ | 0 |
| d__Bacteria;p__Proteobacteria;c__Alphaproteobacteria;o__Rhizobiales;f__Devosiaceae;g__Devosia;s__metagenome | 0 |
| d__Bacteria;p__Firmicutes;c__Clostridia;o__Proteinivoracales;f__uncultured;g__uncultured;s__uncultured_bacterium | 0 |
| d__Bacteria;p__Firmicutes;c__Clostridia;o__Oscillospirales;f__Ruminococcaceae;g__Ruminococcus;__ | 0 |
| d__Bacteria;p__Bacteroidota;c__Bacteroidia;o__Chitinophagales;f__Chitinophagaceae;g__Flavitalea;s__uncultured_bacterium | 0 |
| d__Bacteria;p__Proteobacteria;c__Gammaproteobacteria;o__Coxiellales;f__Coxiellaceae;g__Coxiella;__ | 0 |
| d__Bacteria;p__Proteobacteria;c__Alphaproteobacteria;o__Rhizobiales;f__Rhizobiaceae;g__Allorhizobium-Neorhizobium-Pararhizobium-Rhizobium;__ | 0 |
| d__Bacteria;p__Firmicutes;c__Sulfobacillia;o__Sulfobacillales;f__Sulfobacillaceae;g__uncultured;s__uncultured_bacterium | 0 |
| d__Bacteria;p__Proteobacteria;c__Gammaproteobacteria;o__Burkholderiales;f__Burkholderiaceae;g__Cupriavidus;s__Cupriavidus_necator | 0 |
| d__Bacteria;p__Acidobacteriota;c__Vicinamibacteria;o__Vicinamibacterales;f__uncultured;g__uncultured;__ | 0 |
| d__Bacteria;p__Proteobacteria;c__Gammaproteobacteria;o__Piscirickettsiales;f__Piscirickettsiaceae;g__Candidatus_Endoecteinascidia;__ | 0 |
| d__Bacteria;p__Planctomycetota;c__Planctomycetes;o__Isosphaerales;f__Isosphaeraceae;g__uncultured;s__uncultured_bacterium | 0 |
| d__Bacteria;p__Proteobacteria;c__Alphaproteobacteria;o__Sphingomonadales;f__Sphingomonadaceae;g__Sphingobium;s__Sphingobium_yanoikuyae | 0 |
| d__Bacteria;p__Firmicutes;c__Bacilli;o__Bacillales;f__Bacillaceae;g__Bacillus;s__Bacillus_halodurans | 0 |
| d__Bacteria;p__Proteobacteria;c__Gammaproteobacteria;o__Burkholderiales;f__Burkholderiaceae;g__Burkholderia-Caballeronia-Paraburkholderia;s__Burkholderia_cepacia | 0 |
| d__Bacteria;p__Proteobacteria;c__Gammaproteobacteria;o__Pseudomonadales;f__Pseudohongiellaceae;g__BIyi10;__ | 0 |
| d__Bacteria;p__Proteobacteria;c__Alphaproteobacteria;o__Acetobacterales;f__Acetobacteraceae;g__uncultured;__ | 0 |
| d__Bacteria;p__Bacteroidota;c__Bacteroidia;o__Chitinophagales;f__Chitinophagaceae;__;__ | 0 |
| d__Bacteria;p__Proteobacteria;c__Gammaproteobacteria;o__Nitrosococcales;f__Nitrosococcaceae;g__AqS1;__ | 0 |
| d__Bacteria;p__Acidobacteriota;c__Acidobacteriae;o__Acidobacteriales;f__Acidobacteriaceae_(Subgroup_1);g__Edaphobacter;__ | 0 |
| d__Bacteria;p__Actinobacteriota;c__Actinobacteria;o__Propionibacteriales;f__Nocardioidaceae;g__Nocardioides;s__uncultured_bacterium | 0 |
| d__Bacteria;p__Bacteroidota;c__Bacteroidia;o__Chitinophagales;f__Chitinophagaceae;g__Niastella;s__uncultured_bacterium | 0 |
| d__Bacteria;p__Proteobacteria;c__Gammaproteobacteria;o__Burkholderiales;f__Alcaligenaceae;__;__ | 0 |
| d__Bacteria;p__Firmicutes;c__Clostridia;o__Oscillospirales;f__Oscillospiraceae;__;__ | 0 |
| d__Bacteria;p__Myxococcota;c__Myxococcia;o__Myxococcales;f__Myxococcaceae;g__Stigmatella;__ | 0 |
| d__Bacteria;p__Proteobacteria;c__Alphaproteobacteria;o__Rhizobiales;f__Rhizobiaceae;g__Aminobacter;__ | 0 |
| d__Bacteria;p__Proteobacteria;c__Alphaproteobacteria;o__Sphingomonadales;f__Sphingomonadaceae;g__Altererythrobacter;__ | 0 |
| d__Bacteria;p__Acidobacteriota;c__Acidobacteriae;o__Acidobacteriales;f__uncultured;g__uncultured;s__uncultured_bacterium | 0 |
| d__Bacteria;p__Proteobacteria;c__Alphaproteobacteria;o__Sphingomonadales;f__Sphingomonadaceae;g__uncultured;__ | 0 |
| d__Bacteria;p__Proteobacteria;c__Alphaproteobacteria;o__Micropepsales;f__Micropepsaceae;g__uncultured;__ | 0 |
| d__Bacteria;p__Proteobacteria;c__Alphaproteobacteria;o__Caulobacterales;f__Caulobacteraceae;g__Asticcacaulis;__ | 0 |
| d__Bacteria;p__Planctomycetota;c__Planctomycetes;o__Planctomycetales;f__Schlesneriaceae;g__Planctopirus;__ | 0 |
| d__Bacteria;p__Actinobacteriota;c__Acidimicrobiia;o__uncultured;f__uncultured;g__uncultured;s__uncultured_bacterium | 0 |
| d__Bacteria;p__Firmicutes;c__Clostridia;o__Clostridiales;f__Clostridiaceae;g__Clostridium_sensu_stricto_1;s__Clostridium_beijerinckii | 0 |
| d__Bacteria;p__Proteobacteria;c__Gammaproteobacteria;o__Pseudomonadales;f__Pseudomonadaceae;g__Pseudomonas;s__Pseudomonas_citronellolis | 0 |
| d__Bacteria;p__Firmicutes;c__Bacilli;o__Bacillales;f__Bacillaceae;g__Bacillus;s__Bacillus_coagulans | 0 |
| d__Bacteria;p__Firmicutes;c__Clostridia;o__Clostridiales;f__Clostridiaceae;g__Clostridium_sensu_stricto_7;__ | 0 |
| d__Bacteria;p__Proteobacteria;c__Gammaproteobacteria;__;__;__;__ | 0 |
| d__Bacteria;p__Bacteroidota;c__Bacteroidia;o__Sphingobacteriales;f__Sphingobacteriaceae;g__Pedobacter;__ | 0 |
| d__Bacteria;p__Firmicutes;c__Incertae_Sedis;o__DTU014;f__DTU014;g__DTU014;__ | 0 |
| d__Bacteria;p__Proteobacteria;c__Gammaproteobacteria;o__Gammaproteobacteria_Incertae_Sedis;f__Unknown_Family;g__Candidatus_Ovatusbacter;s__uncultured_bacterium | 0 |
| d__Bacteria;p__Firmicutes;c__Clostridia;o__Peptostreptococcales-Tissierellales;f__Peptostreptococcales-Tissierellales;g__Alkaliphilus;__ | 0 |
| d__Bacteria;p__Proteobacteria;c__Alphaproteobacteria;o__Rhizobiales;f__Rhizobiaceae;g__Allorhizobium-Neorhizobium-Pararhizobium-Rhizobium;s__uncultured_bacterium | 0 |
| d__Bacteria;p__Actinobacteriota;c__Actinobacteria;o__Micrococcales;f__Intrasporangiaceae;g__Pedococcus-Phycicoccus;__ | 0 |
| d__Bacteria;p__Proteobacteria;c__Gammaproteobacteria;o__Xanthomonadales;f__Rhodanobacteraceae;g__Chujaibacter;s__uncultured_bacterium | 0 |
| d__Bacteria;p__Planctomycetota;c__Planctomycetes;o__Isosphaerales;f__Isosphaeraceae;g__Aquisphaera;s__uncultured_bacterium | 0 |
| d__Bacteria;p__Actinobacteriota;c__Thermoleophilia;o__Solirubrobacterales;f__67-14;g__67-14;s__uncultured_bacterium | 0 |
| d__Bacteria;p__Chloroflexi;c__Ktedonobacteria;o__Ktedonobacterales;f__Ktedonobacteraceae;g__uncultured;__ | 0 |
| d__Bacteria;p__Proteobacteria;c__Gammaproteobacteria;o__Burkholderiales;f__Comamonadaceae;g__Hydrogenophaga;__ | 0 |
| d__Bacteria;p__Proteobacteria;c__Alphaproteobacteria;o__Rhizobiales;f__Rhizobiaceae;g__Ensifer;__ | 0 |
| d__Bacteria;p__Proteobacteria;c__Alphaproteobacteria;o__Caulobacterales;f__Caulobacteraceae;g__uncultured;__ | 0 |
| d__Bacteria;p__Proteobacteria;c__Alphaproteobacteria;o__Rhizobiales;f__Beijerinckiaceae;__;__ | 0 |
| d__Bacteria;p__Gemmatimonadota;c__Gemmatimonadetes;o__Gemmatimonadales;f__Gemmatimonadaceae;g__uncultured;s__uncultured_bacterium | 0 |
| d__Bacteria;p__Actinobacteriota;c__Actinobacteria;o__Micrococcales;f__Intrasporangiaceae;g__Knoellia;__ | 0 |
| d__Bacteria;p__Proteobacteria;c__Alphaproteobacteria;o__Sphingomonadales;f__Sphingomonadaceae;g__Novosphingobium;__ | 0 |
| d__Bacteria;p__Firmicutes;c__Dethiobacteria;o__Dethiobacterales;f__Dethiobacteraceae;g__Dethiobacter;s__uncultured_bacterium | 0 |
| d__Bacteria;p__Firmicutes;c__Bacilli;o__Bacillales;f__Bacillaceae;g__Ureibacillus;__ | 0 |
| d__Bacteria;p__Armatimonadota;c__uncultured;o__uncultured;f__uncultured;g__uncultured;s__uncultured_bacterium | 0 |
| d__Bacteria;p__Firmicutes;c__Symbiobacteriia;o__Symbiobacteriales;f__Symbiobacteraceae;g__uncultured;s__uncultured_bacterium | 0 |
| d__Bacteria;p__Proteobacteria;c__Gammaproteobacteria;o__Xanthomonadales;f__Rhodanobacteraceae;g__Rhodanobacter;s__uncultured_bacterium | 0 |
| d__Bacteria;p__Acidobacteriota;c__Acidobacteriae;o__Acidobacteriales;f__Acidobacteriaceae_(Subgroup_1);__;__ | 0 |
| d__Bacteria;p__Firmicutes;c__Bacilli;o__Paenibacillales;f__Paenibacillaceae;g__Thermobacillus;__ | 0 |
| d__Bacteria;p__Gemmatimonadota;c__Gemmatimonadetes;o__Gemmatimonadales;f__Gemmatimonadaceae;g__Gemmatimonas;__ | 0 |
| d__Bacteria;p__Firmicutes;c__Limnochordia;o__Limnochordia;f__Limnochordia;g__Hydrogenispora;s__uncultured_bacterium | 0 |
| d__Bacteria;p__Actinobacteriota;c__Actinobacteria;o__Propionibacteriales;f__Nocardioidaceae;g__Marmoricola;__ | 0 |
| d__Bacteria;p__Firmicutes;c__Bacilli;o__Bacillales;f__Planococcaceae;g__Lysinibacillus;__ | 0 |
| d__Bacteria;p__Bacteroidota;c__Bacteroidia;o__Chitinophagales;f__Chitinophagaceae;g__Ferruginibacter;__ | 0 |
| d__Bacteria;p__Bacteroidota;c__Bacteroidia;o__Flavobacteriales;f__Crocinitomicaceae;g__Fluviicola;__ | 0 |
| d__Bacteria;p__Firmicutes;c__Clostridia;o__Oscillospirales;f__Hungateiclostridiaceae;g__Ruminiclostridium;s__uncultured_bacterium | 0 |
| d__Bacteria;p__Actinobacteriota;c__Actinobacteria;o__Frankiales;f__Acidothermaceae;g__Acidothermus;__ | 0 |
| d__Bacteria;p__Chloroflexi;c__Ktedonobacteria;o__Ktedonobacterales;f__Ktedonobacteraceae;g__uncultured;s__uncultured_bacterium | 0 |
| d__Bacteria;p__Actinobacteriota;c__Actinobacteria;o__Frankiales;f__Acidothermaceae;g__Acidothermus;s__uncultured_bacterium | 0 |
| d__Bacteria;p__Proteobacteria;c__Gammaproteobacteria;o__Gammaproteobacteria_Incertae_Sedis;f__Unknown_Family;g__Acidibacter;s__uncultured_bacterium | 0 |
| d__Bacteria;p__Proteobacteria;c__Alphaproteobacteria;o__Rhizobiales;f__Xanthobacteraceae;g__Afipia;__ | 0 |
| d__Bacteria;p__Firmicutes;c__Clostridia;o__Clostridiales;f__Clostridiaceae;g__Clostridium_sensu_stricto_1;s__Clostridium_baratii | 0 |
| d__Bacteria;p__Proteobacteria;c__Gammaproteobacteria;o__Burkholderiales;f__Comamonadaceae;g__Methylibium;__ | 0 |
| d__Bacteria;p__Proteobacteria;c__Gammaproteobacteria;o__B2M28;f__B2M28;g__B2M28;__ | 0 |
| d__Bacteria;p__Firmicutes;c__Bacilli;o__Exiguobacterales;f__Exiguobacteraceae;g__Exiguobacterium;__ | 0 |
| d__Bacteria;p__Actinobacteriota;c__Actinobacteria;o__Corynebacteriales;f__Mycobacteriaceae;g__Mycobacterium;__ | 0 |
| d__Bacteria;p__Bacteroidota;c__Bacteroidia;o__Cytophagales;f__Hymenobacteraceae;g__Pontibacter;__ | 0 |
| d__Bacteria;p__Proteobacteria;c__Gammaproteobacteria;o__Burkholderiales;f__Methylophilaceae;__;__ | 0 |
| d__Bacteria;p__Firmicutes;c__Clostridia;o__Peptostreptococcales-Tissierellales;f__Family_XI;g__Tissierella;__ | 0 |
| d__Bacteria;p__Proteobacteria;c__Alphaproteobacteria;o__Rhizobiales;f__Pleomorphomonadaceae;g__Chthonobacter;__ | 0 |
| d__Bacteria;p__Planctomycetota;c__Phycisphaerae;o__Tepidisphaerales;f__WD2101_soil_group;g__WD2101_soil_group;s__uncultured_bacterium | 0 |
| d__Bacteria;p__Proteobacteria;c__Gammaproteobacteria;o__Pseudomonadales;f__Moraxellaceae;g__uncultured;s__metagenome | 0 |
| d__Bacteria;p__Proteobacteria;c__Gammaproteobacteria;o__Nitrosococcales;f__Nitrosococcaceae;__;__ | 0 |
| d__Bacteria;p__Bacteroidota;c__Bacteroidia;o__Chitinophagales;f__Chitinophagaceae;g__uncultured;__ | 0 |
| d__Bacteria;p__Proteobacteria;c__Alphaproteobacteria;o__Micropepsales;f__Micropepsaceae;g__uncultured;s__metagenome | 0 |
| d__Bacteria;p__Proteobacteria;c__Alphaproteobacteria;o__Rhizobiales;f__Methyloligellaceae;g__Methyloceanibacter;__ | 0 |
| d__Bacteria;p__Firmicutes;c__Bacilli;o__Bacillales;f__Planococcaceae;g__Sporosarcina;__ | 0 |
| d__Bacteria;p__Firmicutes;c__Bacilli;o__Alicyclobacillales;f__Alicyclobacillaceae;g__Alicyclobacillus;__ | 0 |
| d__Bacteria;p__Bdellovibrionota;c__Oligoflexia;o__Oligoflexales;f__Oligoflexaceae;g__uncultured;__ | 0 |
| d__Bacteria;p__Firmicutes;c__Bacilli;o__Paenibacillales;f__Paenibacillaceae;g__Paenibacillus;s__Paenibacillus_sp. | 0 |
| d__Bacteria;p__Firmicutes;c__Symbiobacteriia;o__Symbiobacteriales;f__Symbiobacteraceae;g__Symbiobacterium;__ | 0 |
| d__Bacteria;p__Firmicutes;c__Bacilli;o__Bacillales;f__Bacillaceae;g__Ureibacillus;s__Ureibacillus_thermosphaericus | 0 |
| d__Bacteria;p__Patescibacteria;c__Saccharimonadia;o__Saccharimonadales;f__Saccharimonadales;g__Saccharimonadales;s__uncultured_bacterium | 0 |
| d__Bacteria;p__Myxococcota;c__Polyangia;o__Haliangiales;f__Haliangiaceae;g__Haliangium;s__uncultured_bacterium | 0 |
| d__Bacteria;p__Proteobacteria;c__Alphaproteobacteria;o__Paracaedibacterales;f__Paracaedibacteraceae;g__uncultured;s__uncultured_bacterium | 0 |
| d__Bacteria;p__Bacteroidota;c__Bacteroidia;o__Chitinophagales;f__Chitinophagaceae;g__Flavisolibacter;s__uncultured_bacterium | 0 |
| d__Bacteria;p__Proteobacteria;c__Alphaproteobacteria;o__Rhodobacterales;f__Rhodobacteraceae;g__Rhodobacter;__ | 0 |
| d__Bacteria;p__Proteobacteria;c__Alphaproteobacteria;o__Rhizobiales;f__Rhizobiaceae;g__Ensifer;s__Ensifer_adhaerens | 0 |
| d__Bacteria;p__Proteobacteria;c__Alphaproteobacteria;o__Dongiales;f__Dongiaceae;g__Dongia;__ | 0 |
| d__Bacteria;p__Patescibacteria;c__Saccharimonadia;o__Saccharimonadales;__;__;__ | 0 |
| d__Bacteria;p__Actinobacteriota;c__Actinobacteria;o__Micrococcales;f__Microbacteriaceae;__;__ | 0 |
| d__Bacteria;p__Actinobacteriota;c__Actinobacteria;o__Micrococcales;f__Micrococcaceae;g__Arthrobacter;__ | 0 |
| d__Bacteria;p__Acidobacteriota;c__Acidobacteriae;o__Acidobacteriales;f__Acidobacteriaceae_(Subgroup_1);g__uncultured;s__uncultured_bacterium | 0 |
| d__Bacteria;p__Actinobacteriota;c__Actinobacteria;o__Propionibacteriales;f__Propionibacteriaceae;g__Microlunatus;__ | 0 |
| d__Bacteria;p__Firmicutes;c__Bacilli;o__Bacillales;f__Bacillaceae;g__Oceanobacillus;__ | 0 |
| d__Bacteria;p__Proteobacteria;c__Gammaproteobacteria;o__Legionellales;f__Legionellaceae;g__Legionella;s__Legionella_longbeachae | 0 |
| d__Bacteria;p__Firmicutes;c__Bacilli;o__Bacillales;f__Bacillaceae;g__Bacillus;s__Bacillus_lentus | 0 |
| d__Bacteria;p__Bacteroidota;c__Bacteroidia;o__Flavobacteriales;f__Flavobacteriaceae;g__Flavobacterium;__ | 0 |
| d__Bacteria;p__Bacteroidota;c__Bacteroidia;o__Flavobacteriales;f__Weeksellaceae;g__Chryseobacterium;__ | 0 |
| d__Bacteria;p__Proteobacteria;c__Alphaproteobacteria;o__Rhizobiales;f__Beijerinckiaceae;g__Microvirga;__ | 0 |
| d__Bacteria;p__Firmicutes;c__Bacilli;o__Bacillales;f__Bacillaceae;__;__ | 0 |
| d__Bacteria;p__Firmicutes;c__Desulfotomaculia;o__Desulfotomaculales;f__Desulfotomaculaceae;g__Desulfofarcimen;__ | 0 |
| d__Bacteria;p__Actinobacteriota;c__Actinobacteria;o__Pseudonocardiales;f__Pseudonocardiaceae;g__Pseudonocardia;__ | 0 |
| d__Bacteria;p__Firmicutes;c__Thermacetogenia;o__Thermacetogeniales;f__Thermacetogeniaceae;g__Syntrophaceticus;s__uncultured_bacterium | 0 |
| d__Bacteria;p__Myxococcota;c__Myxococcia;o__Myxococcales;f__Vulgatibacteraceae;g__Vulgatibacter;__ | 0 |
| d__Bacteria;p__Firmicutes;c__Clostridia;o__Oscillospirales;f__Hungateiclostridiaceae;g__HN-HF0106;s__uncultured_bacterium | 0 |
| d__Bacteria;p__Firmicutes;c__Clostridia;o__Clostridiales;f__Clostridiaceae;g__Clostridium_sensu_stricto_1;s__Clostridium_saccharobutylicum | 0 |
| d__Bacteria;p__Acidobacteriota;c__Acidobacteriae;o__Acidobacteriales;f__Koribacteraceae;g__Candidatus_Koribacter;__ | 0 |
| d__Bacteria;p__Patescibacteria;c__Saccharimonadia;o__Saccharimonadales;f__LWQ8;g__LWQ8;s__uncultured_bacterium | 0 |
| d__Bacteria;p__Bacteroidota;c__Bacteroidia;o__Chitinophagales;f__Chitinophagaceae;g__uncultured;s__uncultured_bacterium | 0 |
| d__Bacteria;p__Bacteroidota;c__Bacteroidia;o__Sphingobacteriales;f__Sphingobacteriaceae;g__uncultured;__ | 0 |
| d__Bacteria;p__Verrucomicrobiota;c__Verrucomicrobiae;o__Pedosphaerales;f__Pedosphaeraceae;g__Ellin516;__ | 0 |
| d__Bacteria;p__Firmicutes;c__Limnochordia;o__Limnochordales;f__Limnochordaceae;g__Limnochordaceae;s__uncultured_bacterium | 0 |
| d__Bacteria;p__Firmicutes;c__Clostridia;o__Oscillospirales;f__Hungateiclostridiaceae;__;__ | 0 |
| d__Bacteria;p__Acidobacteriota;c__Acidobacteriae;o__Acidobacteriales;f__Acidobacteriaceae_(Subgroup_1);g__Granulicella;__ | 0 |
| d__Bacteria;p__Firmicutes;c__Limnochordia;o__M55-D21;f__M55-D21;g__M55-D21;__ | 0 |
| d__Bacteria;p__Proteobacteria;c__Gammaproteobacteria;o__Burkholderiales;f__Methylophilaceae;g__MM2;__ | 0 |
| d__Bacteria;p__Firmicutes;c__Syntrophomonadia;o__Syntrophomonadales;f__Syntrophomonadaceae;g__uncultured;__ | 0 |
| d__Bacteria;p__Proteobacteria;c__Alphaproteobacteria;o__Rhizobiales;f__Xanthobacteraceae;g__uncultured;__ | 0 |
| d__Bacteria;p__Planctomycetota;c__Planctomycetes;o__Pirellulales;f__Pirellulaceae;g__uncultured;__ | 0 |
| d__Bacteria;p__Actinobacteriota;c__Actinobacteria;o__Catenulisporales;f__Actinospicaceae;g__Actinospica;__ | 0 |
| d__Bacteria;p__Proteobacteria;c__Alphaproteobacteria;o__Caulobacterales;f__Caulobacteraceae;g__uncultured;s__metagenome | 0 |
| d__Bacteria;p__Bdellovibrionota;c__Bdellovibrionia;o__Bdellovibrionales;f__Bdellovibrionaceae;g__Bdellovibrio;s__uncultured_bacterium | 0 |
| d__Bacteria;p__Firmicutes;c__Desulfotomaculia;o__Desulfotomaculales;f__Desulfotomaculaceae;g__Desulfotomaculum;s__Desulfotomaculum_ferrireducens | 0 |
| d__Bacteria;p__Bacteroidota;c__Bacteroidia;o__Sphingobacteriales;f__Sphingobacteriaceae;__;__ | 0 |
| d__Bacteria;p__Bacteroidota;c__Bacteroidia;o__Chitinophagales;f__Chitinophagaceae;g__Flavisolibacter;__ | 0 |
| d__Bacteria;p__Actinobacteriota;c__Actinobacteria;o__Micrococcales;f__Microbacteriaceae;g__Gryllotalpicola;__ | 0 |
| d__Bacteria;p__Bdellovibrionota;c__Oligoflexia;o__0319-6G20;f__0319-6G20;g__0319-6G20;s__uncultured_bacterium | 0 |
| d__Bacteria;p__Firmicutes;c__Bacilli;o__Bacillales;f__Bacillaceae;g__Bacillus;s__Bacillus_methanolicus | 0 |
| d__Bacteria;p__Armatimonadota;c__Armatimonadia;o__Armatimonadales;f__Armatimonadales;g__Armatimonadales;s__uncultured_bacterium | 0 |
| d__Bacteria;p__Firmicutes;c__Bacilli;o__Bacillales;f__Planococcaceae;g__Chryseomicrobium;s__uncultured_bacterium | 0 |
| d__Bacteria;p__Proteobacteria;c__Gammaproteobacteria;o__Burkholderiales;f__Comamonadaceae;g__Azohydromonas;__ | 0 |
| d__Bacteria;p__Proteobacteria;c__Gammaproteobacteria;o__Burkholderiales;f__Rhodocyclaceae;g__Azoarcus;__ | 0 |
| d__Bacteria;p__Bacteroidota;c__Bacteroidia;o__Chitinophagales;f__Chitinophagaceae;g__Terrimonas;__ | 0 |
| d__Bacteria;p__Firmicutes;c__Bacilli;o__Paenibacillales;f__Paenibacillaceae;g__Paenibacillus;s__Paenibacillus_beijingensis | 0 |
| d__Bacteria;p__Proteobacteria;c__Gammaproteobacteria;o__Legionellales;f__Legionellaceae;g__Legionella;__ | 0 |
| d__Bacteria;p__Proteobacteria;c__Gammaproteobacteria;o__Diplorickettsiales;f__Diplorickettsiaceae;g__Aquicella;s__uncultured_bacterium | 0 |
| d__Bacteria;p__Firmicutes;c__Limnochordia;o__MBA03;f__MBA03;g__MBA03;s__uncultured_bacterium | 0 |
| d__Bacteria;p__Proteobacteria;c__Gammaproteobacteria;o__Burkholderiales;f__Alcaligenaceae;g__Parapusillimonas;__ | 0 |
| d__Bacteria;p__Proteobacteria;c__Gammaproteobacteria;o__Xanthomonadales;f__Rhodanobacteraceae;g__Luteibacter;__ | 0 |
| d__Bacteria;p__Firmicutes;c__Clostridia;o__Lachnospirales;f__Lachnospiraceae;g__Cellulosilyticum;s__uncultured_bacterium | 0 |
| d__Bacteria;p__Planctomycetota;c__Planctomycetes;o__Pirellulales;f__Pirellulaceae;g__uncultured;s__uncultured_bacterium | 0 |
| d__Bacteria;p__Firmicutes;c__Clostridia;o__Oscillospirales;f__Hungateiclostridiaceae;g__Ruminiclostridium;__ | 0 |
| d__Bacteria;p__Planctomycetota;c__Planctomycetes;o__Planctomycetales;f__Rubinisphaeraceae;g__Planctomicrobium;s__uncultured_bacterium | 0 |
| d__Bacteria;p__Firmicutes;c__Clostridia;o__Peptostreptococcales-Tissierellales;f__Peptostreptococcaceae;g__Paraclostridium;s__Paraclostridium_bifermentans | 0 |
| d__Bacteria;p__Firmicutes;c__Bacilli;o__Erysipelotrichales;f__Erysipelotrichaceae;g__Turicibacter;__ | 0 |
| d__Bacteria;p__Actinobacteriota;c__Actinobacteria;o__Frankiales;f__Frankiaceae;g__Jatrophihabitans;__ | 0 |
| d__Bacteria;p__Firmicutes;c__Clostridia;o__Lachnospirales;f__Lachnospiraceae;g__uncultured;__ | 0 |
| d__Bacteria;p__Actinobacteriota;c__Actinobacteria;o__Frankiales;__;__;__ | 0 |
| d__Bacteria;p__Proteobacteria;c__Alphaproteobacteria;o__Rhizobiales;f__Rhizobiales_Incertae_Sedis;g__Bauldia;s__uncultured_bacterium | 0 |
| d__Bacteria;p__Firmicutes;c__Clostridia;o__Clostridia_UCG-014;f__Clostridia_UCG-014;g__Clostridia_UCG-014;s__uncultured_bacterium | 0 |
| d__Bacteria;p__Actinobacteriota;c__Acidimicrobiia;o__IMCC26256;f__IMCC26256;g__IMCC26256;s__uncultured_bacterium | 0 |
| d__Bacteria;p__Firmicutes;c__Clostridia;o__Oscillospirales;f__Ruminococcaceae;g__Ruminococcus;s__uncultured_rumen | 0 |
| d__Bacteria;p__Bacteroidota;c__Bacteroidia;o__Cytophagales;f__Spirosomaceae;g__Dyadobacter;__ | 0 |
| d__Bacteria;p__Proteobacteria;c__Alphaproteobacteria;o__Rhizobiales;f__Devosiaceae;__;__ | 0 |
| d__Bacteria;p__Firmicutes;c__Bacilli;o__Paenibacillales;f__Paenibacillaceae;g__Paenibacillus;s__Paenibacillus_swuensis | 0 |
| d__Bacteria;p__Proteobacteria;c__Gammaproteobacteria;o__Xanthomonadales;f__Rhodanobacteraceae;g__Mizugakiibacter;__ | 0 |
| d__Bacteria;p__Abditibacteriota;c__Abditibacteria;o__Abditibacteriales;f__Abditibacteriaceae;g__Abditibacterium;s__uncultured_bacterium | 0 |
| d__Bacteria;p__Firmicutes;c__Clostridia;o__Lachnospirales;f__Lachnospiraceae;g__Lachnospiraceae_NK4A136_group;__ | 0 |
| d__Bacteria;p__Proteobacteria;c__Gammaproteobacteria;o__Pseudomonadales;f__Pseudomonadaceae;g__Pseudomonas;s__uncultured_bacterium | 0 |
| d__Bacteria;p__Firmicutes;c__Bacilli;o__Bacillales;f__Bacillaceae;g__Bacillus;s__Bacillus_sp. | 0 |
| d__Bacteria;p__Proteobacteria;c__Alphaproteobacteria;o__Rhizobiales;f__Methyloligellaceae;__;__ | 0 |
| d__Bacteria;p__Firmicutes;c__Thermacetogenia;o__Thermacetogeniales;f__Thermacetogeniaceae;__;__ | 0 |
| d__Bacteria;p__Planctomycetota;c__Planctomycetes;o__Gemmatales;f__Gemmataceae;g__uncultured;s__uncultured_bacterium | 0 |
| d__Bacteria;p__Firmicutes;c__Clostridia;o__Caldicoprobacterales;f__Caldicoprobacteraceae;g__Caldicoprobacter;s__uncultured_bacterium | 0 |
| d__Bacteria;p__Firmicutes;c__Clostridia;o__Clostridiales;f__Clostridiaceae;g__Clostridium_sensu_stricto_6;s__Clostridium_cellulovorans | 0 |
| d__Bacteria;p__Acidobacteriota;c__Acidobacteriae;o__Acidobacteriales;f__Acidobacteriaceae_(Subgroup_1);g__uncultured;s__uncultured_forest | 0 |
| d__Bacteria;p__Firmicutes;c__Bacilli;o__Paenibacillales;f__Paenibacillaceae;__;__ | 0 |
| d__Bacteria;p__Dependentiae;c__Babeliae;o__Babeliales;f__Vermiphilaceae;g__Vermiphilaceae;s__uncultured_bacterium | 0 |
| d__Bacteria;p__Proteobacteria;c__Alphaproteobacteria;o__Rhizobiales;f__Xanthobacteraceae;g__Rhodoplanes;__ | 0 |
| d__Bacteria;p__Firmicutes;c__Bacilli;o__Bacillales;f__Planococcaceae;g__Solibacillus;s__uncultured_bacterium | 0 |
| d__Bacteria;p__Firmicutes;c__Bacilli;o__Paenibacillales;f__Paenibacillaceae;g__Cohnella;s__Cohnella_sp. | 0 |
| d__Bacteria;p__Firmicutes;c__Clostridia;o__Peptostreptococcales-Tissierellales;f__Peptostreptococcaceae;g__Intestinibacter;__ | 0 |
| d__Bacteria;p__Firmicutes;c__Clostridia;o__Thermincolales;f__Thermincolaceae;g__Thermincola;__ | 0 |
| d__Bacteria;p__Firmicutes;c__Bacilli;o__Bacillales;f__Sporolactobacillaceae;__;__ | 0 |
| d__Bacteria;p__Verrucomicrobiota;c__Verrucomicrobiae;o__Pedosphaerales;f__Pedosphaeraceae;__;__ | 0 |
| d__Bacteria;p__Firmicutes;c__Bacilli;o__Alicyclobacillales;f__Alicyclobacillaceae;g__Alicyclobacillus;s__Alicyclobacillus_acidoterrestris | 0 |
| d__Bacteria;p__Firmicutes;c__Clostridia;o__Lachnospirales;f__Lachnospiraceae;g__Herbinix;s__uncultured_bacterium | 0 |
| d__Bacteria;p__Proteobacteria;c__Gammaproteobacteria;o__Burkholderiales;f__Alcaligenaceae;g__uncultured;s__uncultured_bacterium | 0 |
| d__Bacteria;p__Verrucomicrobiota;c__Verrucomicrobiae;o__Pedosphaerales;f__Pedosphaeraceae;g__uncultured;__ | 0 |
| d__Bacteria;p__Proteobacteria;c__Alphaproteobacteria;o__Caulobacterales;f__Caulobacteraceae;g__Phenylobacterium;__ | 0 |
| d__Bacteria;p__Proteobacteria;c__Gammaproteobacteria;o__Burkholderiales;f__Burkholderiaceae;g__Burkholderia-Caballeronia-Paraburkholderia;s__Burkholderia_multivorans | 0 |
| d__Bacteria;p__Firmicutes;c__Clostridia;o__Oscillospirales;f__UCG-010;g__UCG-010;s__uncultured_bacterium | 0 |
| d__Bacteria;p__Proteobacteria;c__Gammaproteobacteria;o__Burkholderiales;f__Comamonadaceae;g__Ramlibacter;s__uncultured_bacterium | 0 |
| d__Bacteria;p__Acidobacteriota;c__Acidobacteriae;o__Acidobacteriales;f__Acidobacteriaceae_(Subgroup_1);g__Acidipila-Silvibacterium;s__uncultured_Acidobacteria | 0 |
| d__Bacteria;p__Firmicutes;c__Clostridia;o__Peptococcales;f__Peptococcaceae;g__Dehalobacterium;__ | 0 |
| d__Bacteria;p__Bacteroidota;c__Bacteroidia;o__Sphingobacteriales;f__Sphingobacteriaceae;g__Mucilaginibacter;__ | 0 |
| d__Bacteria;p__Firmicutes;c__Bacilli;o__Bacillales;f__Planococcaceae;g__Chryseomicrobium;__ | 0 |
| d__Bacteria;p__Firmicutes;c__Bacilli;o__Bacillales;f__Bacillaceae;g__Fictibacillus;__ | 0 |
| d__Bacteria;p__Actinobacteriota;c__Actinobacteria;o__Micrococcales;f__Micrococcaceae;g__Pseudarthrobacter;s__Arthrobacter_sp. | 0 |
| d__Bacteria;p__Proteobacteria;c__Gammaproteobacteria;o__KF-JG30-C25;f__KF-JG30-C25;g__KF-JG30-C25;s__uncultured_bacterium | 0 |
| d__Bacteria;p__Actinobacteriota;c__Thermoleophilia;o__Solirubrobacterales;f__Solirubrobacteraceae;g__Conexibacter;__ | 0 |
| d__Bacteria;p__Proteobacteria;c__Alphaproteobacteria;o__Rhizobiales;f__Hyphomicrobiaceae;g__Hyphomicrobium;s__Hyphomicrobium_sp. | 0 |
| d__Bacteria;p__Actinobacteriota;c__Acidimicrobiia;o__uncultured;f__uncultured;g__uncultured;__ | 0 |
| d__Bacteria;p__Bdellovibrionota;c__Bdellovibrionia;o__Bdellovibrionales;f__Bdellovibrionaceae;g__Bdellovibrio;s__Bdellovibrio_bacteriovorus | 0 |
| d__Bacteria;p__Proteobacteria;c__Alphaproteobacteria;o__Caulobacterales;f__Caulobacteraceae;g__Caulobacter;__ | 0 |
| d__Bacteria;p__Myxococcota;c__Myxococcia;o__Myxococcales;f__Myxococcaceae;__;__ | 0 |
| d__Bacteria;p__Firmicutes;c__Clostridia;o__Lachnospirales;f__Lachnospiraceae;g__Lachnospiraceae_UCG-001;s__uncultured_bacterium | 0 |
| d__Bacteria;p__Myxococcota;c__Myxococcia;o__Myxococcales;f__Myxococcaceae;g__P3OB-42;__ | 0 |
| d__Bacteria;p__Chloroflexi;c__Ktedonobacteria;o__Ktedonobacterales;f__JG30-KF-AS9;g__JG30-KF-AS9;s__uncultured_bacterium | 0 |
| d__Bacteria;p__Proteobacteria;c__Alphaproteobacteria;o__Elsterales;f__uncultured;g__uncultured;s__uncultured_Alphaproteobacteria | 0 |
| d__Bacteria;p__Proteobacteria;c__Gammaproteobacteria;o__Diplorickettsiales;f__Diplorickettsiaceae;g__uncultured;__ | 0 |
| d__Bacteria;p__Proteobacteria;c__Gammaproteobacteria;o__Burkholderiales;f__Comamonadaceae;__;__ | 0 |
| d__Bacteria;p__Proteobacteria;c__Alphaproteobacteria;o__Rhodospirillales;f__uncultured;g__uncultured;s__uncultured_bacterium | 0 |
| d__Bacteria;p__Dependentiae;c__Babeliae;o__Babeliales;f__Vermiphilaceae;g__Vermiphilaceae;__ | 0 |
| d__Bacteria;p__Firmicutes;c__Clostridia;o__Oscillospirales;f__Hungateiclostridiaceae;g__HN-HF0106;__ | 0 |
| d__Bacteria;p__Firmicutes;c__Bacilli;o__Alicyclobacillales;f__Alicyclobacillaceae;g__Tumebacillus;s__Tumebacillus_algifaecis | 0 |
| d__Bacteria;p__Firmicutes;c__Bacilli;o__Bacillales;f__Bacillaceae;g__Pseudogracilibacillus;__ | 0 |
| d__Bacteria;p__Firmicutes;c__Incertae_Sedis;o__DTU014;f__DTU014;g__DTU014;s__uncultured_bacterium | 0 |
| d__Bacteria;p__Proteobacteria;c__Gammaproteobacteria;o__Xanthomonadales;f__Rhodanobacteraceae;__;__ | 0 |
| d__Bacteria;p__Firmicutes;c__Bacilli;o__Bacillales;f__Planococcaceae;g__Sporosarcina;s__uncultured_bacterium | 0 |
| d__Bacteria;p__Firmicutes;c__Moorellia;o__Desulfitibacterales;f__Desulfitibacteraceae;g__Desulfitibacter;__ | 0 |
| d__Bacteria;p__Proteobacteria;c__Gammaproteobacteria;o__Burkholderiales;f__Oxalobacteraceae;__;__ | 0 |
| d__Bacteria;p__Firmicutes;c__Bacilli;o__Exiguobacterales;f__Exiguobacteraceae;g__Exiguobacterium;s__Exiguobacterium_sp. | 0 |
| d__Bacteria;p__Firmicutes;c__Clostridia;o__Peptococcales;f__Peptococcaceae;__;__ | 0 |
| d__Bacteria;p__Myxococcota;c__Polyangia;o__Polyangiales;f__Phaselicystidaceae;g__Phaselicystis;s__uncultured_bacterium | 0 |
| d__Bacteria;p__Firmicutes;c__Bacilli;o__Bacillales;f__Bacillaceae;g__Bacillus;s__Bacillus_thuringiensis | 0 |
| d__Bacteria;p__Proteobacteria;c__Alphaproteobacteria;o__Rhizobiales;f__D05-2;g__D05-2;__ | 0 |
| d__Bacteria;p__Proteobacteria;c__Alphaproteobacteria;o__Caulobacterales;f__Caulobacteraceae;__;__ | 0 |
| d__Bacteria;p__Planctomycetota;c__Planctomycetes;o__Planctomycetales;f__uncultured;g__uncultured;s__uncultured_bacterium | 0 |
| d__Bacteria;p__Firmicutes;c__Clostridia;o__Clostridia;f__Gracilibacteraceae;g__Lutispora;s__uncultured_bacterium | 0 |
| d__Bacteria;p__Proteobacteria;c__Gammaproteobacteria;o__Burkholderiales;f__Comamonadaceae;g__Caenimonas;__ | 0 |
| d__Bacteria;p__Firmicutes;c__Clostridia;o__Peptostreptococcales-Tissierellales;f__Family_XI;g__Tepidimicrobium;__ | 0 |
| d__Bacteria;p__Bdellovibrionota;c__Bdellovibrionia;o__Bdellovibrionales;f__Bdellovibrionaceae;g__OM27_clade;s__uncultured_bacterium | 0 |
| d__Bacteria;p__Chloroflexi;c__Ktedonobacteria;o__C0119;f__C0119;g__C0119;s__uncultured_bacterium | 0 |
| d__Bacteria;p__Firmicutes;c__Bacilli;o__Bacillales;f__Bacillaceae;g__Bacillus;s__Bacillus_paralicheniformis | 0 |
| d__Bacteria;p__Firmicutes;c__Clostridia;o__Peptostreptococcales-Tissierellales;f__Peptostreptococcaceae;g__Terrisporobacter;__ | 0 |
| d__Bacteria;p__Cyanobacteria;c__Vampirivibrionia;o__Vampirovibrionales;f__Vampirovibrionaceae;__;__ | 0 |
| d__Bacteria;p__Bacteroidota;c__Bacteroidia;o__Chitinophagales;f__Chitinophagaceae;g__Parafilimonas;__ | 0 |
| d__Bacteria;p__Firmicutes;c__Bacilli;o__Bacillales;f__Sporolactobacillaceae;g__Pullulanibacillus;__ | 0 |
| d__Bacteria;p__Firmicutes;c__Clostridia;o__Lachnospirales;f__Lachnospiraceae;g__Lachnoclostridium;__ | 0 |
| d__Bacteria;p__Proteobacteria;c__Alphaproteobacteria;o__Rhodobacterales;f__Rhodobacteraceae;g__Rhodobacter;s__uncultured_bacterium | 0 |
| d__Bacteria;p__Actinobacteriota;c__Acidimicrobiia;o__Microtrichales;f__Iamiaceae;g__Iamia;__ | 0 |
| d__Bacteria;p__Proteobacteria;c__Gammaproteobacteria;o__Xanthomonadales;f__Xanthomonadaceae;g__Arenimonas;s__uncultured_bacterium | 0 |
| d__Bacteria;p__Gemmatimonadota;c__Longimicrobia;o__Longimicrobiales;f__Longimicrobiaceae;g__Longimicrobiaceae;s__uncultured_bacterium | 0 |
| d__Bacteria;p__Acidobacteriota;c__Blastocatellia;o__Blastocatellales;f__Blastocatellaceae;g__Blastocatellaceae;s__uncultured_bacterium | 0 |
| d__Bacteria;p__Gemmatimonadota;c__S0134_terrestrial_group;o__S0134_terrestrial_group;f__S0134_terrestrial_group;g__S0134_terrestrial_group;s__uncultured_bacterium | 0 |
| d__Bacteria;p__Firmicutes;c__Bacilli;o__Paenibacillales;f__Paenibacillaceae;g__Paenibacillus;s__Paenibacillaceae_bacterium | 0 |
| d__Bacteria;p__Acidobacteriota;c__Vicinamibacteria;o__Vicinamibacterales;f__Vicinamibacteraceae;g__Vicinamibacteraceae;__ | 0 |
| d__Bacteria;p__Proteobacteria;c__Gammaproteobacteria;o__Xanthomonadales;f__Xanthomonadaceae;g__Lysobacter;s__uncultured_bacterium | 0 |
| d__Bacteria;p__Proteobacteria;c__Gammaproteobacteria;o__Xanthomonadales;f__Rhodanobacteraceae;g__Dokdonella;__ | 0 |
| d__Bacteria;p__Firmicutes;c__Clostridia;o__Lachnospirales;f__Lachnospiraceae;g__Cellulosilyticum;__ | 0 |
| d__Bacteria;p__Acidobacteriota;c__Acidobacteriae;o__Acidobacteriales;__;__;__ | 0 |
| d__Bacteria;p__Proteobacteria;c__Gammaproteobacteria;o__Burkholderiales;f__Alcaligenaceae;g__Bordetella;__ | 0 |
| d__Bacteria;p__Firmicutes;c__Clostridia;o__Lachnospirales;f__Lachnospiraceae;g__uncultured;s__uncultured_bacterium | 0 |
| d__Bacteria;p__Firmicutes;c__Bacilli;o__Paenibacillales;f__Paenibacillaceae;g__Paenibacillus;s__uncultured_bacterium | 0 |
| d__Bacteria;p__Proteobacteria;c__Alphaproteobacteria;o__Sphingomonadales;f__Sphingomonadaceae;g__uncultured;s__uncultured_bacterium | 0 |
| d__Bacteria;p__Bdellovibrionota;c__Oligoflexia;o__0319-6G20;f__0319-6G20;g__0319-6G20;__ | 0 |
| d__Bacteria;p__Acidobacteriota;c__Acidobacteriae;o__Acidobacteriales;f__Acidobacteriaceae_(Subgroup_1);g__Acidobacterium;__ | 0 |
| d__Bacteria;p__Firmicutes;c__Limnochordia;o__MBA03;f__MBA03;g__MBA03;__ | 0 |
| d__Bacteria;p__Firmicutes;c__Bacilli;o__Bacillales;f__Bacillaceae;g__Cerasibacillus;__ | 0 |
| d__Bacteria;p__Proteobacteria;c__Alphaproteobacteria;o__Tistrellales;f__Geminicoccaceae;g__Candidatus_Alysiosphaera;__ | 0 |
| d__Bacteria;p__Proteobacteria;c__Gammaproteobacteria;o__Xanthomonadales;f__Xanthomonadaceae;__;__ | 0 |
| d__Bacteria;p__Proteobacteria;c__Gammaproteobacteria;o__Burkholderiales;f__Oxalobacteraceae;g__uncultured;__ | 0 |
| d__Bacteria;p__Proteobacteria;c__Gammaproteobacteria;o__EPR3968-O8a-Bc78;f__EPR3968-O8a-Bc78;g__EPR3968-O8a-Bc78;s__uncultured_bacterium | 0 |
| d__Bacteria;p__Proteobacteria;c__Alphaproteobacteria;o__Micropepsales;f__Micropepsaceae;__;__ | 0 |
| d__Bacteria;p__Actinobacteriota;c__Actinobacteria;o__Propionibacteriales;f__Nocardioidaceae;g__Actinopolymorpha;__ | 0 |
| d__Bacteria;p__Acidobacteriota;c__Vicinamibacteria;o__Vicinamibacterales;f__Vicinamibacteraceae;g__Luteitalea;__ | 0 |
| d__Bacteria;p__Firmicutes;c__Desulfitobacteriia;o__Desulfitobacteriales;f__Desulfitobacteriaceae;g__Desulfitobacterium;s__Desulfitobacterium_hafniense | 0 |
| d__Bacteria;p__Firmicutes;c__Clostridia;o__Peptostreptococcales-Tissierellales;f__Anaerovoracaceae;g__Anaerovorax;s__uncultured_bacterium | 0 |
| d__Bacteria;p__Proteobacteria;c__Alphaproteobacteria;o__Rhodospirillales;f__Rhodospirillaceae;g__uncultured;s__metagenome | 0 |
| d__Bacteria;p__Proteobacteria;c__Gammaproteobacteria;o__Burkholderiales;f__Burkholderiaceae;g__Lautropia;__ | 0 |
| d__Bacteria;p__Proteobacteria;c__Alphaproteobacteria;o__Defluviicoccales;f__Defluviicoccaceae;g__Defluviicoccus;__ | 0 |
| d__Bacteria;p__Acidobacteriota;c__Acidobacteriae;o__Acidobacteriales;f__Acidobacteriaceae_(Subgroup_1);g__Occallatibacter;__ | 0 |
| d__Bacteria;p__Bacteroidota;c__Bacteroidia;o__Chitinophagales;f__Chitinophagaceae;g__Flaviaesturariibacter;s__uncultured_bacterium | 0 |
| d__Bacteria;p__Firmicutes;c__Clostridia;o__Peptostreptococcales-Tissierellales;f__Sedimentibacteraceae;g__Sedimentibacter;__ | 0 |
| d__Bacteria;p__Firmicutes;c__Clostridia;o__Clostridiales;f__Clostridiaceae;g__Clostridium_sensu_stricto_1;s__Clostridium_sardiniense | 0 |
| d__Bacteria;p__Firmicutes;c__Bacilli;o__Bacillales;f__Bacillaceae;g__Gracilibacillus;__ | 0 |
| d__Bacteria;p__Proteobacteria;c__Alphaproteobacteria;o__Rickettsiales;f__Mitochondria;g__Mitochondria;s__Andalucia_godoyi | 0 |
| d__Bacteria;p__Actinobacteriota;c__Actinobacteria;o__Propionibacteriales;f__Propionibacteriaceae;g__Tessaracoccus;__ | 0 |
| d__Bacteria;p__Planctomycetota;c__Planctomycetes;o__Isosphaerales;f__Isosphaeraceae;g__Singulisphaera;__ | 0 |
| d__Bacteria;p__Bacteroidota;c__Bacteroidia;o__Chitinophagales;f__Chitinophagaceae;g__Flavitalea;__ | 0 |
| d__Bacteria;p__Firmicutes;c__Clostridia;o__Peptostreptococcales-Tissierellales;f__Sedimentibacteraceae;g__Sedimentibacter;s__uncultured_bacterium | 0 |
| d__Bacteria;p__Firmicutes;c__Clostridia;o__Oscillospirales;f__Ruminococcaceae;g__Anaerotruncus;__ | 0 |
| d__Bacteria;p__Firmicutes;c__Clostridia;o__Oscillospirales;f__Oscillospiraceae;g__uncultured;s__uncultured_rumen | 0 |
| d__Bacteria;p__Actinobacteriota;c__Thermoleophilia;o__Solirubrobacterales;f__Solirubrobacteraceae;__;__ | 0 |
| d__Bacteria;p__Proteobacteria;c__Gammaproteobacteria;o__Burkholderiales;f__Methylophilaceae;g__MM2;s__uncultured_bacterium | 0 |
| d__Bacteria;p__Firmicutes;c__Bacilli;o__Bacillales;f__Bacillaceae;g__Bacillus;s__Bacillus_oceanisediminis | 0 |
| d__Bacteria;p__Myxococcota;c__Polyangia;o__Polyangiales;f__Polyangiaceae;g__Sorangium;__ | 0 |
| d__Bacteria;p__Acidobacteriota;c__Acidobacteriae;o__Acidobacteriales;f__Acidobacteriaceae_(Subgroup_1);g__Terriglobus;__ | 0 |
| d__Bacteria;p__Planctomycetota;c__Planctomycetes;o__Gemmatales;f__Gemmataceae;g__Gemmata;__ | 0 |
| d__Bacteria;p__Acidobacteriota;c__Vicinamibacteria;o__Vicinamibacterales;f__uncultured;g__uncultured;s__uncultured_Acidobacteria | 0 |
| d__Bacteria;p__Proteobacteria;c__Gammaproteobacteria;o__Coxiellales;f__Coxiellaceae;g__Coxiella;s__Coxiella_burnetii | 0 |
| d__Bacteria;p__Dependentiae;c__Babeliae;o__Babeliales;f__UBA12409;g__UBA12409;s__uncultured_bacterium | 0 |
| d__Bacteria;p__Firmicutes;c__Clostridia;o__Oscillospirales;f__Oscillospiraceae;g__Papillibacter;__ | 0 |
| d__Bacteria;p__Firmicutes;c__Bacilli;o__Bacillales;f__Bacillaceae;g__Geobacillus;__ | 0 |
| d__Bacteria;p__Firmicutes;c__Bacilli;o__Bacillales;f__Planococcaceae;__;__ | 0 |
| d__Bacteria;p__Actinobacteriota;c__Actinobacteria;o__Micrococcales;f__Intrasporangiaceae;__;__ | 0 |
| d__Bacteria;p__Myxococcota;c__Polyangia;o__Polyangiales;f__Sandaracinaceae;g__uncultured;s__uncultured_bacterium | 0 |
| d__Bacteria;p__Firmicutes;c__Limnochordia;o__Limnochordia;f__Limnochordia;g__Limnochordia;s__uncultured_bacterium | 0 |
| d__Bacteria;p__Actinobacteriota;c__Actinobacteria;o__Propionibacteriales;f__Nocardioidaceae;g__Nocardioides;s__Nocardioides_albus | 0 |
| d__Bacteria;p__Firmicutes;c__Clostridia;o__Lachnospirales;f__Lachnospiraceae;g__Roseburia;s__uncultured_bacterium | 0 |
| d__Bacteria;p__Firmicutes;c__Clostridia;o__Clostridiales;f__Oxobacteraceae;g__Oxobacter;__ | 0 |
| d__Bacteria;p__Firmicutes;c__Bacilli;o__Bacillales;f__Planococcaceae;g__Rummeliibacillus;__ | 0 |
| d__Bacteria;p__Firmicutes;c__Clostridia;o__Eubacteriales;f__Garciellaceae;g__Irregularibacter;__ | 0 |
| d__Bacteria;p__Firmicutes;c__Clostridia;__;__;__;__ | 0 |
| d__Bacteria;p__Proteobacteria;c__Alphaproteobacteria;o__Rhizobiales;f__Beijerinckiaceae;g__alphaI_cluster;__ | 0 |
| d__Bacteria;p__Bacteroidota;c__Bacteroidia;o__Sphingobacteriales;f__env.OPS_17;g__env.OPS_17;s__uncultured_bacterium | 0 |
| d__Bacteria;p__Actinobacteriota;c__Actinobacteria;o__Micrococcales;f__Intrasporangiaceae;g__Knoellia;s__Knoellia_sp. | 0 |
| d__Bacteria;p__Firmicutes;c__Bacilli;o__Thermoactinomycetales;f__Thermoactinomycetaceae;g__Thermoflavimicrobium;__ | 0 |
| d__Bacteria;p__Bacteroidota;c__Bacteroidia;o__Sphingobacteriales;f__Sphingobacteriaceae;g__Pedobacter;s__uncultured_bacterium | 0 |
| d__Bacteria;p__Armatimonadota;c__Fimbriimonadia;o__Fimbriimonadales;f__Fimbriimonadaceae;g__Fimbriimonadaceae;s__uncultured_bacterium | 0 |
| d__Bacteria;p__Firmicutes;c__Clostridia;o__Lachnospirales;f__Defluviitaleaceae;g__Defluviitalea;__ | 0 |
| d__Bacteria;p__Proteobacteria;c__Alphaproteobacteria;o__Rhizobiales;f__KF-JG30-B3;g__KF-JG30-B3;__ | 0 |
| d__Bacteria;p__Acidobacteriota;c__Acidobacteriae;o__Bryobacterales;f__Bryobacteraceae;g__Bryobacter;s__uncultured_Acidobacteria | 0 |
| d__Bacteria;p__Firmicutes;c__Thermacetogenia;o__Thermacetogeniales;f__Thermacetogeniaceae;g__uncultured;s__uncultured_bacterium | 0 |
| d__Bacteria;p__Firmicutes;c__Bacilli;o__Bacillales;f__Bacillaceae;g__uncultured;s__uncultured_compost | 0 |
| d__Bacteria;p__Chloroflexi;c__OLB14;o__OLB14;f__OLB14;g__OLB14;__ | 0 |
| d__Bacteria;p__Firmicutes;c__Clostridia;o__Peptostreptococcales-Tissierellales;f__Thermotaleaceae;g__Crassaminicella;__ | 0 |
| d__Bacteria;p__Proteobacteria;c__Alphaproteobacteria;o__Sphingomonadales;f__Sphingomonadaceae;__;__ | 0 |
| d__Bacteria;p__Bacteroidota;c__Bacteroidia;o__Cytophagales;f__Cytophagaceae;g__Rhodocytophaga;__ | 0 |
| d__Bacteria;p__Acidobacteriota;c__Blastocatellia;o__Blastocatellales;f__Blastocatellaceae;g__uncultured;s__uncultured_bacterium | 0 |
| d__Bacteria;p__Firmicutes;c__Clostridia;o__Peptostreptococcales-Tissierellales;f__Anaerovoracaceae;g__Family_XIII_AD3011_group;s__uncultured_bacterium | 0 |
| d__Bacteria;p__Actinobacteriota;c__Actinobacteria;o__Frankiales;f__Nakamurellaceae;g__Nakamurella;__ | 0 |
| d__Bacteria;p__Bacteroidota;c__Bacteroidia;o__Chitinophagales;f__Chitinophagaceae;g__Edaphobaculum;s__metagenome | 0 |
| d__Bacteria;p__Firmicutes;c__Clostridia;o__Oscillospirales;f__Hungateiclostridiaceae;g__UCG-012;__ | 0 |
| d__Bacteria;p__Proteobacteria;c__Alphaproteobacteria;o__Acetobacterales;f__Acetobacteraceae;g__Acidisphaera;__ | 0 |
| d__Bacteria;p__Actinobacteriota;c__Actinobacteria;o__Micrococcales;f__Intrasporangiaceae;g__Janibacter;__ | 0 |
| d__Bacteria;p__Actinobacteriota;c__Acidimicrobiia;o__Microtrichales;f__Ilumatobacteraceae;g__uncultured;s__uncultured_bacterium | 0 |
| d__Bacteria;p__Firmicutes;c__Bacilli;o__Bacillales;f__Bacillaceae;g__Bacillus;s__uncultured_soil | 0 |
| d__Bacteria;p__Actinobacteriota;c__Actinobacteria;o__Micromonosporales;f__Micromonosporaceae;__;__ | 0 |
| d__Bacteria;p__Proteobacteria;c__Alphaproteobacteria;o__Rhizobiales;f__Xanthobacteraceae;g__Rhodopseudomonas;s__Rhodopseudomonas_palustris | 0 |
| d__Bacteria;p__Firmicutes;c__Clostridia;o__Peptostreptococcales-Tissierellales;f__Family_XI;g__Sporanaerobacter;s__uncultured_bacterium | 0 |
| d__Bacteria;p__Proteobacteria;c__Gammaproteobacteria;o__Methylococcales;f__Methylococcaceae;g__Methylocaldum;__ | 0 |
| d__Bacteria;p__Actinobacteriota;c__Actinobacteria;o__Frankiales;f__Frankiaceae;g__Jatrophihabitans;s__uncultured_bacterium | 0 |
| d__Bacteria;p__Firmicutes;c__Desulfotomaculia;o__Desulfotomaculales;f__Pelotomaculaceae;g__Pelotomaculum;__ | 0 |
| d__Bacteria;p__Actinobacteriota;c__Actinobacteria;o__Streptomycetales;f__Streptomycetaceae;g__Streptomyces;__ | 0 |
| d__Bacteria;p__Proteobacteria;c__Alphaproteobacteria;o__Sphingomonadales;f__Sphingomonadaceae;g__Hephaestia;s__uncultured_bacterium | 0 |
| d__Bacteria;p__Firmicutes;c__Clostridia;o__Peptococcales;f__Peptococcaceae;g__uncultured;__ | 0 |
| d__Bacteria;p__Firmicutes;c__Bacilli;o__Paenibacillales;f__Paenibacillaceae;g__Paenibacillus;s__Paenibacillus_yonginensis | 0 |
| d__Bacteria;p__Chloroflexi;c__AD3;o__AD3;f__AD3;g__AD3;s__uncultured_Thermomicrobia | 0 |
| d__Bacteria;p__Firmicutes;c__Clostridia;o__Lachnospirales;f__Lachnospiraceae;g__Anaerosporobacter;__ | 0 |
| d__Bacteria;p__Firmicutes;c__Desulfitobacteriia;o__Desulfitobacteriales;f__Desulfitobacteriaceae;g__Desulfosporosinus;s__uncultured_bacterium | 0 |
| d__Bacteria;p__Firmicutes;c__Limnochordia;o__Limnochordales;f__Limnochordaceae;g__Limnochorda;__ | 0 |
| d__Bacteria;p__Proteobacteria;c__Gammaproteobacteria;o__Xanthomonadales;f__Xanthomonadaceae;g__Luteimonas;s__uncultured_bacterium | 0 |
| d__Bacteria;p__Firmicutes;c__Clostridia;o__Clostridiales;f__Clostridiaceae;g__Clostridium_sensu_stricto_6;s__Clostridium_bornimense | 0 |
| d__Bacteria;p__Firmicutes;c__Thermoanaerobacteria;o__Thermoanaerobacterales;f__SRB2;g__SRB2;__ | 0 |
| d__Bacteria;p__Proteobacteria;c__Alphaproteobacteria;o__Reyranellales;f__Reyranellaceae;g__Reyranella;__ | 0 |
| d__Bacteria;p__Firmicutes;c__Clostridia;o__Lachnospirales;f__Lachnospiraceae;g__Anaerocolumna;s__uncultured_bacterium | 0 |
| d__Bacteria;p__Firmicutes;c__Clostridia;o__Lachnospirales;f__Lachnospiraceae;g__Lachnospiraceae_NK4A136_group;s__uncultured_bacterium | 0 |
| d__Bacteria;p__Firmicutes;c__Bacilli;o__Bacillales;f__Bacillaceae;g__Anoxybacillus;s__Anoxybacillus_flavithermus | 0 |
| d__Bacteria;p__Proteobacteria;c__Alphaproteobacteria;o__Sphingomonadales;f__Sphingomonadaceae;g__Sphingopyxis;__ | 0 |
| d__Bacteria;p__Proteobacteria;c__Alphaproteobacteria;o__Dongiales;f__Dongiaceae;g__Dongia;s__uncultured_bacterium | 0 |
| d__Bacteria;p__Verrucomicrobiota;c__Verrucomicrobiae;o__Opitutales;f__Opitutaceae;g__Lacunisphaera;s__metagenome | 0 |
| d__Bacteria;p__Firmicutes;c__Thermovenabulia;o__Thermovenabulales;f__Thermovenabulales;g__Tepidanaerobacter;__ | 0 |
| d__Bacteria;p__Planctomycetota;c__Planctomycetes;o__Planctomycetales;f__Rubinisphaeraceae;g__SH-PL14;s__uncultured_bacterium | 0 |
| d__Bacteria;p__Firmicutes;c__Bacilli;o__Bacillales;f__Bacillaceae;g__Bacillus;s__Bacillus_smithii | 0 |
| d__Bacteria;p__Verrucomicrobiota;c__Verrucomicrobiae;o__Opitutales;f__Opitutaceae;g__Opitutus;s__uncultured_Opitutus | 0 |
| d__Bacteria;p__Proteobacteria;c__Alphaproteobacteria;o__Elsterales;f__uncultured;g__uncultured;s__uncultured_bacterium | 0 |
| d__Bacteria;p__Proteobacteria;c__Gammaproteobacteria;o__Burkholderiales;f__Burkholderiaceae;g__Limnobacter;__ | 0 |
| d__Bacteria;p__Firmicutes;c__Bacilli;o__Bacillales;f__Bacillaceae;g__Bacillus;s__Bacillus_simplex | 0 |
| d__Bacteria;p__Firmicutes;c__Bacilli;o__Bacillales;f__Bacillaceae;g__Oceanobacillus;s__uncultured_bacterium | 0 |
| d__Bacteria;p__Dependentiae;c__Babeliae;o__Babeliales;f__Babeliaceae;g__Babeliaceae;s__uncultured_bacterium | 0 |
| d__Bacteria;p__Planctomycetota;c__Planctomycetes;o__Gemmatales;f__Gemmataceae;g__Gemmata;s__uncultured_bacterium | 0 |
| d__Bacteria;p__Proteobacteria;c__Gammaproteobacteria;o__Diplorickettsiales;f__Diplorickettsiaceae;g__Aquicella;s__uncultured_Aquicella | 0 |
| d__Bacteria;p__Proteobacteria;c__Gammaproteobacteria;o__Burkholderiales;f__Oxalobacteraceae;g__Herbaspirillum;s__Herbaspirillum_seropedicae | 0 |
| d__Bacteria;p__Proteobacteria;c__Alphaproteobacteria;o__Rhizobiales;f__Rhizobiaceae;g__Allorhizobium-Neorhizobium-Pararhizobium-Rhizobium;s__metagenome | 0 |
| d__Bacteria;p__Proteobacteria;c__Alphaproteobacteria;o__Rhizobiales;f__Methyloligellaceae;g__Methyloceanibacter;s__uncultured_bacterium | 0 |
| d__Bacteria;p__Firmicutes;c__Clostridia;o__Lachnospirales;f__Lachnospiraceae;g__[Eubacterium]_ruminantium_group;s__uncultured_bacterium | 0 |
| d__Bacteria;p__Firmicutes;c__Desulfotomaculia;o__Desulfotomaculales;f__Desulfotomaculaceae;g__Desulfohalotomaculum;__ | 0 |
| d__Bacteria;p__Proteobacteria;c__Alphaproteobacteria;o__Rhizobiales;f__Beijerinckiaceae;g__uncultured;__ | 0 |
| d__Bacteria;p__Firmicutes;c__Clostridia;o__Peptostreptococcales-Tissierellales;f__Family_XI;g__Tissierella;s__uncultured_Tissierella | 0 |
| d__Bacteria;p__Proteobacteria;c__Alphaproteobacteria;o__uncultured;f__uncultured;g__uncultured;s__uncultured_bacterium | 0 |
| d__Bacteria;p__Firmicutes;c__Limnochordia;o__Limnochordales;f__Limnochordaceae;g__Limnochordaceae;s__uncultured_compost | 0 |
| d__Bacteria;p__Actinobacteriota;c__Actinobacteria;o__Pseudonocardiales;f__Pseudonocardiaceae;g__Kutzneria;__ | 0 |
| d__Bacteria;p__Firmicutes;c__Desulfotomaculia;o__Desulfotomaculales;f__Desulfotomaculales_Incertae_Sedis;__;__ | 0 |
| d__Bacteria;p__Firmicutes;c__Clostridia;o__Caldicoprobacterales;f__Caldicoprobacteraceae;g__Caldicoprobacter;__ | 0 |
| d__Bacteria;p__Planctomycetota;c__Planctomycetes;o__Isosphaerales;f__Isosphaeraceae;g__Tundrisphaera;__ | 0 |
| d__Bacteria;p__Actinobacteriota;c__Actinobacteria;o__Kineosporiales;f__Kineosporiaceae;g__Angustibacter;__ | 0 |
| d__Bacteria;p__Bacteroidota;c__Bacteroidia;o__Cytophagales;f__Microscillaceae;g__uncultured;s__uncultured_bacterium | 0 |
| d__Bacteria;p__Proteobacteria;c__Alphaproteobacteria;o__Rhizobiales;f__Xanthobacteraceae;g__Rhodopseudomonas;__ | 0 |
| d__Bacteria;p__Proteobacteria;c__Alphaproteobacteria;o__Rhizobiales;f__Rhizobiaceae;g__Aliihoeflea;__ | 0 |
| d__Bacteria;p__Firmicutes;c__Clostridia;o__Oscillospirales;f__Ethanoligenenaceae;__;__ | 0 |
| d__Bacteria;p__Firmicutes;c__Bacilli;o__Bacillales;f__Planococcaceae;g__Bhargavaea;__ | 0 |
| d__Bacteria;p__Firmicutes;c__Bacilli;o__Bacillales;f__Bacillaceae;g__Terribacillus;s__Terribacillus_goriensis | 0 |
| d__Bacteria;p__Planctomycetota;c__Phycisphaerae;o__Phycisphaerales;f__Phycisphaeraceae;g__uncultured;s__uncultured_bacterium | 0 |
| d__Bacteria;p__Firmicutes;c__Clostridia;o__uncultured;f__uncultured;g__uncultured;__ | 0 |
| d__Bacteria;p__Cyanobacteria;c__Vampirivibrionia;o__Vampirovibrionales;f__Vampirovibrionaceae;g__Vampirovibrio;__ | 0 |
| d__Bacteria;p__Actinobacteriota;c__Actinobacteria;o__Frankiales;f__uncultured;g__uncultured;s__uncultured_bacterium | 0 |
| d__Bacteria;p__Firmicutes;c__Clostridia;o__Oscillospirales;f__Oscillospiraceae;g__Sporobacter;s__uncultured_bacterium | 0 |
| d__Bacteria;p__Proteobacteria;c__Alphaproteobacteria;o__Rhizobiales;__;__;__ | 0 |
| d__Bacteria;p__Firmicutes;c__Symbiobacteriia;o__Symbiobacteriales;f__Symbiobacteraceae;g__Symbiobacterium;s__Symbiobacterium_thermophilum | 0 |
| d__Bacteria;p__Proteobacteria;c__Gammaproteobacteria;o__Burkholderiales;f__Nitrosomonadaceae;g__MND1;__ | 0 |
| d__Bacteria;p__Actinobacteriota;c__Actinobacteria;o__Pseudonocardiales;f__Pseudonocardiaceae;g__Amycolatopsis;__ | 0 |
| d__Bacteria;p__Proteobacteria;c__Alphaproteobacteria;o__Reyranellales;f__Reyranellaceae;g__uncultured;__ | 0 |
| d__Bacteria;p__Chloroflexi;c__AD3;o__AD3;f__AD3;g__AD3;__ | 0 |
| d__Bacteria;p__Proteobacteria;c__Alphaproteobacteria;o__Ferrovibrionales;f__Ferrovibrionaceae;g__Ferrovibrio;__ | 0 |
| d__Bacteria;p__Actinobacteriota;c__Actinobacteria;o__Micrococcales;f__Microbacteriaceae;g__Microbacterium;__ | 0 |
| d__Bacteria;p__Firmicutes;c__Bacilli;o__Bacillales;f__Planococcaceae;g__Lysinibacillus;s__uncultured_bacterium | 0 |
| d__Bacteria;p__Verrucomicrobiota;c__Verrucomicrobiae;o__Opitutales;f__Opitutaceae;g__Opitutus;s__uncultured_bacterium | 0 |
| d__Bacteria;p__Proteobacteria;c__Alphaproteobacteria;o__Azospirillales;f__Azospirillaceae;g__Azospirillum;__ | 0 |
| d__Bacteria;p__Proteobacteria;c__Gammaproteobacteria;o__Burkholderiales;f__Hydrogenophilaceae;g__Thiobacillus;s__uncultured_bacterium | 0 |
| d__Bacteria;p__Actinobacteriota;c__Acidimicrobiia;o__Microtrichales;f__Ilumatobacteraceae;g__uncultured;__ | 0 |
| d__Bacteria;p__Firmicutes;c__Clostridia;o__Lachnospirales;f__Defluviitaleaceae;g__Defluviitalea;s__uncultured_bacterium | 0 |
| d__Bacteria;p__Myxococcota;c__Myxococcia;o__Myxococcales;f__Myxococcaceae;g__Myxococcus;__ | 0 |
| d__Bacteria;p__Firmicutes;c__Clostridia;o__Christensenellales;f__Christensenellaceae;g__Christensenellaceae_R-7_group;s__uncultured_bacterium | 0 |
| d__Bacteria;p__Firmicutes;c__Bacilli;o__Alicyclobacillales;f__Alicyclobacillaceae;g__Alicyclobacillus;s__uncultured_bacterium | 0 |
| d__Bacteria;p__Firmicutes;c__Limnochordia;o__uncultured;f__uncultured;g__uncultured;s__uncultured_bacterium | 0 |
| d__Bacteria;p__Actinobacteriota;c__Actinobacteria;o__Micrococcales;f__Dermacoccaceae;g__Flexivirga;__ | 0 |
| d__Bacteria;p__Bacteroidota;c__Bacteroidia;o__Chitinophagales;f__Chitinophagaceae;g__Parafilimonas;s__uncultured_bacterium | 0 |
| d__Bacteria;p__Firmicutes;c__Clostridia;o__Peptostreptococcales-Tissierellales;f__Family_XI;g__Tepidimicrobium;s__Clostridiaceae_bacterium | 0 |
| d__Bacteria;p__Firmicutes;c__Limnochordia;__;__;__;__ | 0 |
| d__Bacteria;p__Proteobacteria;c__Alphaproteobacteria;o__uncultured;f__uncultured;g__uncultured;__ | 0 |
| d__Bacteria;p__Proteobacteria;c__Gammaproteobacteria;o__Burkholderiales;f__Rhodocyclaceae;g__uncultured;__ | 0 |
| d__Bacteria;p__Bdellovibrionota;c__Oligoflexia;o__Silvanigrellales;f__Silvanigrellaceae;g__uncultured;s__uncultured_bacterium | 0 |
| d__Bacteria;p__Firmicutes;c__Clostridia;o__Oscillospirales;f__Oscillospiraceae;g__NK4A214_group;__ | 0 |
| d__Bacteria;p__Cyanobacteria;c__Cyanobacteriia;o__Chloroplast;f__Chloroplast;g__Chloroplast;__ | 0 |
| d__Bacteria;p__Actinobacteriota;c__Actinobacteria;o__Frankiales;f__Geodermatophilaceae;g__Geodermatophilus;__ | 0 |
| d__Bacteria;p__Proteobacteria;c__Alphaproteobacteria;o__Acetobacterales;f__Acetobacteraceae;g__Roseomonas;__ | 0 |
| d__Bacteria;p__Bacteroidota;c__Bacteroidia;o__Chitinophagales;f__Chitinophagaceae;g__Taibaiella;__ | 0 |
| d__Bacteria;p__Firmicutes;c__Bacilli;o__Bacillales;f__Bacillaceae;g__Paucisalibacillus;__ | 0 |
| d__Bacteria;p__Verrucomicrobiota;c__Verrucomicrobiae;o__Verrucomicrobiales;f__Verrucomicrobiaceae;g__Prosthecobacter;__ | 0 |
| d__Bacteria;p__Proteobacteria;c__Alphaproteobacteria;o__Sphingomonadales;f__Sphingomonadaceae;g__Hephaestia;s__Sphingomonas_sp. | 0 |
| d__Bacteria;p__Firmicutes;c__Symbiobacteriia;o__Symbiobacteriales;f__uncultured;g__uncultured;__ | 0 |
| d__Bacteria;p__Proteobacteria;c__Alphaproteobacteria;o__Sphingomonadales;f__Sphingomonadaceae;g__Ellin6055;__ | 0 |
| d__Bacteria;p__Proteobacteria;c__Alphaproteobacteria;o__Acetobacterales;f__Acetobacteraceae;__;__ | 0 |
| d__Bacteria;p__Proteobacteria;c__Alphaproteobacteria;o__Micavibrionales;f__uncultured;g__uncultured;s__uncultured_bacterium | 0 |
| d__Bacteria;p__Proteobacteria;c__Alphaproteobacteria;o__Rhizobiales;f__Beijerinckiaceae;g__Methylorosula;__ | 0 |
| d__Bacteria;p__Acidobacteriota;c__Vicinamibacteria;o__Vicinamibacterales;f__uncultured;g__uncultured;s__uncultured_bacterium | 0 |
| d__Bacteria;p__Proteobacteria;c__Alphaproteobacteria;o__Caulobacterales;f__Caulobacteraceae;g__Brevundimonas;s__metagenome | 0 |
| d__Bacteria;p__Bacteroidota;c__Bacteroidia;o__Cytophagales;f__Hymenobacteraceae;g__Adhaeribacter;s__uncultured_bacterium | 0 |
| d__Bacteria;p__Proteobacteria;c__Alphaproteobacteria;o__Rhizobiales;f__Hyphomicrobiaceae;g__uncultured;__ | 0 |
| d__Bacteria;p__Proteobacteria;c__Gammaproteobacteria;o__Enterobacterales;f__Enterobacteriaceae;g__Escherichia-Shigella;__ | 0 |
| d__Bacteria;p__Actinobacteriota;c__Thermoleophilia;o__Gaiellales;f__Gaiellaceae;g__Gaiella;s__uncultured_bacterium | 0 |
| d__Bacteria;p__Acidobacteriota;c__Vicinamibacteria;o__Vicinamibacterales;__;__;__ | 0 |
| d__Bacteria;p__Bacteroidota;c__Bacteroidia;o__Sphingobacteriales;f__env.OPS_17;g__env.OPS_17;__ | 0 |
| d__Bacteria;p__Bacteroidota;c__Bacteroidia;o__Chitinophagales;f__Chitinophagaceae;g__Edaphobaculum;__ | 0 |
| d__Bacteria;p__Proteobacteria;c__Gammaproteobacteria;o__1013-28-CG33;f__1013-28-CG33;g__1013-28-CG33;s__uncultured_bacterium | 0 |
| d__Bacteria;p__Actinobacteriota;c__Actinobacteria;o__Micrococcales;f__Micrococcaceae;__;__ | 0 |
| d__Bacteria;p__Firmicutes;c__Desulfotomaculia;o__Desulfotomaculales;f__Desulfotomaculales_Incertae_Sedis;g__Desulfallas-Sporotomaculum;__ | 0 |
| d__Bacteria;p__Firmicutes;c__Clostridia;o__Lachnospirales;f__Lachnospiraceae;g__Anaerocolumna;__ | 0 |
| d__Bacteria;p__Firmicutes;c__Clostridia;o__Oscillospirales;f__[Eubacterium]_coprostanoligenes_group;g__[Eubacterium]_coprostanoligenes_group;s__uncultured_bacterium | 0 |
| d__Bacteria;p__Planctomycetota;c__Planctomycetes;o__Pirellulales;f__Pirellulaceae;g__Pirellula;s__uncultured_bacterium | 0 |
| d__Bacteria;p__Acidobacteriota;c__Blastocatellia;o__11-24;f__11-24;g__11-24;s__uncultured_bacterium | 0 |
| d__Bacteria;p__Proteobacteria;c__Alphaproteobacteria;o__Micropepsales;f__Micropepsaceae;g__Rhizomicrobium;__ | 0 |
| d__Bacteria;p__Gemmatimonadota;c__Gemmatimonadetes;o__Gemmatimonadales;f__Gemmatimonadaceae;g__uncultured;__ | 0 |
| d__Bacteria;p__Deinococcota;c__Deinococci;o__Deinococcales;f__Trueperaceae;g__Truepera;s__uncultured_bacterium | 0 |
| d__Bacteria;p__Actinobacteriota;c__Actinobacteria;o__Micrococcales;f__Microbacteriaceae;g__uncultured;__ | 0 |
| d__Bacteria;p__Proteobacteria;c__Alphaproteobacteria;__;__;__;__ | 0 |
| d__Bacteria;p__Firmicutes;c__Clostridia;o__Monoglobales;f__Monoglobaceae;g__Monoglobus;__ | 0 |
| d__Bacteria;p__Actinobacteriota;c__Thermoleophilia;o__Solirubrobacterales;f__Solirubrobacteraceae;g__Solirubrobacter;__ | 0 |
| d__Bacteria;p__Halanaerobiaeota;c__Halanaerobiia;o__Halanaerobiales;f__Halanaerobiaceae;g__Halocella;s__uncultured_bacterium | 0 |
| d__Bacteria;p__Proteobacteria;c__Alphaproteobacteria;o__Rhodospirillales;f__Magnetospirillaceae;__;__ | 0 |
| d__Bacteria;p__Proteobacteria;c__Alphaproteobacteria;o__Puniceispirillales;__;__;__ | 0 |
| d__Bacteria;p__Actinobacteriota;c__Actinobacteria;o__Micrococcales;f__Microbacteriaceae;g__Leifsonia;__ | 0 |
| d__Bacteria;p__Firmicutes;c__Moorellia;o__Desulfitibacterales;f__Desulfitibacteraceae;g__Desulfitibacter;s__uncultured_bacterium | 0 |
| d__Bacteria;p__Cyanobacteria;c__Sericytochromatia;o__Sericytochromatia;f__Sericytochromatia;g__Sericytochromatia;__ | 0 |
| d__Bacteria;p__Firmicutes;c__Thermacetogenia;o__Thermacetogeniales;f__Thermacetogeniaceae;g__Syntrophaceticus;__ | 0 |
| d__Bacteria;p__Firmicutes;c__Clostridia;o__Lachnospirales;f__Lachnospiraceae;g__[Eubacterium]_xylanophilum_group;s__uncultured_bacterium | 0 |
| d__Bacteria;p__Firmicutes;c__Clostridia;o__Oscillospirales;f__Hungateiclostridiaceae;g__UCG-012;s__uncultured_organism | 0 |
| d__Bacteria;p__Firmicutes;c__Clostridia;o__Oscillospirales;f__Hungateiclostridiaceae;g__Hungateiclostridium;s__Hungateiclostridium_thermocellum | 0 |
| d__Bacteria;p__Firmicutes;c__Clostridia;o__Clostridia_vadinBB60_group;f__Clostridia_vadinBB60_group;g__Clostridia_vadinBB60_group;s__uncultured_bacterium | 0 |
| d__Bacteria;p__Bacteroidota;c__Bacteroidia;o__Chitinophagales;f__Chitinophagaceae;g__Puia;s__uncultured_bacterium | 0 |
| d__Bacteria;p__Firmicutes;c__Desulfotomaculia;o__Desulfotomaculales;__;__;__ | 0 |
| d__Bacteria;p__Firmicutes;c__Clostridia;o__Clostridiales;f__Clostridiaceae;g__Clostridium_sensu_stricto_7;s__Clostridium_novyi | 0 |
| d__Bacteria;p__Proteobacteria;c__Alphaproteobacteria;o__Rhizobiales;f__Beijerinckiaceae;g__Methylovirgula;__ | 0 |
| d__Bacteria;p__Chloroflexi;c__Chloroflexia;o__Thermomicrobiales;f__Thermomicrobiaceae;g__Nitrolancea;s__uncultured_bacterium | 0 |
| d__Bacteria;p__Verrucomicrobiota;c__Verrucomicrobiae;o__Opitutales;f__Opitutaceae;__;__ | 0 |
| d__Bacteria;p__Proteobacteria;c__Alphaproteobacteria;o__Rhodobacterales;f__Rhodobacteraceae;__;__ | 0 |
| d__Bacteria;p__Proteobacteria;c__Alphaproteobacteria;o__Rhizobiales;f__Hyphomicrobiaceae;g__uncultured;s__uncultured_bacterium | 0 |
| d__Bacteria;p__Firmicutes;c__Bacilli;o__Izemoplasmatales;f__Izemoplasmatales;g__Izemoplasmatales;s__uncultured_bacterium | 0 |
| d__Bacteria;p__Bacteroidota;c__Bacteroidia;o__Chitinophagales;f__Chitinophagaceae;g__Edaphobaculum;s__uncultured_bacterium | 0 |
| d__Bacteria;p__Firmicutes;c__Bacilli;o__Alicyclobacillales;f__Alicyclobacillaceae;g__Tumebacillus;s__Tumebacillus_ginsengisoli | 0 |
| d__Bacteria;p__Proteobacteria;c__Alphaproteobacteria;o__Rhodospirillales;f__Magnetospiraceae;g__uncultured;s__uncultured_bacterium | 0 |
| d__Bacteria;p__Dependentiae;c__Babeliae;o__Babeliales;f__Babeliaceae;g__Babeliaceae;__ | 0 |
| d__Bacteria;p__Proteobacteria;c__Gammaproteobacteria;o__Piscirickettsiales;f__Piscirickettsiaceae;g__Candidatus_Endoecteinascidia;s__uncultured_bacterium | 0 |
| d__Bacteria;p__Chloroflexi;c__OLB14;o__OLB14;f__OLB14;g__OLB14;s__uncultured_bacterium | 0 |
| d__Bacteria;p__Firmicutes;c__Clostridia;o__Clostridiales;f__Clostridiaceae;g__Clostridium_sensu_stricto_18;s__Clostridium_botulinum | 0 |
| d__Bacteria;p__Planctomycetota;c__Planctomycetes;o__Isosphaerales;f__Isosphaeraceae;g__Paludisphaera;s__uncultured_bacterium | 0 |
| d__Bacteria;p__Firmicutes;c__Bacilli;o__Bacillales;f__Bacillaceae;g__Pseudogracilibacillus;s__uncultured_bacterium | 0 |
| d__Bacteria;p__Firmicutes;c__Desulfitobacteriia;o__Desulfitobacteriales;f__Syntrophobotulaceae;g__Dehalobacter;__ | 0 |
| d__Bacteria;p__Firmicutes;c__Bacilli;o__Bacillales;f__Bacillaceae;g__Geobacillus;s__Geobacillus_thermodenitrificans | 0 |
| d__Bacteria;p__Proteobacteria;c__Alphaproteobacteria;o__Rhizobiales;f__Rhizobiaceae;g__Shinella;__ | 0 |
| d__Bacteria;p__Chloroflexi;c__AD3;o__AD3;f__AD3;g__AD3;s__uncultured_bacterium | 0 |
| d__Bacteria;p__Proteobacteria;c__Gammaproteobacteria;o__Beggiatoales;f__Beggiatoaceae;g__uncultured;s__uncultured_bacterium | 0 |
| d__Bacteria;p__Firmicutes;c__Bacilli;o__Paenibacillales;f__Paenibacillaceae;g__Paenibacillus;s__Paenibacillus_larvae | 0 |
| d__Bacteria;p__Firmicutes;c__Clostridia;o__Lachnospirales;f__Lachnospiraceae;g__Lachnospiraceae_UCG-007;s__uncultured_bacterium | 0 |
| d__Bacteria;p__Proteobacteria;c__Alphaproteobacteria;o__Rhizobiales;f__Rhizobiaceae;g__Pseudaminobacter;s__uncultured_bacterium | 0 |
| d__Bacteria;p__Proteobacteria;c__Alphaproteobacteria;o__Sphingomonadales;f__Sphingomonadaceae;g__Novosphingobium;s__Novosphingobium_subterraneum | 0 |
| d__Bacteria;p__Proteobacteria;c__Gammaproteobacteria;o__Nitrosococcales;f__Nitrosococcaceae;g__wb1-P19;s__uncultured_bacterium | 0 |
| d__Bacteria;p__Proteobacteria;c__Alphaproteobacteria;o__Micropepsales;f__Micropepsaceae;g__Micropepsis;s__uncultured_bacterium | 0 |
| d__Bacteria;p__Proteobacteria;c__Alphaproteobacteria;o__Rhizobiales;f__Xanthobacteraceae;g__uncultured;s__metagenome | 0 |
| d__Bacteria;p__Firmicutes;c__Clostridia;o__Lachnospirales;f__Lachnospiraceae;g__Anaerosporobacter;s__uncultured_bacterium | 0 |
| d__Bacteria;p__Proteobacteria;c__Gammaproteobacteria;o__Burkholderiales;f__Nitrosomonadaceae;g__MND1;s__uncultured_bacterium | 0 |
| d__Bacteria;p__Bacteroidota;c__Bacteroidia;o__Chitinophagales;f__Chitinophagaceae;g__Ferruginibacter;s__uncultured_bacterium | 0 |
| d__Bacteria;p__Firmicutes;c__Clostridia;o__Lachnospirales;f__Lachnospiraceae;g__[Ruminococcus]_gauvreauii_group;s__uncultured_bacterium | 0 |
| d__Bacteria;p__Firmicutes;c__Bacilli;o__Paenibacillales;f__Paenibacillaceae;g__Thermobacillus;s__uncultured_bacterium | 0 |
| d__Bacteria;p__Proteobacteria;c__Alphaproteobacteria;o__Rhizobiales;f__Rhizobiaceae;g__Mesorhizobium;s__Mesorhizobium_sp. | 0 |
| d__Bacteria;p__Actinobacteriota;c__Actinobacteria;o__Micrococcales;f__Intrasporangiaceae;g__uncultured;s__uncultured_bacterium | 0 |
| d__Bacteria;p__Firmicutes;c__Desulfotomaculia;o__Desulfotomaculales;f__Pelotomaculaceae;g__Cryptanaerobacter;s__uncultured_bacterium | 0 |
| d__Bacteria;p__Actinobacteriota;c__Acidimicrobiia;o__Microtrichales;__;__;__ | 0 |
| d__Bacteria;p__Proteobacteria;c__Alphaproteobacteria;o__Caulobacterales;f__Caulobacteraceae;g__Caulobacter;s__uncultured_bacterium | 0 |
| d__Bacteria;p__Proteobacteria;c__Alphaproteobacteria;o__Caulobacterales;f__Caulobacteraceae;g__uncultured;s__uncultured_bacterium | 0 |
| d__Bacteria;p__Firmicutes;c__Thermaerobacteria;o__Thermaerobacterales;f__Thermaerobacteraceae;g__Thermaerobacter;__ | 0 |
| d__Bacteria;p__Proteobacteria;c__Alphaproteobacteria;o__Rhizobiales;f__uncultured;g__uncultured;s__uncultured_bacterium | 0 |
| d__Bacteria;p__Actinobacteriota;c__Actinobacteria;o__Micrococcales;f__Microbacteriaceae;g__Mycetocola;__ | 0 |
| d__Bacteria;p__Actinobacteriota;c__Actinobacteria;o__Propionibacteriales;f__Propionibacteriaceae;g__Microlunatus;s__uncultured_bacterium | 0 |
| d__Bacteria;p__Actinobacteriota;c__Actinobacteria;o__Propionibacteriales;f__Nocardioidaceae;__;__ | 0 |
| d__Bacteria;p__Firmicutes;c__Bacilli;o__Bacillales;f__Bacillaceae;g__uncultured;s__uncultured_bacterium | 0 |
| d__Bacteria;p__Proteobacteria;c__Gammaproteobacteria;o__Burkholderiales;f__Alcaligenaceae;g__Achromobacter;__ | 0 |
| d__Bacteria;p__Acidobacteriota;c__Acidobacteriae;o__Acidobacteriales;f__Acidobacteriaceae_(Subgroup_1);g__uncultured;s__uncultured_Acidobacteria | 0 |
| d__Bacteria;p__Proteobacteria;c__Gammaproteobacteria;o__Xanthomonadales;f__Rhodanobacteraceae;g__Dokdonella;s__uncultured_bacterium | 0 |
| d__Bacteria;p__Actinobacteriota;c__Thermoleophilia;o__Solirubrobacterales;f__Solirubrobacteraceae;g__Patulibacter;s__uncultured_bacterium | 0 |
| d__Bacteria;p__Verrucomicrobiota;c__Verrucomicrobiae;o__Pedosphaerales;f__Pedosphaeraceae;g__Ellin516;s__uncultured_bacterium | 0 |
| d__Bacteria;p__Firmicutes;c__Clostridia;o__Lachnospirales;f__Lachnospiraceae;g__Epulopiscium;s__uncultured_organism | 0 |
| d__Bacteria;p__Acidobacteriota;c__Acidobacteriae;o__Solibacterales;f__Solibacteraceae;g__Candidatus_Solibacter;__ | 0 |
| d__Bacteria;p__Proteobacteria;c__Alphaproteobacteria;o__Rickettsiales;f__Rickettsiaceae;__;__ | 0 |
| d__Bacteria;p__Firmicutes;c__Bacilli;o__Paenibacillales;f__Paenibacillaceae;g__Paenibacillus;s__Paenibacillus_polymyxa | 0 |
| d__Bacteria;p__Firmicutes;c__Clostridia;o__Clostridiales;f__Clostridiaceae;g__Clostridium_sensu_stricto_15;s__Clostridium_cochlearium | 0 |
| d__Bacteria;p__Firmicutes;c__Clostridia;o__Clostridiales;f__Clostridiaceae;g__Clostridium_sensu_stricto_1;s__Clostridium_isatidis | 0 |
| d__Bacteria;p__Proteobacteria;c__Gammaproteobacteria;o__Legionellales;f__Legionellaceae;g__Legionella;s__Legionella_pneumophila | 0 |
| d__Bacteria;p__Firmicutes;c__Bacilli;o__Thermoactinomycetales;f__Thermoactinomycetaceae;g__Novibacillus;s__Novibacillus_thermophilus | 0 |
| d__Bacteria;p__Firmicutes;c__Bacilli;o__Thermoactinomycetales;f__Thermoactinomycetaceae;g__Thermoactinomyces;__ | 0 |
| d__Bacteria;p__Acidobacteriota;c__Acidobacteriae;o__Acidobacteriales;f__Acidobacteriaceae_(Subgroup_1);g__Terracidiphilus;__ | 0 |
| d__Bacteria;p__Bdellovibrionota;c__Oligoflexia;o__Silvanigrellales;f__Silvanigrellaceae;__;__ | 0 |
| d__Bacteria;p__Acidobacteriota;c__Vicinamibacteria;o__Vicinamibacterales;f__Vicinamibacteraceae;g__Luteitalea;s__uncultured_bacterium | 0 |
| d__Bacteria;p__Actinobacteriota;c__Actinobacteria;o__Micromonosporales;f__Micromonosporaceae;g__Longispora;s__uncultured_bacterium | 0 |
| d__Bacteria;p__Proteobacteria;c__Gammaproteobacteria;o__Gammaproteobacteria_Incertae_Sedis;f__Unknown_Family;g__Candidatus_Berkiella;s__uncultured_bacterium | 0 |
| d__Bacteria;p__Gemmatimonadota;c__Gemmatimonadetes;o__Gemmatimonadales;f__Gemmatimonadaceae;g__Gemmatimonas;s__uncultured_bacterium | 0 |
| d__Bacteria;p__Firmicutes;c__Limnochordia;o__Limnochordales;f__uncultured;g__uncultured;s__uncultured_bacterium | 0 |
| d__Bacteria;p__Proteobacteria;c__Alphaproteobacteria;o__Rhizobiales;f__Methyloligellaceae;g__uncultured;__ | 0 |
| d__Bacteria;p__Firmicutes;c__Bacilli;o__Bacillales;f__Planococcaceae;g__Solibacillus;__ | 0 |
| d__Bacteria;p__Proteobacteria;c__Gammaproteobacteria;o__B2M28;f__B2M28;g__B2M28;s__uncultured_Coxiella | 0 |
| d__Bacteria;p__Firmicutes;c__Desulfotomaculia;o__Desulfotomaculales;f__Desulfotomaculales_Incertae_Sedis;g__Desulfallas-Sporotomaculum;s__uncultured_bacterium | 0 |
| d__Bacteria;p__Actinobacteriota;c__Actinobacteria;o__Corynebacteriales;f__Nocardiaceae;g__Rhodococcus;s__Rhodococcus_sp. | 0 |
| d__Bacteria;p__Verrucomicrobiota;c__Verrucomicrobiae;o__Chthoniobacterales;f__Chthoniobacteraceae;g__LD29;__ | 0 |
| d__Bacteria;p__Actinobacteriota;c__Actinobacteria;o__Micrococcales;f__Intrasporangiaceae;g__Ornithinimicrobium;__ | 0 |
| d__Bacteria;p__Firmicutes;c__Bacilli;o__Bacillales;__;__;__ | 0 |
| d__Bacteria;p__Firmicutes;c__Clostridia;o__Oscillospirales;f__Ruminococcaceae;g__[Eubacterium]_siraeum_group;s__uncultured_bacterium | 0 |
| d__Bacteria;p__Firmicutes;c__Bacilli;o__Bacillales;f__Bacillaceae;g__Fictibacillus;s__Fictibacillus_phosphorivorans | 0 |
| d__Bacteria;p__Proteobacteria;c__Gammaproteobacteria;o__Salinisphaerales;f__Solimonadaceae;__;__ | 0 |
| d__Bacteria;p__Proteobacteria;c__Alphaproteobacteria;o__Rhizobiales;f__uncultured;g__uncultured;__ | 0 |
| d__Bacteria;p__Verrucomicrobiota;c__Verrucomicrobiae;o__Pedosphaerales;f__Pedosphaeraceae;g__uncultured;s__uncultured_bacterium | 0 |
| d__Bacteria;p__Firmicutes;c__Clostridia;o__Oscillospirales;f__Ruminococcaceae;g__Caproiciproducens;__ | 0 |
| d__Bacteria;p__Bacteroidota;c__Bacteroidia;o__Chitinophagales;f__Chitinophagaceae;g__Arachidicoccus;__ | 0 |
| d__Bacteria;p__Proteobacteria;c__Gammaproteobacteria;o__Burkholderiales;f__Oxalobacteraceae;g__Herbaspirillum;__ | 0 |
| d__Bacteria;p__Firmicutes;c__Bacilli;o__Paenibacillales;f__Paenibacillaceae;g__Cohnella;s__uncultured_bacterium | 0 |
| d__Bacteria;p__Firmicutes;c__Bacilli;o__Bacillales;f__Bacillaceae;g__Bacillus;s__Bacillus_subtilis | 0 |
| d__Bacteria;p__Actinobacteriota;c__Thermoleophilia;o__Solirubrobacterales;f__Solirubrobacteraceae;g__Solirubrobacter;s__uncultured_bacterium | 0 |
| d__Bacteria;p__Firmicutes;c__Clostridia;o__Clostridiales;f__Clostridiaceae;g__Clostridium_sensu_stricto_8;s__uncultured_bacterium | 0 |
| d__Bacteria;p__Firmicutes;c__Moorellia;__;__;__;__ | 0 |
| d__Bacteria;p__Firmicutes;c__Negativicutes;o__Veillonellales-Selenomonadales;f__Sporomusaceae;__;__ | 0 |
| d__Bacteria;p__Proteobacteria;c__Gammaproteobacteria;o__Pseudomonadales;f__Pseudomonadaceae;g__Pseudomonas;s__Pseudomonas_sp. | 0 |
| d__Bacteria;p__Acidobacteriota;c__Vicinamibacteria;o__Vicinamibacterales;f__Vicinamibacteraceae;__;__ | 0 |
| d__Bacteria;p__Firmicutes;c__Clostridia;o__Lachnospirales;f__Lachnospiraceae;g__Anaerostignum;__ | 0 |
| d__Bacteria;p__Firmicutes;c__Bacilli;o__Bacillales;f__Bacillaceae;g__Bacillus;s__Bacillus_cereus | 0 |
| d__Bacteria;p__Proteobacteria;c__Alphaproteobacteria;o__Rickettsiales;__;__;__ | 0 |
| d__Bacteria;p__Chloroflexi;c__Dehalococcoidia;o__SAR202_clade;f__SAR202_clade;g__SAR202_clade;s__uncultured_bacterium | 0 |
| d__Bacteria;p__Myxococcota;c__Polyangia;o__Polyangiales;f__Sandaracinaceae;g__Sandaracinus;s__uncultured_bacterium | 0 |
| d__Bacteria;p__Planctomycetota;c__Planctomycetes;o__Isosphaerales;f__Isosphaeraceae;g__uncultured;s__uncultured_Singulisphaera | 0 |
| d__Bacteria;p__Actinobacteriota;c__Actinobacteria;o__Streptomycetales;f__Streptomycetaceae;g__Streptomyces;s__Streptomyces_sp. | 0 |
| d__Bacteria;p__Planctomycetota;c__Planctomycetes;o__Pirellulales;f__Pirellulaceae;g__Rhodopirellula;s__uncultured_bacterium | 0 |
| d__Bacteria;p__Proteobacteria;c__Alphaproteobacteria;o__Rhizobiales;f__Devosiaceae;g__Devosia;s__uncultured_bacterium | 0 |
| d__Bacteria;p__Proteobacteria;c__Alphaproteobacteria;o__Rhizobiales;f__Rhizobiaceae;g__Allorhizobium-Neorhizobium-Pararhizobium-Rhizobium;s__Rhizobium_sp. | 0 |
| d__Bacteria;p__Planctomycetota;c__Planctomycetes;o__Gemmatales;f__Gemmataceae;g__uncultured;s__uncultured_planctomycete | 0 |
| d__Bacteria;p__Firmicutes;c__Bacilli;o__Paenibacillales;f__Paenibacillaceae;g__Paenibacillus;s__Paenibacillus_mucilaginosus | 0 |
| d__Bacteria;p__Firmicutes;c__Bacilli;o__Bacillales;f__Bacillaceae;g__Ornithinibacillus;__ | 0 |
| d__Bacteria;p__Firmicutes;c__Clostridia;o__Oscillospirales;f__Ruminococcaceae;g__Caproiciproducens;s__uncultured_bacterium | 0 |
| d__Bacteria;p__Firmicutes;c__Bacilli;o__Bacillales;f__Bacillaceae;g__Anoxybacillus;__ | 0 |
| d__Bacteria;p__Firmicutes;c__Clostridia;o__Lachnospirales;f__Lachnospiraceae;g__[Eubacterium]_hallii_group;s__[Eubacterium]_hallii | 0 |
| d__Bacteria;p__Proteobacteria;c__Gammaproteobacteria;o__Gammaproteobacteria_Incertae_Sedis;f__Unknown_Family;__;__ | 0 |
| d__Bacteria;p__Proteobacteria;c__Gammaproteobacteria;o__Burkholderiales;f__SC-I-84;g__SC-I-84;s__uncultured_bacterium | 0 |
| d__Bacteria;p__Proteobacteria;c__Alphaproteobacteria;o__Rhizobiales;f__Hyphomicrobiaceae;g__Pedomicrobium;__ | 0 |
| d__Bacteria;p__Proteobacteria;c__Alphaproteobacteria;o__Sphingomonadales;f__Sphingomonadaceae;g__Sphingobium;s__uncultured_Alphaproteobacteria | 0 |
| d__Bacteria;p__Bacteroidota;c__Bacteroidia;o__Chitinophagales;f__Chitinophagaceae;g__Taibaiella;s__uncultured_bacterium | 0 |
| d__Bacteria;p__Firmicutes;c__Clostridia;o__Peptostreptococcales-Tissierellales;f__Family_XI;g__Tissierella;s__uncultured_bacterium | 0 |
| d__Bacteria;p__Firmicutes;c__Clostridia;o__Clostridiales;f__Clostridiaceae;g__Clostridium_sensu_stricto_13;__ | 0 |
| d__Bacteria;p__Bacteroidota;c__Bacteroidia;o__Sphingobacteriales;f__Sphingobacteriaceae;g__Arcticibacter;__ | 0 |
| d__Bacteria;p__Firmicutes;c__Clostridia;o__Peptostreptococcales-Tissierellales;f__Family_XI;g__Anaerosalibacter;__ | 0 |
| d__Bacteria;p__Firmicutes;c__Bacilli;o__Bacillales;f__Bacillaceae;g__Thalassobacillus;__ | 0 |
| d__Bacteria;p__Firmicutes;c__Clostridia;o__Clostridiales;f__Clostridiaceae;__;__ | 0 |
| d__Bacteria;p__Firmicutes;c__Bacilli;o__Bacillales;f__Bacillaceae;g__Virgibacillus;__ | 0 |
| d__Bacteria;p__Bacteroidota;c__Bacteroidia;o__Chitinophagales;f__Chitinophagaceae;g__Puia;__ | 0 |
| d__Bacteria;p__Firmicutes;c__Incertae_Sedis;o__DTU014;f__DTU014;g__DTU014;s__uncultured_Firmicutes | 0 |
| d__Bacteria;p__Firmicutes;c__Bacilli;o__Thermoactinomycetales;f__Thermoactinomycetaceae;__;__ | 0 |
| d__Bacteria;p__Proteobacteria;c__Gammaproteobacteria;o__Acidiferrobacterales;f__Acidiferrobacteraceae;g__Sulfurifustis;__ | 0 |
| d__Bacteria;p__Firmicutes;c__Bacilli;o__Bacillales;f__Bacillaceae;g__Halobacillus;__ | 0 |
| d__Bacteria;p__Actinobacteriota;c__Actinobacteria;o__Micrococcales;f__Microbacteriaceae;g__Mycetocola;s__Mycetocola_sp. | 0 |
| d__Bacteria;p__Proteobacteria;c__Alphaproteobacteria;o__Sphingomonadales;f__Sphingomonadaceae;g__Novosphingobium;s__uncultured_bacterium | 0 |
| d__Bacteria;p__Proteobacteria;c__Gammaproteobacteria;o__Burkholderiales;f__Nitrosomonadaceae;g__Ellin6067;s__uncultured_bacterium | 0 |
| d__Bacteria;p__Myxococcota;c__Polyangia;o__Haliangiales;f__Haliangiaceae;g__Haliangium;s__metagenome | 0 |
| d__Bacteria;p__Acidobacteriota;c__Acidobacteriae;o__Acidobacteriales;f__uncultured;g__uncultured;s__uncultured_Acidobacteriaceae | 0 |
| d__Bacteria;p__Firmicutes;c__Thermovenabulia;o__Thermovenabulales;f__Thermovenabulales;g__Tepidanaerobacter;s__uncultured_bacterium | 0 |
| d__Bacteria;p__Proteobacteria;c__Gammaproteobacteria;o__Burkholderiales;f__Alcaligenaceae;g__Parapusillimonas;s__uncultured_bacterium | 0 |
| d__Bacteria;p__Firmicutes;c__Clostridia;o__Oscillospirales;f__Hungateiclostridiaceae;g__uncultured;s__uncultured_bacterium | 0 |
| d__Bacteria;p__Myxococcota;c__Polyangia;o__Polyangiales;f__BIrii41;g__BIrii41;s__metagenome | 0 |
| d__Bacteria;p__Firmicutes;c__D8A-2;o__D8A-2;f__D8A-2;g__D8A-2;s__uncultured_bacterium | 0 |
| d__Bacteria;p__Proteobacteria;c__Gammaproteobacteria;o__Immundisolibacterales;f__Immundisolibacteraceae;g__Immundisolibacter;__ | 0 |
| d__Bacteria;p__Firmicutes;c__Bacilli;o__Paenibacillales;f__Paenibacillaceae;g__Paenibacillus;s__uncultured_Paenibacillus | 0 |
| d__Bacteria;p__Firmicutes;c__Desulfitobacteriia;o__Desulfitobacteriales;f__Desulfitobacteriaceae;g__Desulfosporosinus;s__Desulfosporosinus_youngiae | 0 |
| d__Bacteria;p__Proteobacteria;c__Gammaproteobacteria;o__Gammaproteobacteria_Incertae_Sedis;f__Unknown_Family;g__Acidibacter;s__uncultured_gamma | 0 |
| d__Bacteria;p__Proteobacteria;c__Gammaproteobacteria;o__Legionellales;f__Legionellaceae;g__Legionella;s__uncultured_bacterium | 0 |
| d__Bacteria;p__Proteobacteria;c__Alphaproteobacteria;o__Rhizobiales;f__D05-2;g__D05-2;s__uncultured_bacterium | 0 |
| d__Bacteria;p__Firmicutes;c__Clostridia;o__Oscillospirales;f__Hungateiclostridiaceae;g__uncultured;__ | 0 |
| d__Bacteria;p__Firmicutes;c__Clostridia;o__Oscillospirales;f__Ethanoligenenaceae;g__Ethanoligenens;__ | 0 |
| d__Bacteria;p__Actinobacteriota;c__Actinobacteria;o__Corynebacteriales;f__Nocardiaceae;g__Rhodococcus;__ | 0 |
| d__Bacteria;p__Proteobacteria;c__Alphaproteobacteria;o__Azospirillales;f__Azospirillaceae;g__Skermanella;s__uncultured_bacterium | 0 |
| d__Bacteria;p__Acidobacteriota;c__Acidobacteriae;o__Acidobacteriales;f__Acidobacteriaceae_(Subgroup_1);g__Edaphobacter;s__uncultured_Acidobacteria | 0 |
| d__Bacteria;p__Deinococcota;c__Deinococci;o__Deinococcales;f__Trueperaceae;g__Truepera;__ | 0 |
| d__Bacteria;p__Firmicutes;c__Bacilli;o__Thermoactinomycetales;f__Thermoactinomycetaceae;g__Planifilum;__ | 0 |
| d__Bacteria;p__Firmicutes;c__Bacilli;o__Aneurinibacillales;f__Aneurinibacillaceae;g__Aneurinibacillus;s__Aneurinibacillus_sp. | 0 |
| d__Bacteria;p__Proteobacteria;c__Alphaproteobacteria;o__Caulobacterales;f__Caulobacteraceae;g__Brevundimonas;s__Brevundimonas_diminuta | 0 |
| d__Bacteria;p__Actinobacteriota;c__Acidimicrobiia;o__Microtrichales;f__Microtrichaceae;g__uncultured;s__uncultured_bacterium | 0 |
| d__Bacteria;p__Proteobacteria;c__Gammaproteobacteria;o__Burkholderiales;f__Alcaligenaceae;g__Candidimonas;s__Candidimonas_humi | 0 |
| d__Bacteria;p__Actinobacteriota;c__Actinobacteria;o__Micrococcales;f__Micrococcaceae;g__Paeniglutamicibacter;__ | 0 |
| d__Bacteria;p__Firmicutes;c__Limnochordia;o__Limnochordia;f__Limnochordia;g__Hydrogenispora;s__uncultured_Clostridia | 0 |
| d__Bacteria;p__Firmicutes;c__Bacilli;o__Bacillales;f__Bacillaceae;g__Amphibacillus;__ | 0 |
| d__Bacteria;p__Actinobacteriota;c__Acidimicrobiia;o__Microtrichales;f__uncultured;g__uncultured;__ | 0 |
| d__Bacteria;p__Firmicutes;c__Bacilli;o__Bacillales;f__Planococcaceae;g__Domibacillus;__ | 0 |
| d__Bacteria;p__Firmicutes;c__Clostridia;o__Lachnospirales;f__Lachnospiraceae;g__Blautia;__ | 0 |
| d__Bacteria;p__Cyanobacteria;c__Vampirivibrionia;o__Vampirovibrionales;f__Vampirovibrionales;g__Vampirovibrionales;s__uncultured_bacterium | 0 |
| d__Bacteria;p__Firmicutes;c__Clostridia;o__Clostridiales;f__Clostridiaceae;g__Clostridium_sensu_stricto_18;__ | 0 |
| d__Bacteria;p__Firmicutes;c__Bacilli;o__Bacillales;f__Bacillaceae;g__Halobacillus;s__Halobacillus_halophilus | 0 |
| d__Bacteria;p__Bdellovibrionota;c__Oligoflexia;o__Oligoflexales;f__Oligoflexaceae;g__uncultured;s__uncultured_bacterium | 0 |
| d__Bacteria;p__Myxococcota;c__Polyangia;o__Polyangiales;f__Polyangiaceae;g__Pajaroellobacter;__ | 0 |
| d__Bacteria;p__Firmicutes;c__Bacilli;o__Alicyclobacillales;f__Alicyclobacillaceae;g__Alicyclobacillus;s__Alicyclobacillus_herbarius | 0 |
| d__Bacteria;p__Myxococcota;c__Polyangia;o__Polyangiales;f__Polyangiaceae;g__Sorangium;s__Sorangium_cellulosum | 0 |
| d__Bacteria;p__Proteobacteria;c__Alphaproteobacteria;o__Sphingomonadales;f__Sphingomonadaceae;g__Sphingopyxis;s__Sphingopyxis_terrae | 0 |
| d__Bacteria;p__Proteobacteria;c__Gammaproteobacteria;o__Beggiatoales;f__Beggiatoaceae;g__uncultured;__ | 0 |
| d__Bacteria;p__Firmicutes;c__Clostridia;o__Oscillospirales;f__Oscillospiraceae;g__NK4A214_group;s__uncultured_bacterium | 0 |
| d__Bacteria;p__Proteobacteria;c__Alphaproteobacteria;o__Zavarziniales;f__uncultured;g__uncultured;s__metagenome | 0 |
| d__Bacteria;p__Firmicutes;c__Bacilli;o__Paenibacillales;f__Paenibacillaceae;g__Paenibacillus;s__Paenibacillus_naphthalenovorans | 0 |
| d__Bacteria;p__Firmicutes;c__Clostridia;o__Oscillospirales;f__Ethanoligenenaceae;g__Incertae_Sedis;__ | 0 |
| d__Bacteria;p__Planctomycetota;c__Planctomycetes;o__Isosphaerales;f__Isosphaeraceae;g__Singulisphaera;s__uncultured_bacterium | 0 |
| d__Bacteria;p__Cyanobacteria;c__Vampirivibrionia;o__Vampirovibrionales;f__Vampirovibrionaceae;g__Vampirovibrionaceae;__ | 0 |
| d__Bacteria;p__Firmicutes;c__Clostridia;o__Oscillospirales;f__Oscillospiraceae;g__UCG-005;s__uncultured_rumen | 0 |
| d__Bacteria;p__Proteobacteria;c__Alphaproteobacteria;o__Rickettsiales;f__Mitochondria;g__Mitochondria;__ | 0 |
| d__Bacteria;p__Firmicutes;c__Bacilli;o__Bacillales;f__Bacillaceae;g__Bacillus;s__Bacillus_cohnii | 0 |
| d__Bacteria;p__Acidobacteriota;c__Acidobacteriae;o__Acidobacteriales;f__Koribacteraceae;g__Candidatus_Koribacter;s__uncultured_bacterium | 0 |
| d__Bacteria;p__Chloroflexi;c__Gitt-GS-136;o__Gitt-GS-136;f__Gitt-GS-136;g__Gitt-GS-136;s__uncultured_bacterium | 0 |
| d__Bacteria;p__Firmicutes;c__Bacilli;o__Paenibacillales;f__Paenibacillaceae;g__Ammoniphilus;__ | 0 |
| d__Bacteria;p__Firmicutes;c__Clostridia;o__Clostridiales;f__Clostridiaceae;g__Clostridium_sensu_stricto_1;s__uncultured_Clostridium | 0 |
| d__Bacteria;p__Proteobacteria;c__Alphaproteobacteria;o__Rhizobiales;f__Rhizobiales_Incertae_Sedis;g__uncultured;s__uncultured_bacterium | 0 |
| d__Bacteria;p__Firmicutes;c__Limnochordia;o__Limnochordales;f__uncultured;g__uncultured;__ | 0 |
| d__Bacteria;p__Proteobacteria;c__Alphaproteobacteria;o__Rhizobiales;f__Beijerinckiaceae;g__Methylobacterium-Methylorubrum;s__Methylorubrum_extorquens | 0 |
| d__Bacteria;p__Proteobacteria;c__Gammaproteobacteria;o__Burkholderiales;f__Oxalobacteraceae;g__Massilia;s__Massilia_timonae | 0 |
| d__Bacteria;p__Proteobacteria;c__Alphaproteobacteria;o__Rhizobiales;f__Rhizobiaceae;g__Mesorhizobium;s__metagenome | 0 |
| d__Bacteria;p__Firmicutes;c__Bacilli;o__Bacillales;f__Bacillaceae;g__Bacillus;s__Bacillus_thermoamylovorans | 0 |
| d__Bacteria;p__Firmicutes;c__Clostridia;o__Lachnospirales;f__Lachnospiraceae;g__Lachnospiraceae_UCG-007;__ | 0 |
| d__Bacteria;p__Proteobacteria;c__Alphaproteobacteria;o__Defluviicoccales;f__Defluviicoccaceae;g__Defluviicoccus;s__uncultured_bacterium | 0 |
| d__Bacteria;p__Firmicutes;c__Bacilli;o__Lactobacillales;f__Lactobacillaceae;g__Lactobacillus;s__Lactobacillus_johnsonii | 0 |
| d__Bacteria;p__Verrucomicrobiota;c__Verrucomicrobiae;o__Opitutales;f__Opitutaceae;g__IMCC26134;__ | 0 |
| d__Bacteria;p__Proteobacteria;c__Gammaproteobacteria;o__Burkholderiales;f__Comamonadaceae;g__Rhizobacter;__ | 0 |
| d__Bacteria;p__Chloroflexi;c__TK10;o__TK10;f__TK10;g__TK10;__ | 0 |
| d__Bacteria;p__Proteobacteria;c__Gammaproteobacteria;o__Burkholderiales;f__Rhodocyclaceae;g__Thauera;s__uncultured_bacterium | 0 |
| d__Bacteria;p__Bacteroidota;c__Bacteroidia;o__Bacteroidales;f__SB-5;g__SB-5;s__uncultured_bacterium | 0 |
| d__Bacteria;p__Proteobacteria;c__Gammaproteobacteria;o__Burkholderiales;f__Gallionellaceae;__;__ | 0 |
| d__Bacteria;p__Acidobacteriota;c__Vicinamibacteria;o__Vicinamibacterales;f__Vicinamibacteraceae;g__Vicinamibacteraceae;s__uncultured_Acidobacteria | 0 |
| d__Bacteria;p__Proteobacteria;c__Gammaproteobacteria;o__Diplorickettsiales;f__Diplorickettsiaceae;g__uncultured;s__uncultured_bacterium | 0 |
| d__Bacteria;p__Proteobacteria;c__Gammaproteobacteria;o__Burkholderiales;f__Comamonadaceae;g__Hydrogenophaga;s__uncultured_bacterium | 0 |
| d__Bacteria;p__Acidobacteriota;c__Holophagae;o__Holophagales;f__Holophagaceae;__;__ | 0 |
| d__Bacteria;p__Proteobacteria;c__Gammaproteobacteria;o__Xanthomonadales;f__Xanthomonadaceae;g__Pseudoxanthomonas;s__Pseudoxanthomonas_sp. | 0 |
| d__Bacteria;p__Planctomycetota;c__Phycisphaerae;o__Phycisphaerales;f__Phycisphaeraceae;g__SM1A02;s__uncultured_bacterium | 0 |
| d__Bacteria;p__Actinobacteriota;c__Actinobacteria;o__Frankiales;f__Frankiaceae;g__Frankia;s__Frankia_sp. | 0 |
| d__Bacteria;p__Proteobacteria;c__Alphaproteobacteria;o__Holosporales;f__Holosporaceae;g__uncultured;__ | 0 |
| d__Bacteria;p__Fusobacteriota;c__Fusobacteriia;o__Fusobacteriales;f__Fusobacteriaceae;g__Fusobacterium;s__Fusobacterium_nucleatum | 0 |
| d__Bacteria;p__Proteobacteria;c__Gammaproteobacteria;o__JG36-TzT-191;f__JG36-TzT-191;g__JG36-TzT-191;s__uncultured_bacterium | 0 |
| d__Bacteria;p__Actinobacteriota;c__Actinobacteria;o__Micrococcales;f__Cellulomonadaceae;g__Cellulomonas;__ | 0 |
| d__Bacteria;p__Firmicutes;c__Clostridia;o__Peptostreptococcales-Tissierellales;f__Peptostreptococcaceae;__;__ | 0 |
| d__Bacteria;p__Actinobacteriota;c__Actinobacteria;o__PeM15;f__PeM15;g__PeM15;s__metagenome | 0 |
| d__Bacteria;p__Verrucomicrobiota;c__Verrucomicrobiae;o__Verrucomicrobiales;f__Verrucomicrobiaceae;g__Roseimicrobium;__ | 0 |
| d__Bacteria;p__Proteobacteria;c__Gammaproteobacteria;o__Xanthomonadales;f__Rhodanobacteraceae;g__Ahniella;s__uncultured_bacterium | 0 |
| d__Bacteria;p__Firmicutes;c__Clostridia;o__Clostridiales;f__Caloramatoraceae;g__Fonticella;s__uncultured_bacterium | 0 |
| d__Bacteria;p__Planctomycetota;c__Phycisphaerae;o__MSBL9;f__4572-13;g__4572-13;s__uncultured_bacterium | 0 |
| d__Bacteria;p__Proteobacteria;c__Gammaproteobacteria;o__Acidiferrobacterales;f__Acidiferrobacteraceae;__;__ | 0 |
| d__Bacteria;p__Actinobacteriota;c__Acidimicrobiia;o__Microtrichales;f__Iamiaceae;g__Iamia;s__uncultured_bacterium | 0 |
| d__Bacteria;p__Proteobacteria;c__Gammaproteobacteria;o__Burkholderiales;f__Methylophilaceae;g__Methylobacillus;__ | 0 |
| d__Bacteria;p__Actinobacteriota;c__Actinobacteria;o__Corynebacteriales;f__Mycobacteriaceae;g__Mycobacterium;s__Mycobacterium_intracellulare | 0 |
| d__Bacteria;p__Actinobacteriota;c__Acidimicrobiia;o__Microtrichales;f__Ilumatobacteraceae;g__Ilumatobacter;s__uncultured_bacterium | 0 |
| d__Bacteria;p__Proteobacteria;c__Gammaproteobacteria;o__Burkholderiales;f__Nitrosomonadaceae;g__uncultured;s__metagenome | 0 |
| d__Bacteria;p__Firmicutes;c__Clostridia;o__Peptostreptococcales-Tissierellales;f__Peptostreptococcales-Tissierellales;g__Alkaliphilus;s__uncultured_bacterium | 0 |
| d__Bacteria;p__Proteobacteria;c__Gammaproteobacteria;o__CCD24;f__CCD24;g__CCD24;s__uncultured_bacterium | 0 |
| d__Bacteria;p__Bacteroidota;c__Bacteroidia;o__Sphingobacteriales;f__KD3-93;g__KD3-93;__ | 0 |
| d__Bacteria;p__Proteobacteria;c__Gammaproteobacteria;o__Xanthomonadales;f__Rhodanobacteraceae;g__Pseudofulvimonas;s__uncultured_bacterium | 0 |
| d__Bacteria;p__Bacteroidota;c__Bacteroidia;o__Chitinophagales;f__Chitinophagaceae;g__Chitinophaga;__ | 0 |
| d__Bacteria;p__Myxococcota;c__Polyangia;o__mle1-27;f__mle1-27;g__mle1-27;__ | 0 |
| d__Bacteria;p__Bacteroidota;c__Bacteroidia;o__Bacteroidales;f__Prevotellaceae;g__Prevotella_7;s__Prevotella_denticola | 0 |
| d__Bacteria;p__Proteobacteria;c__Gammaproteobacteria;o__Burkholderiales;f__Sutterellaceae;g__uncultured;s__uncultured_bacterium | 0 |
| d__Bacteria;p__Proteobacteria;c__Alphaproteobacteria;o__Holosporales;f__Holosporaceae;g__uncultured;s__uncultured_bacterium | 0 |
| d__Bacteria;p__Patescibacteria;c__Microgenomatia;o__Candidatus_Roizmanbacteria;f__Candidatus_Roizmanbacteria;g__Candidatus_Roizmanbacteria;s__uncultured_organism | 0 |
| d__Bacteria;p__Firmicutes;c__Bacilli;o__Lactobacillales;f__Lactobacillaceae;g__Lactobacillus;s__Lactobacillus_helveticus | 0 |
| d__Bacteria;p__Planctomycetota;c__Planctomycetes;o__Planctomycetales;f__Schlesneriaceae;g__Schlesneria;s__uncultured_bacterium | 0 |
| d__Bacteria;p__Bacteroidota;c__Bacteroidia;o__Cytophagales;f__Microscillaceae;g__Chryseolinea;__ | 0 |
| d__Bacteria;p__Planctomycetota;c__Planctomycetes;o__Pirellulales;f__Pirellulaceae;g__Pirellula;__ | 0 |
| d__Bacteria;p__Actinobacteriota;c__Acidimicrobiia;o__IMCC26256;f__IMCC26256;g__IMCC26256;__ | 0 |
| d__Bacteria;p__Chloroflexi;c__KD4-96;o__KD4-96;f__KD4-96;g__KD4-96;s__uncultured_bacterium | 0 |
| d__Bacteria;p__Acidobacteriota;c__Vicinamibacteria;o__Vicinamibacterales;f__Vicinamibacteraceae;g__Vicinamibacteraceae;s__uncultured_bacterium | 0 |
| d__Bacteria;p__Firmicutes;c__Bacilli;o__Lactobacillales;f__Streptococcaceae;g__Streptococcus;s__Streptococcus_sanguinis | 0 |
| d__Bacteria;p__Actinobacteriota;c__Actinobacteria;o__Frankiales;f__Sporichthyaceae;__;__ | 0 |
| d__Bacteria;p__Proteobacteria;c__Alphaproteobacteria;o__Rhodobacterales;f__Rhodobacteraceae;g__uncultured;__ | 0 |
| d__Bacteria;p__Myxococcota;c__Polyangia;o__Polyangiales;f__Phaselicystidaceae;g__Phaselicystis;__ | 0 |
| d__Bacteria;p__Proteobacteria;c__Alphaproteobacteria;o__Rhizobiales;f__Beijerinckiaceae;g__Methylosinus;__ | 0 |
| d__Bacteria;p__Proteobacteria;c__Gammaproteobacteria;o__Burkholderiales;f__Comamonadaceae;g__Rhizobacter;s__uncultured_bacterium | 0 |
| d__Bacteria;p__Bacteroidota;c__Bacteroidia;o__Cytophagales;f__Microscillaceae;g__Chryseolinea;s__uncultured_bacterium | 0 |
| d__Bacteria;p__Proteobacteria;c__Gammaproteobacteria;o__EPR3968-O8a-Bc78;f__EPR3968-O8a-Bc78;g__EPR3968-O8a-Bc78;__ | 0 |
| d__Bacteria;p__Proteobacteria;c__Gammaproteobacteria;o__Pseudomonadales;f__Moraxellaceae;g__Acinetobacter;s__Acinetobacter_radioresistens | 0 |
| d__Bacteria;p__Proteobacteria;c__Alphaproteobacteria;o__Acetobacterales;f__Acetobacteraceae;g__Rhodovastum;__ | 0 |
| d__Bacteria;p__Verrucomicrobiota;c__Verrucomicrobiae;o__Verrucomicrobiales;f__Akkermansiaceae;g__Akkermansia;__ | 0 |
| d__Bacteria;p__Bacteroidota;c__Bacteroidia;o__Cytophagales;f__Spirosomaceae;g__Emticicia;__ | 0 |
| d__Bacteria;p__Firmicutes;c__Bacilli;o__Bacillales;f__Bacillaceae;g__Anoxybacillus;s__Anoxybacillus_sp. | 0 |
| d__Bacteria;p__Acidobacteriota;c__Holophagae;o__Holophagales;f__Holophagaceae;g__Geothrix;__ | 0 |
| d__Bacteria;p__Proteobacteria;c__Alphaproteobacteria;o__Rhizobiales;f__Beijerinckiaceae;g__Methylocystis;__ | 0 |
| d__Bacteria;p__Myxococcota;c__Myxococcia;o__Myxococcales;f__Anaeromyxobacteraceae;g__Anaeromyxobacter;s__uncultured_bacterium | 0 |
| d__Bacteria;p__Bacteroidota;c__Bacteroidia;o__Cytophagales;f__Microscillaceae;g__Ohtaekwangia;s__uncultured_bacterium | 0 |
| d__Bacteria;p__Proteobacteria;c__Alphaproteobacteria;o__Rhizobiales;f__Xanthobacteraceae;g__Pseudorhodoplanes;__ | 0 |
| d__Bacteria;p__Proteobacteria;c__Alphaproteobacteria;o__Thalassobaculales;f__Thalassobaculaceae;g__Thalassobaculum;__ | 0 |
| d__Bacteria;p__Planctomycetota;c__Phycisphaerae;o__Phycisphaerales;f__Phycisphaeraceae;g__SM1A02;__ | 0 |
| d__Bacteria;p__Planctomycetota;c__Phycisphaerae;o__CCM11a;f__CCM11a;g__CCM11a;s__uncultured_bacterium | 0 |
| d__Bacteria;p__Proteobacteria;c__Gammaproteobacteria;o__Salinisphaerales;f__Solimonadaceae;g__Polycyclovorans;__ | 0 |
| d__Bacteria;p__Proteobacteria;c__Alphaproteobacteria;o__Rhodobacterales;f__Rhodobacteraceae;g__Defluviimonas;__ | 0 |
| d__Bacteria;p__Proteobacteria;c__Gammaproteobacteria;o__Steroidobacterales;f__Steroidobacteraceae;g__Steroidobacter;__ | 0 |
| d__Bacteria;p__Actinobacteriota;c__Actinobacteria;o__Micrococcales;f__Microbacteriaceae;g__Lysinimonas;__ | 0 |
| d__Bacteria;p__Proteobacteria;c__Gammaproteobacteria;o__Burkholderiales;f__Comamonadaceae;g__Curvibacter;s__uncultured_bacterium | 0 |
| d__Bacteria;p__Proteobacteria;c__Alphaproteobacteria;o__Rhizobiales;f__Rhizobiales_Incertae_Sedis;g__Bauldia;__ | 0 |
| d__Bacteria;p__Proteobacteria;c__Gammaproteobacteria;o__Xanthomonadales;f__Xanthomonadaceae;g__Vulcaniibacterium;__ | 0 |
| d__Bacteria;p__Proteobacteria;c__Gammaproteobacteria;o__Burkholderiales;f__Rhodocyclaceae;g__Thauera;__ | 0 |
| d__Bacteria;p__Proteobacteria;c__Alphaproteobacteria;o__Rhizobiales;f__Methyloligellaceae;g__uncultured;s__uncultured_bacterium | 0 |
| d__Bacteria;p__Patescibacteria;c__Saccharimonadia;o__Saccharimonadales;f__Saccharimonadales;g__Saccharimonadales;s__uncultured_soil | 0 |
| d__Bacteria;p__Desulfobacterota;c__Syntrophia;o__Syntrophales;f__Smithellaceae;g__Smithella;__ | 0 |
| d__Bacteria;p__Actinobacteriota;c__Actinobacteria;o__Streptosporangiales;f__Streptosporangiaceae;__;__ | 0 |
| d__Bacteria;p__Actinobacteriota;c__Acidimicrobiia;o__Microtrichales;f__Microtrichaceae;g__Sva0996_marine_group;__ | 0 |
| d__Bacteria;p__Proteobacteria;c__Gammaproteobacteria;o__Steroidobacterales;f__Steroidobacteraceae;g__Steroidobacter;s__uncultured_bacterium | 0 |
| d__Bacteria;p__Proteobacteria;c__Gammaproteobacteria;o__Pseudomonadales;f__Moraxellaceae;__;__ | 0 |
| d__Bacteria;p__Actinobacteriota;c__Actinobacteria;o__Frankiales;f__Frankiaceae;g__Frankia;__ | 0 |
| d__Bacteria;p__Bacteroidota;c__Bacteroidia;o__Chitinophagales;f__Chitinophagaceae;g__Pseudoflavitalea;__ | 0 |
| d__Bacteria;p__Proteobacteria;c__Gammaproteobacteria;o__Burkholderiales;f__Rhodocyclaceae;__;__ | 0 |
| d__Bacteria;p__Spirochaetota;c__Spirochaetia;o__Spirochaetales;f__Spirochaetaceae;g__Spirochaeta;s__uncultured_bacterium | 0 |
| d__Bacteria;p__Proteobacteria;c__Gammaproteobacteria;o__Burkholderiales;f__Comamonadaceae;g__Acidovorax;__ | 0 |
| d__Bacteria;p__Actinobacteriota;c__Actinobacteria;o__Frankiales;f__Nakamurellaceae;g__Nakamurella;s__uncultured_bacterium | 0 |
| d__Bacteria;p__Proteobacteria;c__Gammaproteobacteria;o__Xanthomonadales;f__Xanthomonadaceae;g__Pseudoxanthomonas;s__Pseudoxanthomonas_japonensis | 0 |
| d__Bacteria;p__Actinobacteriota;c__Acidimicrobiia;o__Microtrichales;f__Microtrichaceae;g__uncultured;__ | 0 |
| d__Bacteria;p__Firmicutes;c__Clostridia;o__Oscillospirales;f__Hungateiclostridiaceae;g__Anaerobacterium;__ | 0 |
| d__Bacteria;p__Proteobacteria;c__Alphaproteobacteria;o__Rhodospirillales;f__Magnetospiraceae;g__uncultured;s__metagenome | 0 |
| d__Bacteria;p__Proteobacteria;c__Alphaproteobacteria;o__Rhizobiales;f__Rhizobiaceae;g__Pseudaminobacter;s__Mesorhizobium_sp. | 0 |
| d__Bacteria;p__Proteobacteria;c__Gammaproteobacteria;o__Enterobacterales;f__Erwiniaceae;g__Pantoea;s__Pantoea_vagans | 0 |
| d__Bacteria;p__Actinobacteriota;c__Actinobacteria;o__Pseudonocardiales;f__Pseudonocardiaceae;g__Pseudonocardia;s__uncultured_bacterium | 0 |
| d__Bacteria;p__Chloroflexi;c__Dehalococcoidia;o__S085;f__S085;g__S085;s__uncultured_bacterium | 0 |
| d__Bacteria;p__Dependentiae;c__Babeliae;o__Babeliales;f__Babeliales;g__Babeliales;s__uncultured_bacterium | 0 |
| d__Bacteria;p__Actinobacteriota;c__Actinobacteria;o__Micrococcales;f__Microbacteriaceae;g__Agromyces;__ | 0 |
| d__Bacteria;p__Proteobacteria;c__Gammaproteobacteria;o__Enterobacterales;f__Erwiniaceae;g__Pantoea;s__Pantoea_agglomerans | 0 |
| d__Bacteria;p__Proteobacteria;c__Gammaproteobacteria;o__Burkholderiales;f__Comamonadaceae;g__Rubrivivax;__ | 0 |
| d__Bacteria;p__Actinobacteriota;c__Actinobacteria;o__Propionibacteriales;f__Nocardioidaceae;g__Aeromicrobium;__ | 0 |
| d__Bacteria;p__Firmicutes;c__Clostridia;o__Oscillospirales;f__Oscillospiraceae;g__Oscillibacter;__ | 0 |
| d__Bacteria;p__Proteobacteria;c__Gammaproteobacteria;o__Burkholderiales;f__Rhodocyclaceae;g__Dechloromonas;s__uncultured_bacterium | 0 |
| d__Bacteria;p__Actinobacteriota;c__Actinobacteria;o__Pseudonocardiales;f__Pseudonocardiaceae;g__Crossiella;s__uncultured_bacterium | 0 |
| d__Bacteria;p__Proteobacteria;c__Gammaproteobacteria;o__Enterobacterales;f__Erwiniaceae;g__Pantoea;__ | 0 |
| d__Bacteria;p__Proteobacteria;c__Alphaproteobacteria;o__uncultured;f__uncultured;g__uncultured;s__metagenome | 0 |
| d__Bacteria;p__Proteobacteria;c__Gammaproteobacteria;o__Pseudomonadales;f__Pseudomonadaceae;g__Pseudomonas;s__Pseudomonas_aeruginosa | 0 |
| d__Bacteria;p__Actinobacteriota;c__Actinobacteria;o__Streptosporangiales;f__Thermomonosporaceae;g__Actinomadura;__ | 0 |
| d__Bacteria;p__Proteobacteria;c__Alphaproteobacteria;o__Reyranellales;f__Reyranellaceae;g__Reyranella;s__uncultured_bacterium | 0 |
| d__Bacteria;p__Chloroflexi;c__Anaerolineae;o__Ardenticatenales;f__uncultured;g__uncultured;s__uncultured_bacterium | 0 |
| d__Bacteria;p__Proteobacteria;c__Gammaproteobacteria;o__Burkholderiales;f__Methylophilaceae;g__UBA6140;s__uncultured_bacterium | 0 |
| d__Bacteria;p__Firmicutes;c__Bacilli;o__Bacillales;f__Bacillaceae;g__Bacillus;s__uncultured_compost | 0 |
| d__Bacteria;p__Proteobacteria;c__Alphaproteobacteria;o__Rhizobiales;f__Rhizobiales_Incertae_Sedis;g__Nordella;s__uncultured_bacterium | 0 |
| d__Bacteria;p__Cyanobacteria;c__Vampirivibrionia;o__Vampirovibrionales;f__Vampirovibrionaceae;g__Vampirovibrionaceae;s__uncultured_bacterium | 0 |
| d__Bacteria;p__Acidobacteriota;c__Acidobacteriae;o__Solibacterales;f__Solibacteraceae;g__Candidatus_Solibacter;s__uncultured_bacterium | 0 |
| d__Bacteria;p__Proteobacteria;c__Alphaproteobacteria;o__Rhizobiales;f__Xanthobacteraceae;g__Nitrobacter;__ | 0 |
| d__Bacteria;p__Firmicutes;c__Bacilli;o__Bacillales;f__Bacillaceae;g__Bacillus;s__Bacillus_licheniformis | 0 |
| d__Bacteria;p__Proteobacteria;c__Alphaproteobacteria;o__Rhizobiales;f__Xanthobacteraceae;g__Rhodoplanes;s__uncultured_bacterium | 0 |
| d__Bacteria;p__Actinobacteriota;c__Actinobacteria;o__Corynebacteriales;f__Nocardiaceae;g__Gordonia;__ | 0 |
| d__Bacteria;p__Proteobacteria;c__Gammaproteobacteria;o__Burkholderiales;f__Comamonadaceae;g__Schlegelella;__ | 0 |
| d__Bacteria;p__Verrucomicrobiota;c__Verrucomicrobiae;o__Pedosphaerales;f__Pedosphaeraceae;g__Pedosphaeraceae;__ | 0 |
| d__Bacteria;p__Myxococcota;c__Polyangia;o__Polyangiales;f__Sandaracinaceae;g__Sandaracinus;__ | 0 |
| d__Bacteria;p__Proteobacteria;c__Gammaproteobacteria;o__R7C24;f__R7C24;g__R7C24;__ | 0 |
| d__Bacteria;p__Bacteroidota;c__Bacteroidia;o__Cytophagales;f__Spirosomaceae;g__Leadbetterella;__ | 0 |
| d__Bacteria;p__Desulfobacterota;c__uncultured;o__uncultured;f__uncultured;g__uncultured;__ | 0 |
| d__Bacteria;p__Bacteroidota;c__Bacteroidia;o__Sphingobacteriales;f__Sphingobacteriaceae;g__Pseudopedobacter;__ | 0 |
| d__Bacteria;p__Firmicutes;c__Bacilli;o__Bacillales;f__Planococcaceae;g__Jeotgalibacillus;__ | 0 |
| d__Bacteria;p__Proteobacteria;c__Gammaproteobacteria;o__PLTA13;f__PLTA13;g__PLTA13;__ | 0 |
| d__Bacteria;p__Proteobacteria;c__Alphaproteobacteria;o__Rhizobiales;f__Xanthobacteraceae;g__Starkeya;__ | 0 |
| d__Bacteria;p__Actinobacteriota;c__Actinobacteria;o__Streptomycetales;f__Streptomycetaceae;g__Streptomyces;s__Streptomyces_griseoplanus | 0 |
| d__Bacteria;__;__;__;__;__;__ | 0 |
| d__Bacteria;p__Proteobacteria;c__Alphaproteobacteria;o__Acetobacterales;f__Acetobacteraceae;g__uncultured;s__uncultured_bacterium | 0 |
| d__Bacteria;p__Proteobacteria;c__Alphaproteobacteria;o__Rickettsiales;f__Rickettsiaceae;g__Rickettsia;__ | 0 |
| d__Bacteria;p__Proteobacteria;c__Alphaproteobacteria;o__Rhizobiales;f__Beijerinckiaceae;g__Methylorosula;s__uncultured_bacterium | 0 |
| d__Bacteria;p__Elusimicrobiota;c__Endomicrobia;o__Endomicrobiales;f__Endomicrobiaceae;g__Endomicrobium;s__uncultured_bacterium | 0 |
| d__Bacteria;p__Bacteroidota;c__Bacteroidia;o__Chitinophagales;f__Chitinophagaceae;g__Niabella;__ | 0 |
| d__Bacteria;p__Proteobacteria;c__Gammaproteobacteria;o__Xanthomonadales;f__Xanthomonadaceae;g__Vulcaniibacterium;s__uncultured_bacterium | 0 |
| d__Bacteria;p__Proteobacteria;c__Gammaproteobacteria;o__Burkholderiales;f__Methylophilaceae;g__UBA6140;__ | 0 |
| d__Bacteria;p__Actinobacteriota;c__Actinobacteria;o__Micrococcales;f__Microbacteriaceae;g__Leifsonia;s__Leifsonia_sp. | 0 |
| d__Bacteria;p__Proteobacteria;c__Gammaproteobacteria;o__Steroidobacterales;f__Steroidobacteraceae;g__uncultured;s__uncultured_bacterium | 0 |
| d__Bacteria;p__Proteobacteria;c__Alphaproteobacteria;o__Caulobacterales;f__Caulobacteraceae;g__Caulobacter;s__Caulobacter_vibrioides | 0 |
| d__Bacteria;p__Firmicutes;c__Bacilli;o__Aneurinibacillales;f__Aneurinibacillaceae;g__Aneurinibacillus;s__Aneurinibacillus_soli | 0 |
| d__Bacteria;p__Proteobacteria;c__Gammaproteobacteria;o__Salinisphaerales;f__Solimonadaceae;g__Fontimonas;__ | 0 |
| d__Bacteria;p__Actinobacteriota;c__Thermoleophilia;o__Solirubrobacterales;f__Solirubrobacteraceae;g__Parviterribacter;s__uncultured_bacterium | 0 |
| d__Bacteria;p__Proteobacteria;c__Gammaproteobacteria;o__Xanthomonadales;f__Xanthomonadaceae;g__Arenimonas;s__Arenimonas_malthae | 0 |
| d__Bacteria;p__Proteobacteria;c__Gammaproteobacteria;o__Salinisphaerales;f__Solimonadaceae;g__Panacagrimonas;__ | 0 |
| d__Bacteria;p__Proteobacteria;c__Gammaproteobacteria;o__Burkholderiales;f__Comamonadaceae;g__Xenophilus;__ | 0 |
| d__Bacteria;p__Actinobacteriota;c__Actinobacteria;o__Micrococcales;f__Microbacteriaceae;g__Microbacterium;s__Microbacterium_sp. | 0 |
| d__Bacteria;p__Proteobacteria;c__Gammaproteobacteria;o__Burkholderiales;f__Burkholderiaceae;g__Burkholderia-Caballeronia-Paraburkholderia;s__Burkholderia_sp. | 0 |
| d__Bacteria;p__Proteobacteria;c__Gammaproteobacteria;o__Burkholderiales;f__Comamonadaceae;g__uncultured;s__uncultured_bacterium | 0 |
| d__Bacteria;p__Firmicutes;c__Clostridia;o__Christensenellales;f__Christensenellaceae;g__uncultured;s__uncultured_bacterium | 0 |
| d__Bacteria;p__Firmicutes;c__Clostridia;o__Lachnospirales;f__Lachnospiraceae;g__Lachnospiraceae_UCG-003;s__uncultured_bacterium | 0 |
| d__Bacteria;p__Proteobacteria;c__Alphaproteobacteria;o__Rhizobiales;f__Beijerinckiaceae;g__Chelatococcus;__ | 0 |
| d__Bacteria;p__Proteobacteria;c__Alphaproteobacteria;o__Rhizobiales;f__Rhizobiaceae;g__Allorhizobium-Neorhizobium-Pararhizobium-Rhizobium;s__uncultured_Alphaproteobacteria | 0 |
| d__Bacteria;p__Firmicutes;c__Sulfobacillia;o__Sulfobacillales;f__Sulfobacillaceae;g__uncultured;__ | 0 |
| d__Bacteria;p__Proteobacteria;c__Gammaproteobacteria;o__Burkholderiales;f__Comamonadaceae;g__Limnohabitans;s__uncultured_bacterium | 0 |
| d__Bacteria;p__Actinobacteriota;c__Actinobacteria;o__Micromonosporales;f__Micromonosporaceae;g__Micromonospora;__ | 0 |
| d__Bacteria;p__Bacteroidota;c__Bacteroidia;o__Cytophagales;f__Spirosomaceae;__;__ | 0 |
| d__Bacteria;p__Chloroflexi;c__Ktedonobacteria;o__Ktedonobacterales;f__Ktedonobacteraceae;g__JG30a-KF-32;__ | 0 |
| d__Bacteria;p__Proteobacteria;c__Gammaproteobacteria;o__Xanthomonadales;f__Xanthomonadaceae;g__uncultured;s__uncultured_Luteimonas | 0 |
| d__Bacteria;p__Proteobacteria;c__Gammaproteobacteria;o__Salinisphaerales;f__Solimonadaceae;g__Solimonas;__ | 0 |
| d__Bacteria;p__Actinobacteriota;c__Thermoleophilia;o__Gaiellales;f__uncultured;g__uncultured;__ | 0 |
| d__Bacteria;p__Proteobacteria;c__Alphaproteobacteria;o__Elsterales;__;__;__ | 0 |
| d__Bacteria;p__Proteobacteria;c__Gammaproteobacteria;o__Burkholderiales;__;__;__ | 0 |
| d__Bacteria;p__Proteobacteria;c__Gammaproteobacteria;o__Xanthomonadales;f__Xanthomonadaceae;g__Pseudoxanthomonas;s__Pseudoxanthomonas_mexicana | 0 |
| d__Bacteria;p__Proteobacteria;c__Gammaproteobacteria;o__Burkholderiales;f__Comamonadaceae;g__Hydrogenophaga;s__Hydrogenophaga_pseudoflava | 0 |
| d__Bacteria;p__Bacteroidota;c__Bacteroidia;o__Chitinophagales;f__Chitinophagaceae;g__Sediminibacterium;__ | 0 |
| d__Bacteria;p__Proteobacteria;c__Gammaproteobacteria;o__Xanthomonadales;f__Xanthomonadaceae;g__Arenimonas;__ | 0 |
| d__Bacteria;p__Proteobacteria;c__Alphaproteobacteria;o__Sphingomonadales;f__Sphingomonadaceae;g__Sphingobium;__ | 0 |
| d__Bacteria;p__Firmicutes;c__Clostridia;o__Peptostreptococcales-Tissierellales;f__Peptostreptococcaceae;g__uncultured;__ | 0 |
| d__Bacteria;p__Actinobacteriota;c__Actinobacteria;o__Corynebacteriales;f__Nocardiaceae;g__Rhodococcus;s__Rhodococcus_rhodochrous | 0 |
| d__Bacteria;p__Proteobacteria;c__Gammaproteobacteria;o__Burkholderiales;f__Rhodocyclaceae;g__Dechloromonas;__ | 0 |
| d__Bacteria;p__Proteobacteria;c__Gammaproteobacteria;o__Burkholderiales;f__Nitrosomonadaceae;g__uncultured;__ | 0 |
| d__Bacteria;p__Actinobacteriota;c__Actinobacteria;o__Corynebacteriales;f__Nocardiaceae;g__Gordonia;s__Gordonia_rubripertincta | 0 |
| d__Bacteria;p__Proteobacteria;c__Alphaproteobacteria;o__Rhizobiales;f__Rhizobiales_Incertae_Sedis;g__Nordella;__ | 0 |
| d__Bacteria;p__Verrucomicrobiota;c__Verrucomicrobiae;o__Verrucomicrobiales;f__Rubritaleaceae;g__Luteolibacter;s__uncultured_bacterium | 0 |
| d__Bacteria;p__Proteobacteria;c__Alphaproteobacteria;o__Rhizobiales;f__Xanthobacteraceae;g__Bradyrhizobium;s__Bradyrhizobium_sp. | 0 |
| d__Bacteria;p__Proteobacteria;c__Gammaproteobacteria;o__Xanthomonadales;f__Rhodanobacteraceae;g__Rhodanobacter;s__Rhodanobacter_sp. | 0 |
| d__Bacteria;p__Actinobacteriota;c__Actinobacteria;o__Micrococcales;f__Intrasporangiaceae;g__Tetrasphaera;__ | 0 |
| d__Bacteria;p__Myxococcota;c__Myxococcia;o__Myxococcales;f__Vulgatibacteraceae;g__Vulgatibacter;s__uncultured_bacterium | 0 |
| d__Bacteria;p__Bacteroidota;c__Bacteroidia;o__Cytophagales;f__Cyclobacteriaceae;g__uncultured;s__uncultured_bacterium | 0 |
| d__Bacteria;p__Proteobacteria;c__Gammaproteobacteria;o__Burkholderiales;f__Nitrosomonadaceae;g__oc32;__ | 0 |
| d__Bacteria;p__Planctomycetota;c__Phycisphaerae;o__Tepidisphaerales;f__CPla-3_termite_group;g__CPla-3_termite_group;s__uncultured_planctomycete | 0 |
| d__Bacteria;p__Actinobacteriota;c__Actinobacteria;o__Streptomycetales;f__Streptomycetaceae;g__Streptomyces;s__Streptomyces_flavoviridis | 0 |
| d__Bacteria;p__Proteobacteria;c__Gammaproteobacteria;o__Burkholderiales;f__Methylophilaceae;g__Methylotenera;__ | 0 |
| d__Bacteria;p__Bacteroidota;c__Bacteroidia;o__Flavobacteriales;f__Flavobacteriaceae;g__Flavobacterium;s__uncultured_bacterium | 0 |
| d__Bacteria;p__Myxococcota;c__Polyangia;o__Polyangiales;f__BIrii41;g__BIrii41;s__uncultured_bacterium | 0 |
| d__Bacteria;p__Myxococcota;c__Polyangia;o__Blfdi19;f__Blfdi19;g__Blfdi19;__ | 0 |
| d__Bacteria;p__WPS-2;c__WPS-2;o__WPS-2;f__WPS-2;g__WPS-2;s__uncultured_bacterium | 0 |
| d__Bacteria;p__WS4;c__WS4;o__WS4;f__WS4;g__WS4;s__uncultured_bacterium | 0 |
| d__Bacteria;p__Actinobacteriota;c__Actinobacteria;o__Micromonosporales;f__Micromonosporaceae;g__Plantactinospora;__ | 0 |
| d__Bacteria;p__Proteobacteria;__;__;__;__;__ | 0 |
| d__Bacteria;p__Firmicutes;c__Clostridia;o__Clostridiales;f__Clostridiaceae;g__Clostridium_sensu_stricto_12;__ | 0 |
| d__Bacteria;p__Planctomycetota;c__BD7-11;o__BD7-11;f__BD7-11;g__BD7-11;s__uncultured_bacterium | 0 |
| d__Bacteria;p__Chloroflexi;c__Anaerolineae;o__Caldilineales;f__Caldilineaceae;g__uncultured;s__uncultured_bacterium | 0 |
| d__Bacteria;p__Chloroflexi;c__TK10;o__TK10;f__TK10;g__TK10;s__uncultured_bacterium | 0 |
| d__Bacteria;p__Proteobacteria;c__Gammaproteobacteria;o__Xanthomonadales;f__Xanthomonadaceae;g__Luteimonas;s__Luteimonas_sp. | 0 |
| d__Bacteria;p__Planctomycetota;c__Planctomycetes;o__Planctomycetales;f__Rubinisphaeraceae;g__SH-PL14;__ | 0 |
| d__Bacteria;p__Firmicutes;c__Clostridia;o__Peptostreptococcales-Tissierellales;f__Family_XI;g__Keratinibaculum;__ | 0 |
| d__Bacteria;p__Verrucomicrobiota;c__Verrucomicrobiae;o__Opitutales;f__Opitutaceae;g__Lacunisphaera;__ | 0 |
| d__Bacteria;p__Firmicutes;c__Clostridia;o__Monoglobales;f__Monoglobaceae;g__Monoglobus;s__uncultured_bacterium | 0 |
| d__Bacteria;p__Patescibacteria;c__Saccharimonadia;o__Saccharimonadales;f__WWH38;g__WWH38;s__uncultured_bacterium | 0 |
| d__Bacteria;p__Actinobacteriota;c__Actinobacteria;o__Pseudonocardiales;f__Pseudonocardiaceae;g__Saccharopolyspora;__ | 0 |
| d__Bacteria;p__Verrucomicrobiota;c__Verrucomicrobiae;o__Chthoniobacterales;f__Chthoniobacteraceae;g__LD29;s__uncultured_bacterium | 0 |
| d__Bacteria;p__Bacteroidota;c__Bacteroidia;o__Cytophagales;f__Spirosomaceae;g__Spirosoma;__ | 0 |
| d__Bacteria;p__Acidobacteriota;c__Acidobacteriae;o__Acidobacteriales;f__Acidobacteriaceae_(Subgroup_1);g__Granulicella;s__uncultured_bacterium | 0 |
| d__Bacteria;p__Proteobacteria;c__Gammaproteobacteria;o__Pseudomonadales;f__Moraxellaceae;g__uncultured;__ | 0 |
| d__Bacteria;p__Firmicutes;c__Bacilli;o__Bacillales;f__Planococcaceae;g__Paenisporosarcina;__ | 0 |
| d__Bacteria;p__Dadabacteria;c__Dadabacteriia;o__Dadabacteriales;f__Dadabacteriales;g__Dadabacteriales;s__uncultured_bacterium | 0 |
| d__Bacteria;p__Firmicutes;c__Syntrophomonadia;o__Syntrophomonadales;f__Syntrophomonadaceae;g__Syntrophomonas;s__uncultured_bacterium | 0 |
| d__Bacteria;p__Actinobacteriota;c__Actinobacteria;o__Corynebacteriales;f__Mycobacteriaceae;g__Mycobacterium;s__Mycobacterium_kumamotonense | 0 |
| d__Bacteria;p__Actinobacteriota;c__Actinobacteria;o__Pseudonocardiales;f__Pseudonocardiaceae;g__Actinopolyspora;__ | 0 |
| d__Bacteria;p__Actinobacteriota;c__Actinobacteria;o__Micrococcales;f__Intrasporangiaceae;g__Terrabacter;__ | 0 |
| d__Bacteria;p__Actinobacteriota;c__Actinobacteria;o__Propionibacteriales;f__Nocardioidaceae;g__Aeromicrobium;s__uncultured_bacterium | 0 |
| d__Bacteria;p__Proteobacteria;c__Gammaproteobacteria;o__Burkholderiales;f__Comamonadaceae;g__Piscinibacter;__ | 0 |
| d__Bacteria;p__Proteobacteria;c__Alphaproteobacteria;o__Azospirillales;f__Azospirillaceae;g__Nitrospirillum;__ | 0 |
| d__Bacteria;p__Proteobacteria;c__Gammaproteobacteria;o__EV818SWSAP88;f__EV818SWSAP88;g__EV818SWSAP88;s__uncultured_bacterium | 0 |
| d__Bacteria;p__Firmicutes;c__Clostridia;o__Oscillospirales;f__Ruminococcaceae;g__uncultured;s__uncultured_bacterium | 0 |
| d__Bacteria;p__Firmicutes;c__Clostridia;o__Christensenellales;f__Christensenellaceae;g__uncultured;__ | 0 |
| d__Bacteria;p__Proteobacteria;c__Alphaproteobacteria;o__Ferrovibrionales;f__Ferrovibrionales;__;__ | 0 |
| d__Bacteria;p__Proteobacteria;c__Gammaproteobacteria;o__R7C24;f__R7C24;g__R7C24;s__metagenome | 0 |
| d__Bacteria;p__Actinobacteriota;c__Actinobacteria;o__Frankiales;f__Sporichthyaceae;g__Sporichthya;__ | 0 |
| d__Bacteria;p__Verrucomicrobiota;c__Verrucomicrobiae;o__Chthoniobacterales;f__Xiphinematobacteraceae;g__Candidatus_Xiphinematobacter;__ | 0 |
| d__Bacteria;p__Proteobacteria;c__Gammaproteobacteria;o__Coxiellales;f__Coxiellaceae;g__Coxiella;s__metagenome | 0 |
| d__Bacteria;p__Proteobacteria;c__Alphaproteobacteria;o__Elsterales;f__URHD0088;g__URHD0088;__ | 0 |
| d__Bacteria;p__Firmicutes;c__Bacilli;o__Paenibacillales;f__Paenibacillaceae;g__Paenibacillus;s__Paenibacillus_xylanexedens | 0 |
| d__Bacteria;p__Proteobacteria;c__Gammaproteobacteria;o__EV818SWSAP88;f__EV818SWSAP88;g__EV818SWSAP88;s__uncultured_Ectothiorhodospira | 0 |
| d__Bacteria;p__Proteobacteria;c__Gammaproteobacteria;o__Xanthomonadales;f__Rhodanobacteraceae;g__Tahibacter;__ | 0 |
| d__Bacteria;p__Proteobacteria;c__Alphaproteobacteria;o__Micavibrionales;f__uncultured;g__uncultured;__ | 0 |
| d__Bacteria;p__Proteobacteria;c__Gammaproteobacteria;o__Xanthomonadales;f__Xanthomonadaceae;g__Pseudoxanthomonas;s__uncultured_bacterium | 0 |
| d__Bacteria;p__Proteobacteria;c__Gammaproteobacteria;o__Xanthomonadales;f__Xanthomonadaceae;g__Stenotrophomonas;s__uncultured_Pseudomonas | 0 |
| d__Bacteria;p__Firmicutes;c__Desulfotomaculia;o__Ammonifexales;f__Desulforudaceae;g__Candidatus_Desulforudis;s__uncultured_bacterium | 0 |
| d__Bacteria;p__Proteobacteria;c__Gammaproteobacteria;o__Salinisphaerales;f__Solimonadaceae;g__uncultured;s__uncultured_bacterium | 0 |
| d__Bacteria;p__Desulfobacterota;c__Desulfuromonadia;o__Bradymonadales;f__Bradymonadales;g__Bradymonadales;__ | 0 |
| d__Bacteria;p__Bacteroidota;c__Bacteroidia;o__Chitinophagales;f__Saprospiraceae;g__uncultured;s__uncultured_bacterium | 0 |
| d__Bacteria;p__Proteobacteria;c__Alphaproteobacteria;o__Rhizobiales;f__Hyphomicrobiaceae;__;__ | 0 |
| d__Bacteria;p__Proteobacteria;c__Alphaproteobacteria;o__Rhizobiales;f__Xanthobacteraceae;g__Bradyrhizobium;s__uncultured_bacterium | 0 |
| d__Bacteria;p__Planctomycetota;c__Planctomycetes;o__Isosphaerales;f__Isosphaeraceae;g__Isosphaera;s__uncultured_bacterium | 0 |
| d__Bacteria;p__Proteobacteria;c__Gammaproteobacteria;o__Xanthomonadales;f__Rhodanobacteraceae;g__Chujaibacter;s__Chujaibacter_soli | 0 |
| d__Bacteria;p__Bdellovibrionota;c__Bdellovibrionia;o__Bdellovibrionales;f__Bdellovibrionaceae;g__OM27_clade;s__metagenome | 0 |
| d__Bacteria;p__Chloroflexi;c__Dehalococcoidia;o__S085;f__S085;g__S085;s__metagenome | 0 |
| d__Bacteria;p__Verrucomicrobiota;c__Verrucomicrobiae;o__Opitutales;f__Opitutaceae;g__Opitutus;s__metagenome | 0 |
| d__Bacteria;p__Proteobacteria;c__Gammaproteobacteria;o__Burkholderiales;f__SC-I-84;g__SC-I-84;__ | 0 |
| d__Bacteria;p__Armatimonadota;c__Fimbriimonadia;o__Fimbriimonadales;f__Fimbriimonadaceae;__;__ | 0 |
| d__Bacteria;p__Proteobacteria;c__Gammaproteobacteria;o__Burkholderiales;f__Burkholderiaceae;g__Pandoraea;__ | 0 |
| d__Bacteria;p__Firmicutes;c__uncultured;o__uncultured;f__uncultured;g__uncultured;s__uncultured_bacterium | 0 |
| d__Bacteria;p__Proteobacteria;c__Alphaproteobacteria;o__Caulobacterales;f__Caulobacteraceae;g__Brevundimonas;s__Brevundimonas_bullata | 0 |
| d__Bacteria;p__Chloroflexi;c__Anaerolineae;o__SBR1031;f__A4b;g__A4b;s__uncultured_bacterium | 0 |
| d__Bacteria;p__Spirochaetota;c__Spirochaetia;o__Spirochaetales;f__Spirochaetaceae;g__Spirochaeta_2;__ | 0 |
| d__Bacteria;p__Verrucomicrobiota;c__Verrucomicrobiae;o__Verrucomicrobiales;f__Verrucomicrobiaceae;g__Prosthecobacter;s__uncultured_bacterium | 0 |
| d__Bacteria;p__Proteobacteria;c__Gammaproteobacteria;o__Burkholderiales;f__Burkholderiaceae;g__Limnobacter;s__uncultured_bacterium | 0 |
| d__Bacteria;p__Myxococcota;c__Polyangia;o__Polyangiales;f__Polyangiaceae;g__Pajaroellobacter;s__metagenome | 0 |
| d__Bacteria;p__Verrucomicrobiota;c__Verrucomicrobiae;o__Chthoniobacterales;f__Chthoniobacteraceae;g__Candidatus_Udaeobacter;__ | 0 |
| d__Bacteria;p__Proteobacteria;c__Alphaproteobacteria;o__Rhizobiales;f__Amb-16S-1323;g__Amb-16S-1323;s__uncultured_bacterium | 0 |
| d__Bacteria;p__Proteobacteria;c__Gammaproteobacteria;o__Burkholderiales;f__Rhodocyclaceae;g__Azoarcus;s__Azoarcus_sp. | 0 |
| d__Bacteria;p__Proteobacteria;c__Gammaproteobacteria;o__Salinisphaerales;f__Solimonadaceae;g__Nevskia;__ | 0 |
| d__Bacteria;p__Firmicutes;c__Clostridia;o__Clostridiales;f__Clostridiaceae;g__Clostridium_sensu_stricto_8;__ | 0 |
| d__Bacteria;p__Planctomycetota;c__Planctomycetes;o__Gemmatales;f__Gemmataceae;__;__ | 0 |
| d__Bacteria;p__Acidobacteriota;c__Subgroup_5;o__Subgroup_5;f__Subgroup_5;g__Subgroup_5;__ | 0 |
| d__Bacteria;p__Proteobacteria;c__Gammaproteobacteria;o__Xanthomonadales;f__Xanthomonadaceae;g__Pseudoxanthomonas;s__Pseudoxanthomonas_indica | 0 |
| d__Bacteria;p__Proteobacteria;c__Alphaproteobacteria;o__Rickettsiales;f__AB1;g__AB1;__ | 0 |
| d__Bacteria;p__Proteobacteria;c__Alphaproteobacteria;o__Caulobacterales;f__Hyphomonadaceae;__;__ | 0 |
| d__Bacteria;p__Sumerlaeota;c__Sumerlaeia;o__Sumerlaeales;f__Sumerlaeaceae;g__Sumerlaea;__ | 0 |
| d__Bacteria;p__Actinobacteriota;c__Actinobacteria;o__Pseudonocardiales;f__Pseudonocardiaceae;g__Amycolatopsis;s__Amycolatopsis_sp. | 0 |
| d__Bacteria;p__Firmicutes;c__Clostridia;o__Clostridiales;f__Caloramatoraceae;g__Caloramator;s__uncultured_bacterium | 0 |
| d__Bacteria;p__Proteobacteria;c__Gammaproteobacteria;o__Xanthomonadales;__;__;__ | 0 |
| d__Bacteria;p__Actinobacteriota;c__Actinobacteria;o__Catenulisporales;f__Actinospicaceae;g__Actinospica;s__Actinospica_acidiphila | 0 |
| d__Bacteria;p__Proteobacteria;c__Gammaproteobacteria;o__Burkholderiales;f__Oxalobacteraceae;g__Massilia;s__Massilia_armeniaca | 0 |
| d__Bacteria;p__Firmicutes;c__Clostridia;o__Peptococcales;f__Peptococcaceae;g__uncultured;s__uncultured_bacterium | 0 |
| d__Bacteria;p__Bdellovibrionota;c__Bdellovibrionia;o__Bdellovibrionales;f__Bdellovibrionaceae;g__OM27_clade;__ | 0 |
| d__Bacteria;p__Proteobacteria;c__Alphaproteobacteria;o__Sphingomonadales;f__Sphingomonadaceae;g__Novosphingobium;s__metagenome | 0 |
| d__Bacteria;p__Proteobacteria;c__Gammaproteobacteria;o__Enterobacterales;f__Erwiniaceae;g__Pantoea;s__Pantoea_ananatis | 0 |
| d__Bacteria;p__Firmicutes;c__Bacilli;o__Thermoactinomycetales;f__Thermoactinomycetaceae;g__uncultured;__ | 0 |
| d__Bacteria;p__Actinobacteriota;c__Actinobacteria;o__Micrococcales;f__Promicromonosporaceae;g__Isoptericola;__ | 0 |
| d__Bacteria;p__Firmicutes;c__Bacilli;o__Paenibacillales;f__Paenibacillaceae;g__Thermobacillus;s__uncultured_compost | 0 |
| d__Bacteria;p__Proteobacteria;c__Alphaproteobacteria;o__Kiloniellales;f__Kiloniellaceae;g__uncultured;s__uncultured_bacterium | 0 |
| d__Bacteria;p__Proteobacteria;c__Gammaproteobacteria;o__Gammaproteobacteria_Incertae_Sedis;f__Unknown_Family;g__Candidatus_Berkiella;__ | 0 |
| d__Bacteria;p__Firmicutes;c__Bacilli;o__Caldalkalibacillales;f__Caldalkalibacillaceae;g__Caldalkalibacillus;__ | 0 |
| d__Bacteria;p__Firmicutes;c__Bacilli;o__Paenibacillales;f__Paenibacillaceae;g__Thermobacillus;s__Thermobacillus_composti | 0 |
| d__Bacteria;p__Gemmatimonadota;c__Gemmatimonadetes;o__Gemmatimonadales;f__Gemmatimonadaceae;__;__ | 0 |
| d__Bacteria;p__Actinobacteriota;c__Actinobacteria;o__Micromonosporales;f__Micromonosporaceae;g__Phytohabitans;__ | 0 |
| d__Bacteria;p__Bacteroidota;c__Bacteroidia;o__Flavobacteriales;f__Cryomorphaceae;__;__ | 0 |
| d__Bacteria;p__Proteobacteria;c__Alphaproteobacteria;o__Rhizobiales;f__Labraceae;g__Labrys;s__metagenome | 0 |
| d__Bacteria;p__Firmicutes;c__Clostridia;o__Peptostreptococcales-Tissierellales;f__Peptostreptococcales-Tissierellales;g__Brassicibacter;__ | 0 |
| d__Bacteria;p__Proteobacteria;c__Alphaproteobacteria;o__Rhizobiales;f__Beijerinckiaceae;g__Bosea;__ | 0 |
| d__Bacteria;p__Chloroflexi;c__Chloroflexia;o__Chloroflexales;f__Roseiflexaceae;g__Roseiflexus;__ | 0 |
| d__Bacteria;p__Actinobacteriota;c__Thermoleophilia;o__Solirubrobacterales;f__Solirubrobacteraceae;g__Parviterribacter;__ | 0 |
| d__Bacteria;p__Firmicutes;c__Clostridia;o__Lachnospirales;f__Lachnospiraceae;g__Lachnoclostridium;s__[Clostridium]_sphenoides | 0 |
| d__Bacteria;p__Actinobacteriota;c__Actinobacteria;o__Propionibacteriales;f__Nocardioidaceae;g__Aeromicrobium;s__Aeromicrobium_sp. | 0 |
| d__Bacteria;p__Hydrogenedentes;c__Hydrogenedentia;o__Hydrogenedentiales;f__Hydrogenedensaceae;g__Hydrogenedensaceae;s__uncultured_bacterium | 0 |
| d__Bacteria;p__Actinobacteriota;c__Actinobacteria;o__Corynebacteriales;f__Mycobacteriaceae;g__Mycobacterium;s__Mycobacterium_sp. | 0 |
| d__Bacteria;p__Actinobacteriota;c__Actinobacteria;o__Propionibacteriales;f__Nocardioidaceae;g__Marmoricola;s__Marmoricola_sp. | 0 |
| d__Bacteria;p__Firmicutes;c__Bacilli;o__Exiguobacterales;f__Exiguobacteraceae;g__Exiguobacterium;s__metagenome | 0 |
| d__Bacteria;p__Proteobacteria;c__Gammaproteobacteria;o__Xanthomonadales;f__Xanthomonadaceae;g__Lysobacter;s__Lysobacter_sp. | 0 |
| d__Bacteria;p__Firmicutes;c__Symbiobacteriia;o__Symbiobacteriales;f__uncultured;g__uncultured;s__uncultured_bacterium | 0 |
| d__Bacteria;p__Proteobacteria;c__Gammaproteobacteria;o__Burkholderiales;f__Rhodocyclaceae;g__Azonexus;__ | 0 |
| d__Bacteria;p__Proteobacteria;c__Alphaproteobacteria;o__Sphingomonadales;f__Sphingomonadaceae;g__Sphingomonas;s__Sphingomonas_sp. | 0 |
| d__Bacteria;p__Proteobacteria;c__Alphaproteobacteria;o__Rhizobiales;f__D05-2;g__D05-2;s__alpha_proteobacterium | 0 |
| d__Bacteria;p__Bacteroidota;c__Bacteroidia;o__Chitinophagales;f__Chitinophagaceae;g__Chitinophaga;s__Chitinophaga_cymbidii | 0 |
| d__Bacteria;p__Firmicutes;c__Clostridia;o__Peptostreptococcales-Tissierellales;f__Thermotaleaceae;__;__ | 0 |
| d__Bacteria;p__Proteobacteria;c__Alphaproteobacteria;o__Elsterales;f__uncultured;g__uncultured;s__metagenome | 0 |
| d__Bacteria;p__Proteobacteria;c__Alphaproteobacteria;o__Azospirillales;f__Azospirillaceae;g__Skermanella;__ | 0 |
| d__Bacteria;p__Cyanobacteria;c__Cyanobacteriia;o__Chloroplast;f__Chloroplast;g__Chloroplast;s__uncultured_organism | 0 |
| d__Bacteria;p__Proteobacteria;c__Gammaproteobacteria;o__JTB23;f__JTB23;g__JTB23;__ | 0 |
| d__Bacteria;p__Proteobacteria;c__Alphaproteobacteria;o__Rhizobiales;f__Rhizobiaceae;g__Ciceribacter;__ | 0 |
| d__Bacteria;p__Actinobacteriota;c__Actinobacteria;o__Micromonosporales;f__Micromonosporaceae;g__Actinocatenispora;__ | 0 |
| d__Bacteria;p__Firmicutes;c__Bacilli;o__Bacillales;f__Planococcaceae;g__Domibacillus;s__metagenome | 0 |
| d__Bacteria;p__Firmicutes;c__Sulfobacillia;o__Sulfobacillales;f__Sulfobacillaceae;g__uncultured;s__bacterium_enrichment | 0 |
| d__Bacteria;p__Proteobacteria;c__Alphaproteobacteria;o__Acetobacterales;f__Acetobacteraceae;g__Acidicaldus;__ | 0 |
| d__Bacteria;p__Bacteroidota;c__Bacteroidia;o__Sphingobacteriales;f__NS11-12_marine_group;g__NS11-12_marine_group;s__uncultured_bacterium | 0 |
| d__Bacteria;p__Proteobacteria;c__Gammaproteobacteria;o__Steroidobacterales;f__Woeseiaceae;g__Woeseia;__ | 0 |
| d__Bacteria;p__Proteobacteria;c__Alphaproteobacteria;o__Sphingomonadales;f__Sphingomonadaceae;g__Hephaestia;__ | 0 |
| d__Bacteria;p__Elusimicrobiota;c__Elusimicrobia;o__Lineage_IV;f__Lineage_IV;g__Lineage_IV;s__uncultured_bacterium | 0 |
| d__Bacteria;p__Acidobacteriota;c__Acidobacteriae;o__PAUC26f;f__PAUC26f;g__PAUC26f;__ | 0 |
| d__Bacteria;p__Actinobacteriota;c__Actinobacteria;o__Streptomycetales;f__Streptomycetaceae;g__Streptomyces;s__Streptomyces_pluripotens | 0 |
| d__Bacteria;p__Proteobacteria;c__Gammaproteobacteria;o__Burkholderiales;f__Comamonadaceae;g__Acidovorax;s__uncultured_bacterium | 0 |
| d__Bacteria;p__Firmicutes;c__Bacilli;o__Paenibacillales;f__Paenibacillaceae;g__Paenibacillus;s__Paenibacillus_baekrokdamisoli | 0 |
| d__Bacteria;p__Bacteroidota;c__Bacteroidia;o__Cytophagales;f__Spirosomaceae;g__Persicitalea;__ | 0 |
| d__Bacteria;p__Firmicutes;c__Bacilli;o__Paenibacillales;f__Paenibacillaceae;g__uncultured;__ | 0 |
| d__Bacteria;p__Firmicutes;c__Clostridia;o__Oscillospirales;f__Ruminococcaceae;g__CAG-352;s__uncultured_rumen | 0 |
| d__Bacteria;p__Hydrogenedentes;c__Hydrogenedentia;o__Hydrogenedentiales;f__Hydrogenedensaceae;g__Hydrogenedensaceae;__ | 0 |
| d__Bacteria;p__Actinobacteriota;c__Actinobacteria;o__Streptosporangiales;f__Thermomonosporaceae;g__Actinoallomurus;__ | 0 |
| d__Bacteria;p__Proteobacteria;c__Alphaproteobacteria;o__Rhodospirillales;f__uncultured;g__uncultured;__ | 0 |
| d__Bacteria;p__Bacteroidota;c__Bacteroidia;__;__;__;__ | 0 |
| d__Bacteria;p__Planctomycetota;c__Phycisphaerae;o__Phycisphaerales;f__Phycisphaeraceae;g__AKYG587;s__uncultured_bacterium | 0 |
| d__Bacteria;p__Actinobacteriota;c__Actinobacteria;o__Corynebacteriales;f__Nocardiaceae;g__Nocardia;s__Nocardia_veterana | 0 |
| d__Bacteria;p__Firmicutes;c__Bacilli;o__Paenibacillales;f__Paenibacillaceae;g__Paenibacillus;s__Paenibacillus_graminis | 0 |
| d__Bacteria;p__Planctomycetota;c__Planctomycetes;o__Gemmatales;f__Gemmataceae;g__Fimbriiglobus;__ | 0 |
| d__Bacteria;p__Proteobacteria;c__Alphaproteobacteria;o__Defluviicoccales;f__uncultured;g__uncultured;__ | 0 |
| d__Bacteria;p__Actinobacteriota;c__Actinobacteria;o__Streptosporangiales;f__Thermomonosporaceae;g__Actinocorallia;__ | 0 |
| d__Bacteria;p__Proteobacteria;c__Alphaproteobacteria;o__Rhizobiales;f__Rhodomicrobiaceae;g__Rhodomicrobium;__ | 0 |
| d__Bacteria;p__Proteobacteria;c__Gammaproteobacteria;o__Burkholderiales;f__Comamonadaceae;g__Aquabacterium;__ | 0 |
| d__Bacteria;p__Desulfobacterota;c__Desulfuromonadia;o__Bradymonadales;f__Bradymonadales;g__Bradymonadales;s__uncultured_delta | 0 |
| d__Bacteria;p__Proteobacteria;c__Alphaproteobacteria;o__Caulobacterales;f__Caulobacteraceae;g__Brevundimonas;s__uncultured_bacterium | 0 |
| d__Bacteria;p__Proteobacteria;c__Gammaproteobacteria;o__Steroidobacterales;f__Woeseiaceae;g__Woeseia;s__uncultured_bacterium | 0 |
| d__Bacteria;p__Proteobacteria;c__Alphaproteobacteria;o__Rhodobacterales;f__Rhodobacteraceae;g__Paracoccus;__ | 0 |
| d__Bacteria;p__Bacteroidota;c__Bacteroidia;o__Bacteroidales;f__Prolixibacteraceae;g__Meniscus;__ | 0 |
| d__Bacteria;p__Proteobacteria;c__Gammaproteobacteria;o__Burkholderiales;f__Comamonadaceae;g__Ideonella;__ | 0 |
| d__Bacteria;p__Actinobacteriota;c__Actinobacteria;o__Pseudonocardiales;f__Pseudonocardiaceae;__;__ | 0 |
| d__Bacteria;p__Actinobacteriota;c__Actinobacteria;o__Streptomycetales;f__Streptomycetaceae;g__Streptomyces;s__Streptomyces_thermovulgaris | 0 |
| d__Bacteria;p__Proteobacteria;c__Gammaproteobacteria;o__Burkholderiales;f__Comamonadaceae;g__Comamonas;__ | 0 |
| d__Bacteria;p__Actinobacteriota;c__Actinobacteria;o__Micrococcales;f__Micrococcaceae;g__Arthrobacter;s__Arthrobacter_sp. | 0 |
| d__Bacteria;p__Proteobacteria;c__Gammaproteobacteria;o__Pseudomonadales;f__Pseudomonadaceae;g__Pseudomonas;s__Pseudomonas_oryzihabitans | 0 |
| d__Bacteria;p__Myxococcota;c__Myxococcia;o__Myxococcales;f__Anaeromyxobacteraceae;g__Anaeromyxobacter;__ | 0 |
| d__Bacteria;p__Proteobacteria;c__Gammaproteobacteria;o__Salinisphaerales;f__Solimonadaceae;g__Panacagrimonas;s__uncultured_bacterium | 0 |
| d__Bacteria;p__Firmicutes;c__Bacilli;o__Bacillales;f__Planococcaceae;g__uncultured;s__Bacillus_sp. | 0 |
| d__Bacteria;p__Proteobacteria;c__Gammaproteobacteria;o__Coxiellales;f__Coxiellaceae;g__Coxiella;s__uncultured_Coxiella | 0 |
| d__Bacteria;p__Planctomycetota;c__Planctomycetes;o__Isosphaerales;f__Isosphaeraceae;g__Paludisphaera;s__Paludisphaera_borealis | 0 |
| d__Bacteria;p__Proteobacteria;c__Gammaproteobacteria;o__Burkholderiales;f__Burkholderiaceae;g__Polynucleobacter;__ | 0 |
| d__Bacteria;p__Actinobacteriota;c__Actinobacteria;o__Corynebacteriales;f__Nocardiaceae;g__Smaragdicoccus;__ | 0 |
| d__Bacteria;p__Proteobacteria;c__Alphaproteobacteria;o__Sphingomonadales;f__Sphingomonadaceae;g__Qipengyuania;__ | 0 |
| d__Bacteria;p__Proteobacteria;c__Alphaproteobacteria;o__Rhodobacterales;f__Rhodobacteraceae;g__uncultured;s__uncultured_bacterium | 0 |
| d__Bacteria;p__Bacteroidota;c__Bacteroidia;o__Flavobacteriales;f__Flavobacteriaceae;g__Flavobacterium;s__Flavobacterium_rivuli | 0 |
| d__Bacteria;p__Bacteroidota;c__Bacteroidia;o__Bacteroidales;f__Bacteroidetes_vadinHA17;g__Bacteroidetes_vadinHA17;__ | 0 |
| d__Bacteria;p__Myxococcota;c__Polyangia;o__Polyangiales;__;__;__ | 0 |
| d__Bacteria;p__Proteobacteria;c__Gammaproteobacteria;o__Burkholderiales;f__TRA3-20;g__TRA3-20;__ | 0 |
| d__Bacteria;p__Cyanobacteria;c__Cyanobacteriia;o__Chloroplast;f__Chloroplast;g__Chloroplast;s__Bryum_argenteum | 0 |
| d__Bacteria;p__Proteobacteria;c__Gammaproteobacteria;o__Burkholderiales;f__Comamonadaceae;g__Rivibacter;s__Schlegelella_thermodepolymerans | 0 |
| d__Bacteria;p__Actinobacteriota;c__Actinobacteria;o__Streptomycetales;f__Streptomycetaceae;g__Streptacidiphilus;__ | 0 |
| d__Bacteria;p__Proteobacteria;c__Alphaproteobacteria;o__uncultured;f__uncultured;g__uncultured;s__uncultured_Rhodospirillaceae | 0 |
| d__Bacteria;p__Myxococcota;c__Polyangia;o__FFCH16767;f__FFCH16767;g__FFCH16767;s__uncultured_bacterium | 0 |
| d__Bacteria;p__Firmicutes;c__Clostridia;o__Oscillospirales;f__Oscillospiraceae;g__Oscillospira;__ | 0 |
| d__Bacteria;p__Actinobacteriota;c__Actinobacteria;o__Micrococcales;f__Promicromonosporaceae;g__Cellulosimicrobium;s__Cellulosimicrobium_cellulans | 0 |
| d__Bacteria;p__Firmicutes;c__Negativicutes;o__Veillonellales-Selenomonadales;f__Sporomusaceae;g__uncultured;s__uncultured_bacterium | 0 |
| d__Bacteria;p__Bacteroidota;c__Bacteroidia;o__Chitinophagales;f__uncultured;g__uncultured;s__metagenome | 0 |
| d__Bacteria;p__Proteobacteria;c__Gammaproteobacteria;o__Burkholderiales;f__Burkholderiaceae;g__Burkholderia-Caballeronia-Paraburkholderia;s__uncultured_bacterium | 0 |
| d__Bacteria;p__Proteobacteria;c__Gammaproteobacteria;o__Burkholderiales;f__Rhodocyclaceae;g__Methyloversatilis;s__beta_proteobacterium | 0 |
| d__Bacteria;p__Proteobacteria;c__Gammaproteobacteria;o__Ectothiorhodospirales;f__Thioalkalispiraceae;g__uncultured;__ | 0 |
| d__Bacteria;p__Proteobacteria;c__Alphaproteobacteria;o__Rhizobiales;f__Devosiaceae;g__uncultured;s__metagenome | 0 |
| d__Bacteria;p__Firmicutes;c__Clostridia;o__Oscillospirales;f__Hungateiclostridiaceae;g__Hungateiclostridium;__ | 0 |
| d__Bacteria;p__Actinobacteriota;c__Thermoleophilia;o__Solirubrobacterales;f__Solirubrobacteraceae;g__uncultured;__ | 0 |
| d__Bacteria;p__Proteobacteria;c__Alphaproteobacteria;o__Azospirillales;f__Inquilinaceae;g__Inquilinus;__ | 0 |
| d__Bacteria;p__Actinobacteriota;c__Actinobacteria;o__Micromonosporales;f__Micromonosporaceae;g__Luedemannella;__ | 0 |
| d__Bacteria;p__Bacteroidota;c__Bacteroidia;o__Cytophagales;f__Microscillaceae;g__Ohtaekwangia;__ | 0 |
| d__Bacteria;p__Proteobacteria;c__Gammaproteobacteria;o__Burkholderiales;f__B1-7BS;g__B1-7BS;s__uncultured_bacterium | 0 |
| d__Bacteria;p__Bacteroidota;c__Bacteroidia;o__Cytophagales;f__Spirosomaceae;g__Persicitalea;s__uncultured_bacterium | 0 |
| d__Bacteria;p__Actinobacteriota;c__Actinobacteria;o__Corynebacteriales;f__Nocardiaceae;g__Gordonia;s__Gordonia_kroppenstedtii | 0 |
| d__Bacteria;p__Proteobacteria;c__Alphaproteobacteria;o__Reyranellales;f__Reyranellaceae;g__Reyranella;s__metagenome | 0 |
| d__Bacteria;p__Actinobacteriota;c__Actinobacteria;o__Streptosporangiales;f__Streptosporangiaceae;g__Sphaerisporangium;__ | 0 |
| d__Bacteria;p__Actinobacteriota;c__Actinobacteria;o__Corynebacteriales;f__Nocardiaceae;g__Nocardia;__ | 0 |
| d__Bacteria;p__Acidobacteriota;c__Acidobacteriae;__;__;__;__ | 0 |
| d__Bacteria;p__Firmicutes;c__Bacilli;o__Bacillales;f__Sporolactobacillaceae;g__Terrilactibacillus;__ | 0 |
| d__Bacteria;p__Actinobacteriota;c__Actinobacteria;o__Micrococcales;f__Micrococcaceae;g__Pseudarthrobacter;s__Pseudarthrobacter_siccitolerans | 0 |
| d__Bacteria;p__Firmicutes;c__Dethiobacteria;o__Dethiobacterales;f__Dethiobacteraceae;g__uncultured;__ | 0 |
| d__Bacteria;p__Actinobacteriota;c__Actinobacteria;o__Frankiales;f__uncultured;g__uncultured;__ | 0 |
| d__Bacteria;p__Proteobacteria;c__Alphaproteobacteria;o__Rhizobiales;f__Xanthobacteraceae;g__Pseudoxanthobacter;__ | 0 |
| d__Bacteria;p__Proteobacteria;c__Gammaproteobacteria;o__Burkholderiales;f__Burkholderiaceae;g__Lautropia;s__uncultured_bacterium | 0 |
| d__Bacteria;p__Firmicutes;c__Bacilli;o__Bacillales;f__Bacillaceae;g__Bacillus;s__Bacillus_safensis | 0 |
| d__Bacteria;p__Proteobacteria;c__Gammaproteobacteria;o__Pseudomonadales;f__Pseudomonadaceae;g__Azotobacter;__ | 0 |
| d__Bacteria;p__Acidobacteriota;c__Thermoanaerobaculia;o__Thermoanaerobaculales;f__Thermoanaerobaculaceae;g__Subgroup_10;s__uncultured_bacterium | 0 |
| d__Bacteria;p__Bacteroidota;c__Bacteroidia;o__Bacteroidales;f__Paludibacteraceae;g__uncultured;__ | 0 |
| d__Bacteria;p__Desulfobacterota;c__uncultured;o__uncultured;f__uncultured;g__uncultured;s__uncultured_bacterium | 0 |
| d__Bacteria;p__NB1-j;c__NB1-j;o__NB1-j;f__NB1-j;g__NB1-j;s__uncultured_bacterium | 0 |
| d__Bacteria;p__Bacteroidota;c__Bacteroidia;o__Bacteroidales;f__Paludibacteraceae;g__uncultured;s__uncultured_bacterium | 0 |
| d__Bacteria;p__Proteobacteria;c__Alphaproteobacteria;o__Rickettsiales;f__SM2D12;g__SM2D12;__ | 0 |
| d__Bacteria;p__Bdellovibrionota;c__Bdellovibrionia;o__Bacteriovoracales;f__Bacteriovoracaceae;g__Peredibacter;s__uncultured_bacterium | 0 |
| d__Bacteria;p__Cyanobacteria;c__Vampirivibrionia;o__Gastranaerophilales;f__Gastranaerophilales;g__Gastranaerophilales;s__uncultured_bacterium | 0 |
| d__Bacteria;p__Firmicutes;c__Clostridia;o__Clostridiales;f__Clostridiaceae;g__Clostridium_sensu_stricto_13;s__uncultured_bacterium | 0 |
| d__Bacteria;p__Planctomycetota;c__Planctomycetes;o__Planctomycetales;f__Schlesneriaceae;g__Planctopirus;s__uncultured_bacterium | 0 |
| d__Bacteria;p__Proteobacteria;c__Alphaproteobacteria;o__Caulobacterales;f__Hyphomonadaceae;g__Hirschia;s__uncultured_bacterium | 0 |
| d__Bacteria;p__Actinobacteriota;c__Acidimicrobiia;o__Microtrichales;f__Ilumatobacteraceae;g__CL500-29_marine_group;s__uncultured_bacterium | 0 |
| d__Bacteria;p__Actinobacteriota;c__Actinobacteria;o__Streptosporangiales;f__Thermomonosporaceae;g__Thermobispora;s__Thermobispora_bispora | 0 |
| d__Bacteria;p__Firmicutes;c__Clostridia;o__Clostridiales;f__Clostridiaceae;g__Clostridium_sensu_stricto_12;s__Clostridium_tyrobutyricum | 0 |
| d__Bacteria;p__Acidobacteriota;c__Blastocatellia;o__Blastocatellales;f__Blastocatellaceae;g__Aridibacter;s__uncultured_bacterium | 0 |
| d__Bacteria;p__Firmicutes;c__Clostridia;o__Christensenellales;f__Christensenellaceae;g__Christensenellaceae_R-7_group;__ | 0 |
| d__Bacteria;p__Proteobacteria;c__Gammaproteobacteria;o__Pseudomonadales;f__Spongiibacteraceae;g__BD1-7_clade;__ | 0 |
| d__Bacteria;p__Proteobacteria;c__Alphaproteobacteria;o__Azospirillales;f__uncultured;g__uncultured;s__uncultured_bacterium | 0 |
| d__Bacteria;p__Proteobacteria;c__Alphaproteobacteria;o__Caulobacterales;f__Hyphomonadaceae;g__SWB02;s__uncultured_bacterium | 0 |
| d__Bacteria;p__Myxococcota;c__Polyangia;o__Polyangiales;f__Sandaracinaceae;g__uncultured;__ | 0 |
| d__Bacteria;p__Firmicutes;c__Clostridia;o__Oscillospirales;f__Hungateiclostridiaceae;g__Pseudobacteroides;__ | 0 |
| d__Bacteria;p__Spirochaetota;c__Spirochaetia;o__Spirochaetales;f__Spirochaetaceae;__;__ | 0 |
| d__Bacteria;p__Firmicutes;c__Bacilli;o__Staphylococcales;f__Staphylococcaceae;g__Staphylococcus;s__Staphylococcus_warneri | 0 |
| d__Bacteria;p__Actinobacteriota;c__Actinobacteria;o__Streptosporangiales;f__Streptosporangiaceae;g__Nonomuraea;s__Nonomuraea_sp. | 0 |
| d__Bacteria;p__Firmicutes;c__Negativicutes;o__Veillonellales-Selenomonadales;f__Sporomusaceae;g__uncultured;__ | 0 |
| d__Bacteria;p__Proteobacteria;c__Gammaproteobacteria;o__Burkholderiales;f__Nitrosomonadaceae;g__IS-44;s__uncultured_bacterium | 0 |
| d__Bacteria;p__Actinobacteriota;c__Acidimicrobiia;o__Actinomarinales;f__uncultured;g__uncultured;__ | 0 |
| d__Bacteria;p__Proteobacteria;c__Gammaproteobacteria;o__Acidiferrobacterales;f__Acidiferrobacteraceae;g__Sulfurifustis;s__uncultured_bacterium | 0 |
| d__Bacteria;p__Planctomycetota;c__Planctomycetes;o__Pirellulales;f__Pirellulaceae;g__Pir4_lineage;s__uncultured_bacterium | 0 |
| d__Bacteria;p__Acidobacteriota;c__Blastocatellia;o__Blastocatellales;f__Blastocatellaceae;__;__ | 0 |
| d__Bacteria;p__Firmicutes;c__Clostridia;o__Peptostreptococcales-Tissierellales;f__Family_XI;__;__ | 0 |
| d__Bacteria;p__Myxococcota;c__Polyangia;o__Nannocystales;f__Nannocystaceae;__;__ | 0 |
| d__Bacteria;p__Myxococcota;c__Polyangia;o__Polyangiales;f__BIrii41;g__BIrii41;__ | 0 |
| d__Bacteria;p__Cyanobacteria;c__Vampirivibrionia;o__Gastranaerophilales;f__Gastranaerophilales;g__Gastranaerophilales;__ | 0 |
| d__Bacteria;p__Firmicutes;c__Clostridia;o__Oscillospirales;f__Ruminococcaceae;g__Candidatus_Soleaferrea;__ | 0 |
| d__Bacteria;p__SAR324_clade(Marine_group_B);c__SAR324_clade(Marine_group_B);o__SAR324_clade(Marine_group_B);f__SAR324_clade(Marine_group_B);g__SAR324_clade(Marine_group_B);__ | 0 |
| d__Bacteria;p__Firmicutes;c__Clostridia;o__Oscillospirales;__;__;__ | 0 |
| d__Bacteria;p__Firmicutes;c__Thermoanaerobacteria;o__Thermoanaerobacterales;f__Family_III;g__Thermoanaerobacterium;s__Thermoanaerobacterium_thermosaccharolyticum | 0 |
| d__Bacteria;p__Planctomycetota;c__Planctomycetes;o__Planctomycetales;f__uncultured;g__uncultured;__ | 0 |
| d__Bacteria;p__Actinobacteriota;c__Acidimicrobiia;o__Microtrichales;f__Microtrichaceae;g__IMCC26207;s__uncultured_bacterium | 0 |
| d__Bacteria;p__MBNT15;c__MBNT15;o__MBNT15;f__MBNT15;g__MBNT15;__ | 0 |
| d__Bacteria;p__Firmicutes;c__Bacilli;o__Bacillales;f__Bacillaceae;g__Bacillus;s__Bacillus_clausii | 0 |
| d__Bacteria;p__Firmicutes;c__Clostridia;o__Clostridia;f__Gracilibacteraceae;g__Gracilibacter;s__uncultured_bacterium | 0 |
| d__Bacteria;p__Firmicutes;c__Clostridia;o__Lachnospirales;f__Lachnospiraceae;g__Lachnoclostridium;s__Lachnoclostridium_phytofermentans | 0 |
| d__Bacteria;p__Verrucomicrobiota;c__Verrucomicrobiae;o__Chthoniobacterales;f__Chthoniobacteraceae;g__Chthoniobacter;s__uncultured_bacterium | 0 |
| d__Bacteria;p__Firmicutes;c__Clostridia;o__Clostridia;f__Gracilibacteraceae;__;__ | 0 |
| d__Bacteria;p__Chloroflexi;c__Chloroflexia;o__Kallotenuales;f__AKIW781;g__AKIW781;s__uncultured_bacterium | 0 |
| d__Bacteria;p__Proteobacteria;c__Gammaproteobacteria;o__Burkholderiales;f__Comamonadaceae;g__Variovorax;__ | 0 |
| d__Bacteria;p__Chloroflexi;c__Chloroflexia;o__Thermomicrobiales;f__JG30-KF-CM45;g__JG30-KF-CM45;__ | 0 |
| d__Bacteria;p__SAR324_clade(Marine_group_B);c__SAR324_clade(Marine_group_B);o__SAR324_clade(Marine_group_B);f__SAR324_clade(Marine_group_B);g__SAR324_clade(Marine_group_B);s__uncultured_bacterium | 0 |
| d__Bacteria;p__Actinobacteriota;c__Actinobacteria;o__Streptosporangiales;f__Streptosporangiaceae;g__Nonomuraea;__ | 0 |
| d__Bacteria;p__Proteobacteria;c__Gammaproteobacteria;o__Pseudomonadales;f__Alcanivoracaceae;g__Ketobacter;s__uncultured_bacterium | 0 |
| d__Bacteria;p__Actinobacteriota;c__Actinobacteria;o__Micrococcales;f__Cellulomonadaceae;g__Actinotalea;__ | 0 |
| d__Bacteria;p__Firmicutes;c__Clostridia;o__Christensenellales;f__Christensenellaceae;__;__ | 0 |
| d__Bacteria;p__Actinobacteriota;c__Acidimicrobiia;o__Actinomarinales;f__uncultured;g__uncultured;s__uncultured_bacterium | 0 |
| d__Bacteria;p__Bacteroidota;c__Bacteroidia;o__Bacteroidales;f__Bacteroidetes_vadinHA17;g__Bacteroidetes_vadinHA17;s__uncultured_bacterium | 0 |
| d__Bacteria;p__Planctomycetota;c__Phycisphaerae;o__Phycisphaerales;f__Phycisphaeraceae;g__Urania-1B-19_marine_sediment_group;s__uncultured_bacterium | 0 |
| d__Bacteria;p__Bacteroidota;c__Bacteroidia;o__Cytophagales;f__Cyclobacteriaceae;g__Imperialibacter;__ | 0 |
| d__Bacteria;p__Proteobacteria;c__Gammaproteobacteria;o__Burkholderiales;f__Nitrosomonadaceae;g__Ellin6067;__ | 0 |
| d__Bacteria;p__Proteobacteria;c__Alphaproteobacteria;o__Puniceispirillales;f__Puniceispirillales_Incertae_Sedis;g__Constrictibacter;s__uncultured_bacterium | 0 |
| d__Bacteria;p__Proteobacteria;c__Gammaproteobacteria;o__Burkholderiales;f__Comamonadaceae;g__Hylemonella;__ | 0 |
| d__Bacteria;p__Proteobacteria;c__Gammaproteobacteria;o__Burkholderiales;f__Rhodocyclaceae;g__uncultured;s__uncultured_bacterium | 0 |
| d__Bacteria;p__Firmicutes;c__Bacilli;o__Bacillales;f__Sporolactobacillaceae;g__Sporolactobacillus;__ | 0 |
| d__Bacteria;p__Firmicutes;c__Negativicutes;o__Veillonellales-Selenomonadales;f__uncultured;g__uncultured;__ | 0 |
| d__Bacteria;p__Verrucomicrobiota;c__Verrucomicrobiae;o__Opitutales;f__Puniceicoccaceae;__;__ | 0 |
| d__Bacteria;p__Acidobacteriota;c__Blastocatellia;o__Blastocatellales;f__Blastocatellaceae;g__Blastocatella;__ | 0 |
| d__Bacteria;p__Firmicutes;c__Thermoanaerobacteria;o__Thermoanaerobacterales;f__Thermoanaerobacteraceae;g__Caloribacterium;__ | 0 |
| d__Bacteria;p__Proteobacteria;c__Alphaproteobacteria;o__Rhizobiales;f__Rhizobiaceae;g__Chelativorans;__ | 0 |
| d__Bacteria;p__Acidobacteriota;c__Subgroup_5;o__Subgroup_5;f__Subgroup_5;g__Subgroup_5;s__uncultured_bacterium | 0 |
| d__Bacteria;p__Proteobacteria;c__Alphaproteobacteria;o__Rhizobiales;f__Rhodobiaceae;g__Tepidamorphus;__ | 0 |
| d__Bacteria;p__Latescibacterota;c__Latescibacteria;o__Latescibacterales;f__Latescibacteraceae;g__Candidatus_Latescibacter;s__uncultured_bacterium | 0 |
| d__Bacteria;p__Firmicutes;c__Negativicutes;o__Veillonellales-Selenomonadales;f__Sporomusaceae;g__Anaerosporomusa;s__uncultured_bacterium | 0 |
| d__Bacteria;p__Firmicutes;c__Negativicutes;o__Veillonellales-Selenomonadales;f__uncultured;g__uncultured;s__uncultured_bacterium | 0 |
| d__Bacteria;p__Acidobacteriota;c__Blastocatellia;o__Pyrinomonadales;f__Pyrinomonadaceae;g__RB41;__ | 0 |
| d__Bacteria;p__Patescibacteria;c__Microgenomatia;o__Candidatus_Roizmanbacteria;f__Candidatus_Roizmanbacteria;g__Candidatus_Roizmanbacteria;s__uncultured_bacterium | 0 |
| d__Bacteria;p__Proteobacteria;c__Alphaproteobacteria;o__Caulobacterales;f__Hyphomonadaceae;g__SWB02;__ | 0 |
| d__Bacteria;p__Myxococcota;c__Myxococcia;o__Myxococcales;f__Myxococcaceae;g__uncultured;__ | 0 |
| d__Bacteria;p__Proteobacteria;c__Gammaproteobacteria;o__R7C24;f__R7C24;g__R7C24;s__uncultured_bacterium | 0 |
| d__Bacteria;p__Bacteroidota;c__Kryptonia;o__Kryptoniales;f__BSV26;g__BSV26;s__uncultured_bacterium | 0 |
| d__Bacteria;p__Fibrobacterota;c__Chitinivibrionia;o__uncultured;f__uncultured;g__uncultured;s__uncultured_bacterium | 0 |
| d__Bacteria;p__Bacteroidota;c__Bacteroidia;o__Sphingobacteriales;f__NS11-12_marine_group;g__NS11-12_marine_group;__ | 0 |
| d__Bacteria;p__Proteobacteria;c__Gammaproteobacteria;o__Burkholderiales;f__Nitrosomonadaceae;g__Nitrosomonas;__ | 0 |
| d__Bacteria;p__Firmicutes;c__Clostridia;o__Clostridiales;f__Clostridiaceae;g__Clostridium_sensu_stricto_12;s__uncultured_bacterium | 0 |
| d__Bacteria;p__Firmicutes;c__Moorellia;o__Moorellales;f__Moorellaceae;g__Moorella;s__Moorella_thermoacetica | 0 |
| d__Bacteria;p__Planctomycetota;c__OM190;o__OM190;f__OM190;g__OM190;__ | 0 |
| d__Bacteria;p__Proteobacteria;c__Gammaproteobacteria;o__Ga0077536;f__Ga0077536;g__Ga0077536;s__uncultured_bacterium | 0 |
| d__Bacteria;p__Acidobacteriota;c__Blastocatellia;o__DS-100;f__DS-100;g__DS-100;s__uncultured_bacterium | 0 |
| d__Bacteria;p__Actinobacteriota;c__Actinobacteria;o__Propionibacteriales;f__Propionibacteriaceae;__;__ | 0 |
| d__Bacteria;p__Firmicutes;c__Clostridia;o__Clostridiales;f__Clostridiaceae;g__Clostridium_sensu_stricto_10;s__uncultured_bacterium | 0 |
| d__Bacteria;p__Latescibacterota;c__Latescibacterota;o__Latescibacterota;f__Latescibacterota;g__Latescibacterota;s__uncultured_bacterium | 0 |
| d__Bacteria;p__Firmicutes;c__Clostridia;o__Clostridiales;f__Clostridiaceae;g__Clostridium_sensu_stricto_10;__ | 0 |
| d__Bacteria;p__Firmicutes;c__Bacilli;o__Bacillales;f__Bacillaceae;g__Geobacillus;s__Geobacillus_sp. | 0 |
| d__Bacteria;p__Firmicutes;c__Bacilli;o__Alicyclobacillales;f__Alicyclobacillaceae;g__Effusibacillus;__ | 0 |
| d__Bacteria;p__Proteobacteria;c__Gammaproteobacteria;o__Arenicellales;f__Arenicellaceae;g__uncultured;__ | 0 |
| d__Bacteria;p__Proteobacteria;c__Gammaproteobacteria;o__Steroidobacterales;f__Woeseiaceae;__;__ | 0 |
| d__Bacteria;p__Spirochaetota;c__Spirochaetia;o__Spirochaetales;f__Spirochaetaceae;g__GWE2-31-10;__ | 0 |
| d__Bacteria;p__Proteobacteria;c__Gammaproteobacteria;o__Pseudomonadales;f__Alcanivoracaceae;g__Ketobacter;__ | 0 |
| d__Bacteria;p__Bacteroidota;c__Bacteroidia;o__Sphingobacteriales;f__Lentimicrobiaceae;g__Lentimicrobium;__ | 0 |
| d__Bacteria;p__Proteobacteria;c__Alphaproteobacteria;o__Rhizobiales;f__Kaistiaceae;g__Kaistia;__ | 0 |
| d__Bacteria;p__Proteobacteria;c__Gammaproteobacteria;o__Pseudomonadales;__;__;__ | 0 |
| d__Bacteria;p__Proteobacteria;c__Gammaproteobacteria;o__Enterobacterales;f__Pasteurellaceae;g__Aggregatibacter;s__Aggregatibacter_aphrophilus | 0 |
| d__Bacteria;p__Acidobacteriota;c__Holophagae;o__Subgroup_7;f__Subgroup_7;g__Subgroup_7;s__uncultured_bacterium | 0 |
| d__Bacteria;p__Planctomycetota;c__OM190;o__OM190;f__OM190;g__OM190;s__uncultured_bacterium | 0 |
| d__Bacteria;p__Proteobacteria;c__Alphaproteobacteria;o__Reyranellales;f__Reyranellaceae;g__uncultured;s__uncultured_bacterium | 0 |
| d__Bacteria;p__Methylomirabilota;c__Methylomirabilia;o__Rokubacteriales;f__Rokubacteriales;g__Rokubacteriales;s__uncultured_bacterium | 0 |
| d__Bacteria;p__Firmicutes;c__Clostridia;o__Clostridiales;f__Caloramatoraceae;g__Fonticella;__ | 0 |
| d__Bacteria;p__Firmicutes;c__Bacilli;o__Bacillales;f__Sporolactobacillaceae;g__Tuberibacillus;s__Tuberibacillus_calidus | 0 |
| d__Bacteria;p__Myxococcota;c__Polyangia;o__Nannocystales;f__Nannocystaceae;g__Nannocystis;__ | 0 |
| d__Bacteria;p__Chloroflexi;c__Anaerolineae;o__SBR1031;f__A4b;g__A4b;__ | 0 |
| d__Bacteria;p__Planctomycetota;c__Phycisphaerae;o__Pla1_lineage;f__Pla1_lineage;g__Pla1_lineage;s__uncultured_bacterium | 0 |
| d__Bacteria;p__Chloroflexi;c__Anaerolineae;o__Ardenticatenales;f__Ardenticatenaceae;g__uncultured;s__uncultured_bacterium | 0 |
| d__Bacteria;p__Proteobacteria;c__Gammaproteobacteria;o__Methylococcales;f__Methylococcaceae;__;__ | 0 |
| d__Bacteria;p__Firmicutes;c__Bacilli;o__Bacillales;f__Bacillaceae;g__Caldibacillus;s__uncultured_compost | 0 |
| d__Bacteria;p__Actinobacteriota;c__Coriobacteriia;o__OPB41;f__OPB41;g__OPB41;s__uncultured_bacterium | 0 |
| d__Bacteria;p__Desulfobacterota;c__Syntrophia;o__Syntrophales;__;__;__ | 0 |
| d__Bacteria;p__Planctomycetota;c__Planctomycetes;o__Pirellulales;f__Pirellulaceae;__;__ | 0 |
| d__Bacteria;p__Proteobacteria;c__Alphaproteobacteria;o__Rhizobiales;f__Beijerinckiaceae;g__Methylocystis;s__Methylocystis_sp. | 0 |
| d__Bacteria;p__Proteobacteria;c__Alphaproteobacteria;o__Kiloniellales;f__Fodinicurvataceae;g__uncultured;__ | 0 |
| d__Bacteria;p__Firmicutes;c__Bacilli;o__Lactobacillales;f__Lactobacillaceae;g__Ligilactobacillus;s__Lactobacillus_acidipiscis | 0 |
| d__Bacteria;p__Planctomycetota;c__Pla4_lineage;o__Pla4_lineage;f__Pla4_lineage;g__Pla4_lineage;__ | 0 |
| d__Bacteria;p__Chloroflexi;c__Anaerolineae;o__Anaerolineales;f__Anaerolineaceae;g__uncultured;__ | 0 |
| d__Bacteria;p__Proteobacteria;c__Gammaproteobacteria;o__PLTA13;f__PLTA13;g__PLTA13;s__uncultured_bacterium | 0 |
| d__Bacteria;p__Planctomycetota;c__Phycisphaerae;o__Tepidisphaerales;f__WD2101_soil_group;g__WD2101_soil_group;__ | 0 |
| d__Bacteria;p__Myxococcota;c__Polyangia;o__Blfdi19;f__Blfdi19;g__Blfdi19;s__uncultured_bacterium | 0 |
| d__Bacteria;p__Bacteroidota;c__Bacteroidia;o__Chitinophagales;f__37-13;g__37-13;__ | 0 |
| d__Bacteria;p__Firmicutes;c__Bacilli;o__Kyrpidiales;f__Kyrpidiaceae;g__Kyrpidia;__ | 0 |
| d__Bacteria;p__Proteobacteria;c__Alphaproteobacteria;o__Parvibaculales;f__Parvibaculaceae;g__Rhodoligotrophos;__ | 0 |
| d__Bacteria;p__Actinobacteriota;c__MB-A2-108;o__MB-A2-108;f__MB-A2-108;g__MB-A2-108;s__uncultured_bacterium | 0 |
| d__Bacteria;p__Bacteroidota;c__Bacteroidia;o__Cytophagales;f__Amoebophilaceae;g__Candidatus_Amoebophilus;__ | 0 |
| d__Bacteria;p__Acidobacteriota;c__Thermoanaerobaculia;o__Thermoanaerobaculales;f__Thermoanaerobaculaceae;g__Subgroup_10;__ | 0 |
| d__Bacteria;p__Firmicutes;c__Clostridia;o__Thermincolales;f__Thermincolaceae;g__Thermincola;s__uncultured_bacterium | 0 |
| d__Bacteria;p__Armatimonadota;c__Chthonomonadetes;o__Chthonomonadales;f__Chthonomonadales;g__Chthonomonadales;s__uncultured_bacterium | 0 |
| d__Bacteria;p__Planctomycetota;c__Planctomycetes;o__Pirellulales;f__Pirellulaceae;g__Pir4_lineage;__ | 0 |
| d__Bacteria;p__Firmicutes;c__Negativicutes;o__Veillonellales-Selenomonadales;f__Sporomusaceae;g__Anaerospora;s__uncultured_bacterium | 0 |
| d__Bacteria;p__Spirochaetota;c__Spirochaetia;o__Spirochaetales;f__Spirochaetaceae;g__GWE2-31-10;s__uncultured_organism | 0 |
| d__Bacteria;p__Chloroflexi;c__Chloroflexia;o__Chloroflexales;f__Roseiflexaceae;g__uncultured;__ | 0 |
| d__Bacteria;p__Planctomycetota;c__Phycisphaerae;o__mle1-8;f__mle1-8;g__mle1-8;s__uncultured_bacterium | 0 |
| d__Bacteria;p__Firmicutes;c__Clostridia;o__Oscillospirales;f__Ruminococcaceae;__;__ | 0 |
| d__Bacteria;p__Firmicutes;c__Clostridia;o__Oscillospirales;f__Oscillospirales;g__Hydrogenoanaerobacterium;s__uncultured_bacterium | 0 |
| d__Bacteria;p__Spirochaetota;c__Spirochaetia;o__Spirochaetales;f__Spirochaetaceae;g__Treponema;__ | 0 |
| d__Bacteria;p__Bacteroidota;c__Rhodothermia;o__Rhodothermales;f__Rhodothermaceae;g__uncultured;s__uncultured_bacterium | 0 |
| d__Bacteria;p__Bacteroidota;c__Ignavibacteria;o__Ignavibacteriales;f__Ignavibacteriaceae;g__Ignavibacterium;s__uncultured_bacterium | 0 |
| d__Bacteria;p__Firmicutes;c__Clostridia;o__Clostridiales;f__Caloramatoraceae;__;__ | 0 |
| d__Bacteria;p__Proteobacteria;c__Alphaproteobacteria;o__Rhizobiales;f__Devosiaceae;g__Devosia;s__bacterium_PA2 | 0 |
| d__Bacteria;p__Firmicutes;c__Clostridia;o__Clostridiales;f__Oxobacteraceae;g__Oxobacter;s__uncultured_bacterium | 0 |
| d__Bacteria;p__Firmicutes;c__Limnochordia;o__Limnochordia;f__Limnochordia;g__Hydrogenispora;__ | 0 |
| d__Bacteria;p__Proteobacteria;c__Alphaproteobacteria;o__Defluviicoccales;__;__;__ | 0 |
| d__Bacteria;p__Chloroflexi;c__Chloroflexia;o__Chloroflexales;f__Chloroflexaceae;g__Candidatus_Chloroploca;__ | 0 |
| d__Bacteria;p__Bacteroidota;c__Bacteroidia;o__Sphingobacteriales;f__AKYH767;g__AKYH767;__ | 0 |
| d__Bacteria;p__Firmicutes;c__Limnochordia;o__Limnochordia;f__Limnochordia;g__Hydrogenispora;s__uncultured_compost | 0 |
| d__Bacteria;p__Firmicutes;c__Clostridia;o__Peptostreptococcales-Tissierellales;f__Family_XI;g__Peptoniphilus;__ | 0 |
| d__Bacteria;p__Bdellovibrionota;c__Bdellovibrionia;o__Bacteriovoracales;f__Bacteriovoracaceae;g__Bacteriovorax;s__uncultured_bacterium | 0 |
| d__Bacteria;p__Armatimonadota;__;__;__;__;__ | 0 |
| d__Bacteria;p__Firmicutes;c__Desulfotomaculia;o__Desulfotomaculales;f__Desulfotomaculaceae;g__Desulfotomaculum;__ | 0 |
| d__Bacteria;p__Proteobacteria;c__Gammaproteobacteria;o__Pseudomonadales;f__Porticoccaceae;g__C1-B045;s__uncultured_bacterium | 0 |
| d__Bacteria;p__Actinobacteriota;c__Acidimicrobiia;o__Microtrichales;f__Microtrichaceae;g__IMCC26207;__ | 0 |
| d__Bacteria;p__Proteobacteria;c__Gammaproteobacteria;o__Xanthomonadales;f__Rhodanobacteraceae;g__uncultured;__ | 0 |
| d__Bacteria;p__Myxococcota;c__Polyangia;o__Polyangiales;f__Polyangiaceae;g__uncultured;s__uncultured_bacterium | 0 |
| d__Bacteria;p__Proteobacteria;c__Gammaproteobacteria;o__CCD24;f__CCD24;g__CCD24;__ | 0 |
| d__Bacteria;p__Proteobacteria;c__Gammaproteobacteria;o__Burkholderiales;f__Comamonadaceae;g__Hylemonella;s__uncultured_bacterium | 0 |
| d__Bacteria;p__Planctomycetota;c__vadinHA49;o__vadinHA49;f__vadinHA49;g__vadinHA49;s__uncultured_bacterium | 0 |
| d__Bacteria;p__WPS-2;c__WPS-2;o__WPS-2;f__WPS-2;g__WPS-2;__ | 0 |
| d__Bacteria;p__Proteobacteria;c__Gammaproteobacteria;o__Methylococcales;f__Methylococcaceae;g__uncultured;__ | 0 |
| d__Bacteria;p__Gemmatimonadota;c__BD2-11_terrestrial_group;o__BD2-11_terrestrial_group;f__BD2-11_terrestrial_group;g__BD2-11_terrestrial_group;s__uncultured_bacterium | 0 |
| d__Bacteria;p__Armatimonadota;c__Chthonomonadetes;o__Chthonomonadales;f__Chthonomonadales;g__Chthonomonadales;__ | 0 |
| d__Bacteria;p__Firmicutes;c__Desulfotomaculia;o__Desulfotomaculales;f__Pelotomaculaceae;__;__ | 0 |
| d__Bacteria;p__Bacteroidota;c__Bacteroidia;o__Bacteroidetes_VC2.1_Bac22;f__Bacteroidetes_VC2.1_Bac22;g__Bacteroidetes_VC2.1_Bac22;s__uncultured_bacterium | 0 |
| d__Bacteria;p__Firmicutes;c__Clostridia;o__Clostridia;f__Gracilibacteraceae;g__Gracilibacter;__ | 0 |
| d__Bacteria;p__Chloroflexi;c__Dehalococcoidia;o__S085;f__S085;g__S085;__ | 0 |
| d__Bacteria;p__Planctomycetota;c__Phycisphaerae;o__Phycisphaerales;f__Phycisphaeraceae;g__CL500-3;s__uncultured_bacterium | 0 |
| d__Bacteria;p__Verrucomicrobiota;c__Verrucomicrobiae;o__Chthoniobacterales;f__Chthoniobacteraceae;g__Chthoniobacter;__ | 0 |
| d__Bacteria;p__Proteobacteria;c__Gammaproteobacteria;o__Pseudomonadales;f__Moraxellaceae;g__uncultured;s__uncultured_bacterium | 0 |
| d__Bacteria;p__Firmicutes;c__Bacilli;o__Bacillales;f__Bacillaceae;g__Bacillus;s__Bacillus_pumilus | 0 |
| d__Bacteria;p__Acidobacteriota;c__Blastocatellia;o__Blastocatellales;f__Blastocatellaceae;g__JGI_0001001-H03;s__uncultured_bacterium | 0 |
| d__Bacteria;p__Bacteroidota;c__Bacteroidia;o__Bacteroidales;f__SB-5;g__SB-5;s__uncultured_organism | 0 |
| d__Bacteria;p__Gemmatimonadota;c__BD2-11_terrestrial_group;o__BD2-11_terrestrial_group;f__BD2-11_terrestrial_group;g__BD2-11_terrestrial_group;__ | 0 |
| d__Bacteria;p__Proteobacteria;c__Gammaproteobacteria;o__Enterobacterales;f__Pasteurellaceae;g__Aggregatibacter;s__uncultured_bacterium | 0 |
| d__Bacteria;p__Firmicutes;c__Desulfotomaculia;o__Desulfotomaculales;f__Desulfotomaculaceae;g__Desulfotomaculum;s__Desulfotomaculum_nigrificans | 0 |
| d__Bacteria;p__Firmicutes;c__Thermoanaerobacteria;o__Thermoanaerobacterales;f__Thermoanaerobacteraceae;g__Caldanaerobius;__ | 0 |
| d__Bacteria;p__Acidobacteriota;c__Blastocatellia;o__Blastocatellales;f__Blastocatellaceae;g__Aridibacter;__ | 0 |
| d__Bacteria;p__Planctomycetota;c__Planctomycetes;o__Pirellulales;f__Pirellulaceae;g__Pir2_lineage;__ | 0 |
| d__Bacteria;p__Acidobacteriota;c__Acidobacteriae;o__Bryobacterales;f__Bryobacteraceae;g__Bryobacter;s__metagenome | 0 |
| d__Bacteria;p__Acidobacteriota;c__Vicinamibacteria;o__Subgroup_17;f__Subgroup_17;g__Subgroup_17;__ | 0 |
| d__Bacteria;p__Fibrobacterota;c__Fibrobacteria;o__Fibrobacterales;f__Fibrobacteraceae;g__possible_genus_04;__ | 0 |
| d__Bacteria;p__Myxococcota;c__Polyangia;o__Polyangiales;f__Polyangiaceae;g__uncultured;__ | 0 |
| d__Bacteria;p__Firmicutes;c__Thermoanaerobacteria;o__Thermoanaerobacterales;f__Family_IV;g__Mahella;__ | 0 |
| d__Bacteria;p__Bacteroidota;c__Bacteroidia;o__Cytophagales;f__Cytophagaceae;g__Sporocytophaga;__ | 0 |
| d__Bacteria;p__Myxococcota;c__Polyangia;o__Polyangiales;f__Sandaracinaceae;g__uncultured;s__metagenome | 0 |
| d__Bacteria;p__Patescibacteria;c__uncultured;o__uncultured;f__uncultured;g__uncultured;s__uncultured_bacterium | 0 |
| d__Bacteria;p__Proteobacteria;c__Alphaproteobacteria;o__Micavibrionales;f__Micavibrionaceae;g__uncultured;s__uncultured_bacterium | 0 |
| d__Bacteria;p__Bacteroidota;c__Bacteroidia;o__Bacteroidales;f__SB-5;g__SB-5;__ | 0 |
| d__Bacteria;p__Acidobacteriota;c__Vicinamibacteria;o__Subgroup_17;f__Subgroup_17;g__Subgroup_17;s__uncultured_Acidobacteria | 0 |
| d__Bacteria;p__Desulfobacterota;c__Syntrophorhabdia;o__Syntrophorhabdales;f__Syntrophorhabdaceae;g__Syntrophorhabdus;s__uncultured_bacterium | 0 |
| d__Bacteria;p__Chloroflexi;c__Anaerolineae;o__SBR1031;f__SBR1031;g__SBR1031;s__uncultured_bacterium | 0 |
| d__Bacteria;p__Firmicutes;c__Bacilli;o__Bacillales;f__Bacillaceae;g__Bacillus;s__Bacillus_mycoides | 0 |
| d__Bacteria;p__Actinobacteriota;c__Actinobacteria;o__Streptosporangiales;f__Thermomonosporaceae;g__Thermostaphylospora;s__Thermostaphylospora_chromogena | 0 |
| d__Bacteria;p__Firmicutes;c__Bacilli;o__Lactobacillales;f__Streptococcaceae;g__Streptococcus;s__Streptococcus_cristatus | 0 |
| d__Bacteria;p__Firmicutes;c__Clostridia;o__Peptostreptococcales-Tissierellales;f__Family_XI;g__Tepidimicrobium;s__Tepidimicrobium_ferriphilum | 0 |
| d__Bacteria;p__Chloroflexi;c__Anaerolineae;o__Anaerolineales;f__Anaerolineaceae;g__uncultured;s__uncultured_bacterium | 0 |
| d__Bacteria;p__Acidobacteriota;c__Blastocatellia;o__Blastocatellales;f__Blastocatellaceae;g__Stenotrophobacter;s__uncultured_bacterium | 0 |
| d__Bacteria;p__Bacteroidota;c__Ignavibacteria;o__Ignavibacteriales;__;__;__ | 0 |
| d__Bacteria;p__Firmicutes;c__Bacilli;o__Bacillales;f__Bacillaceae;g__Aeribacillus;s__Aeribacillus_pallidus | 0 |
| d__Bacteria;p__Proteobacteria;c__Gammaproteobacteria;o__Burkholderiales;f__Rhodocyclaceae;g__Zoogloea;__ | 0 |
| d__Bacteria;p__Planctomycetota;c__Planctomycetes;o__uncultured;f__uncultured;g__uncultured;s__uncultured_bacterium | 0 |
| d__Bacteria;p__Chloroflexi;c__Chloroflexia;o__Thermomicrobiales;f__Thermomicrobiaceae;g__Sphaerobacter;s__Sphaerobacter_thermophilus | 0 |
| d__Bacteria;p__Myxococcota;c__bacteriap25;o__bacteriap25;f__bacteriap25;g__bacteriap25;s__uncultured_bacterium | 0 |
| d__Bacteria;p__Proteobacteria;c__Alphaproteobacteria;o__Thalassobaculales;f__uncultured;g__uncultured;__ | 0 |
| d__Bacteria;p__Armatimonadota;c__Fimbriimonadia;o__Fimbriimonadales;f__Fimbriimonadales;g__Fimbriimonadales;s__uncultured_bacterium | 0 |
| d__Bacteria;p__Bacteroidota;c__Bacteroidia;o__Cytophagales;f__Microscillaceae;g__Hassallia;__ | 0 |
| d__Bacteria;p__Desulfobacterota;c__Syntrophia;o__Syntrophales;f__Syntrophaceae;g__Syntrophus;s__uncultured_bacterium | 0 |
| d__Bacteria;p__Firmicutes;c__Clostridia;o__Eubacteriales;f__Garciellaceae;g__Garciella;__ | 0 |
| d__Bacteria;p__Firmicutes;c__Bacilli;o__Bacillales;f__Bacillaceae;g__Geobacillus;s__Geobacillus_thermoleovorans | 0 |
| d__Bacteria;p__Verrucomicrobiota;c__Verrucomicrobiae;o__Verrucomicrobiales;f__Verrucomicrobiaceae;g__Verrucomicrobium;__ | 0 |
| d__Bacteria;p__MBNT15;c__MBNT15;o__MBNT15;f__MBNT15;g__MBNT15;s__uncultured_bacterium | 0 |
| d__Bacteria;p__Firmicutes;c__Clostridia;o__Oscillospirales;f__Ethanoligenenaceae;g__Incertae_Sedis;s__uncultured_bacterium | 0 |
| d__Bacteria;p__Patescibacteria;c__Gracilibacteria;o__Candidatus_Peribacteria;f__Candidatus_Peribacteria;g__Candidatus_Peribacteria;s__uncultured_bacterium | 0 |
| d__Bacteria;p__Actinobacteriota;c__Acidimicrobiia;o__Microtrichales;f__Ilumatobacteraceae;g__CL500-29_marine_group;__ | 0 |
| d__Bacteria;p__Chloroflexi;c__Chloroflexia;o__Chloroflexales;f__Roseiflexaceae;g__uncultured;s__uncultured_bacterium | 0 |
| d__Bacteria;p__Spirochaetota;c__Spirochaetia;o__Spirochaetales;f__Spirochaetaceae;g__Spirochaeta;__ | 0 |
| d__Bacteria;p__Firmicutes;c__Desulfotomaculia;o__Desulfotomaculales;f__Desulfotomaculaceae;g__Desulfotomaculum;s__Desulfotomaculum_reducens | 0 |
| d__Bacteria;p__Firmicutes;c__Bacilli;o__Brevibacillales;f__Brevibacillaceae;g__Brevibacillus;__ | 0 |
| d__Bacteria;p__Acidobacteriota;c__Vicinamibacteria;o__Vicinamibacterales;f__Vicinamibacteraceae;g__uncultured;s__uncultured_bacterium | 0 |
| d__Bacteria;p__Cyanobacteria;c__Vampirivibrionia;o__Vampirovibrionales;f__Vampirovibrionaceae;g__Vampirovibrio;s__uncultured_bacterium | 0 |
| d__Bacteria;p__Actinobacteriota;c__Actinobacteria;o__Propionibacteriales;f__Propionibacteriaceae;g__Propionicicella;__ | 0 |
| d__Bacteria;p__Verrucomicrobiota;c__Verrucomicrobiae;o__Pedosphaerales;f__Pedosphaeraceae;g__Pedosphaeraceae;s__uncultured_bacterium | 0 |
| d__Bacteria;p__Proteobacteria;c__Gammaproteobacteria;o__Burkholderiales;f__B1-7BS;g__B1-7BS;__ | 0 |
| d__Bacteria;p__Proteobacteria;c__Alphaproteobacteria;o__Rhizobiales;f__Xanthobacteraceae;g__Pseudorhodoplanes;s__uncultured_bacterium | 0 |
| d__Bacteria;p__Desulfobacterota;c__Syntrophia;o__Syntrophales;f__Syntrophaceae;g__Syntrophus;__ | 0 |
| d__Bacteria;p__Actinobacteriota;c__Thermoleophilia;o__Solirubrobacterales;__;__;__ | 0 |
| d__Bacteria;p__Sumerlaeota;c__Sumerlaeia;o__uncultured;f__uncultured;g__uncultured;s__uncultured_organism | 0 |
| d__Bacteria;p__Firmicutes;c__Clostridia;o__Peptostreptococcales-Tissierellales;f__Peptostreptococcaceae;g__Sporacetigenium;__ | 0 |
| d__Bacteria;p__Acidobacteriota;c__Blastocatellia;o__Blastocatellales;f__Blastocatellaceae;g__uncultured;__ | 0 |
| d__Bacteria;p__Firmicutes;c__Clostridia;o__Clostridiales;f__Clostridiaceae;g__Clostridium_sensu_stricto_3;__ | 0 |
| d__Bacteria;p__Proteobacteria;c__Alphaproteobacteria;o__Parvibaculales;f__Parvibaculaceae;g__Parvibaculum;__ | 0 |
| d__Bacteria;p__Patescibacteria;c__Microgenomatia;o__Candidatus_Roizmanbacteria;f__Candidatus_Roizmanbacteria;g__Candidatus_Roizmanbacteria;__ | 0 |
| d__Bacteria;p__Bacteroidota;c__Bacteroidia;o__Bacteroidales;f__Prolixibacteraceae;g__BSV13;s__uncultured_bacterium | 0 |
| d__Bacteria;p__Desulfobacterota;c__Desulfuromonadia;o__PB19;f__PB19;g__PB19;__ | 0 |
| d__Bacteria;p__Myxococcota;__;__;__;__;__ | 0 |
| d__Bacteria;p__Actinobacteriota;c__Actinobacteria;o__Micrococcales;f__Micrococcaceae;g__Paenarthrobacter;s__Arthrobacter_sp. | 0 |
| d__Bacteria;p__Bacteroidota;c__Kapabacteria;o__Kapabacteriales;f__Kapabacteriales;g__Kapabacteriales;s__uncultured_bacterium | 0 |
| d__Bacteria;p__Firmicutes;c__Clostridia;o__Oscillospirales;f__Hungateiclostridiaceae;g__Thermoclostridium;s__Thermoclostridium_stercorarium | 0 |
| d__Bacteria;p__Proteobacteria;c__Alphaproteobacteria;o__Caulobacterales;f__Caulobacteraceae;g__Phenylobacterium;s__metagenome | 0 |
| d__Bacteria;p__Proteobacteria;c__Alphaproteobacteria;o__Caulobacterales;f__Hyphomonadaceae;g__Hirschia;s__metagenome | 0 |
| d__Bacteria;p__Myxococcota;c__Myxococcia;o__Myxococcales;f__Myxococcaceae;g__P3OB-42;s__uncultured_bacterium | 0 |
| d__Bacteria;p__Firmicutes;c__Symbiobacteriia;o__Symbiobacteriales;f__Symbiobacteraceae;g__Symbiobacterium;s__uncultured_bacterium | 0 |
| d__Bacteria;p__Firmicutes;c__Thermoanaerobacteria;o__Thermoanaerobacterales;f__Family_III;g__Thermoanaerobacterium;__ | 0 |
| d__Bacteria;p__Bacteroidota;c__Rhodothermia;o__Rhodothermales;f__Rhodothermaceae;__;__ | 0 |
| d__Bacteria;p__Patescibacteria;c__Gracilibacteria;o__Candidatus_Peregrinibacteria;f__Candidatus_Peregrinibacteria;g__Candidatus_Peregrinibacteria;s__uncultured_bacterium | 0 |
| d__Bacteria;p__Planctomycetota;c__Phycisphaerae;o__MSBL9;f__SG8-4;g__SG8-4;__ | 0 |
| d__Bacteria;p__Proteobacteria;c__Gammaproteobacteria;o__Xanthomonadales;f__Xanthomonadaceae;g__Xanthomonas;__ | 0 |
| d__Bacteria;p__Proteobacteria;c__Gammaproteobacteria;o__Gammaproteobacteria_Incertae_Sedis;f__Unknown_Family;g__Unknown_Family;s__uncultured_bacterium | 0 |
| d__Bacteria;p__Proteobacteria;c__Gammaproteobacteria;o__uncultured;f__uncultured;g__uncultured;s__uncultured_bacterium | 0 |
| d__Bacteria;p__Planctomycetota;c__Phycisphaerae;o__MSBL9;f__GWC2-45-44;g__GWC2-45-44;__ | 0 |
| d__Bacteria;p__Proteobacteria;c__Gammaproteobacteria;o__Enterobacterales;f__Pasteurellaceae;g__Aggregatibacter;__ | 0 |
| d__Bacteria;p__Proteobacteria;c__Gammaproteobacteria;o__WN-HWB-116;f__WN-HWB-116;g__WN-HWB-116;__ | 0 |
| d__Bacteria;p__Proteobacteria;c__Gammaproteobacteria;o__Pseudomonadales;f__Moraxellaceae;g__uncultured;s__uncultured_gamma | 0 |
| d__Bacteria;p__Chloroflexi;c__Anaerolineae;o__Caldilineales;f__Caldilineaceae;g__Litorilinea;s__uncultured_bacterium | 0 |
| d__Bacteria;p__Firmicutes;c__Clostridia;o__Oscillospirales;f__UCG-010;g__UCG-010;__ | 0 |
| d__Bacteria;p__Patescibacteria;c__Microgenomatia;o__Candidatus_Collierbacteria;f__Candidatus_Collierbacteria;g__Candidatus_Collierbacteria;s__uncultured_bacterium | 0 |
| d__Bacteria;p__Proteobacteria;c__Alphaproteobacteria;o__Sphingomonadales;f__Sphingomonadaceae;g__DSSF69;__ | 0 |
| d__Bacteria;p__FCPU426;c__FCPU426;o__FCPU426;f__FCPU426;g__FCPU426;s__uncultured_bacterium | 0 |
| d__Bacteria;p__Firmicutes;c__Clostridia;o__uncultured;f__uncultured;g__uncultured;s__uncultured_bacterium | 0 |
| d__Bacteria;p__Acidobacteriota;c__Blastocatellia;o__Blastocatellales;f__Blastocatellaceae;g__Stenotrophobacter;__ | 0 |
| d__Bacteria;p__Bacteroidota;c__Ignavibacteria;o__Ignavibacteriales;f__LD-RB-34;g__LD-RB-34;__ | 0 |
| d__Bacteria;p__Firmicutes;c__Limnochordia;o__Limnochordia;f__Limnochordia;g__Hydrogenispora;s__Clostridium_sp. | 0 |
| d__Bacteria;p__Firmicutes;c__Clostridia;o__Lachnospirales;f__Lachnospiraceae;g__Lachnoclostridium;s__Clostridium_sp. | 0 |
| d__Bacteria;p__Proteobacteria;c__Gammaproteobacteria;o__Pseudomonadales;f__Pseudomonadaceae;__;__ | 0 |
| d__Bacteria;p__Dependentiae;c__Babeliae;o__Babeliales;__;__;__ | 0 |
| d__Bacteria;p__Proteobacteria;c__Alphaproteobacteria;o__Rhizobiales;f__Rhizobiales_Incertae_Sedis;__;__ | 0 |
| d__Bacteria;p__Proteobacteria;c__Alphaproteobacteria;o__Rhizobiales;f__Beijerinckiaceae;g__Microvirga;s__uncultured_bacterium | 0 |
| d__Bacteria;p__Planctomycetota;c__Planctomycetes;o__Pirellulales;f__Pirellulaceae;g__Thermogutta;__ | 0 |
| d__Bacteria;p__Proteobacteria;c__Alphaproteobacteria;o__AT-s3-44;f__AT-s3-44;g__AT-s3-44;s__uncultured_bacterium | 0 |
| d__Bacteria;p__Chloroflexi;c__JG30-KF-CM66;o__JG30-KF-CM66;f__JG30-KF-CM66;g__JG30-KF-CM66;s__uncultured_bacterium | 0 |
| d__Bacteria;p__Actinobacteriota;c__Actinobacteria;o__Pseudonocardiales;f__Pseudonocardiaceae;g__Pseudonocardia;s__Pseudonocardia_sp. | 0 |
| d__Bacteria;p__Proteobacteria;c__Alphaproteobacteria;o__Rhizobiales;f__Rhizobiaceae;g__Ochrobactrum;__ | 0 |
| d__Bacteria;p__Desulfobacterota;c__Desulfuromonadia;o__Geobacterales;f__Geobacteraceae;g__uncultured;s__uncultured_bacterium | 0 |
| d__Bacteria;p__Patescibacteria;c__Microgenomatia;o__Candidatus_Curtissbacteria;f__Candidatus_Curtissbacteria;g__Candidatus_Curtissbacteria;s__uncultured_bacterium | 0 |
| d__Bacteria;p__Firmicutes;c__Negativicutes;o__Veillonellales-Selenomonadales;__;__;__ | 0 |
| d__Bacteria;p__Firmicutes;c__Desulfitobacteriia;o__Desulfitobacteriales;f__Syntrophobotulaceae;__;__ | 0 |
| d__Bacteria;p__Bacteroidota;c__Bacteroidia;o__Chitinophagales;f__Saprospiraceae;g__uncultured;s__uncultured_Saprospiraceae | 0 |
| d__Bacteria;p__Armatimonadota;c__Armatimonadia;o__Armatimonadales;f__Armatimonadales;g__Armatimonadales;__ | 0 |
| d__Bacteria;p__Bacteroidota;c__Bacteroidia;o__Chitinophagales;f__Saprospiraceae;g__uncultured;__ | 0 |
| d__Bacteria;p__Firmicutes;c__Thermoanaerobacteria;o__Thermoanaerobacterales;__;__;__ | 0 |
| d__Bacteria;p__Bacteroidota;c__Bacteroidia;o__Bacteroidales;f__Prolixibacteraceae;g__BSV13;__ | 0 |
| d__Bacteria;p__Proteobacteria;c__Gammaproteobacteria;o__Steroidobacterales;f__Steroidobacteraceae;g__uncultured;__ | 0 |
| d__Bacteria;p__Chloroflexi;c__Chloroflexia;o__Thermomicrobiales;f__Thermomicrobiaceae;g__Sphaerobacter;__ | 0 |
| d__Bacteria;p__Proteobacteria;c__Alphaproteobacteria;o__Acetobacterales;f__Acetobacteraceae;g__Craurococcus-Caldovatus;__ | 0 |
| d__Bacteria;p__Actinobacteriota;c__Actinobacteria;o__Micrococcales;f__Intrasporangiaceae;g__Ornithinicoccus;__ | 0 |
| d__Bacteria;p__Firmicutes;c__Bacilli;o__Bacillales;f__Bacillaceae;g__Bacillus;s__Bacillus_velezensis | 0 |
| d__Bacteria;p__Acidobacteriota;c__Vicinamibacteria;o__Vicinamibacterales;f__Vicinamibacteraceae;g__uncultured;__ | 0 |
| d__Bacteria;p__Desulfobacterota;c__Desulfobacteria;o__Desulfatiglandales;f__Desulfatiglandaceae;g__Desulfatiglans;__ | 0 |
| d__Bacteria;p__Patescibacteria;c__Microgenomatia;o__Candidatus_Daviesbacteria;f__Candidatus_Daviesbacteria;g__Candidatus_Daviesbacteria;__ | 0 |
| d__Bacteria;p__Proteobacteria;c__Alphaproteobacteria;o__Acetobacterales;f__Acetobacteraceae;g__Roseococcus;__ | 0 |
| d__Bacteria;p__Firmicutes;c__Incertae_Sedis;o__Incertae_Sedis;f__Incertae_Sedis;g__Gelria;__ | 0 |
| d__Bacteria;p__Acidobacteriota;c__Blastocatellia;o__DS-100;f__DS-100;g__DS-100;__ | 0 |
| d__Bacteria;p__Bacteroidota;c__Bacteroidia;o__Cytophagales;f__Microscillaceae;g__uncultured;__ | 0 |
| d__Bacteria;p__Firmicutes;c__Clostridia;o__Clostridiales;f__Clostridiaceae;g__Clostridium_sensu_stricto_9;__ | 0 |
| d__Bacteria;p__Proteobacteria;c__Gammaproteobacteria;o__Xanthomonadales;f__uncultured;g__uncultured;__ | 0 |
| d__Bacteria;p__Proteobacteria;c__Gammaproteobacteria;o__Xanthomonadales;f__Rhodanobacteraceae;g__uncultured;s__uncultured_bacterium | 0 |
| d__Bacteria;p__Desulfobacterota;c__Desulfuromonadia;o__PB19;f__PB19;g__PB19;s__uncultured_bacterium | 0 |
| d__Bacteria;p__Planctomycetota;c__Pla4_lineage;o__Pla4_lineage;f__Pla4_lineage;g__Pla4_lineage;s__uncultured_bacterium | 0 |
| d__Bacteria;p__Proteobacteria;c__Alphaproteobacteria;o__Rhizobiales;f__Rhizobiales_Incertae_Sedis;g__Phreatobacter;__ | 0 |
| d__Bacteria;p__Gemmatimonadota;c__S0134_terrestrial_group;o__S0134_terrestrial_group;f__S0134_terrestrial_group;g__S0134_terrestrial_group;s__uncultured_compost | 0 |
| d__Bacteria;p__Planctomycetota;c__Planctomycetes;o__Pirellulales;f__Pirellulaceae;g__Pirellula;s__metagenome | 0 |
| d__Bacteria;p__Chloroflexi;c__JG30-KF-CM66;o__JG30-KF-CM66;f__JG30-KF-CM66;g__JG30-KF-CM66;__ | 0 |
| d__Bacteria;p__Myxococcota;c__Myxococcia;o__Myxococcales;__;__;__ | 0 |
| d__Bacteria;p__Proteobacteria;c__Alphaproteobacteria;o__Rhizobiales;f__Beijerinckiaceae;g__Camelimonas;__ | 0 |
| d__Bacteria;p__Gemmatimonadota;c__BD2-11_terrestrial_group;o__BD2-11_terrestrial_group;f__BD2-11_terrestrial_group;g__BD2-11_terrestrial_group;s__metagenome | 0 |
| d__Bacteria;p__Desulfobacterota;c__Desulfuromonadia;__;__;__;__ | 0 |
| d__Bacteria;p__Actinobacteriota;c__Thermoleophilia;o__Solirubrobacterales;f__67-14;g__67-14;__ | 0 |
| d__Bacteria;p__Proteobacteria;c__Alphaproteobacteria;o__Rhizobiales;f__Xanthobacteraceae;g__Ancylobacter;__ | 0 |
| d__Bacteria;p__Bacteroidota;c__Ignavibacteria;o__Ignavibacteriales;f__LD-RB-34;g__LD-RB-34;s__uncultured_bacterium | 0 |
| d__Bacteria;p__Verrucomicrobiota;c__Verrucomicrobiae;o__Opitutales;f__Opitutaceae;g__Lacunisphaera;s__uncultured_bacterium | 0 |
| d__Bacteria;p__Chloroflexi;c__Chloroflexia;o__Chloroflexales;f__Herpetosiphonaceae;g__Herpetosiphon;__ | 0 |
| d__Bacteria;p__Bacteroidota;c__Rhodothermia;o__Rhodothermales;f__Rhodothermaceae;g__uncultured;__ | 0 |
| d__Bacteria;p__Patescibacteria;c__Microgenomatia;o__Candidatus_Levybacteria;f__Candidatus_Levybacteria;g__Candidatus_Levybacteria;s__uncultured_bacterium | 0 |
| d__Bacteria;p__Proteobacteria;c__Gammaproteobacteria;o__Burkholderiales;f__Comamonadaceae;g__Diaphorobacter;__ | 0 |
| d__Bacteria;p__Firmicutes;c__Desulfitobacteriia;o__Desulfitobacteriales;f__Desulfitobacteriaceae;g__Desulfitobacterium;__ | 0 |
| d__Bacteria;p__Proteobacteria;c__Gammaproteobacteria;o__Burkholderiales;f__Burkholderiaceae;g__Cupriavidus;__ | 0 |
| d__Bacteria;p__Proteobacteria;c__Alphaproteobacteria;o__Rhizobiales;f__KF-JG30-B3;g__KF-JG30-B3;s__uncultured_bacterium | 0 |
| d__Bacteria;p__Firmicutes;c__Bacilli;o__Staphylococcales;f__Staphylococcaceae;g__Staphylococcus;s__Staphylococcus_pasteuri | 0 |
| d__Bacteria;p__Planctomycetota;c__Planctomycetes;o__Pirellulales;f__Pirellulaceae;g__Pir4_lineage;s__metagenome | 0 |
| d__Bacteria;p__Verrucomicrobiota;c__Verrucomicrobiae;o__Opitutales;f__Opitutaceae;g__Cephaloticoccus;__ | 0 |
| d__Bacteria;p__Planctomycetota;c__Planctomycetes;o__Gemmatales;f__Gemmataceae;g__Fimbriiglobus;s__uncultured_bacterium | 0 |
| d__Bacteria;p__Firmicutes;c__Clostridia;o__Oscillospirales;f__uncultured;g__uncultured;__ | 0 |
| d__Bacteria;p__Proteobacteria;c__Gammaproteobacteria;o__Pseudomonadales;f__Pseudomonadaceae;g__Azotobacter;s__Azotobacter_chroococcum | 0 |
| d__Bacteria;p__Proteobacteria;c__Gammaproteobacteria;o__Pseudomonadales;f__Porticoccaceae;g__C1-B045;__ | 0 |
| d__Bacteria;p__Proteobacteria;c__Gammaproteobacteria;o__EC3;f__EC3;g__EC3;s__uncultured_bacterium | 0 |
| d__Bacteria;p__Firmicutes;c__Bacilli;o__RF39;f__RF39;g__RF39;__ | 0 |
| d__Bacteria;p__Actinobacteriota;c__Actinobacteria;o__Kineosporiales;f__Kineosporiaceae;__;__ | 0 |
| d__Bacteria;p__Firmicutes;c__Clostridia;o__Oscillospirales;f__Hungateiclostridiaceae;g__Ruminiclostridium;s__Ruminiclostridium_cellulolyticum | 0 |
| d__Bacteria;p__Firmicutes;c__Bacilli;o__Staphylococcales;f__Staphylococcaceae;g__Staphylococcus;s__Staphylococcus_hominis | 0 |
| d__Bacteria;p__Proteobacteria;c__Gammaproteobacteria;o__Xanthomonadales;f__Xanthomonadaceae;g__Stenotrophomonas;s__Stenotrophomonas_maltophilia | 0 |
| d__Bacteria;p__Proteobacteria;c__Alphaproteobacteria;o__Micavibrionales;f__Micavibrionaceae;g__uncultured;__ | 0 |
| d__Bacteria;p__Proteobacteria;c__Gammaproteobacteria;o__Burkholderiales;f__Comamonadaceae;g__uncultured;__ | 0 |
| d__Bacteria;p__Proteobacteria;c__Alphaproteobacteria;o__Rhizobiales;f__Xanthobacteraceae;g__Xanthobacter;__ | 0 |
| d__Bacteria;p__Desulfobacterota;c__Desulfuromonadia;o__Geobacterales;f__Geobacteraceae;g__Citrifermentans;s__uncultured_bacterium | 0 |
| d__Bacteria;p__Chloroflexi;c__Anaerolineae;o__Anaerolineales;f__Anaerolineaceae;g__Anaerolinea;s__uncultured_bacterium | 0 |
| d__Bacteria;p__Firmicutes;c__Clostridia;o__Peptostreptococcales-Tissierellales;f__Peptostreptococcaceae;g__Paeniclostridium;s__Paeniclostridium_sordellii | 0 |
| d__Bacteria;p__Proteobacteria;c__Alphaproteobacteria;o__Rhizobiales;f__Xanthobacteraceae;g__Rhodoplanes;s__metagenome | 0 |
| d__Bacteria;p__Bacteroidota;c__Ignavibacteria;o__Ignavibacteriales;f__PHOS-HE36;g__PHOS-HE36;__ | 0 |
| d__Bacteria;p__Patescibacteria;c__Microgenomatia;o__Candidatus_Daviesbacteria;f__Candidatus_Daviesbacteria;g__Candidatus_Daviesbacteria;s__uncultured_bacterium | 0 |
| d__Bacteria;p__Chloroflexi;c__Chloroflexia;o__Chloroflexales;f__Roseiflexaceae;__;__ | 0 |
| d__Bacteria;p__Proteobacteria;c__Gammaproteobacteria;o__Burkholderiales;f__Alcaligenaceae;g__Alcaligenaceae;__ | 0 |
| d__Bacteria;p__Planctomycetota;c__Phycisphaerae;o__Phycisphaerales;f__Phycisphaeraceae;g__Urania-1B-19_marine_sediment_group;__ | 0 |
| d__Bacteria;p__Firmicutes;c__Clostridia;o__Oscillospirales;f__Ethanoligenenaceae;g__Incertae_Sedis;s__[Clostridium]_cellulosi | 0 |
| d__Bacteria;p__Planctomycetota;c__Planctomycetes;o__Pirellulales;f__Pirellulaceae;g__Rhodopirellula;__ | 0 |
| d__Bacteria;p__Desulfobacterota;c__Syntrophia;o__Syntrophales;f__uncultured;g__uncultured;s__uncultured_bacterium | 0 |
| d__Bacteria;p__Proteobacteria;c__Alphaproteobacteria;o__Ferrovibrionales;f__Ferrovibrionaceae;g__Ferrovibrio;s__uncultured_bacterium | 0 |
| d__Bacteria;p__Planctomycetota;c__Phycisphaerae;o__Phycisphaerales;f__AKAU3564_sediment_group;g__AKAU3564_sediment_group;__ | 0 |
| d__Bacteria;p__Chloroflexi;c__Dehalococcoidia;__;__;__;__ | 0 |
| d__Bacteria;p__Acidobacteriota;c__Subgroup_18;o__Subgroup_18;f__Subgroup_18;g__Subgroup_18;s__uncultured_bacterium | 0 |
| d__Bacteria;p__Firmicutes;c__Clostridia;o__Oscillospirales;f__Ruminococcaceae;g__Candidatus_Soleaferrea;s__uncultured_bacterium | 0 |
| d__Bacteria;p__Actinobacteriota;c__Actinobacteria;o__Pseudonocardiales;f__Pseudonocardiaceae;g__Pseudonocardia;s__Pseudonocardia_thermophila | 0 |
| d__Bacteria;p__Patescibacteria;c__Microgenomatia;o__Candidatus_Curtissbacteria;f__Candidatus_Curtissbacteria;g__Candidatus_Curtissbacteria;__ | 0 |
| d__Bacteria;p__Firmicutes;c__Bacilli;o__Brevibacillales;f__Brevibacillaceae;g__Brevibacillus;s__Brevibacillus_brevis | 0 |
| d__Bacteria;p__Firmicutes;c__Bacilli;o__Bacillales;f__Bacillaceae;g__Bacillus;s__Bacillus_ginsengihumi | 0 |
| d__Bacteria;p__Proteobacteria;c__Alphaproteobacteria;o__Tistrellales;f__Geminicoccaceae;g__Geminicoccus;__ | 0 |
| d__Bacteria;p__Proteobacteria;c__Gammaproteobacteria;o__Steroidobacterales;f__Steroidobacteraceae;g__Steroidobacteraceae;s__compost_metagenome | 0 |
| d__Bacteria;p__Bacteroidota;c__Bacteroidia;o__Sphingobacteriales;f__AKYH767;g__AKYH767;s__uncultured_bacterium | 0 |
| d__Bacteria;p__Proteobacteria;c__Alphaproteobacteria;o__Rhizobiales;f__Xanthobacteraceae;g__Pseudorhodoplanes;s__uncultured_Alphaproteobacteria | 0 |
| d__Bacteria;p__Bacteroidota;c__Ignavibacteria;o__SJA-28;f__SJA-28;g__SJA-28;s__uncultured_bacterium | 0 |
| d__Bacteria;p__Acidobacteriota;c__Vicinamibacteria;o__Vicinamibacterales;f__Vicinamibacteraceae;g__uncultured;s__uncultured_Acidobacteria | 0 |
| d__Bacteria;p__Planctomycetota;c__Phycisphaerae;o__Phycisphaerales;f__Phycisphaeraceae;g__CL500-3;__ | 0 |
| d__Bacteria;p__Dependentiae;c__Babeliae;o__Babeliales;f__UBA12409;g__UBA12409;__ | 0 |
| d__Bacteria;p__Proteobacteria;c__Alphaproteobacteria;o__Sphingomonadales;f__Sphingomonadaceae;g__Sphingosinicella;__ | 0 |
| d__Bacteria;p__Verrucomicrobiota;c__Verrucomicrobiae;o__Opitutales;f__Opitutaceae;g__Alterococcus;s__uncultured_bacterium | 0 |
| d__Bacteria;p__Firmicutes;c__Bacilli;o__Entomoplasmatales;f__type_III;g__type_III;s__uncultured_Mollicutes | 0 |
| d__Bacteria;p__Planctomycetota;c__Planctomycetes;o__Planctomycetales;f__Gimesiaceae;g__uncultured;s__uncultured_bacterium | 0 |
| d__Bacteria;p__Chloroflexi;c__Chloroflexia;o__Chloroflexales;f__Roseiflexaceae;g__Roseiflexus;s__uncultured_bacterium | 0 |
| d__Bacteria;p__Myxococcota;c__bacteriap25;o__bacteriap25;f__bacteriap25;g__bacteriap25;s__metagenome | 0 |
| d__Bacteria;p__Firmicutes;c__Clostridia;o__Clostridiales;f__Oxobacteraceae;g__Oxobacter;s__uncultured_compost | 0 |
| d__Bacteria;p__Proteobacteria;c__Gammaproteobacteria;o__Immundisolibacterales;f__Immundisolibacteraceae;g__Immundisolibacter;s__uncultured_bacterium | 0 |
| d__Bacteria;p__Acidobacteriota;c__Blastocatellia;o__Pyrinomonadales;f__Pyrinomonadaceae;g__RB41;s__uncultured_bacterium | 0 |
| d__Bacteria;p__Firmicutes;c__Clostridia;o__Oscillospirales;f__Hungateiclostridiaceae;g__Acetivibrio;__ | 0 |
| d__Bacteria;p__Proteobacteria;c__Gammaproteobacteria;o__Xanthomonadales;f__Rhodanobacteraceae;g__Tahibacter;s__uncultured_bacterium | 0 |
| d__Bacteria;p__Firmicutes;c__Clostridia;o__Clostridiales;f__Clostridiaceae;g__Clostridium_sensu_stricto_7;s__Clostridium_sp. | 0 |
| d__Bacteria;p__WS2;c__WS2;o__WS2;f__WS2;g__WS2;__ | 0 |
| d__Bacteria;p__Desulfobacterota;c__Desulfuromonadia;o__Geobacterales;f__Geobacteraceae;g__Citrifermentans;__ | 0 |
| d__Bacteria;p__Actinobacteriota;c__Actinobacteria;o__Frankiales;f__Geodermatophilaceae;g__Blastococcus;s__uncultured_bacterium | 0 |
| d__Bacteria;p__Spirochaetota;c__Spirochaetia;o__Spirochaetales;f__Spirochaetaceae;g__GWE2-31-10;s__uncultured_gamma | 0 |
| d__Bacteria;p__Firmicutes;c__Clostridia;o__Oscillospirales;f__Oscillospiraceae;g__uncultured;s__uncultured_bacterium | 0 |
| d__Bacteria;p__Actinobacteriota;c__Acidimicrobiia;o__Microtrichales;f__Ilumatobacteraceae;g__Ilumatobacter;__ | 0 |
| d__Bacteria;p__Chloroflexi;c__Chloroflexia;o__Chloroflexales;f__Chloroflexaceae;g__FFCH7168;s__uncultured_bacterium | 0 |
| d__Bacteria;p__Firmicutes;c__Bacilli;o__Brevibacillales;f__Brevibacillaceae;g__Brevibacillus;s__Brevibacillus_thermoruber | 0 |
| d__Bacteria;p__Planctomycetota;c__Phycisphaerae;o__S-70;f__S-70;g__S-70;s__uncultured_bacterium | 0 |
| d__Bacteria;p__Proteobacteria;c__Gammaproteobacteria;o__Acidithiobacillales;f__Acidithiobacillaceae;g__KCM-B-112;s__uncultured_bacterium | 0 |
| d__Bacteria;p__Proteobacteria;c__Gammaproteobacteria;o__Pseudomonadales;f__KI89A_clade;g__KI89A_clade;s__uncultured_bacterium | 0 |
| d__Bacteria;p__Chloroflexi;c__Chloroflexia;o__Thermobaculales;f__Thermobaculaceae;g__Thermobaculum;s__uncultured_bacterium | 0 |
| d__Bacteria;p__Firmicutes;c__Negativicutes;o__Veillonellales-Selenomonadales;f__Veillonellaceae;g__Veillonella;s__uncultured_bacterium | 0 |
| d__Bacteria;p__Dependentiae;c__Babeliae;o__Babeliales;f__Babeliales;g__Babeliales;s__uncultured_Candidatus | 0 |
| d__Bacteria;p__Proteobacteria;c__Gammaproteobacteria;o__Xanthomonadales;f__uncultured;g__uncultured;s__uncultured_bacterium | 0 |
| d__Bacteria;p__Proteobacteria;c__Alphaproteobacteria;o__Rickettsiales;f__SM2D12;g__SM2D12;s__uncultured_bacterium | 0 |
| d__Bacteria;p__Myxococcota;c__Polyangia;o__Polyangiales;f__Polyangiaceae;g__Chondromyces;__ | 0 |
| d__Bacteria;p__Chloroflexi;c__SHA-26;o__SHA-26;f__SHA-26;g__SHA-26;s__uncultured_bacterium | 0 |
| d__Bacteria;p__Acidobacteriota;c__Acidobacteriae;o__AKIW659;f__AKIW659;g__AKIW659;s__uncultured_bacterium | 0 |
| d__Bacteria;p__Proteobacteria;c__Alphaproteobacteria;o__Sphingomonadales;f__Sphingomonadaceae;g__DSSF69;s__uncultured_bacterium | 0 |
| d__Bacteria;p__Firmicutes;c__Clostridia;o__Oscillospirales;f__Oscillospiraceae;g__uncultured;__ | 0 |
| d__Bacteria;p__Proteobacteria;c__Gammaproteobacteria;o__Burkholderiales;f__Alcaligenaceae;g__Pigmentiphaga;__ | 0 |
| d__Bacteria;p__Firmicutes;c__Clostridia;o__Oscillospirales;f__Hungateiclostridiaceae;g__Pseudoclostridium;__ | 0 |
| d__Bacteria;p__Planctomycetota;c__Phycisphaerae;o__MSBL9;f__SG8-4;g__SG8-4;s__uncultured_bacterium | 0 |
| d__Bacteria;p__Firmicutes;c__Clostridia;o__Clostridiales;f__Caloramatoraceae;g__uncultured;__ | 0 |
| d__Bacteria;p__Spirochaetota;c__Leptospirae;o__Leptospirales;f__Leptospiraceae;g__RBG-16-49-21;__ | 0 |
| d__Bacteria;p__Spirochaetota;c__Leptospirae;o__Leptospirales;f__Leptospiraceae;g__Leptonema;__ | 0 |
| d__Bacteria;p__Proteobacteria;c__Gammaproteobacteria;o__Pseudomonadales;f__Porticoccaceae;__;__ | 0 |
| d__Bacteria;p__Proteobacteria;c__Alphaproteobacteria;o__Caulobacterales;f__Caulobacteraceae;g__Caulobacter;s__metagenome | 0 |
| d__Bacteria;p__Proteobacteria;c__Alphaproteobacteria;o__Caulobacterales;f__Hyphomonadaceae;g__uncultured;__ | 0 |
| d__Bacteria;p__Firmicutes;c__Thermoanaerobacteria;o__Thermoanaerobacterales;f__Thermoanaerobacteraceae;g__Brockia;s__uncultured_Symbiobacterium | 0 |
| d__Bacteria;p__Proteobacteria;c__Gammaproteobacteria;o__Xanthomonadales;f__Xanthomonadaceae;g__Pseudoxanthomonas;s__Pseudoxanthomonas_taiwanensis | 0 |
| d__Bacteria;p__Actinobacteriota;c__Acidimicrobiia;o__Microtrichales;f__Ilumatobacteraceae;__;__ | 0 |
| d__Bacteria;p__Firmicutes;c__Bacilli;o__Lactobacillales;f__Streptococcaceae;g__Lactococcus;s__Lactococcus_lactis | 0 |
| d__Bacteria;p__Patescibacteria;c__Gracilibacteria;o__Candidatus_Peribacteria;f__Candidatus_Peribacteria;g__Candidatus_Peribacteria;__ | 0 |
| d__Bacteria;p__Proteobacteria;c__Gammaproteobacteria;o__Gammaproteobacteria_Incertae_Sedis;f__Unknown_Family;g__Candidatus_Berkiella;s__Ectothiorhodospira_sp. | 0 |
| d__Bacteria;p__Spirochaetota;c__Leptospirae;o__Leptospirales;f__Leptospiraceae;__;__ | 0 |
| d__Bacteria;p__Actinobacteriota;c__Actinobacteria;o__Euzebyales;f__Euzebyaceae;g__uncultured;s__uncultured_bacterium | 0 |
| d__Bacteria;p__Verrucomicrobiota;c__Verrucomicrobiae;o__Pedosphaerales;f__Pedosphaeraceae;g__SH3-11;__ | 0 |
| d__Bacteria;p__Chloroflexi;c__Chloroflexia;o__Thermobaculales;f__Thermobaculaceae;g__Thermobaculum;__ | 0 |
| d__Bacteria;p__Patescibacteria;c__Saccharimonadia;o__Saccharimonadales;f__Saccharimonadaceae;g__TM7a;s__uncultured_bacterium | 0 |
| d__Bacteria;p__Chloroflexi;c__KD4-96;o__KD4-96;f__KD4-96;g__KD4-96;__ | 0 |
| d__Bacteria;p__Bacteroidota;c__Bacteroidia;o__Flavobacteriales;__;__;__ | 0 |
| d__Bacteria;p__Desulfobacterota;c__Desulfuromonadia;o__Geobacterales;f__Geobacteraceae;g__Geobacter;__ | 0 |
| d__Bacteria;p__Planctomycetota;c__Phycisphaerae;o__Phycisphaerales;f__Phycisphaeraceae;g__uncultured;__ | 0 |
| d__Bacteria;p__Acidobacteriota;c__Acidobacteriae;o__Solibacterales;f__Solibacteraceae;g__Candidatus_Solibacter;s__uncultured_Acidobacteria | 0 |
| d__Bacteria;p__Patescibacteria;c__Berkelbacteria;o__Berkelbacteria;f__Berkelbacteria;g__Berkelbacteria;s__uncultured_bacterium | 0 |
| d__Bacteria;p__Gemmatimonadota;c__Gemmatimonadetes;o__Gemmatimonadales;f__Gemmatimonadaceae;g__uncultured;s__uncultured_Gemmatimonadetes | 0 |
| d__Bacteria;p__Patescibacteria;c__Dojkabacteria;o__Dojkabacteria;f__Dojkabacteria;g__Dojkabacteria;s__uncultured_bacterium | 0 |
| d__Bacteria;p__Firmicutes;c__Syntrophomonadia;o__Syntrophomonadales;f__Syntrophomonadaceae;g__Syntrophomonas;__ | 0 |
| d__Bacteria;p__Myxococcota;c__Polyangia;o__PS-B29;f__PS-B29;g__PS-B29;__ | 0 |
| d__Bacteria;p__Proteobacteria;c__Alphaproteobacteria;o__Thalassobaculales;f__uncultured;g__uncultured;s__uncultured_bacterium | 0 |
| d__Bacteria;p__Proteobacteria;c__Alphaproteobacteria;o__Rhizobiales;f__Labraceae;g__Labrys;__ | 0 |
| d__Bacteria;p__Proteobacteria;c__Alphaproteobacteria;o__Rhizobiales;f__A0839;g__A0839;s__uncultured_bacterium | 0 |
| d__Bacteria;p__Armatimonadota;c__Chthonomonadetes;o__Chthonomonadales;f__Chthonomonadaceae;g__Chthonomonas;s__uncultured_bacterium | 0 |
| d__Bacteria;p__Firmicutes;c__Clostridia;o__Oscillospirales;f__Oscillospirales;g__Hydrogenoanaerobacterium;__ | 0 |
| d__Bacteria;p__Firmicutes;c__Symbiobacteriia;o__Symbiobacteriales;__;__;__ | 0 |
| d__Bacteria;p__Patescibacteria;c__Microgenomatia;o__Candidatus_Pacebacteria;f__Candidatus_Pacebacteria;g__Candidatus_Pacebacteria;s__uncultured_bacterium | 0 |
| d__Bacteria;p__Proteobacteria;c__Alphaproteobacteria;o__Rhodospirillales;f__Magnetospiraceae;g__uncultured;__ | 0 |
| d__Bacteria;p__Armatimonadota;c__Fimbriimonadia;o__Fimbriimonadales;f__Fimbriimonadales;g__Fimbriimonadales;__ | 0 |
| d__Bacteria;p__Bacteroidota;c__Bacteroidia;o__Bacteroidales;f__Prolixibacteraceae;g__uncultured;s__uncultured_bacterium | 0 |
| d__Bacteria;p__Planctomycetota;c__Planctomycetes;o__Pirellulales;f__Pirellulaceae;g__Blastopirellula;__ | 0 |
| d__Bacteria;p__Actinobacteriota;c__Acidimicrobiia;o__Microtrichales;f__Ilumatobacteraceae;g__uncultured;s__metagenome | 0 |
| d__Bacteria;p__Proteobacteria;c__Alphaproteobacteria;o__Rhizobiales;f__Rhizobiaceae;g__Mesorhizobium;s__uncultured_bacterium | 0 |
| d__Bacteria;p__Proteobacteria;c__Gammaproteobacteria;o__Burkholderiales;f__Burkholderiaceae;g__Cupriavidus;s__Cupriavidus_gilardii | 0 |
| d__Bacteria;p__Firmicutes;c__Negativicutes;o__Veillonellales-Selenomonadales;f__Sporomusaceae;g__Thermosinus;__ | 0 |
| d__Bacteria;p__Proteobacteria;c__Gammaproteobacteria;o__Steroidobacterales;f__Steroidobacteraceae;__;__ | 0 |
| d__Bacteria;p__NB1-j;c__NB1-j;o__NB1-j;f__NB1-j;g__NB1-j;__ | 0 |
| d__Bacteria;p__Bacteroidota;c__Bacteroidia;o__Bacteroidales;f__Marinilabiliaceae;g__[Cytophaga]_xylanolytica_group;__ | 0 |
| d__Bacteria;p__Patescibacteria;c__Saccharimonadia;o__Saccharimonadales;f__YM_S32_TM7_50_20;g__YM_S32_TM7_50_20;__ | 0 |
| d__Bacteria;p__Bacteroidota;c__Bacteroidia;o__Bacteroidales;f__Bacteroidetes_vadinHA17;g__Bacteroidetes_vadinHA17;s__uncultured_Prolixibacter | 0 |
| d__Bacteria;p__Acidobacteriota;c__Blastocatellia;o__Blastocatellales;f__Blastocatellaceae;g__Blastocatella;s__uncultured_bacterium | 0 |
| d__Bacteria;p__Zixibacteria;c__Zixibacteria;o__Zixibacteria;f__Zixibacteria;g__Zixibacteria;s__uncultured_bacterium | 0 |
| d__Bacteria;p__Actinobacteriota;c__Actinobacteria;o__Propionibacteriales;f__Nocardioidaceae;g__Actinopolymorpha;s__uncultured_bacterium | 0 |
| d__Bacteria;p__Firmicutes;c__Clostridia;o__Oscillospirales;f__Oscillospiraceae;g__UCG-005;s__uncultured_bacterium | 0 |
| d__Bacteria;p__Proteobacteria;c__Gammaproteobacteria;o__Burkholderiales;f__Nitrosomonadaceae;__;__ | 0 |
| d__Bacteria;p__Bacteroidota;c__Bacteroidia;o__Flavobacteriales;f__Crocinitomicaceae;g__Crocinitomix;s__uncultured_bacterium | 0 |
| d__Bacteria;p__Verrucomicrobiota;c__Verrucomicrobiae;o__Opitutales;f__Puniceicoccaceae;g__Verruc-01;__ | 0 |
| d__Bacteria;p__Firmicutes;c__Bacilli;o__Bacillales;f__Bacillaceae;g__Bacillus;s__Alicyclobacillus_sp. | 0 |
| d__Bacteria;p__Proteobacteria;c__Gammaproteobacteria;o__Methylococcales;f__Methylomonadaceae;g__Methylomicrobium;__ | 0 |
| d__Bacteria;p__Bdellovibrionota;c__Bdellovibrionia;o__Bacteriovoracales;f__Bacteriovoracaceae;g__Peredibacter;__ | 0 |
| d__Bacteria;p__Planctomycetota;c__Phycisphaerae;o__CCM11a;f__CCM11a;g__CCM11a;__ | 0 |
| d__Bacteria;p__Chloroflexi;c__Chloroflexia;o__Thermobaculales;f__Thermobaculaceae;g__Thermobaculum;s__Thermobaculum_terrenum | 0 |
| d__Bacteria;p__Bacteroidota;c__Bacteroidia;o__Cytophagales;f__Microscillaceae;__;__ | 0 |
| d__Bacteria;p__Firmicutes;c__Limnochordia;o__Limnochordales;f__Limnochordaceae;__;__ | 0 |
| d__Bacteria;p__Firmicutes;c__Clostridia;o__Peptostreptococcales-Tissierellales;f__Peptostreptococcaceae;g__Clostridioides;__ | 0 |
| d__Bacteria;p__Bacteroidota;c__Bacteroidia;o__SM1A07;f__SM1A07;g__SM1A07;s__uncultured_bacterium | 0 |
| d__Bacteria;p__Proteobacteria;c__Alphaproteobacteria;o__Elsterales;f__Elsteraceae;g__uncultured;s__uncultured_bacterium | 0 |
| d__Bacteria;p__Proteobacteria;c__Gammaproteobacteria;o__Methylococcales;f__Methylohalobiaceae;__;__ | 0 |
| d__Bacteria;p__Firmicutes;c__Clostridia;o__Oscillospirales;f__uncultured;g__uncultured;s__uncultured_bacterium | 0 |
| d__Bacteria;p__Firmicutes;c__Negativicutes;o__uncultured;f__uncultured;g__uncultured;s__uncultured_bacterium | 0 |
| d__Bacteria;p__Bacteroidota;c__Ignavibacteria;o__Ignavibacteriales;f__BSV40;g__BSV40;s__uncultured_Chlorobiales | 0 |
| d__Bacteria;p__Gemmatimonadota;c__S0134_terrestrial_group;o__S0134_terrestrial_group;f__S0134_terrestrial_group;g__S0134_terrestrial_group;s__uncultured_Gemmatimonadetes | 0 |
| d__Bacteria;p__Firmicutes;c__Thermacetogenia;o__Thermacetogeniales;f__Thermacetogeniaceae;g__Thermacetogenium;__ | 0 |
| d__Bacteria;p__Firmicutes;c__Desulfitobacteriia;o__Desulfitobacteriales;f__Heliobacteriaceae;g__uncultured;s__uncultured_bacterium | 0 |
| d__Bacteria;p__Proteobacteria;c__Alphaproteobacteria;o__Parvibaculales;f__Parvibaculaceae;__;__ | 0 |
| d__Bacteria;p__Proteobacteria;c__Alphaproteobacteria;o__Sphingomonadales;f__Sphingomonadaceae;g__Sphingobium;s__uncultured_bacterium | 0 |
| d__Bacteria;p__Bacteroidota;c__Bacteroidia;o__Chitinophagales;f__uncultured;g__uncultured;__ | 0 |
| d__Bacteria;p__Proteobacteria;c__Alphaproteobacteria;o__Rhizobiales;f__Beijerinckiaceae;g__Roseiarcus;s__uncultured_bacterium | 0 |
| d__Bacteria;p__Bacteroidota;c__Bacteroidia;o__Cytophagales;f__Cytophagaceae;g__Sporocytophaga;s__uncultured_bacterium | 0 |
| d__Bacteria;p__Planctomycetota;c__Phycisphaerae;o__Tepidisphaerales;f__CPla-3_termite_group;g__CPla-3_termite_group;__ | 0 |
| d__Bacteria;p__Proteobacteria;c__Alphaproteobacteria;o__Rhizobiales;f__Hyphomicrobiaceae;g__Hyphomicrobium;s__Hyphomicrobium_facile | 0 |
| d__Bacteria;p__Firmicutes;c__Bacilli;o__Bacillales;f__Planococcaceae;g__uncultured;__ | 0 |
| d__Bacteria;p__Fibrobacterota;c__Fibrobacteria;o__Fibrobacterales;f__Fibrobacteraceae;g__uncultured;s__uncultured_bacterium | 0 |
| d__Bacteria;p__Proteobacteria;c__Alphaproteobacteria;o__Rhizobiales;f__Beijerinckiaceae;g__Psychroglaciecola;__ | 0 |
| d__Bacteria;p__Proteobacteria;c__Alphaproteobacteria;o__Rickettsiales;f__Mitochondria;g__Mitochondria;s__Malawimonas_jakobiformis | 0 |
| d__Bacteria;p__Proteobacteria;c__Gammaproteobacteria;o__Xanthomonadales;f__Rhodanobacteraceae;g__Dyella;s__Dyella_sp. | 0 |
| d__Bacteria;p__Proteobacteria;c__Gammaproteobacteria;o__Xanthomonadales;f__Rhodanobacteraceae;g__Mizugakiibacter;s__Mizugakiibacter_sediminis | 0 |
| d__Bacteria;p__Acidobacteriota;c__Acidobacteriae;o__Acidobacteriales;f__Acidobacteriaceae_(Subgroup_1);g__Occallatibacter;s__uncultured_Acidobacteria | 0 |
| d__Bacteria;p__Firmicutes;c__Thermoanaerobacteria;o__Thermoanaerobacterales;f__SRB2;g__SRB2;s__uncultured_bacterium | 0 |
| d__Bacteria;p__Verrucomicrobiota;c__Verrucomicrobiae;o__Pedosphaerales;f__Pedosphaeraceae;g__ADurb.Bin063-1;__ | 0 |
| d__Bacteria;p__Acidobacteriota;c__Acidobacteriae;o__Acidobacteriales;f__Acidobacteriaceae_(Subgroup_1);g__Terriglobus;s__uncultured_bacterium | 0 |
| d__Bacteria;p__Actinobacteriota;c__Actinobacteria;o__Pseudonocardiales;f__Pseudonocardiaceae;g__Amycolatopsis;s__uncultured_bacterium | 0 |
| d__Bacteria;p__Proteobacteria;c__Gammaproteobacteria;o__Burkholderiales;f__Burkholderiaceae;g__Burkholderia-Caballeronia-Paraburkholderia;s__Burkholderia_ubonensis | 0 |
| d__Bacteria;p__Firmicutes;c__Bacilli;o__Bacillales;f__Bacillaceae;g__Ureibacillus;s__Ureibacillus_defluvii | 0 |
| d__Bacteria;p__Proteobacteria;c__Alphaproteobacteria;o__Acetobacterales;f__Acetobacteraceae;g__Acidisoma;__ | 0 |
| d__Bacteria;p__Proteobacteria;c__Gammaproteobacteria;o__Burkholderiales;f__Burkholderiaceae;g__Burkholderia-Caballeronia-Paraburkholderia;s__Burkholderia_thailandensis | 0 |
| d__Bacteria;p__Proteobacteria;c__Gammaproteobacteria;o__Burkholderiales;f__Neisseriaceae;g__Neisseria;__ | 0 |
| d__Bacteria;p__Proteobacteria;c__Gammaproteobacteria;o__Burkholderiales;f__Burkholderiaceae;g__Pandoraea;s__Pandoraea_pnomenusa | 0 |
| d__Bacteria;p__Proteobacteria;c__Gammaproteobacteria;o__Burkholderiales;f__Burkholderiaceae;g__Burkholderia-Caballeronia-Paraburkholderia;s__Burkholderia_pseudomallei | 0 |
| d__Bacteria;p__Planctomycetota;c__Planctomycetes;o__Isosphaerales;f__Isosphaeraceae;g__Tundrisphaera;s__uncultured_bacterium | 0 |
| d__Bacteria;p__Proteobacteria;c__Alphaproteobacteria;o__Rhizobiales;f__Beijerinckiaceae;g__Methylovirgula;s__uncultured_bacterium | 0 |
| d__Bacteria;p__Proteobacteria;c__Gammaproteobacteria;o__Burkholderiales;f__Burkholderiaceae;g__Burkholderia-Caballeronia-Paraburkholderia;s__Trinickia_symbiotica | 0 |
| d__Bacteria;p__Proteobacteria;c__Gammaproteobacteria;o__Coxiellales;f__Coxiellaceae;g__Coxiella;s__uncultured_Legionellales | 0 |
| d__Bacteria;p__Bacteroidota;c__Bacteroidia;o__Sphingobacteriales;f__Sphingobacteriaceae;g__Mucilaginibacter;s__Mucilaginibacter_sp. | 0 |
| d__Bacteria;p__Armatimonadota;c__Fimbriimonadia;o__Fimbriimonadales;f__Fimbriimonadaceae;g__Fimbriimonas;__ | 0 |
| d__Bacteria;p__Proteobacteria;c__Alphaproteobacteria;o__Caulobacterales;f__Caulobacteraceae;g__Asticcacaulis;s__uncultured_bacterium | 0 |
| d__Bacteria;p__Actinobacteriota;c__Actinobacteria;o__Pseudonocardiales;f__Pseudonocardiaceae;g__Saccharopolyspora;s__Saccharopolyspora_gregorii | 0 |
| d__Bacteria;p__Acidobacteriota;c__Acidobacteriae;o__Acidobacteriales;f__uncultured;g__uncultured;s__uncultured_Acidobacteria | 0 |
| d__Bacteria;p__Firmicutes;c__Bacilli;o__Lactobacillales;f__Carnobacteriaceae;g__Granulicatella;s__uncultured_organism | 0 |
| d__Bacteria;p__Proteobacteria;c__Alphaproteobacteria;o__Rickettsiales;f__Rickettsiaceae;g__Candidatus_Megaira;s__uncultured_bacterium | 0 |
| d__Bacteria;p__Firmicutes;c__Clostridia;o__Peptostreptococcales-Tissierellales;f__Sedimentibacteraceae;g__Sedimentibacter;s__uncultured_Sedimentibacter | 0 |
| d__Bacteria;p__Proteobacteria;c__Gammaproteobacteria;o__Gammaproteobacteria_Incertae_Sedis;f__Unknown_Family;g__Candidatus_Ovatusbacter;__ | 0 |
| d__Bacteria;p__Firmicutes;c__Clostridia;o__Peptostreptococcales-Tissierellales;f__Family_XI;g__uncultured;__ | 0 |
| d__Bacteria;p__Acidobacteriota;c__Acidobacteriae;o__Subgroup_2;f__Subgroup_2;g__Subgroup_2;s__uncultured_bacterium | 0 |
| d__Bacteria;p__Bacteroidota;c__Bacteroidia;o__Sphingobacteriales;f__CWT_CU03-E12;g__CWT_CU03-E12;__ | 0 |
| d__Bacteria;p__Actinobacteriota;c__Thermoleophilia;o__Solirubrobacterales;f__Solirubrobacteraceae;g__Solirubrobacter;s__metagenome | 0 |
| d__Bacteria;p__Firmicutes;c__Bacilli;o__Bacillales;f__Bacillaceae;g__Halobacillus;s__Halobacillus_mangrovi | 0 |
| d__Bacteria;p__Bdellovibrionota;c__Oligoflexia;o__0319-6G20;f__0319-6G20;g__0319-6G20;s__uncultured_soil | 0 |
| d__Bacteria;p__Actinobacteriota;c__Actinobacteria;o__Frankiales;f__Frankiaceae;__;__ | 0 |
| d__Bacteria;p__Bacteroidota;c__Bacteroidia;o__Sphingobacteriales;f__Sphingobacteriaceae;g__Mucilaginibacter;s__uncultured_bacterium | 0 |
| d__Bacteria;p__Planctomycetota;c__Phycisphaerae;o__Phycisphaerales;f__Phycisphaeraceae;__;__ | 0 |
| d__Bacteria;p__Verrucomicrobiota;c__Lentisphaeria;o__SS1-B-02-17;f__SS1-B-02-17;g__SS1-B-02-17;__ | 0 |
| d__Bacteria;p__Proteobacteria;c__Alphaproteobacteria;o__Rhizobiales;f__Devosiaceae;g__Devosia;s__uncultured_Alphaproteobacteria | 0 |
| d__Bacteria;p__Proteobacteria;c__Gammaproteobacteria;o__Burkholderiales;f__Burkholderiaceae;g__Burkholderia-Caballeronia-Paraburkholderia;s__Burkholderia_contaminans | 0 |
| d__Bacteria;p__Proteobacteria;c__Gammaproteobacteria;o__Burkholderiales;f__Burkholderiaceae;__;__ | 0 |
| d__Bacteria;p__Proteobacteria;c__Gammaproteobacteria;o__Xanthomonadales;f__Rhodanobacteraceae;g__Dyella;s__Dyella_thiooxydans | 0 |
| d__Bacteria;p__Firmicutes;c__Bacilli;o__Entomoplasmatales;f__Entomoplasmataceae;g__[Mycoplasma]_group;__ | 0 |
| d__Bacteria;p__Actinobacteriota;c__Actinobacteria;o__Frankiales;f__Sporichthyaceae;g__Longivirga;__ | 0 |
| d__Bacteria;p__Actinobacteriota;c__Actinobacteria;o__Streptosporangiales;f__Streptosporangiaceae;g__Sphaerisporangium;s__uncultured_bacterium | 0 |
| d__Bacteria;p__Actinobacteriota;c__Actinobacteria;o__Corynebacteriales;f__Mycobacteriaceae;g__Mycobacterium;s__Mycobacterium_avium | 0 |
| d__Bacteria;p__Proteobacteria;c__Alphaproteobacteria;o__Micropepsales;f__Micropepsaceae;g__uncultured;s__uncultured_Alphaproteobacteria | 0 |
| d__Bacteria;p__Proteobacteria;c__Alphaproteobacteria;o__Paracaedibacterales;f__Paracaedibacteraceae;g__Candidatus_Paracaedibacter;__ | 0 |
| d__Bacteria;p__Planctomycetota;c__Planctomycetes;o__Gemmatales;f__Gemmataceae;g__Gemmata;s__metagenome | 0 |
| d__Bacteria;p__Proteobacteria;c__Alphaproteobacteria;o__Rhizobiales;f__KF-JG30-B3;g__KF-JG30-B3;s__metagenome | 0 |
| d__Bacteria;p__Proteobacteria;c__Alphaproteobacteria;o__Caulobacterales;f__Caulobacteraceae;g__Asticcacaulis;s__uncultured_Asticcacaulis | 0 |
| d__Bacteria;p__Proteobacteria;c__Alphaproteobacteria;o__Rhizobiales;f__Rhodomicrobiaceae;g__Rhodomicrobium;s__uncultured_bacterium | 0 |
| d__Bacteria;p__Firmicutes;c__Clostridia;o__Clostridiales;f__Clostridiaceae;g__Clostridium_sensu_stricto_15;s__Clostridium_tetani | 0 |
| d__Bacteria;p__Proteobacteria;c__Gammaproteobacteria;o__BD72BR169;f__BD72BR169;g__BD72BR169;s__uncultured_bacterium | 0 |
| d__Bacteria;p__Proteobacteria;c__Gammaproteobacteria;o__Burkholderiales;f__Burkholderiaceae;g__Burkholderia-Caballeronia-Paraburkholderia;s__Paraburkholderia_oxyphila | 0 |
| d__Bacteria;p__Proteobacteria;c__Gammaproteobacteria;o__Burkholderiales;f__Burkholderiaceae;g__Burkholderia-Caballeronia-Paraburkholderia;s__Burkholderia_vietnamiensis | 0 |
| d__Bacteria;p__Firmicutes;c__Bacilli;o__Lactobacillales;f__Carnobacteriaceae;g__Carnobacterium;s__Carnobacterium_inhibens | 0 |
| d__Bacteria;p__Firmicutes;c__Bacilli;o__Paenibacillales;f__Paenibacillaceae;g__Paenibacillus;s__Paenibacillus_donghaensis | 0 |
| d__Bacteria;p__Bacteroidota;c__Bacteroidia;o__Chitinophagales;f__Chitinophagaceae;g__Haoranjiania;__ | 0 |
| d__Bacteria;p__Proteobacteria;c__Alphaproteobacteria;o__Puniceispirillales;f__uncultured;g__uncultured;__ | 0 |
| d__Bacteria;p__Proteobacteria;c__Alphaproteobacteria;o__Rhizobiales;f__Xanthobacteraceae;g__Afipia;s__Afipia_felis | 0 |
| d__Bacteria;p__Proteobacteria;c__Alphaproteobacteria;o__Rhodospirillales;f__Rhodospirillaceae;g__uncultured;__ | 0 |
| d__Bacteria;p__Firmicutes;c__Desulfotomaculia;o__Desulfotomaculales;f__Desulfotomaculales_Incertae_Sedis;g__uncultured;s__uncultured_bacterium | 0 |
| d__Bacteria;p__Firmicutes;c__Bacilli;o__Bacillales;f__Planococcaceae;g__Solibacillus;s__Solibacillus_silvestris | 0 |
| d__Bacteria;p__Proteobacteria;c__Gammaproteobacteria;o__Salinisphaerales;f__Solimonadaceae;g__Alkanibacter;__ | 0 |
| d__Bacteria;p__Chloroflexi;c__Chloroflexia;o__Elev-1554;f__Elev-1554;g__Elev-1554;s__uncultured_bacterium | 0 |
| d__Bacteria;p__Firmicutes;c__Syntrophomonadia;o__Syntrophomonadales;f__Syntrophomonadaceae;g__uncultured;s__uncultured_bacterium | 0 |
| d__Bacteria;p__Firmicutes;c__Bacilli;o__Bacillales;f__Bacillaceae;g__Bacillus;s__Bacillus_kochii | 0 |
| d__Bacteria;p__Proteobacteria;c__Gammaproteobacteria;o__Pseudomonadales;f__Moraxellaceae;g__Acinetobacter;__ | 0 |
| d__Bacteria;p__Proteobacteria;c__Gammaproteobacteria;o__Burkholderiales;f__TRA3-20;g__TRA3-20;s__metagenome | 0 |
| d__Bacteria;p__Actinobacteriota;c__Actinobacteria;o__Micrococcales;f__Demequinaceae;g__Demequina;__ | 0 |
| d__Bacteria;p__Proteobacteria;c__Alphaproteobacteria;o__AT-s3-44;f__AT-s3-44;g__AT-s3-44;__ | 0 |
| d__Bacteria;p__Bacteroidota;c__Bacteroidia;o__Chitinophagales;f__Chitinophagaceae;g__Dinghuibacter;s__uncultured_bacterium | 0 |
| d__Bacteria;p__Planctomycetota;c__Planctomycetes;o__Planctomycetales;f__Gimesiaceae;g__uncultured;__ | 0 |
| d__Bacteria;p__Proteobacteria;c__Alphaproteobacteria;o__Caulobacterales;f__Caulobacteraceae;g__Caulobacter;s__Caulobacter_sp. | 0 |
| d__Bacteria;p__Actinobacteriota;c__Actinobacteria;o__Streptosporangiales;f__Thermomonosporaceae;g__Thermomonospora;s__Thermomonospora_curvata | 0 |
| d__Bacteria;p__Myxococcota;c__Polyangia;o__Polyangiales;f__Polyangiaceae;__;__ | 0 |
| d__Bacteria;p__Myxococcota;c__Myxococcia;o__Myxococcales;f__Myxococcaceae;g__Archangium;__ | 0 |
| d__Bacteria;p__Desulfobacterota;c__Desulfuromonadia;o__Desulfuromonadales;__;__;__ | 0 |
| d__Bacteria;p__Proteobacteria;c__Gammaproteobacteria;o__Enterobacterales;f__Pectobacteriaceae;g__Dickeya;__ | 0 |
| d__Bacteria;p__Firmicutes;c__Bacilli;o__Lactobacillales;f__Streptococcaceae;g__Streptococcus;s__Streptococcus_thermophilus | 0 |
| d__Bacteria;p__Gemmatimonadota;c__Gemmatimonadetes;o__Gemmatimonadales;f__Gemmatimonadaceae;g__uncultured;s__uncultured_organism | 0 |
| d__Bacteria;p__Proteobacteria;c__Gammaproteobacteria;o__Methylococcales;f__Methylococcaceae;g__Methyloparacoccus;__ | 0 |
| d__Bacteria;p__Firmicutes;c__Dethiobacteria;o__Dethiobacterales;f__Dethiobacteraceae;g__uncultured;s__uncultured_bacterium | 0 |
| d__Bacteria;p__Proteobacteria;c__Alphaproteobacteria;o__Rhizobiales;f__Devosiaceae;g__uncultured;__ | 0 |
| d__Bacteria;p__Bdellovibrionota;c__Oligoflexia;o__Oligoflexales;f__Oligoflexaceae;g__Oligoflexus;__ | 0 |
| d__Bacteria;p__Proteobacteria;c__Alphaproteobacteria;o__Sphingomonadales;f__Sphingomonadaceae;g__Ellin6055;s__uncultured_proteobacterium | 0 |
| d__Bacteria;p__Myxococcota;c__Myxococcia;o__Myxococcales;f__Myxococcaceae;g__Corallococcus;s__Corallococcus_coralloides | 0 |
| d__Bacteria;p__Planctomycetota;c__OM190;o__OM190;f__OM190;g__OM190;s__uncultured_planctomycete | 0 |
| d__Bacteria;p__Proteobacteria;c__Alphaproteobacteria;o__Rickettsiales;f__SM2D12;g__SM2D12;s__metagenome | 0 |
| d__Bacteria;p__Verrucomicrobiota;c__Verrucomicrobiae;o__Chthoniobacterales;f__Terrimicrobiaceae;g__Terrimicrobium;__ | 0 |
| d__Bacteria;p__Proteobacteria;c__Alphaproteobacteria;o__Rhizobiales;f__Devosiaceae;g__uncultured;s__uncultured_Alphaproteobacteria | 0 |
| d__Bacteria;p__Desulfobacterota;c__Desulfuromonadia;o__Geobacterales;f__Geobacteraceae;g__Geobacter;s__uncultured_bacterium | 0 |
| d__Bacteria;p__Proteobacteria;c__Alphaproteobacteria;o__Azospirillales;f__Azospirillaceae;g__Azospirillum;s__Azospirillum_lipoferum | 0 |
| d__Bacteria;p__Proteobacteria;c__Gammaproteobacteria;o__Burkholderiales;f__Rhodocyclaceae;g__Azoarcus;s__uncultured_Azoarcus | 0 |
| d__Bacteria;p__Firmicutes;c__BRH-c20a;o__BRH-c20a;f__BRH-c20a;g__BRH-c20a;s__uncultured_bacterium | 0 |
| d__Bacteria;p__Proteobacteria;c__Alphaproteobacteria;o__Rhodobacterales;f__Rhodobacteraceae;g__Amaricoccus;__ | 0 |
| d__Bacteria;p__Firmicutes;c__Symbiobacteriia;o__Symbiobacteriales;f__Symbiobacteriales;g__Caldinitratiruptor;s__Caldinitratiruptor_microaerophilus | 0 |
| d__Bacteria;p__Proteobacteria;c__Alphaproteobacteria;o__Rhodobacterales;f__Rhodobacteraceae;g__Amaricoccus;s__metagenome | 0 |
| d__Bacteria;p__Proteobacteria;c__Alphaproteobacteria;o__Dongiales;f__Dongiaceae;g__Dongia;s__metagenome | 0 |
| d__Bacteria;p__Firmicutes;c__Clostridia;o__Oscillospirales;f__Ruminococcaceae;g__uncultured;s__uncultured_Clostridium | 0 |
| d__Bacteria;p__Proteobacteria;c__Gammaproteobacteria;o__Diplorickettsiales;f__Diplorickettsiaceae;g__Rickettsiella;__ | 0 |
| d__Bacteria;p__Proteobacteria;c__Alphaproteobacteria;o__Rhizobiales;f__Methylopilaceae;__;__ | 0 |
| d__Bacteria;p__Planctomycetota;c__Planctomycetes;o__Isosphaerales;f__Isosphaeraceae;g__Singulisphaera;s__metagenome | 0 |
| d__Bacteria;p__Proteobacteria;c__Alphaproteobacteria;o__Rhizobiales;f__Beijerinckiaceae;g__Methylocystis;s__Methylocystis | 0 |
| d__Bacteria;p__Elusimicrobiota;c__Elusimicrobia;o__MVP-88;f__MVP-88;g__MVP-88;s__uncultured_bacterium | 0 |
| d__Bacteria;p__Bacteroidota;c__Bacteroidia;o__Cytophagales;f__Spirosomaceae;g__Lacihabitans;s__uncultured_bacterium | 0 |
| d__Bacteria;p__Proteobacteria;c__Gammaproteobacteria;o__Ectothiorhodospirales;f__Thioalkalispiraceae;g__Thioalkalispira-Sulfurivermis;__ | 0 |
| d__Bacteria;p__Proteobacteria;c__Alphaproteobacteria;o__Rhizobiales;f__Rhizobiaceae;g__Ochrobactrum;s__Ochrobactrum_intermedium | 0 |
| d__Bacteria;p__Verrucomicrobiota;c__Verrucomicrobiae;o__Verrucomicrobiales;f__Verrucomicrobiaceae;g__uncultured;s__uncultured_bacterium | 0 |
| d__Bacteria;p__Planctomycetota;c__Planctomycetes;o__Pirellulales;f__Pirellulaceae;g__Blastopirellula;s__uncultured_bacterium | 0 |
| d__Bacteria;p__Proteobacteria;c__Alphaproteobacteria;o__Azospirillales;f__Azospirillaceae;g__Skermanella;s__Skermanella_xinjiangensis | 0 |
| d__Bacteria;p__Firmicutes;c__Clostridia;o__Oscillospirales;f__Oscillospiraceae;g__Papillibacter;s__uncultured_bacterium | 0 |
| d__Bacteria;p__Proteobacteria;c__Alphaproteobacteria;o__Parvibaculales;f__Parvibaculaceae;g__uncultured;s__uncultured_bacterium | 0 |
| d__Bacteria;p__Planctomycetota;c__Phycisphaerae;o__MSBL9;f__KCLunmb-38-53;g__KCLunmb-38-53;s__uncultured_bacterium | 0 |
| d__Bacteria;p__Proteobacteria;c__Alphaproteobacteria;o__Sphingomonadales;f__Sphingomonadaceae;g__Ellin6055;s__uncultured_bacterium | 0 |
| d__Bacteria;p__Firmicutes;c__Clostridia;o__Oscillospirales;f__[Eubacterium]_coprostanoligenes_group;g__[Eubacterium]_coprostanoligenes_group;__ | 0 |
| d__Bacteria;p__Proteobacteria;c__Alphaproteobacteria;o__Caulobacterales;f__Hyphomonadaceae;g__Hirschia;__ | 0 |
| d__Bacteria;p__Bdellovibrionota;c__Bdellovibrionia;o__Bacteriovoracales;f__Bacteriovoracaceae;g__uncultured;__ | 0 |
| d__Bacteria;p__Proteobacteria;c__Alphaproteobacteria;o__Rhizobiales;f__Xanthobacteraceae;g__Afipia;s__uncultured_Alphaproteobacteria | 0 |
| d__Bacteria;p__Proteobacteria;c__Alphaproteobacteria;o__Azospirillales;__;__;__ | 0 |
| d__Bacteria;p__Actinobacteriota;c__Actinobacteria;o__Propionibacteriales;f__Propionibacteriaceae;g__Acidipropionibacterium;s__Acidipropionibacterium_acidipropionici | 0 |
| d__Bacteria;p__Verrucomicrobiota;c__Verrucomicrobiae;o__Pedosphaerales;f__Pedosphaeraceae;g__Oikopleura;s__uncultured_bacterium | 0 |
| d__Bacteria;p__Chloroflexi;c__Anaerolineae;o__RBG-13-54-9;f__RBG-13-54-9;g__RBG-13-54-9;s__uncultured_bacterium | 0 |
| d__Bacteria;p__Firmicutes;c__Negativicutes;o__Veillonellales-Selenomonadales;f__Sporomusaceae;g__Propionispora;__ | 0 |
| d__Bacteria;p__Actinobacteriota;c__Actinobacteria;o__Streptosporangiales;f__Thermomonosporaceae;g__Thermobispora;__ | 0 |
| d__Bacteria;p__Elusimicrobiota;c__Lineage_IIb;o__Lineage_IIb;f__Lineage_IIb;g__Lineage_IIb;s__uncultured_bacterium | 0 |
| d__Bacteria;p__Chloroflexi;c__Anaerolineae;o__SBR1031;f__SBR1031;g__SBR1031;__ | 0 |
| d__Bacteria;p__Myxococcota;c__bacteriap25;o__bacteriap25;f__bacteriap25;g__bacteriap25;__ | 0 |
| d__Bacteria;p__Firmicutes;c__Clostridia;o__Peptostreptococcales-Tissierellales;f__Thermotaleaceae;g__Anaerosolibacter;__ | 0 |
| d__Bacteria;p__Myxococcota;c__Myxococcia;o__Myxococcales;f__Myxococcaceae;g__uncultured;s__uncultured_bacterium | 0 |
| d__Bacteria;p__Proteobacteria;c__Alphaproteobacteria;o__Rhizobiales;f__Pleomorphomonadaceae;g__Prosthecomicrobium;__ | 0 |
| d__Bacteria;p__Chloroflexi;c__Chloroflexia;o__Chloroflexales;f__Herpetosiphonaceae;g__Herpetosiphon;s__uncultured_bacterium | 0 |
| d__Bacteria;p__Bdellovibrionota;c__Oligoflexia;o__Silvanigrellales;f__Silvanigrellaceae;g__Silvanigrella;__ | 0 |
| d__Bacteria;p__Firmicutes;c__Desulfitobacteriia;o__Desulfitobacteriales;f__Desulfitobacteriaceae;g__Desulfosporosinus;__ | 0 |
| d__Bacteria;p__Proteobacteria;c__Alphaproteobacteria;o__Sphingomonadales;f__Sphingomonadaceae;g__Sphingomonas;s__metagenome | 0 |
| d__Bacteria;p__Bacteroidota;c__Bacteroidia;o__Chitinophagales;f__Saprospiraceae;g__Phaeodactylibacter;s__uncultured_bacterium | 0 |
| d__Bacteria;p__Desulfobacterota;c__Syntrophobacteria;o__Syntrophobacterales;f__Syntrophobacteraceae;g__Syntrophobacter;s__uncultured_bacterium | 0 |
| d__Bacteria;p__Chloroflexi;c__Chloroflexia;o__Thermomicrobiales;f__AKYG1722;g__AKYG1722;s__uncultured_bacterium | 0 |
| d__Bacteria;p__Planctomycetota;c__Planctomycetes;o__Planctomycetales;__;__;__ | 0 |
| d__Bacteria;p__Patescibacteria;c__Gracilibacteria;o__Gracilibacteria;f__Gracilibacteria;g__Gracilibacteria;s__uncultured_organism | 0 |
| d__Bacteria;p__WS2;c__WS2;o__WS2;f__WS2;g__WS2;s__uncultured_bacterium | 0 |
| d__Bacteria;p__Proteobacteria;c__Alphaproteobacteria;o__Azospirillales;f__Azospirillaceae;__;__ | 0 |
| d__Bacteria;p__Proteobacteria;c__Alphaproteobacteria;o__Rhizobiales;f__Beijerinckiaceae;g__Bosea;s__Bosea_sp. | 0 |
| d__Bacteria;p__Myxococcota;c__Polyangia;__;__;__;__ | 0 |
| d__Bacteria;p__Proteobacteria;c__Alphaproteobacteria;o__Rhizobiales;f__Beijerinckiaceae;g__uncultured;s__uncultured_bacterium | 0 |
| d__Bacteria;p__Dependentiae;c__Babeliae;o__Babeliales;f__Babeliales;g__Babeliales;__ | 0 |
| d__Bacteria;p__Firmicutes;c__Bacilli;o__Lactobacillales;f__Lactobacillaceae;g__Weissella;s__Weissella_paramesenteroides | 0 |
| d__Bacteria;p__Proteobacteria;c__Gammaproteobacteria;o__Xanthomonadales;f__Xanthomonadaceae;g__Thermomonas;s__Thermomonas_koreensis | 0 |
| d__Bacteria;p__Nitrospirota;c__Nitrospiria;o__Nitrospirales;f__Nitrospiraceae;g__Nitrospira;__ | 0 |
| d__Bacteria;p__Proteobacteria;c__Alphaproteobacteria;o__Rhizobiales;f__Pleomorphomonadaceae;g__Pleomorphomonas;__ | 0 |
| d__Bacteria;p__Planctomycetota;c__Phycisphaerae;o__Tepidisphaerales;f__Tepidisphaeraceae;g__Tepidisphaeraceae;s__uncultured_bacterium | 0 |
| d__Bacteria;p__Firmicutes;c__Bacilli;o__Bacillales;f__Planococcaceae;g__Paenisporosarcina;s__Sporosarcina_sp. | 0 |
| d__Bacteria;p__Desulfobacterota;__;__;__;__;__ | 0 |
| d__Bacteria;p__Firmicutes;c__Moorellia;o__Desulfitibacterales;f__Desulfitibacteraceae;g__Desulfitibacter;s__uncultured_Desulfitibacter | 0 |
| d__Bacteria;p__Myxococcota;c__Polyangia;o__Polyangiales;f__Phaselicystidaceae;g__Phaselicystis;s__uncultured_delta | 0 |
| d__Bacteria;p__Proteobacteria;c__Gammaproteobacteria;o__Burkholderiales;f__Alcaligenaceae;g__Achromobacter;s__Achromobacter_xylosoxidans | 0 |
| d__Bacteria;p__Patescibacteria;c__Dojkabacteria;o__Dojkabacteria;f__Dojkabacteria;g__Dojkabacteria;__ | 0 |
| d__Bacteria;p__Proteobacteria;c__Gammaproteobacteria;o__Burkholderiales;f__Burkholderiaceae;g__uncultured;__ | 0 |
| d__Bacteria;p__Proteobacteria;c__Alphaproteobacteria;o__Tistrellales;f__Geminicoccaceae;g__uncultured;s__uncultured_bacterium | 0 |
| d__Bacteria;p__Chloroflexi;c__Anaerolineae;o__SJA-15;f__SJA-15;g__SJA-15;s__uncultured_bacterium | 0 |
| d__Bacteria;p__Bdellovibrionota;c__Oligoflexia;o__Oligoflexales;f__Oligoflexaceae;g__uncultured;s__metagenome | 0 |
| d__Bacteria;p__Patescibacteria;c__Microgenomatia;o__Candidatus_Woesebacteria;f__Candidatus_Woesebacteria;g__Candidatus_Woesebacteria;s__uncultured_bacterium | 0 |
| d__Bacteria;p__Proteobacteria;c__Alphaproteobacteria;o__Rhizobiales;f__Rhizobiaceae;g__Allorhizobium-Neorhizobium-Pararhizobium-Rhizobium;s__Rhizobium_selenitireducens | 0 |
| d__Bacteria;p__Verrucomicrobiota;c__Verrucomicrobiae;o__Verrucomicrobiales;f__Rubritaleaceae;g__Luteolibacter;__ | 0 |
| d__Bacteria;p__Verrucomicrobiota;c__Verrucomicrobiae;o__Chthoniobacterales;f__Chthoniobacteraceae;__;__ | 0 |
| d__Bacteria;p__Firmicutes;c__Negativicutes;o__Veillonellales-Selenomonadales;f__Sporomusaceae;g__Thermosinus;s__Thermosinus_carboxydivorans | 0 |
| d__Bacteria;p__Bacteroidota;c__Kryptonia;o__Kryptoniales;f__MSB-3C8;g__MSB-3C8;__ | 0 |
| d__Bacteria;p__Planctomycetota;c__Phycisphaerae;o__MSBL9;f__GWC2-45-44;g__GWC2-45-44;s__uncultured_bacterium | 0 |
| d__Bacteria;p__Chloroflexi;c__Anaerolineae;o__Ardenticatenales;f__uncultured;g__uncultured;__ | 0 |
| d__Bacteria;p__Firmicutes;c__Desulfitobacteriia;o__Desulfitobacteriales;f__Desulfitobacteriaceae;g__Desulfosporosinus;s__Desulfosporosinus_meridiei | 0 |
| d__Bacteria;p__Patescibacteria;c__Gracilibacteria;o__Gracilibacteria;f__Gracilibacteria;g__Gracilibacteria;s__uncultured_bacterium | 0 |
| d__Bacteria;p__Firmicutes;c__Clostridia;o__Clostridiales;f__Clostridiaceae;g__Clostridium_sensu_stricto_12;s__Clostridium_carboxidivorans | 0 |
| d__Bacteria;p__Proteobacteria;c__Alphaproteobacteria;o__Rhizobiales;f__Rhizobiaceae;g__Nitratireductor;__ | 0 |
| d__Bacteria;p__Firmicutes;c__Bacilli;o__Bacillales;f__Planococcaceae;g__Planomicrobium;s__uncultured_bacterium | 0 |
| d__Bacteria;p__Firmicutes;c__Bacilli;o__Bacillales;f__Planococcaceae;g__Sporosarcina;s__Sporosarcina_globispora | 0 |
| d__Bacteria;p__Firmicutes;c__Clostridia;o__Oscillospirales;f__Hungateiclostridiaceae;g__Thermoclostridium;__ | 0 |
| d__Bacteria;p__Chloroflexi;c__Anaerolineae;o__Caldilineales;f__Caldilineaceae;g__uncultured;__ | 0 |
| d__Bacteria;p__Firmicutes;c__Bacilli;o__Paenibacillales;f__Paenibacillaceae;g__Paenibacillus;s__Paenibacillus_stellifer | 0 |
| d__Bacteria;p__Proteobacteria;c__Gammaproteobacteria;o__Burkholderiales;f__Oxalobacteraceae;g__Undibacterium;__ | 0 |
| d__Bacteria;p__Actinobacteriota;c__Actinobacteria;o__Micrococcales;f__Microbacteriaceae;g__Galbitalea;__ | 0 |
| d__Bacteria;p__Planctomycetota;c__Phycisphaerae;o__mle1-8;f__mle1-8;g__mle1-8;s__uncultured_Schlesneria | 0 |
| d__Bacteria;p__Armatimonadota;c__uncultured;o__uncultured;f__uncultured;g__uncultured;__ | 0 |
| d__Bacteria;p__Planctomycetota;c__Planctomycetes;o__Planctomycetales;f__Rubinisphaeraceae;g__SH-PL14;s__uncultured_planctomycete | 0 |
| d__Bacteria;p__Latescibacterota;c__Latescibacteria;o__Latescibacterales;f__Latescibacteraceae;g__Candidatus_Latescibacter;__ | 0 |
| d__Bacteria;p__Proteobacteria;c__Alphaproteobacteria;o__Acetobacterales;f__Acetobacteraceae;g__Craurococcus-Caldovatus;s__uncultured_bacterium | 0 |
| d__Bacteria;p__Proteobacteria;c__Gammaproteobacteria;o__Burkholderiales;f__Nitrosomonadaceae;g__mle1-7;s__uncultured_bacterium | 0 |
| d__Bacteria;p__Proteobacteria;c__Alphaproteobacteria;o__Caulobacterales;f__Caulobacteraceae;g__Phenylobacterium;s__uncultured_Phenylobacterium | 0 |
| d__Bacteria;p__Firmicutes;c__Bacilli;o__Bacillales;f__Planococcaceae;g__Planomicrobium;__ | 0 |
| d__Bacteria;p__Bacteroidota;c__Bacteroidia;o__Flavobacteriales;f__NS9_marine_group;g__NS9_marine_group;s__uncultured_bacterium | 0 |
| d__Bacteria;p__Myxococcota;c__Polyangia;o__mle1-27;f__mle1-27;g__mle1-27;s__uncultured_bacterium | 0 |
| d__Bacteria;p__Myxococcota;c__Polyangia;o__Polyangiales;f__Polyangiaceae;g__Pajaroellobacter;s__uncultured_bacterium | 0 |
| d__Bacteria;p__Firmicutes;c__Clostridia;o__Clostridia_vadinBB60_group;f__Clostridia_vadinBB60_group;g__Clostridia_vadinBB60_group;__ | 0 |
| d__Bacteria;p__Proteobacteria;c__Alphaproteobacteria;o__Rickettsiales;f__Rickettsiaceae;g__uncultured;s__uncultured_bacterium | 0 |
| d__Bacteria;p__Proteobacteria;c__Alphaproteobacteria;o__Sphingomonadales;f__Sphingomonadaceae;g__Sphingoaurantiacus;__ | 0 |
| d__Bacteria;p__Patescibacteria;c__Microgenomatia;o__Candidatus_Collierbacteria;f__Candidatus_Collierbacteria;g__Candidatus_Collierbacteria;__ | 0 |
| d__Bacteria;p__Proteobacteria;c__Gammaproteobacteria;o__Enterobacterales;f__Morganellaceae;__;__ | 0 |
| d__Bacteria;p__Firmicutes;c__Bacilli;o__Alicyclobacillales;f__Alicyclobacillaceae;g__Alicyclobacillus;s__Alicyclobacillus_acidocaldarius | 0 |
| d__Bacteria;p__Planctomycetota;c__Phycisphaerae;o__Phycisphaerales;f__AKAU3564_sediment_group;g__AKAU3564_sediment_group;s__uncultured_organism | 0 |
| d__Bacteria;p__Firmicutes;c__Clostridia;o__Oscillospirales;f__Ruminococcaceae;g__Anaerotruncus;s__uncultured_compost | 0 |
| d__Bacteria;p__Planctomycetota;c__Phycisphaerae;o__MSBL9;__;__;__ | 0 |
| d__Bacteria;p__Actinobacteriota;c__Actinobacteria;o__Micrococcales;f__Micrococcaceae;g__Citricoccus;__ | 0 |
| d__Bacteria;p__Desulfobacterota;c__Syntrophobacteria;o__Syntrophobacterales;f__Syntrophobacteraceae;g__Syntrophobacter;__ | 0 |
| d__Bacteria;p__Acidobacteriota;c__Vicinamibacteria;o__Subgroup_17;f__Subgroup_17;g__Subgroup_17;s__uncultured_bacterium | 0 |
| d__Bacteria;p__WS1;c__WS1;o__WS1;f__WS1;g__WS1;s__uncultured_bacterium | 0 |
| d__Bacteria;p__Proteobacteria;c__Alphaproteobacteria;o__Rhizobiales;f__Hyphomicrobiaceae;g__Filomicrobium;__ | 0 |
| d__Bacteria;p__Acidobacteriota;c__Subgroup_21;o__Subgroup_21;f__Subgroup_21;g__Subgroup_21;s__uncultured_bacterium | 0 |
| d__Bacteria;p__Firmicutes;c__Bacilli;o__Bacillales;f__Planococcaceae;g__Jeotgalibacillus;s__Jeotgalibacillus_malaysiensis | 0 |
| d__Bacteria;p__Proteobacteria;c__Gammaproteobacteria;o__CHAB-XI-27;f__CHAB-XI-27;g__CHAB-XI-27;s__uncultured_bacterium | 0 |
| d__Bacteria;p__Verrucomicrobiota;c__Chlamydiae;o__LD1-PA32;f__LD1-PA32;g__LD1-PA32;__ | 0 |
| d__Bacteria;p__Firmicutes;c__Clostridia;o__Oscillospirales;f__Oscillospiraceae;g__Colidextribacter;__ | 0 |
| d__Bacteria;p__Actinobacteriota;c__Actinobacteria;o__Micromonosporales;f__Micromonosporaceae;g__Luedemannella;s__uncultured_bacterium | 0 |
| d__Bacteria;p__Firmicutes;c__Negativicutes;o__Veillonellales-Selenomonadales;f__Sporomusaceae;g__Sporomusa;__ | 0 |
| d__Bacteria;p__Proteobacteria;c__Alphaproteobacteria;o__Azospirillales;f__Azospirillaceae;g__Desertibacter;s__Desertibacter_roseus | 0 |
| d__Bacteria;p__Chloroflexi;c__SHA-26;o__SHA-26;f__SHA-26;g__SHA-26;__ | 0 |
| d__Bacteria;p__Elusimicrobiota;c__Lineage_IIa;o__Lineage_IIa;f__Lineage_IIa;g__Lineage_IIa;__ | 0 |
| d__Bacteria;p__Proteobacteria;c__Alphaproteobacteria;o__Rhizobiales;f__Rhizobiaceae;g__Allorhizobium-Neorhizobium-Pararhizobium-Rhizobium;s__uncultured_Rhizobium | 0 |
| d__Bacteria;p__Proteobacteria;c__Gammaproteobacteria;o__Chromatiales;f__Chromatiaceae;g__uncultured;s__uncultured_bacterium | 0 |
| d__Bacteria;p__Verrucomicrobiota;c__Omnitrophia;o__Omnitrophales;f__Omnitrophaceae;g__Candidatus_Omnitrophus;s__uncultured_bacterium | 0 |
| d__Bacteria;p__Proteobacteria;c__Alphaproteobacteria;o__Rhizobiales;f__Rhizobiaceae;g__Allorhizobium-Neorhizobium-Pararhizobium-Rhizobium;s__Rhizobium_etli | 0 |
| d__Bacteria;p__Proteobacteria;c__Gammaproteobacteria;o__Burkholderiales;f__Comamonadaceae;g__Azohydromonas;s__uncultured_bacterium | 0 |
| d__Bacteria;p__Firmicutes;c__Bacilli;o__Paenibacillales;f__Paenibacillaceae;g__Paenibacillus;s__Paenibacillus_kobensis | 0 |
| d__Bacteria;p__Actinobacteriota;c__Thermoleophilia;o__Solirubrobacterales;f__Solirubrobacteraceae;g__uncultured;s__uncultured_bacterium | 0 |
| d__Bacteria;p__Firmicutes;c__Bacilli;o__Bacillales;f__Planococcaceae;g__Lysinibacillus;s__Lysinibacillus_sp. | 0 |
| d__Bacteria;p__Proteobacteria;c__Alphaproteobacteria;o__Azospirillales;f__Azospirillales_Incertae_Sedis;g__Stella;__ | 0 |
| d__Bacteria;p__Proteobacteria;c__Alphaproteobacteria;o__Sphingomonadales;f__Sphingomonadaceae;g__Novosphingobium;s__Novosphingobium_sp. | 0 |
| d__Bacteria;p__Proteobacteria;c__Gammaproteobacteria;o__Ectothiorhodospirales;f__Ectothiorhodospiraceae;g__uncultured;s__uncultured_bacterium | 0 |
| d__Bacteria;p__Firmicutes;c__Clostridia;o__Clostridiales;f__Clostridiaceae;g__Haloimpatiens;__ | 0 |
| d__Bacteria;p__Cyanobacteria;c__Vampirivibrionia;o__Gastranaerophilales;f__Gastranaerophilales;g__Gastranaerophilales;s__uncultured_organism | 0 |
| d__Bacteria;p__Proteobacteria;c__Gammaproteobacteria;o__Nitrosococcales;f__Nitrosococcaceae;g__wb1-P19;__ | 0 |
| d__Bacteria;p__Actinobacteriota;c__Actinobacteria;o__Corynebacteriales;f__Mycobacteriaceae;g__Mycobacterium;s__Mycobacterium_neoaurum | 0 |
| d__Bacteria;p__Proteobacteria;c__Alphaproteobacteria;o__Rhodospirillales;f__Magnetospirillaceae;g__Magnetospirillum;__ | 0 |
| d__Bacteria;p__Actinobacteriota;c__Rubrobacteria;o__Rubrobacterales;f__Rubrobacteriaceae;g__Rubrobacter;__ | 0 |
| d__Bacteria;p__Proteobacteria;c__Alphaproteobacteria;o__Azospirillales;f__Azospirillaceae;g__Azospirillum;s__Azospirillum_sp. | 0 |
| d__Bacteria;p__FCPU426;c__FCPU426;o__FCPU426;f__FCPU426;g__FCPU426;__ | 0 |
| d__Bacteria;p__Proteobacteria;c__Gammaproteobacteria;o__Burkholderiales;f__Comamonadaceae;g__Comamonas;s__uncultured_bacterium | 0 |
| d__Bacteria;p__Bacteroidota;c__Ignavibacteria;o__Ignavibacteriales;f__PHOS-HE36;g__PHOS-HE36;s__uncultured_bacterium | 0 |
| d__Bacteria;p__Proteobacteria;c__Alphaproteobacteria;o__Azospirillales;f__Azospirillaceae;g__Azospirillum;s__Azospirillum_brasilense | 0 |
| d__Bacteria;p__Acidobacteriota;c__Acidobacteriae;o__Elev-16S-1166;f__Elev-16S-1166;g__Elev-16S-1166;__ | 0 |
| d__Bacteria;p__Firmicutes;c__Desulfotomaculia;o__Desulfotomaculales;f__Pelotomaculaceae;g__Pelotomaculum;s__uncultured_Pelotomaculum | 0 |
| d__Bacteria;p__Acidobacteriota;c__Acidobacteriae;o__Acidobacteriae;f__Acidobacteriae;g__Paludibaculum;s__uncultured_bacterium | 0 |
| d__Bacteria;p__Verrucomicrobiota;c__Verrucomicrobiae;o__Chthoniobacterales;__;__;__ | 0 |
| d__Bacteria;p__Proteobacteria;c__Alphaproteobacteria;o__Rhizobiales;f__Rhizobiales_Incertae_Sedis;g__uncultured;__ | 0 |
| d__Bacteria;p__Planctomycetota;c__Phycisphaerae;o__MSBL9;f__KCLunmb-38-53;g__KCLunmb-38-53;__ | 0 |
| d__Bacteria;p__Chloroflexi;c__Ktedonobacteria;o__Ktedonobacterales;f__Ktedonobacteraceae;g__1921-3;s__uncultured_Thermosporotrichaceae | 0 |
| d__Bacteria;p__SAR324_clade(Marine_group_B);c__SAR324_clade(Marine_group_B);o__SAR324_clade(Marine_group_B);f__SAR324_clade(Marine_group_B);g__SAR324_clade(Marine_group_B);s__uncultured_proteobacterium | 0 |
| d__Bacteria;p__Firmicutes;c__D8A-2;o__D8A-2;f__D8A-2;g__D8A-2;__ | 0 |
| d__Bacteria;p__Fibrobacterota;c__Fibrobacteria;o__Fibrobacterales;f__Fibrobacteraceae;g__possible_genus_04;s__uncultured_bacterium | 0 |
| d__Bacteria;p__Proteobacteria;c__Gammaproteobacteria;o__Burkholderiales;f__Comamonadaceae;g__Azohydromonas;s__Azohydromonas_australica | 0 |
| d__Bacteria;p__Verrucomicrobiota;c__Verrucomicrobiae;o__Chthoniobacterales;f__Chthoniobacteraceae;g__Candidatus_Udaeobacter;s__uncultured_bacterium | 0 |
| d__Bacteria;p__Chloroflexi;c__Chloroflexia;o__Thermomicrobiales;f__JG30-KF-CM45;g__JG30-KF-CM45;s__metagenome | 0 |
| d__Bacteria;p__Proteobacteria;c__Alphaproteobacteria;o__Rickettsiales;f__AB1;g__AB1;s__uncultured_bacterium | 0 |
| d__Bacteria;p__Firmicutes;c__Clostridia;o__Oscillospirales;f__Butyricicoccaceae;g__Butyricicoccus;__ | 0 |
| d__Bacteria;p__Nitrospirota;c__Nitrospiria;o__Nitrospirales;f__Nitrospiraceae;g__Nitrospira;s__uncultured_bacterium | 0 |
| d__Bacteria;p__Actinobacteriota;c__Actinobacteria;o__Micromonosporales;f__Micromonosporaceae;g__Virgisporangium;__ | 0 |
| d__Bacteria;p__Desulfobacterota;c__Desulfuromonadia;o__Desulfuromonadales;f__Desulfuromonadaceae;__;__ | 0 |
| d__Bacteria;p__Firmicutes;c__Bacilli;o__Bacillales;f__Planococcaceae;g__Lysinibacillus;s__Bacillus_sp. | 0 |
| d__Bacteria;p__Bacteroidota;c__Bacteroidia;o__Chitinophagales;f__Chitinophagaceae;g__Chitinophaga;s__Chitinophaga_sp. | 0 |
| d__Bacteria;p__Proteobacteria;c__Gammaproteobacteria;o__Enterobacterales;__;__;__ | 0 |
| d__Bacteria;p__Myxococcota;c__Myxococcia;o__Myxococcales;f__27F-1492R;g__27F-1492R;s__uncultured_bacterium | 0 |
| d__Bacteria;p__Proteobacteria;c__Alphaproteobacteria;o__Azospirillales;f__uncultured;g__uncultured;__ | 0 |
| d__Bacteria;p__Chloroflexi;c__TK10;o__TK10;f__TK10;g__TK10;s__metagenome | 0 |
| d__Bacteria;p__Firmicutes;c__Bacilli;o__Bacillales;f__Bacillaceae;g__Bacillus;s__Bacillus_asahii | 0 |
| d__Bacteria;p__Firmicutes;c__Bacilli;o__Bacillales;f__Bacillaceae;g__Geobacillus;s__Geobacillus_genomosp. | 0 |
| d__Bacteria;p__Proteobacteria;c__Alphaproteobacteria;o__Rhizobiales;f__Hyphomicrobiaceae;g__Filomicrobium;s__uncultured_compost | 0 |
| d__Bacteria;p__Fibrobacterota;c__Fibrobacteria;o__Fibrobacterales;__;__;__ | 0 |
| d__Bacteria;p__Proteobacteria;c__Alphaproteobacteria;o__Rhizobiales;f__Rhizobiaceae;g__Neorhizobium;__ | 0 |
| d__Bacteria;p__Firmicutes;c__Bacilli;o__Kyrpidiales;f__Kyrpidiaceae;g__Kyrpidia;s__Kyrpidia_tusciae | 0 |
| d__Bacteria;p__Myxococcota;c__Myxococcia;o__Myxococcales;f__Myxococcaceae;g__KD3-10;__ | 0 |
| d__Bacteria;p__Firmicutes;c__Bacilli;o__Bacillales;f__Planococcaceae;g__Lysinibacillus;s__Lysinibacillus_sphaericus | 0 |
| d__Bacteria;p__Proteobacteria;c__Alphaproteobacteria;o__Azospirillales;f__Azospirillaceae;g__uncultured;s__metagenome | 0 |
| d__Bacteria;p__Firmicutes;c__Desulfitobacteriia;o__Desulfitobacteriales;f__Desulfitobacteriaceae;__;__ | 0 |
| d__Bacteria;p__Proteobacteria;c__Alphaproteobacteria;o__Micavibrionales;f__uncultured;g__uncultured;s__metagenome | 0 |
| d__Bacteria;p__Proteobacteria;c__Gammaproteobacteria;o__JTB23;f__JTB23;g__JTB23;s__uncultured_bacterium | 0 |
| d__Bacteria;p__Gemmatimonadota;c__S0134_terrestrial_group;o__S0134_terrestrial_group;f__S0134_terrestrial_group;g__S0134_terrestrial_group;__ | 0 |
| d__Bacteria;p__Proteobacteria;c__Alphaproteobacteria;o__Rhizobiales;f__Beijerinckiaceae;g__Microvirga;s__uncultured_Microvirga | 0 |
| d__Bacteria;p__Bacteroidota;c__Bacteroidia;o__Chitinophagales;f__Chitinophagaceae;g__Pseudoflavitalea;s__uncultured_bacterium | 0 |
| d__Bacteria;p__Planctomycetota;c__Phycisphaerae;o__Tepidisphaerales;f__Tepidisphaeraceae;g__Tepidisphaera;__ | 0 |
| d__Bacteria;p__Actinobacteriota;c__Actinobacteria;o__Micrococcales;f__Cellulomonadaceae;__;__ | 0 |
| d__Bacteria;p__Myxococcota;c__Polyangia;o__UASB-TL25;f__UASB-TL25;g__UASB-TL25;__ | 0 |
| d__Bacteria;p__Proteobacteria;c__Alphaproteobacteria;o__Rhizobiales;f__Rhizobiaceae;g__Allorhizobium-Neorhizobium-Pararhizobium-Rhizobium;s__Rhizobium_flavum | 0 |
| d__Bacteria;p__Proteobacteria;c__Alphaproteobacteria;o__Rhizobiales;f__Rhizobiaceae;g__Allorhizobium-Neorhizobium-Pararhizobium-Rhizobium;s__Rhizobium_leguminosarum | 0 |
| d__Bacteria;p__Bacteroidota;c__Bacteroidia;o__Sphingobacteriales;f__NS11-12_marine_group;g__NS11-12_marine_group;s__metagenome | 0 |
| d__Bacteria;p__Actinobacteriota;c__Acidimicrobiia;__;__;__;__ | 0 |
| d__Bacteria;p__Firmicutes;c__Desulfotomaculia;o__Desulfotomaculales;f__Desulfotomaculales_Incertae_Sedis;g__Desulfallas-Sporotomaculum;s__Desulfallas_gibsoniae | 0 |
| d__Bacteria;p__Cyanobacteria;c__Vampirivibrionia;o__Vampirovibrionales;f__Vampirovibrionales;g__Vampirovibrionales;__ | 0 |
| d__Bacteria;p__Patescibacteria;c__Microgenomatia;o__Candidatus_Chisholmbacteria;f__Candidatus_Chisholmbacteria;g__Candidatus_Chisholmbacteria;s__uncultured_bacterium | 0 |
| d__Bacteria;p__Acidobacteriota;c__Blastocatellia;o__Pyrinomonadales;f__Pyrinomonadaceae;g__Pyrinomonas;s__uncultured_bacterium | 0 |
| d__Bacteria;p__Actinobacteriota;c__Coriobacteriia;o__OPB41;f__OPB41;g__OPB41;__ | 0 |
| d__Bacteria;p__Firmicutes;c__Moorellia;o__Moorellales;f__Moorellaceae;g__Moorella;__ | 0 |
| d__Bacteria;p__Bacteroidota;c__Bacteroidia;o__Bacteroidales;f__Prolixibacteraceae;g__Roseimarinus;s__uncultured_bacterium | 0 |
| d__Bacteria;p__Firmicutes;c__Negativicutes;o__Veillonellales-Selenomonadales;f__Sporomusaceae;g__Sporomusa;s__Sporomusa_sphaeroides | 0 |
| d__Bacteria;p__Proteobacteria;c__Gammaproteobacteria;o__Pseudomonadales;f__Moraxellaceae;g__Acinetobacter;s__Acinetobacter_johnsonii | 0 |
| d__Bacteria;p__Firmicutes;c__Desulfotomaculia;o__Desulfotomaculales;f__Pelotomaculaceae;g__Cryptanaerobacter;__ | 0 |
| d__Bacteria;p__Verrucomicrobiota;c__Verrucomicrobiae;o__Verrucomicrobiales;f__Verrucomicrobiaceae;g__uncultured;__ | 0 |
| d__Bacteria;p__Bacteroidota;c__Bacteroidia;o__Chitinophagales;f__uncultured;g__uncultured;s__uncultured_bacterium | 0 |
| d__Bacteria;p__Acidobacteriota;c__Vicinamibacteria;o__Vicinamibacterales;f__Vicinamibacteraceae;g__Vicinamibacter;s__uncultured_bacterium | 0 |
| d__Bacteria;p__Firmicutes;c__Bacilli;o__Bacillales;f__Sporolactobacillaceae;g__Tuberibacillus;s__Pullulanibacillus_sp. | 0 |
| d__Bacteria;p__Planctomycetota;c__Phycisphaerae;o__C86;f__C86;g__C86;s__uncultured_bacterium | 0 |
| d__Bacteria;p__Firmicutes;c__Bacilli;o__Paenibacillales;f__Paenibacillaceae;g__Paenibacillus;s__Paenibacillus_curdlanolyticus | 0 |
| d__Bacteria;p__Planctomycetota;c__Phycisphaerae;o__Phycisphaerales;f__AKAU3564_sediment_group;g__AKAU3564_sediment_group;s__uncultured_bacterium | 0 |
| d__Bacteria;p__Firmicutes;c__Desulfotomaculia;o__Desulfotomaculales;f__Pelotomaculaceae;g__Pelotomaculum;s__uncultured_bacterium | 0 |
| d__Bacteria;p__Proteobacteria;c__Gammaproteobacteria;o__Ga0077536;f__Ga0077536;g__Ga0077536;__ | 0 |
| d__Bacteria;p__Firmicutes;c__Bacilli;o__Bacillales;f__Bacillaceae;g__Bacillus;s__Bacillus_badius | 0 |
| d__Bacteria;p__Firmicutes;c__Clostridia;o__Lachnospirales;f__Lachnospiraceae;g__Lachnoclostridium;s__[Clostridium]_saccharolyticum | 0 |
| d__Bacteria;p__Actinobacteriota;c__Actinobacteria;o__Micrococcales;f__Microbacteriaceae;g__Microbacterium;s__Microbacterium_immunditiarum | 0 |
| d__Bacteria;p__Methylomirabilota;c__Methylomirabilia;o__Rokubacteriales;f__WX65;g__WX65;s__uncultured_bacterium | 0 |
| d__Bacteria;p__Proteobacteria;c__Alphaproteobacteria;o__Sphingomonadales;f__Sphingomonadaceae;g__Sphingomonas;s__uncultured_Sphingomonas | 0 |
| d__Bacteria;p__Proteobacteria;c__Alphaproteobacteria;o__Rhizobiales;f__Beijerinckiaceae;g__Methylobacterium-Methylorubrum;__ | 0 |
| d__Bacteria;p__Proteobacteria;c__Gammaproteobacteria;o__Burkholderiales;f__Hydrogenophilaceae;g__uncultured;__ | 0 |
| d__Bacteria;p__Firmicutes;c__Clostridia;o__Clostridiales;f__Clostridiaceae;g__Clostridium_sensu_stricto_9;s__uncultured_bacterium | 0 |
| d__Bacteria;p__Planctomycetota;c__Planctomycetes;o__uncultured;f__uncultured;g__uncultured;__ | 0 |
| d__Bacteria;p__Proteobacteria;c__Alphaproteobacteria;o__Tistrellales;f__Geminicoccaceae;g__Candidatus_Alysiosphaera;s__uncultured_bacterium | 0 |
| d__Bacteria;p__Armatimonadota;c__uncultured;o__uncultured;f__uncultured;g__uncultured;s__candidate_division | 0 |
| d__Bacteria;p__Desulfobacterota;c__Desulfovibrionia;o__Desulfovibrionales;f__Desulfovibrionaceae;g__Desulfovibrio;__ | 0 |
| d__Bacteria;p__Bacteroidota;c__Bacteroidia;o__Cytophagales;f__Cyclobacteriaceae;__;__ | 0 |
| d__Bacteria;p__Firmicutes;c__Desulfitobacteriia;o__Desulfitobacteriales;f__Heliobacteriaceae;g__Heliobacteriaceae;s__uncultured_Firmicutes | 0 |
| d__Bacteria;p__Proteobacteria;c__Alphaproteobacteria;o__Rhizobiales;f__Beijerinckiaceae;g__Microvirga;s__Microvirga_sp. | 0 |
| d__Bacteria;p__Actinobacteriota;c__Actinobacteria;o__Frankiales;f__Geodermatophilaceae;g__Blastococcus;__ | 0 |
| d__Bacteria;p__Fibrobacterota;c__Fibrobacteria;o__Fibrobacterales;f__Fibrobacteraceae;g__uncultured;__ | 0 |
| d__Bacteria;p__Proteobacteria;c__Gammaproteobacteria;o__Xanthomonadales;f__Rhodanobacteraceae;g__Frateuria;__ | 0 |
| d__Bacteria;p__Firmicutes;c__Clostridia;o__Oscillospirales;f__Ethanoligenenaceae;g__Incertae_Sedis;s__uncultured_Ruminococcaceae | 0 |
| d__Bacteria;p__Acidobacteriota;c__Acidobacteriae;o__Acidobacteriales;f__Acidobacteriaceae_(Subgroup_1);g__Acidobacteriaceae_(Subgroup_1);s__uncultured_bacterium | 0 |
| d__Bacteria;p__Proteobacteria;c__Alphaproteobacteria;o__Rhodobacterales;f__Rhodobacteraceae;g__Rubellimicrobium;s__uncultured_bacterium | 0 |
| d__Bacteria;p__Bacteroidota;c__Bacteroidia;o__Chitinophagales;f__Chitinophagaceae;g__Arachidicoccus;s__uncultured_bacterium | 0 |
| d__Bacteria;p__Firmicutes;c__Clostridia;o__Peptostreptococcales-Tissierellales;f__Caminicellaceae;g__Caminicella;s__uncultured_bacterium | 0 |
| d__Bacteria;p__Proteobacteria;c__Gammaproteobacteria;o__Burkholderiales;f__Burkholderiaceae;g__Burkholderia-Caballeronia-Paraburkholderia;s__Paraburkholderia_xenovorans | 0 |
| d__Bacteria;p__Proteobacteria;c__Gammaproteobacteria;o__Burkholderiales;f__Comamonadaceae;g__uncultured;s__metagenome | 0 |
| d__Bacteria;p__Proteobacteria;c__Alphaproteobacteria;o__Caulobacterales;f__Caulobacteraceae;g__uncultured;s__uncultured_Caulobacter | 0 |
| d__Bacteria;p__Actinobacteriota;c__Acidimicrobiia;o__IMCC26256;f__IMCC26256;g__IMCC26256;s__Aciditerrimonas_sp. | 0 |
| d__Bacteria;p__Proteobacteria;c__Gammaproteobacteria;o__Burkholderiales;f__Burkholderiaceae;g__Burkholderia-Caballeronia-Paraburkholderia;s__Paraburkholderia_caribensis | 0 |
| d__Bacteria;p__Proteobacteria;c__Gammaproteobacteria;o__Pseudomonadales;f__Moraxellaceae;g__Acinetobacter;s__Acinetobacter_pittii | 0 |
| d__Bacteria;p__Cyanobacteria;c__Cyanobacteriia;o__Leptolyngbyales;f__Leptolyngbyaceae;__;__ | 0 |
| d__Bacteria;p__Proteobacteria;c__Gammaproteobacteria;o__Xanthomonadales;f__Rhodanobacteraceae;g__Chujaibacter;s__uncultured_gamma | 0 |
| d__Bacteria;p__Firmicutes;c__Bacilli;o__Staphylococcales;f__Gemellaceae;g__Gemella;__ | 0 |
| d__Bacteria;p__Firmicutes;c__Clostridia;o__Peptostreptococcales-Tissierellales;f__Anaerovoracaceae;__;__ | 0 |
| d__Bacteria;p__Proteobacteria;c__Alphaproteobacteria;o__Puniceispirillales;f__EF100-94H03;g__EF100-94H03;s__uncultured_bacterium | 0 |
| d__Bacteria;p__Proteobacteria;c__Alphaproteobacteria;o__Paracaedibacterales;f__Paracaedibacteraceae;g__Candidatus_Finniella;__ | 0 |
| d__Bacteria;p__Proteobacteria;c__Gammaproteobacteria;o__Enterobacterales;f__Enterobacteriaceae;g__Klebsiella;s__Klebsiella_variicola | 0 |
| d__Bacteria;p__Proteobacteria;c__Alphaproteobacteria;o__Rhodobacterales;f__Rhodobacteraceae;g__Tabrizicola;s__uncultured_bacterium | 0 |
| d__Bacteria;p__Fibrobacterota;c__Fibrobacteria;o__Fibrobacterales;f__Fibrobacteraceae;g__uncultured;s__metagenome | 0 |
| d__Bacteria;p__Proteobacteria;c__Gammaproteobacteria;o__Enterobacterales;f__Pasteurellaceae;g__Haemophilus;__ | 0 |
| d__Bacteria;p__Proteobacteria;c__Alphaproteobacteria;o__Rhodobacterales;f__Rhodobacteraceae;g__Defluviimonas;s__Defluviimonas_indica | 0 |
| d__Bacteria;p__Acidobacteriota;c__Acidobacteriae;o__Acidobacteriales;f__Acidobacteriaceae_(Subgroup_1);g__Acidobacterium;s__Acidobacterium_capsulatum | 0 |
| d__Bacteria;p__Proteobacteria;c__Gammaproteobacteria;o__Nitrosococcales;f__Nitrosococcaceae;g__Nitrosococcus;__ | 0 |
| d__Bacteria;p__Actinobacteriota;c__Actinobacteria;o__Micromonosporales;f__Micromonosporaceae;g__Longispora;__ | 0 |
| d__Bacteria;p__Firmicutes;c__Bacilli;o__Bacillales;f__Bacillaceae;g__Ureibacillus;s__Ureibacillus_terrenus | 0 |
| d__Bacteria;p__RCP2-54;c__RCP2-54;o__RCP2-54;f__RCP2-54;g__RCP2-54;s__uncultured_bacterium | 0 |
| d__Bacteria;p__Planctomycetota;c__Planctomycetes;o__Gemmatales;f__Gemmataceae;g__uncultured;s__metagenome | 0 |
| d__Bacteria;p__Cyanobacteria;c__Cyanobacteriia;o__Chloroplast;f__Chloroplast;g__Chloroplast;s__Cynomorium_songaricum | 0 |
| d__Bacteria;p__Deinococcota;c__Deinococci;o__Deinococcales;f__Deinococcaceae;g__Deinococcus;__ | 0 |
| d__Bacteria;p__Firmicutes;c__Desulfotomaculia;o__Desulfotomaculales;f__Desulfurisporaceae;g__Desulfurispora;s__uncultured_bacterium | 0 |
| d__Bacteria;p__Proteobacteria;c__Alphaproteobacteria;o__Acetobacterales;f__Acetobacteraceae;g__Acidisphaera;s__uncultured_Alphaproteobacteria | 0 |
| d__Bacteria;p__Firmicutes;c__Bacilli;o__Paenibacillales;f__Paenibacillaceae;g__Ammoniibacillus;s__Ammoniibacillus_agariperforans | 0 |
| d__Bacteria;p__Proteobacteria;c__Gammaproteobacteria;o__Burkholderiales;f__Burkholderiaceae;g__Cupriavidus;s__Cupriavidus_sp. | 0 |
| d__Bacteria;p__Actinobacteriota;c__Actinobacteria;__;__;__;__ | 0 |
| d__Bacteria;p__Proteobacteria;c__Gammaproteobacteria;o__uncultured;f__uncultured;g__uncultured;__ | 0 |
| d__Bacteria;p__Firmicutes;c__Bacilli;o__Bacillales;f__Planococcaceae;g__Sporosarcina;s__Lysinibacillus_sphaericus | 0 |
| d__Bacteria;p__Firmicutes;c__Sulfobacillia;o__Sulfobacillales;f__Sulfobacillaceae;g__Sulfobacillus;s__Sulfobacillus_acidophilus | 0 |
| d__Bacteria;p__Bacteroidota;c__Bacteroidia;o__Flavobacteriales;f__Crocinitomicaceae;g__Fluviicola;s__uncultured_bacterium | 0 |
| d__Bacteria;p__Bacteroidota;c__Bacteroidia;o__Sphingobacteriales;f__Sphingobacteriaceae;g__Solitalea;s__uncultured_bacterium | 0 |
| d__Bacteria;p__Proteobacteria;c__Gammaproteobacteria;o__Burkholderiales;f__Rhodocyclaceae;g__Denitratisoma;s__uncultured_bacterium | 0 |
| d__Bacteria;p__Proteobacteria;c__Gammaproteobacteria;o__Enterobacterales;f__Alteromonadaceae;g__Rheinheimera;__ | 0 |
| d__Bacteria;p__Proteobacteria;c__Gammaproteobacteria;o__Pseudomonadales;f__Pseudomonadaceae;g__Pseudomonas;s__Pseudomonas_brassicacearum | 0 |
| d__Bacteria;p__Proteobacteria;c__Gammaproteobacteria;o__Xanthomonadales;f__Rhodanobacteraceae;g__Rhodanobacter;s__Rhodanobacter_lindaniclasticus | 0 |
| d__Bacteria;p__Proteobacteria;c__Gammaproteobacteria;o__Burkholderiales;f__Nitrosomonadaceae;g__DSSD61;s__uncultured_bacterium | 0 |
| d__Bacteria;p__Proteobacteria;c__Gammaproteobacteria;o__Salinisphaerales;f__Solimonadaceae;g__uncultured;__ | 0 |
| d__Bacteria;p__Proteobacteria;c__Gammaproteobacteria;o__Burkholderiales;f__Burkholderiaceae;g__Limnobacter;s__uncultured_organism | 0 |
| d__Bacteria;p__Proteobacteria;c__Alphaproteobacteria;o__NRL2;f__NRL2;g__NRL2;s__uncultured_Alphaproteobacteria | 0 |
| d__Bacteria;p__Bacteroidota;c__Bacteroidia;o__Flavobacteriales;f__Cryomorphaceae;g__uncultured;s__uncultured_bacterium | 0 |
| d__Bacteria;p__Firmicutes;c__Clostridia;o__Lachnospirales;f__Lachnospiraceae;g__uncultured;s__uncultured_organism | 0 |
| d__Bacteria;p__Firmicutes;c__Dethiobacteria;o__Dethiobacterales;f__Dethiobacteraceae;g__Dethiobacter;__ | 0 |
| d__Bacteria;p__Proteobacteria;c__Gammaproteobacteria;o__Burkholderiales;f__Procabacteriaceae;g__Procabacter;s__Candidatus_Procabacter | 0 |
| d__Bacteria;p__Cyanobacteria;c__Cyanobacteriia;o__Chloroplast;f__Chloroplast;g__Chloroplast;s__uncultured_Oscillatoria | 0 |
| d__Bacteria;p__Planctomycetota;c__Planctomycetes;o__Isosphaerales;f__Isosphaeraceae;g__Aquisphaera;s__uncultured_planctomycete | 0 |
| d__Bacteria;p__Proteobacteria;c__Gammaproteobacteria;o__Burkholderiales;f__Burkholderiaceae;g__Limnobacter;s__Limnobacter_sp. | 0 |
| d__Bacteria;p__Proteobacteria;c__Gammaproteobacteria;o__Burkholderiales;f__Burkholderiaceae;g__Burkholderia-Caballeronia-Paraburkholderia;s__Burkholderia_lata | 0 |
| d__Bacteria;p__Bacteroidota;c__Bacteroidia;o__Sphingobacteriales;f__KD3-93;g__KD3-93;s__metagenome | 0 |
| d__Bacteria;p__Firmicutes;c__Clostridia;o__Lachnospirales;f__Lachnospiraceae;g__Tyzzerella;s__uncultured_organism | 0 |
| d__Bacteria;p__Dependentiae;c__Babeliae;o__Babeliales;f__Babeliaceae;__;__ | 0 |
| d__Bacteria;p__Proteobacteria;c__Gammaproteobacteria;o__Burkholderiales;f__Nitrosomonadaceae;g__Nitrosospira;__ | 0 |
| d__Bacteria;p__Bacteroidota;c__Bacteroidia;o__Chitinophagales;f__Chitinophagaceae;g__Lacibacter;__ | 0 |
| d__Bacteria;p__Proteobacteria;c__Gammaproteobacteria;o__Xanthomonadales;f__Xanthomonadaceae;g__Arenimonas;s__metagenome | 0 |
| d__Bacteria;p__Firmicutes;c__Limnochordia;o__Limnochordales;f__Limnochordaceae;g__Limnochordaceae;s__uncultured_Firmicutes | 0 |
| d__Bacteria;p__Firmicutes;c__Clostridia;o__Oscillospirales;f__Hungateiclostridiaceae;g__Acetivibrio;s__uncultured_bacterium | 0 |
| d__Bacteria;p__Proteobacteria;c__Gammaproteobacteria;o__Xanthomonadales;f__Rhodanobacteraceae;g__Fulvimonas;s__Fulvimonas_soli | 0 |
| d__Bacteria;p__Proteobacteria;c__Alphaproteobacteria;o__Azospirillales;f__Azospirillaceae;g__Nitrospirillum;s__Nitrospirillum_amazonense | 0 |
| d__Bacteria;p__Proteobacteria;c__Alphaproteobacteria;o__Acetobacterales;f__Acetobacteraceae;g__Rhodovastum;s__uncultured_bacterium | 0 |
| d__Bacteria;p__Bacteroidota;c__Bacteroidia;o__Cytophagales;f__Microscillaceae;g__OLB12;s__uncultured_bacterium | 0 |
| d__Bacteria;p__Proteobacteria;c__Gammaproteobacteria;o__Burkholderiales;f__Burkholderiaceae;g__Burkholderia-Caballeronia-Paraburkholderia;s__uncultured_beta | 0 |
| d__Bacteria;p__Proteobacteria;c__Gammaproteobacteria;o__uncultured;f__uncultured;g__uncultured;s__uncultured_gamma | 0 |
| d__Bacteria;p__Firmicutes;c__Clostridia;o__Lachnospirales;f__Defluviitaleaceae;g__Defluviitaleaceae_UCG-011;__ | 0 |
| d__Bacteria;p__Proteobacteria;c__Gammaproteobacteria;o__Acidithiobacillales;f__Acidithiobacillaceae;g__KCM-B-112;__ | 0 |
| d__Bacteria;p__Proteobacteria;c__Alphaproteobacteria;o__Rhizobiales;f__Devosiaceae;g__Arsenicitalea;__ | 0 |
| d__Bacteria;p__Verrucomicrobiota;c__Verrucomicrobiae;o__Opitutales;f__Opitutaceae;g__uncultured;s__uncultured_Verrucomicrobia | 0 |
| d__Bacteria;p__Proteobacteria;c__Gammaproteobacteria;o__Burkholderiales;f__Alcaligenaceae;g__uncultured;__ | 0 |
| d__Bacteria;p__Proteobacteria;c__Gammaproteobacteria;o__Xanthomonadales;f__Rhodanobacteraceae;g__Rhodanobacter;s__Rhodanobacter_soli | 0 |
| d__Bacteria;p__Firmicutes;c__Clostridia;o__Clostridiales;f__Clostridiaceae;g__Clostridium_sensu_stricto_1;s__Clostridium_botulinum | 0 |
| d__Bacteria;p__Bacteroidota;c__Bacteroidia;o__Cytophagales;f__Cyclobacteriaceae;g__Algoriphagus;__ | 0 |
| d__Bacteria;p__Planctomycetota;c__Planctomycetes;o__Planctomycetales;f__Schlesneriaceae;g__Schlesneria;__ | 0 |
| d__Bacteria;p__Proteobacteria;c__Alphaproteobacteria;o__Holosporales;f__Holosporaceae;__;__ | 0 |
| d__Bacteria;p__Proteobacteria;c__Gammaproteobacteria;o__Pseudomonadales;f__Pseudohongiellaceae;g__BIyi10;s__metagenome | 0 |
| d__Bacteria;p__Firmicutes;c__Clostridia;o__Lachnospirales;f__Lachnospiraceae;g__Butyrivibrio;__ | 0 |
| d__Bacteria;p__Proteobacteria;c__Gammaproteobacteria;o__Xanthomonadales;f__Rhodanobacteraceae;g__Dyella;s__Frateuria_aurantia | 0 |
| d__Bacteria;p__Proteobacteria;c__Gammaproteobacteria;o__Xanthomonadales;f__Xanthomonadaceae;g__Arenimonas;s__Arenimonas_sp. | 0 |
| d__Bacteria;p__Proteobacteria;c__Alphaproteobacteria;o__Puniceispirillales;f__uncultured;g__uncultured;s__metagenome | 0 |
| d__Bacteria;p__Proteobacteria;c__Gammaproteobacteria;o__Legionellales;f__Legionellaceae;g__Legionella;s__Legionella_sp. | 0 |
| d__Bacteria;p__Firmicutes;c__Clostridia;o__Peptostreptococcales-Tissierellales;f__Peptostreptococcales-Tissierellales;__;__ | 0 |
| d__Bacteria;p__Proteobacteria;c__Gammaproteobacteria;o__Burkholderiales;f__Nitrosomonadaceae;g__Nitrosomonas;s__uncultured_bacterium | 0 |
| d__Bacteria;p__Proteobacteria;c__Alphaproteobacteria;o__Puniceispirillales;f__uncultured;g__uncultured;s__uncultured_bacterium | 0 |
| d__Bacteria;p__Proteobacteria;c__Alphaproteobacteria;o__Rickettsiales;f__Rickettsiaceae;g__Candidatus_Megaira;__ | 0 |
| d__Bacteria;p__Proteobacteria;c__Gammaproteobacteria;o__Diplorickettsiales;f__Diplorickettsiaceae;g__Diplorickettsiaceae;s__uncultured_bacterium | 0 |
| d__Bacteria;p__Proteobacteria;c__Gammaproteobacteria;o__Enterobacterales;f__Enterobacteriaceae;g__Raoultella;s__Raoultella_ornithinolytica | 0 |
| d__Bacteria;p__Actinobacteriota;c__Actinobacteria;o__Micrococcales;f__Microbacteriaceae;g__Marisediminicola;__ | 0 |
| d__Bacteria;p__Firmicutes;c__Clostridia;o__Oscillospirales;f__Ruminococcaceae;g__uncultured;__ | 0 |
| d__Bacteria;p__Actinobacteriota;c__Actinobacteria;o__Micrococcales;f__Microbacteriaceae;g__Cryobacterium;__ | 0 |
| d__Bacteria;p__Firmicutes;c__Clostridia;o__Clostridiales;f__Clostridiaceae;g__Hathewaya;s__Clostridiaceae_bacterium | 0 |
| d__Bacteria;p__Firmicutes;c__Desulfotomaculia;o__Desulfotomaculales;f__Desulfurisporaceae;g__Desulfurispora;__ | 0 |
| d__Bacteria;p__Proteobacteria;c__Gammaproteobacteria;o__Nitrosococcales;f__Methylophagaceae;g__Methylophaga;__ | 0 |
| d__Bacteria;p__Proteobacteria;c__Alphaproteobacteria;o__Puniceispirillales;f__Puniceispirillales_Incertae_Sedis;g__Constrictibacter;__ | 0 |
| d__Bacteria;p__Proteobacteria;c__Gammaproteobacteria;o__Steroidobacterales;f__Steroidobacteraceae;g__uncultured;s__metagenome | 0 |
| d__Bacteria;p__Proteobacteria;c__Alphaproteobacteria;o__Rhizobiales;f__Beijerinckiaceae;g__Methylobacterium-Methylorubrum;s__Methylobacterium_radiotolerans | 0 |
| d__Bacteria;p__Planctomycetota;c__Planctomycetes;o__Pirellulales;f__Pirellulaceae;g__uncultured;s__uncultured_Planctomycetaceae | 0 |
| d__Bacteria;p__Firmicutes;c__Clostridia;o__Oscillospirales;f__Ruminococcaceae;g__Paludicola;__ | 0 |
| d__Bacteria;p__Firmicutes;c__Clostridia;o__Clostridiales;f__Clostridiaceae;g__Clostridium_sensu_stricto_5;__ | 0 |
| d__Bacteria;p__Firmicutes;c__Bacilli;o__uncultured;f__uncultured;g__uncultured;s__uncultured_compost | 0 |
| d__Bacteria;p__Bacteroidota;c__Bacteroidia;o__Cytophagales;f__Cyclobacteriaceae;g__Tunicatimonas;__ | 0 |
| d__Bacteria;p__Proteobacteria;c__Alphaproteobacteria;o__Sphingomonadales;f__Sphingomonadaceae;g__Porphyrobacter;__ | 0 |
| d__Bacteria;p__Firmicutes;c__Clostridia;o__Clostridiales;f__Clostridiaceae;g__Clostridium_sensu_stricto_1;s__Clostridium_septicum | 0 |
| d__Bacteria;p__Desulfobacterota;c__Desulfuromonadia;o__Desulfuromonadales;f__Syntrophotaleaceae;g__Syntrophotalea;__ | 0 |
| d__Bacteria;p__Proteobacteria;c__Alphaproteobacteria;o__Micropepsales;f__Micropepsaceae;g__Micropepsis;__ | 0 |
| d__Bacteria;p__Proteobacteria;c__Alphaproteobacteria;o__Rhodobacterales;f__Rhodobacteraceae;g__Defluviimonas;s__bacterium_enrichment | 0 |
| d__Bacteria;p__Proteobacteria;c__Alphaproteobacteria;o__Rhizobiales;f__Hyphomicrobiaceae;g__uncultured;s__uncultured_Hyphomicrobiaceae | 0 |
| d__Bacteria;p__Proteobacteria;c__Gammaproteobacteria;o__Burkholderiales;f__Rhodocyclaceae;g__Uliginosibacterium;__ | 0 |
| d__Bacteria;p__Actinobacteriota;c__Actinobacteria;o__Micrococcales;f__Bogoriellaceae;g__Georgenia;__ | 0 |
| d__Bacteria;p__Actinobacteriota;c__Actinobacteria;o__Micrococcales;f__Micrococcaceae;g__Nesterenkonia;__ | 0 |
| d__Bacteria;p__Actinobacteriota;c__Actinobacteria;o__Nitriliruptorales;f__Nitriliruptoraceae;g__Egicoccus;__ | 0 |
| d__Bacteria;p__Firmicutes;c__Clostridia;o__Peptostreptococcales-Tissierellales;f__Anaerovoracaceae;g__Anaerovorax;__ | 0 |
| d__Bacteria;p__Proteobacteria;c__Gammaproteobacteria;o__Burkholderiales;f__Nitrosomonadaceae;g__966-1;__ | 0 |
| d__Bacteria;p__Actinobacteriota;c__Thermoleophilia;o__Gaiellales;f__Gaiellaceae;g__Gaiella;__ | 0 |
| d__Bacteria;p__Proteobacteria;c__Gammaproteobacteria;o__Pseudomonadales;f__Pseudomonadaceae;g__Pseudomonas;s__Pseudomonas_frederiksbergensis | 0 |
| d__Bacteria;p__Firmicutes;c__Bacilli;o__Haloplasmatales;f__Haloplasmataceae;g__Haloplasma;s__uncultured_bacterium | 0 |
| d__Bacteria;p__Proteobacteria;c__Gammaproteobacteria;o__Piscirickettsiales;f__Piscirickettsiaceae;g__Candidatus_Endoecteinascidia;s__uncultured_gamma | 0 |
| d__Bacteria;p__Planctomycetota;c__Planctomycetes;o__Planctomycetales;f__uncultured;g__uncultured;s__uncultured_planctomycete | 0 |
| d__Bacteria;p__Actinobacteriota;c__Actinobacteria;o__Nitriliruptorales;f__Nitriliruptoraceae;g__Nitriliruptoraceae;__ | 0 |
| d__Bacteria;p__Proteobacteria;c__Gammaproteobacteria;o__Enterobacterales;f__Enterobacteriaceae;g__Escherichia-Shigella;s__Shigella_sonnei | 0 |
| d__Bacteria;p__Firmicutes;c__Bacilli;o__Bacillales;f__Bacillaceae;g__Oceanobacillus;s__Oceanobacillus_iheyensis | 0 |
| d__Bacteria;p__Firmicutes;c__Bacilli;o__Lactobacillales;f__Lactobacillaceae;g__Weissella;s__Weissella_cibaria | 0 |
| d__Bacteria;p__Acidobacteriota;c__Blastocatellia;o__Blastocatellales;f__Blastocatellaceae;g__uncultured;s__uncultured_Acidobacteria | 0 |
| d__Bacteria;p__Proteobacteria;c__Gammaproteobacteria;o__Xanthomonadales;f__Xanthomonadaceae;g__uncultured;__ | 0 |
| d__Bacteria;p__Bdellovibrionota;c__Bdellovibrionia;o__Bacteriovoracales;f__Bacteriovoracaceae;__;__ | 0 |
| d__Bacteria;p__Proteobacteria;c__Gammaproteobacteria;o__Pseudomonadales;f__Pseudomonadaceae;g__Pseudomonas;s__Pseudomonas_fluorescens | 0 |
| d__Bacteria;p__Acidobacteriota;c__Blastocatellia;o__Blastocatellales;f__Blastocatellaceae;g__JGI_0001001-H03;__ | 0 |
| d__Bacteria;p__Myxococcota;c__Polyangia;o__Polyangiales;f__Polyangiaceae;g__Minicystis;__ | 0 |
| d__Bacteria;p__Proteobacteria;c__Gammaproteobacteria;o__Burkholderiales;f__Rhodocyclaceae;g__Azoarcus;s__uncultured_bacterium | 0 |
| d__Bacteria;p__Bacteroidota;c__Bacteroidia;o__Cytophagales;__;__;__ | 0 |
| d__Bacteria;p__Proteobacteria;c__Gammaproteobacteria;o__Burkholderiales;f__Alcaligenaceae;g__Bordetella;s__Bordetella_petrii | 0 |
| d__Bacteria;p__Proteobacteria;c__Gammaproteobacteria;o__Pseudomonadales;f__Halomonadaceae;g__Halomonas;__ | 0 |
| d__Bacteria;p__Firmicutes;c__Bacilli;o__Thermoactinomycetales;f__Thermoactinomycetaceae;g__Laceyella;s__Laceyella_sp. | 0 |
| d__Bacteria;p__Proteobacteria;c__Gammaproteobacteria;o__Burkholderiales;f__Comamonadaceae;g__Rhizobacter;s__Chaetosphaeridium_globosum | 0 |
| d__Bacteria;p__Firmicutes;c__Bacilli;o__Bacillales;f__Bacillaceae;g__Bacillus;s__Bacillus_butanolivorans | 0 |
| d__Bacteria;p__Firmicutes;c__Bacilli;o__Paenibacillales;f__Paenibacillaceae;g__Paenibacillus;s__Paenibacillus_borealis | 0 |
| d__Bacteria;p__Firmicutes;c__Clostridia;o__Peptostreptococcales-Tissierellales;f__Peptostreptococcaceae;g__Tepidibacter;__ | 0 |
| d__Bacteria;p__Proteobacteria;c__Gammaproteobacteria;o__Pseudomonadales;f__Cellvibrionaceae;g__Cellvibrio;__ | 0 |
| d__Bacteria;p__Verrucomicrobiota;c__Kiritimatiellae;o__WCHB1-41;f__WCHB1-41;g__WCHB1-41;s__uncultured_bacterium | 0 |
| d__Bacteria;p__Proteobacteria;c__Alphaproteobacteria;o__Sphingomonadales;f__Sphingomonadaceae;g__Blastomonas;__ | 0 |
| d__Bacteria;p__Acidobacteriota;c__Aminicenantia;o__Aminicenantales;f__Aminicenantales;g__Aminicenantales;s__uncultured_bacterium | 0 |
| d__Bacteria;p__Actinobacteriota;c__Actinobacteria;o__Streptosporangiales;f__Streptosporangiaceae;g__Planotetraspora;__ | 0 |
| d__Bacteria;p__Proteobacteria;c__Gammaproteobacteria;o__EC3;f__EC3;g__EC3;__ | 0 |
| d__Bacteria;p__Proteobacteria;c__Gammaproteobacteria;o__Diplorickettsiales;f__Diplorickettsiaceae;g__uncultured;s__metagenome | 0 |
| d__Bacteria;p__Proteobacteria;c__Gammaproteobacteria;o__Enterobacterales;f__Enterobacteriaceae;g__Escherichia-Shigella;s__Shigella_boydii | 0 |
| d__Bacteria;p__Bacteroidota;c__Bacteroidia;o__Cytophagales;f__Cyclobacteriaceae;g__uncultured;__ | 0 |
| d__Bacteria;p__Actinobacteriota;c__Actinobacteria;o__Frankiales;f__Frankiales;g__Frankiales;s__uncultured_bacterium | 0 |
| d__Bacteria;p__Bacteroidota;c__Bacteroidia;o__Flavobacteriales;f__Weeksellaceae;g__Moheibacter;__ | 0 |
| d__Bacteria;p__Actinobacteriota;c__Actinobacteria;o__Nitriliruptorales;f__Nitriliruptoraceae;__;__ | 0 |
| d__Bacteria;p__Firmicutes;c__Bacilli;o__Bacillales;f__Bacillaceae;g__Bacillus;s__Bacillus_infantis | 0 |
| d__Bacteria;p__Bacteroidota;c__Bacteroidia;o__Chitinophagales;f__Chitinophagaceae;g__Flavitalea;s__Flavitalea_populi | 0 |
| d__Bacteria;p__Proteobacteria;c__Gammaproteobacteria;o__Burkholderiales;f__Oxalobacteraceae;g__Massilia;s__Massilia_putida | 0 |
| d__Bacteria;p__Actinobacteriota;c__Actinobacteria;o__Corynebacteriales;f__Nocardiaceae;g__Nocardia;s__Nocardia_nova | 0 |
| d__Bacteria;p__Firmicutes;c__Clostridia;o__Clostridiales;f__uncultured;g__uncultured;s__uncultured_Clostridia | 0 |
| d__Bacteria;p__Firmicutes;c__Thermacetogenia;o__Thermacetogeniales;f__Thermacetogeniaceae;g__Thermacetogenium;s__uncultured_bacterium | 0 |
| d__Bacteria;p__Firmicutes;c__Bacilli;o__Thermoactinomycetales;f__Thermoactinomycetaceae;g__Kroppenstedtia;__ | 0 |
| d__Bacteria;p__Firmicutes;c__Bacilli;o__Bacillales;f__Bacillaceae;g__Bacillus;s__Bacillus_lehensis | 0 |
| d__Bacteria;p__Proteobacteria;c__Alphaproteobacteria;o__Rhizobiales;f__Xanthobacteraceae;g__uncultured;s__uncultured_Hyphomicrobiaceae | 0 |
| d__Bacteria;p__Proteobacteria;c__Gammaproteobacteria;o__Pseudomonadales;f__Moraxellaceae;g__Alkanindiges;s__uncultured_bacterium | 0 |
| d__Bacteria;p__Firmicutes;c__Clostridia;o__Peptostreptococcales-Tissierellales;f__Family_XI;g__Tepidimicrobium;s__[Clostridium]_ultunense | 0 |
| d__Bacteria;p__Firmicutes;c__Limnochordia;o__Limnochordales;f__Limnochordaceae;g__Limnochorda;s__uncultured_bacterium | 0 |
| d__Bacteria;p__Firmicutes;c__Desulfotomaculia;o__Desulfotomaculales;f__Desulfotomaculaceae;g__Desulfofarcimen;s__Desulfofarcimen_acetoxidans | 0 |
| d__Bacteria;p__Firmicutes;c__Symbiobacteriia;o__Symbiobacteriales;f__Symbiobacteraceae;g__uncultured;__ | 0 |
| d__Bacteria;p__Firmicutes;c__Desulfitobacteriia;o__Desulfitobacteriales;f__Desulfitobacteriaceae;g__Desulfitobacterium;s__uncultured_Desulfosporosinus | 0 |
| d__Bacteria;p__Firmicutes;c__Clostridia;o__Clostridiales;f__Clostridiaceae;g__Clostridium_sensu_stricto_13;s__Clostridium_estertheticum | 0 |
| d__Bacteria;p__Proteobacteria;c__Alphaproteobacteria;o__Sphingomonadales;f__Sphingomonadaceae;g__Rhizorhapis;__ | 0 |
| d__Bacteria;p__Planctomycetota;c__Planctomycetes;o__Isosphaerales;f__Isosphaeraceae;g__Aquisphaera;s__uncultured_Singulisphaera | 0 |
| d__Bacteria;p__Firmicutes;c__Bacilli;o__Alicyclobacillales;f__Alicyclobacillaceae;g__Alicyclobacillus;s__Alicyclobacillus_fodiniaquatilis | 0 |
| d__Bacteria;p__Firmicutes;c__Desulfitobacteriia;o__Desulfitobacteriales;f__uncultured;g__uncultured;s__uncultured_bacterium | 0 |
| d__Bacteria;p__Firmicutes;c__Symbiobacteriia;o__Symbiobacteriales;f__Symbiobacteraceae;__;__ | 0 |
| d__Bacteria;p__Proteobacteria;c__Alphaproteobacteria;o__Rhizobiales;f__Rhizobiales_Incertae_Sedis;g__Phreatobacter;s__uncultured_bacterium | 0 |
| d__Bacteria;p__Acidobacteriota;c__Acidobacteriae;o__Acidobacteriales;f__Acidobacteriaceae_(Subgroup_1);g__Acidipila-Silvibacterium;s__Acidobacteriaceae_bacterium | 0 |
| d__Bacteria;p__Firmicutes;c__Negativicutes;o__Veillonellales-Selenomonadales;f__Sporomusaceae;g__Anaerospora;__ | 0 |
| d__Bacteria;p__Proteobacteria;c__Gammaproteobacteria;o__Burkholderiales;f__Nitrosomonadaceae;g__DSSD61;__ | 0 |
| d__Bacteria;p__Actinobacteriota;c__Actinobacteria;o__Micrococcales;f__Micrococcaceae;g__Paenarthrobacter;__ | 0 |
| d__Bacteria;p__Actinobacteriota;c__Actinobacteria;o__Corynebacteriales;f__Corynebacteriaceae;g__Lawsonella;s__uncultured_bacterium | 0 |
| d__Bacteria;p__Firmicutes;c__Negativicutes;o__Veillonellales-Selenomonadales;f__Sporomusaceae;g__Sporomusa;s__Sporomusa_malonica | 0 |
| d__Bacteria;p__Firmicutes;c__Bacilli;o__Bacillales;f__Planococcaceae;g__Planococcus;__ | 0 |
| d__Bacteria;p__Firmicutes;c__Bacilli;o__Bacillales;f__Planococcaceae;g__Viridibacillus;__ | 0 |
| d__Bacteria;p__Firmicutes;c__Bacilli;o__Bacillales;f__Bacillaceae;g__Caldibacillus;__ | 0 |
| d__Bacteria;p__Actinobacteriota;c__Actinobacteria;o__Corynebacteriales;f__Mycobacteriaceae;g__Mycobacterium;s__Mycobacterium_paraterrae | 0 |
| d__Bacteria;p__Firmicutes;c__Clostridia;o__Oscillospirales;f__Oscillospiraceae;g__Oscillibacter;s__uncultured_bacterium | 0 |
| d__Bacteria;p__Proteobacteria;c__Gammaproteobacteria;o__Burkholderiales;f__Comamonadaceae;g__Pelomonas;s__uncultured_bacterium | 0 |
| d__Bacteria;p__Actinobacteriota;c__Actinobacteria;o__Micrococcales;f__Microbacteriaceae;g__Chryseoglobus;__ | 0 |
| d__Bacteria;p__Firmicutes;c__Bacilli;o__Thermoactinomycetales;f__Thermoactinomycetaceae;g__Thermoflavimicrobium;s__uncultured_bacterium | 0 |
| d__Bacteria;p__Firmicutes;c__Negativicutes;o__Veillonellales-Selenomonadales;f__Sporomusaceae;g__Anaerosinus;__ | 0 |
| d__Bacteria;p__Cyanobacteria;c__Cyanobacteriia;o__Cyanobacteriales;f__uncultured;g__uncultured;s__uncultured_bacterium | 0 |
| d__Bacteria;p__Proteobacteria;c__Gammaproteobacteria;o__Burkholderiales;f__Burkholderiaceae;g__Burkholderia-Caballeronia-Paraburkholderia;s__Paraburkholderia_kururiensis | 0 |
| d__Bacteria;p__Proteobacteria;c__Gammaproteobacteria;o__Burkholderiales;f__Rhodocyclaceae;g__Zoogloea;s__uncultured_bacterium | 0 |
| d__Bacteria;p__Proteobacteria;c__Gammaproteobacteria;o__Xanthomonadales;f__Rhodanobacteraceae;g__Dyella;s__uncultured_bacterium | 0 |
| d__Bacteria;p__Firmicutes;c__Bacilli;o__Lactobacillales;f__Lactobacillaceae;g__Limosilactobacillus;__ | 0 |
| d__Bacteria;p__Proteobacteria;c__Alphaproteobacteria;o__Sphingomonadales;f__Sphingomonadaceae;g__Sphingomonas;s__uncultured_Alphaproteobacteria | 0 |
| d__Bacteria;p__Proteobacteria;c__Alphaproteobacteria;o__Rhizobiales;f__Rhizobiaceae;g__Aurantimonas;__ | 0 |
| d__Bacteria;p__Proteobacteria;c__Gammaproteobacteria;o__Burkholderiales;f__Burkholderiaceae;g__Burkholderia-Caballeronia-Paraburkholderia;s__Paraburkholderia_hospita | 0 |
| d__Bacteria;p__Firmicutes;c__Clostridia;o__Lachnospirales;f__Lachnospiraceae;g__[Ruminococcus]_gnavus_group;s__uncultured_organism | 0 |
| d__Bacteria;p__Firmicutes;c__Bacilli;o__Lactobacillales;__;__;__ | 0 |
| d__Bacteria;p__Proteobacteria;c__Gammaproteobacteria;o__Burkholderiales;f__Burkholderiaceae;g__Burkholderia-Caballeronia-Paraburkholderia;s__Paraburkholderia_sp. | 0 |
| d__Bacteria;p__Patescibacteria;c__Microgenomatia;o__Candidatus_Levybacteria;f__Candidatus_Levybacteria;g__Candidatus_Levybacteria;__ | 0 |
| d__Bacteria;p__Proteobacteria;c__Alphaproteobacteria;o__Rhizobiales;f__Beijerinckiaceae;g__Methylocapsa;s__uncultured_bacterium | 0 |
| d__Bacteria;p__Firmicutes;c__Clostridia;o__Lachnospirales;f__Lachnospiraceae;g__Dorea;s__uncultured_bacterium | 0 |
| d__Bacteria;p__Patescibacteria;c__Saccharimonadia;o__Saccharimonadales;f__LWQ8;g__LWQ8;s__uncultured_Candidatus | 0 |
| d__Bacteria;p__Proteobacteria;c__Alphaproteobacteria;o__Acetobacterales;f__Acetobacteraceae;g__uncultured;s__metagenome | 0 |
| d__Bacteria;p__Proteobacteria;c__Gammaproteobacteria;o__Burkholderiales;f__Alcaligenaceae;g__Candidimonas;__ | 0 |
| d__Bacteria;p__Firmicutes;c__Clostridia;o__Lachnospirales;f__Lachnospiraceae;g__[Ruminococcus]_torques_group;s__uncultured_organism | 0 |
| d__Bacteria;p__Proteobacteria;c__Alphaproteobacteria;o__Rhizobiales;f__Methyloligellaceae;g__Methyloligella;s__Methyloligella_solikamskensis | 0 |
| d__Bacteria;p__Planctomycetota;c__Phycisphaerae;o__Phycisphaerales;f__Phycisphaeraceae;g__I-8;s__uncultured_bacterium | 0 |
| d__Bacteria;p__Patescibacteria;c__Saccharimonadia;o__Saccharimonadales;f__WWH38;g__WWH38;__ | 0 |
| d__Bacteria;p__Firmicutes;c__Clostridia;o__Peptostreptococcales-Tissierellales;f__Family_XI;g__Fenollaria;__ | 0 |
| d__Bacteria;p__Firmicutes;c__Limnochordia;o__MBA03;f__MBA03;g__MBA03;s__uncultured_prokaryote | 0 |
| d__Bacteria;p__Proteobacteria;c__Gammaproteobacteria;o__Enterobacterales;f__Enterobacteriaceae;g__Enterobacter;s__Leclercia_sp. | 0 |
| d__Bacteria;p__Proteobacteria;c__Gammaproteobacteria;o__Enterobacterales;f__Enterobacteriaceae;g__Klebsiella;s__Klebsiella_oxytoca | 0 |
| d__Bacteria;p__Proteobacteria;c__Gammaproteobacteria;o__Burkholderiales;f__Burkholderiaceae;g__Burkholderia-Caballeronia-Paraburkholderia;s__Caballeronia_zhejiangensis | 0 |
| d__Bacteria;p__Firmicutes;c__Negativicutes;o__Veillonellales-Selenomonadales;f__Selenomonadaceae;g__Megamonas;__ | 0 |
| d__Bacteria;p__Firmicutes;c__Clostridia;o__Peptostreptococcales-Tissierellales;f__Family_XI;g__Anaerococcus;s__uncultured_bacterium | 0 |
| d__Bacteria;p__Proteobacteria;c__Gammaproteobacteria;o__Enterobacterales;f__Enterobacteriaceae;g__Enterobacter;s__Enterobacter_hormaechei | 0 |
| d__Bacteria;p__Proteobacteria;c__Gammaproteobacteria;o__Burkholderiales;f__Burkholderiaceae;g__Ralstonia;s__Ralstonia_mannitolilytica | 0 |
| d__Bacteria;p__Firmicutes;c__Clostridia;o__Lachnospirales;f__Lachnospiraceae;g__Eisenbergiella;__ | 0 |
| d__Bacteria;p__Firmicutes;c__Bacilli;o__Erysipelotrichales;f__Erysipelatoclostridiaceae;g__Erysipelotrichaceae_UCG-003;__ | 0 |
| d__Bacteria;p__Proteobacteria;c__Gammaproteobacteria;o__Xanthomonadales;f__Rhodanobacteraceae;g__Fulvimonas;__ | 0 |
| d__Bacteria;p__Halanaerobiaeota;c__Halanaerobiia;o__Halanaerobiales;f__Halobacteroidaceae;g__uncultured;__ | 0 |
| d__Bacteria;p__Firmicutes;c__Bacilli;o__Lactobacillales;f__Enterococcaceae;g__Enterococcus;s__Enterococcus_raffinosus | 0 |
| d__Bacteria;p__Proteobacteria;c__Gammaproteobacteria;o__Enterobacterales;f__Enterobacteriaceae;g__Citrobacter;s__Citrobacter_freundii | 0 |
| d__Bacteria;p__Proteobacteria;c__Gammaproteobacteria;o__Enterobacterales;f__Pasteurellaceae;g__Haemophilus;s__Haemophilus_influenzae | 0 |
| d__Bacteria;p__Firmicutes;c__Bacilli;o__Lactobacillales;f__Lactobacillaceae;g__Lacticaseibacillus;__ | 0 |
| d__Bacteria;p__Proteobacteria;c__Gammaproteobacteria;o__Burkholderiales;f__Methylophilaceae;g__Methylophilus;s__uncultured_bacterium | 0 |
| d__Bacteria;p__Firmicutes;c__Bacilli;o__Lactobacillales;f__Enterococcaceae;g__Enterococcus;s__uncultured_bacterium | 0 |
| d__Bacteria;p__Firmicutes;c__Clostridia;o__Oscillospirales;f__Ruminococcaceae;g__Incertae_Sedis;s__uncultured_bacterium | 0 |
| d__Bacteria;p__Firmicutes;c__Clostridia;o__Oscillospirales;f__Ruminococcaceae;g__Faecalibacterium;s__Faecalibacterium_prausnitzii | 0 |
| d__Bacteria;p__Firmicutes;c__Clostridia;o__Lachnospirales;f__Lachnospiraceae;g__Lachnospira;__ | 0 |
| d__Bacteria;p__Firmicutes;c__Bacilli;o__Lactobacillales;f__Lactobacillaceae;g__Lacticaseibacillus;s__Lactobacillus_paracasei | 0 |
| d__Bacteria;p__Firmicutes;c__Clostridia;o__Lachnospirales;f__Lachnospiraceae;g__[Ruminococcus]_torques_group;__ | 0 |
| d__Bacteria;p__Proteobacteria;c__Gammaproteobacteria;o__Xanthomonadales;f__Rhodanobacteraceae;g__Dokdonella;s__uncultured_Dokdonella | 0 |
| d__Bacteria;p__Acidobacteriota;c__Acidobacteriae;o__Acidobacteriales;f__Acidobacteriaceae_(Subgroup_1);g__Granulicella;s__uncultured_Acidobacteria | 0 |
| d__Bacteria;p__Bacteroidota;c__Bacteroidia;o__Sphingobacteriales;f__Lentimicrobiaceae;g__Lentimicrobiaceae;s__uncultured_prokaryote | 0 |
| d__Bacteria;p__Firmicutes;c__Clostridia;o__Clostridiales;f__Clostridiaceae;g__Clostridium_sensu_stricto_1;s__metagenome | 0 |
| d__Bacteria;p__Bacteroidota;c__Bacteroidia;o__Bacteroidales;f__Bacteroidaceae;g__Bacteroides;s__Bacteroides_dorei | 0 |
| d__Bacteria;p__Actinobacteriota;c__Actinobacteria;o__Micromonosporales;f__Micromonosporaceae;g__Actinoplanes;__ | 0 |
| d__Bacteria;p__Proteobacteria;c__Alphaproteobacteria;o__Sphingomonadales;f__Sphingomonadaceae;g__Sphingomonas;s__Oryza_sativa | 0 |
| d__Bacteria;p__Proteobacteria;c__Gammaproteobacteria;o__Xanthomonadales;f__Rhodanobacteraceae;g__Mizugakiibacter;s__uncultured_Xanthomonadaceae | 0 |
| d__Bacteria;p__Proteobacteria;c__Alphaproteobacteria;o__Holosporales;f__Holosporaceae;g__Candidatus_Bealeia;__ | 0 |
| d__Bacteria;p__Cyanobacteria;c__Cyanobacteriia;o__Cyanobacteriales;f__Nostocaceae;g__Nostoc_PCC-7524;s__Nostoc_sp. | 0 |
| d__Bacteria;p__Patescibacteria;c__Microgenomatia;o__Microgenomatia;f__Microgenomatia;g__Microgenomatia;s__uncultured_bacterium | 0 |
| d__Bacteria;p__Proteobacteria;c__Gammaproteobacteria;o__Burkholderiales;f__Rhodocyclaceae;g__Azoarcus;s__Azoarcus_olearius | 0 |
| d__Bacteria;p__Proteobacteria;c__Gammaproteobacteria;o__Burkholderiales;f__Leeiaceae;g__Leeia;s__uncultured_bacterium | 0 |
| d__Bacteria;p__Proteobacteria;c__Gammaproteobacteria;o__EC3;f__EC3;g__EC3;s__uncultured_organism | 0 |
| d__Bacteria;p__Proteobacteria;c__Gammaproteobacteria;o__Burkholderiales;f__Gallionellaceae;g__Candidatus_Nitrotoga;__ | 0 |
| d__Bacteria;p__Bacteroidota;c__Bacteroidia;o__Cytophagales;f__Spirosomaceae;g__Leadbetterella;s__uncultured_bacterium | 0 |
| d__Bacteria;p__Patescibacteria;c__WWE3;o__WWE3;f__WWE3;g__WWE3;__ | 0 |
| d__Bacteria;p__Nitrospirota;c__Thermodesulfovibrionia;o__uncultured;f__uncultured;g__uncultured;s__uncultured_bacterium | 0 |
| d__Bacteria;p__Proteobacteria;c__Alphaproteobacteria;o__Caulobacterales;f__Parvularculaceae;g__Amphiplicatus;s__uncultured_bacterium | 0 |
| d__Bacteria;p__Proteobacteria;c__Gammaproteobacteria;o__Pseudomonadales;f__211ds20;g__211ds20;__ | 0 |
| d__Bacteria;p__Actinobacteriota;c__Actinobacteria;o__Frankiales;f__Sporichthyaceae;g__uncultured;s__uncultured_bacterium | 0 |
| d__Bacteria;p__Nitrospirota;c__Leptospirillia;o__Leptospirillales;f__Leptospirillaceae;g__Leptospirillum;s__uncultured_bacterium | 0 |
| d__Bacteria;p__Proteobacteria;c__Alphaproteobacteria;o__Rhodospirillales;f__uncultured;g__uncultured;s__metagenome | 0 |
| d__Bacteria;p__Firmicutes;c__Clostridia;o__Clostridia_vadinBB60_group;f__Clostridia_vadinBB60_group;g__Clostridia_vadinBB60_group;s__uncultured_organism | 0 |
| d__Bacteria;p__Firmicutes;c__Clostridia;o__Lachnospirales;f__Lachnospiraceae;g__Lachnospiraceae_NK4B4_group;s__uncultured_bacterium | 0 |
| d__Bacteria;p__Firmicutes;c__Clostridia;o__Clostridiales;f__Clostridiaceae;g__Clostridium_sensu_stricto_12;s__Clostridium_kluyveri | 0 |
| d__Bacteria;p__Proteobacteria;c__Gammaproteobacteria;o__Burkholderiales;f__Rhodocyclaceae;g__Sulfuritalea;__ | 0 |
| d__Bacteria;p__Acidobacteriota;c__Vicinamibacteria;o__Vicinamibacterales;f__Vicinamibacteraceae;g__Vicinamibacteraceae;s__uncultured_Acidobacteriales | 0 |
| d__Bacteria;p__Proteobacteria;c__Gammaproteobacteria;o__Burkholderiales;f__Oxalobacteraceae;g__Massilia;s__Janthinobacterium_agaricidamnosum | 0 |
| d__Bacteria;p__Proteobacteria;c__Gammaproteobacteria;o__Enterobacterales;f__Enterobacteriaceae;g__Escherichia-Shigella;s__Shigella_flexneri | 0 |
| d__Bacteria;p__Proteobacteria;c__Gammaproteobacteria;o__Burkholderiales;f__Gallionellaceae;g__Sideroxydans;__ | 0 |
| d__Bacteria;p__Firmicutes;c__Clostridia;o__Clostridiales;f__Clostridiaceae;g__Clostridium_sensu_stricto_12;s__Clostridium_pasteurianum | 0 |
| d__Bacteria;p__Firmicutes;c__Bacilli;o__Bacillales;f__Bacillaceae;g__Sinibacillus;s__Sinibacillus_soli | 0 |
| d__Bacteria;p__Proteobacteria;c__Alphaproteobacteria;o__Caedibacterales;f__Caedibacteraceae;__;__ | 0 |
| d__Bacteria;p__Proteobacteria;c__Gammaproteobacteria;o__Acidiferrobacterales;f__Acidiferrobacteraceae;g__uncultured;s__uncultured_bacterium | 0 |
| d__Bacteria;p__Firmicutes;c__Clostridia;o__Oscillospirales;f__Hungateiclostridiaceae;g__Fastidiosipila;__ | 0 |
| d__Bacteria;p__Firmicutes;c__Clostridia;o__Peptostreptococcales-Tissierellales;f__Proteiniboraceae;g__Proteiniborus;__ | 0 |
| d__Bacteria;p__Proteobacteria;c__Gammaproteobacteria;o__Enterobacterales;f__Yersiniaceae;g__Serratia;s__Serratia_liquefaciens | 0 |
| d__Bacteria;p__Actinobacteriota;c__Actinobacteria;o__Micrococcales;f__Micrococcaceae;g__Kocuria;__ | 0 |
| d__Bacteria;p__Firmicutes;c__Clostridia;o__Eubacteriales;f__Garciellaceae;__;__ | 0 |
| d__Bacteria;p__Firmicutes;c__Clostridia;o__Clostridiales;f__Clostridiaceae;g__uncultured;__ | 0 |
| d__Bacteria;p__Proteobacteria;c__Gammaproteobacteria;o__Halothiobacillales;f__Halothiobacillaceae;g__Halothiobacillus;s__Halothiobacillus_neapolitanus | 0 |
| d__Bacteria;p__Proteobacteria;c__Gammaproteobacteria;o__Burkholderiales;f__Rhodocyclaceae;g__Methyloversatilis;s__uncultured_bacterium | 0 |
| d__Bacteria;p__Firmicutes;c__Bacilli;o__Bacillales;f__Bacillaceae;g__Anaerobacillus;s__uncultured_bacterium | 0 |
| d__Bacteria;p__Myxococcota;c__Myxococcia;o__Myxococcales;f__Myxococcaceae;g__Cystobacter;__ | 0 |
| d__Bacteria;p__Firmicutes;c__Thermoanaerobacteria;o__Thermoanaerobacterales;f__Thermoanaerobacteraceae;g__Caldanaerobius;s__uncultured_bacterium | 0 |
| d__Bacteria;p__Desulfobacterota;c__Desulfobulbia;o__Desulfobulbales;f__Desulfocapsaceae;g__[Desulfobacterium]_catecholicum_group;s__uncultured_bacterium | 0 |
| d__Bacteria;p__Proteobacteria;c__Gammaproteobacteria;o__Burkholderiales;f__Hydrogenophilaceae;g__Thiobacillus;__ | 0 |
| d__Bacteria;p__Myxococcota;c__Polyangia;o__Polyangiales;f__Eel-36e1D6;g__Eel-36e1D6;__ | 0 |
| d__Bacteria;p__Firmicutes;c__Clostridia;o__Clostridiales;f__Clostridiaceae;g__Clostridium_sensu_stricto_12;s__Clostridium_luticellarii | 0 |
| d__Bacteria;p__Actinobacteriota;c__Actinobacteria;o__Micrococcales;f__Intrasporangiaceae;g__Intrasporangium;__ | 0 |
| d__Bacteria;p__Proteobacteria;c__Alphaproteobacteria;o__Caedibacterales;f__Caedibacteraceae;g__Caedibacter;__ | 0 |
| d__Bacteria;p__Firmicutes;c__Bacilli;o__Bacillales;f__Planococcaceae;g__Lysinibacillus;s__uncultured_prokaryote | 0 |
| d__Bacteria;p__Firmicutes;c__Clostridia;o__Peptostreptococcales-Tissierellales;f__Peptostreptococcaceae;g__uncultured;s__uncultured_bacterium | 0 |
| d__Bacteria;p__Chloroflexi;c__P2-11E;o__P2-11E;f__P2-11E;g__P2-11E;s__uncultured_bacterium | 0 |
| d__Bacteria;p__Firmicutes;c__Incertae_Sedis;o__Incertae_Sedis;f__Incertae_Sedis;g__Gelria;s__anaerobic_digester | 0 |
| d__Bacteria;p__Acidobacteriota;c__Acidobacteriae;o__Acidobacteriales;f__Acidobacteriaceae_(Subgroup_1);g__Occallatibacter;s__Occallatibacter_riparius | 0 |
| d__Bacteria;p__Firmicutes;c__Bacilli;o__Bacillales;f__Bacillaceae;g__Ureibacillus;s__uncultured_bacterium | 0 |
| d__Bacteria;p__DTB120;c__DTB120;o__DTB120;f__DTB120;g__DTB120;s__uncultured_bacterium | 0 |
| d__Bacteria;p__Acidobacteriota;c__Acidobacteriae;o__Acidobacteriales;f__Acidobacteriaceae_(Subgroup_1);g__Terracidiphilus;s__uncultured_Acidobacteria | 0 |
| d__Bacteria;p__Firmicutes;c__Clostridia;o__Oscillospirales;f__Ruminococcaceae;g__Caproiciproducens;s__uncultured_Ruminococcus | 0 |
| d__Bacteria;p__Firmicutes;c__Clostridia;o__Lachnospirales;f__Lachnospiraceae;g__Cellulosilyticum;s__Cellulosilyticum_lentocellum | 0 |
| d__Bacteria;p__Firmicutes;c__Bacilli;o__Paenibacillales;f__Paenibacillaceae;g__Paenibacillaceae;s__Paenibacillus_sp. | 0 |
| d__Bacteria;p__Proteobacteria;c__Gammaproteobacteria;o__Burkholderiales;f__Hydrogenophilaceae;g__uncultured;s__uncultured_bacterium | 0 |
| d__Bacteria;p__Proteobacteria;c__Alphaproteobacteria;o__Rhizobiales;f__Rhizobiaceae;g__uncultured;s__uncultured_bacterium | 0 |
| d__Bacteria;p__Proteobacteria;c__Alphaproteobacteria;o__Rhodospirillales;f__Magnetospirillaceae;g__uncultured;__ | 0 |
| d__Bacteria;p__Proteobacteria;c__Alphaproteobacteria;o__Rhodospirillales;f__Magnetospirillaceae;g__uncultured;s__metagenome | 0 |
| d__Bacteria;p__Proteobacteria;c__Alphaproteobacteria;o__Acetobacterales;f__Acetobacteraceae;g__Acidiphilium;s__uncultured_bacterium | 0 |
| d__Bacteria;p__Bacteroidota;c__Bacteroidia;o__Chitinophagales;f__Chitinophagaceae;g__Edaphobaculum;s__Nemorincola_caseinilytica | 0 |
| d__Bacteria;p__Firmicutes;c__Bacilli;o__Bacillales;f__Bacillaceae;g__Bacillus;s__Bacillus_drentensis | 0 |
| d__Bacteria;p__Acidobacteriota;c__Acidobacteriae;o__Acidobacteriales;f__Acidobacteriaceae_(Subgroup_1);g__Terracidiphilus;s__Terracidiphilus_gabretensis | 0 |
| d__Bacteria;p__Proteobacteria;c__Gammaproteobacteria;o__Burkholderiales;f__Alcaligenaceae;g__Bordetella;s__Bordetella_bronchialis | 0 |
| d__Bacteria;p__Chloroflexi;c__Ktedonobacteria;o__Ktedonobacterales;f__JG30-KF-AS9;g__JG30-KF-AS9;__ | 0 |
| d__Bacteria;p__Acidobacteriota;c__Acidobacteriae;o__Acidobacteriales;f__Acidobacteriaceae_(Subgroup_1);g__Occallatibacter;s__uncultured_bacterium | 0 |
| d__Bacteria;p__Acidobacteriota;c__Acidobacteriae;o__Acidobacteriales;f__Acidobacteriaceae_(Subgroup_1);g__Acidobacterium;s__uncultured_bacterium | 0 |
| d__Bacteria;p__Actinobacteriota;c__Actinobacteria;o__Pseudonocardiales;f__Pseudonocardiaceae;g__Saccharopolyspora;s__Saccharopolyspora_rectivirgula | 0 |
| d__Bacteria;p__Actinobacteriota;c__Actinobacteria;o__Corynebacteriales;f__Mycobacteriaceae;g__Mycobacterium;s__uncultured_bacterium | 0 |
| d__Bacteria;p__Bacteroidota;c__Bacteroidia;o__Sphingobacteriales;f__Sphingobacteriaceae;g__Mucilaginibacter;s__metagenome | 0 |
| d__Bacteria;p__Verrucomicrobiota;c__Lentisphaeria;__;__;__;__ | 0 |
| d__Bacteria;p__Planctomycetota;c__Phycisphaerae;o__Phycisphaerales;f__Phycisphaeraceae;g__I-8;__ | 0 |
| d__Bacteria;p__Proteobacteria;c__Gammaproteobacteria;o__Diplorickettsiales;f__Diplorickettsiaceae;__;__ | 0 |
| d__Bacteria;p__Firmicutes;c__Bacilli;o__Bacillales;f__Planococcaceae;g__Planococcus;s__Planococcus_plakortidis | 0 |
| d__Bacteria;p__Proteobacteria;c__Gammaproteobacteria;o__Xanthomonadales;f__Rhodanobacteraceae;g__Rhodanobacter;s__uncultured_gamma | 0 |
| d__Bacteria;p__Actinobacteriota;c__Thermoleophilia;o__Solirubrobacterales;f__Solirubrobacteraceae;g__Conexibacter;s__uncultured_bacterium | 0 |
| d__Bacteria;p__Proteobacteria;c__Alphaproteobacteria;o__Defluviicoccales;f__uncultured;g__uncultured;s__uncultured_soil | 0 |
| d__Bacteria;p__Actinobacteriota;c__Actinobacteria;o__Micrococcales;f__Microbacteriaceae;g__Cryocola;s__Cryocola_antiquus | 0 |
| d__Bacteria;p__Actinobacteriota;c__Actinobacteria;o__Pseudonocardiales;f__Pseudonocardiaceae;g__Actinokineospora;__ | 0 |
| d__Bacteria;p__Proteobacteria;c__Gammaproteobacteria;o__Pseudomonadales;f__Marinobacteraceae;g__Marinobacter;__ | 0 |
| d__Bacteria;p__Fibrobacterota;c__Fibrobacteria;o__Fibrobacterales;f__Fibrobacterales;g__BBMC-4;s__uncultured_bacterium | 0 |
| d__Bacteria;p__Bacteroidota;c__Bacteroidia;o__Chitinophagales;f__Chitinophagaceae;g__Thermoflavifilum;__ | 0 |
| d__Bacteria;p__Acidobacteriota;c__Acidobacteriae;o__Acidobacteriales;f__Acidobacteriaceae_(Subgroup_1);g__Acidipila-Silvibacterium;s__Acidobacteria_bacterium | 0 |
| d__Bacteria;p__Proteobacteria;c__Gammaproteobacteria;o__Pseudomonadales;f__Marinobacteraceae;g__Marinobacter;s__uncultured_bacterium | 0 |
| d__Bacteria;p__Proteobacteria;c__Alphaproteobacteria;o__Elsterales;f__uncultured;g__uncultured;s__uncultured_forest | 0 |
| d__Bacteria;p__Proteobacteria;c__Gammaproteobacteria;o__HOC36;f__HOC36;g__HOC36;__ | 0 |
| d__Bacteria;p__Proteobacteria;c__Alphaproteobacteria;o__Zavarziniales;f__uncultured;g__uncultured;__ | 0 |
| d__Bacteria;p__Patescibacteria;c__Parcubacteria;o__Candidatus_Kaiserbacteria;f__Candidatus_Kaiserbacteria;g__Candidatus_Kaiserbacteria;s__uncultured_bacterium | 0 |
| d__Bacteria;p__Proteobacteria;c__Alphaproteobacteria;o__Acetobacterales;f__Acetobacteraceae;g__Acetobacteraceae;s__Acidisphaera_sp. | 0 |
| d__Bacteria;p__Proteobacteria;c__Alphaproteobacteria;o__Zavarziniales;f__uncultured;g__uncultured;s__uncultured_bacterium | 0 |
| d__Bacteria;p__Proteobacteria;c__Alphaproteobacteria;o__Rhizobiales;f__Xanthobacteraceae;g__Pseudolabrys;s__uncultured_Pseudolabrys | 0 |
| d__Bacteria;p__Planctomycetota;c__Phycisphaerae;o__Tepidisphaerales;f__CPla-3_termite_group;g__CPla-3_termite_group;s__uncultured_bacterium | 0 |
| d__Bacteria;p__Firmicutes;c__Bacilli;o__Entomoplasmatales;f__Entomoplasmataceae;g__Entomoplasma;__ | 0 |
| d__Bacteria;p__Acidobacteriota;c__Acidobacteriae;o__Acidobacteriales;f__Acidobacteriaceae_(Subgroup_1);g__Occallatibacter;s__uncultured_Acidobacterium | 0 |
| d__Bacteria;p__Proteobacteria;c__Alphaproteobacteria;o__Reyranellales;f__Reyranellaceae;g__Reyranella;s__alpha_proteobacterium | 0 |
| d__Bacteria;p__Proteobacteria;c__Alphaproteobacteria;o__Rickettsiales;f__Rickettsiales;g__Candidatus_Jidaibacter;__ | 0 |
| d__Bacteria;p__Gemmatimonadota;c__AKAU4049;o__AKAU4049;f__AKAU4049;g__AKAU4049;s__uncultured_bacterium | 0 |
| d__Bacteria;p__Planctomycetota;c__Phycisphaerae;o__Phycisphaerales;f__Phycisphaeraceae;g__uncultured;s__uncultured_organism | 0 |
| d__Bacteria;p__Firmicutes;c__Bacilli;o__Bacillales;f__Planococcaceae;g__Lysinibacillus;s__Lysinibacillus_endophyticus | 0 |
| d__Bacteria;p__Actinobacteriota;__;__;__;__;__ | 0 |
| d__Bacteria;p__Cyanobacteria;c__Vampirivibrionia;o__Vampirovibrionales;f__Vampirovibrionaceae;g__Vampirovibrio;s__uncultured_soil | 0 |
| d__Bacteria;p__Proteobacteria;c__Gammaproteobacteria;o__Xanthomonadales;f__Rhodanobacteraceae;g__Rhodanobacter;s__Rhodanobacter_caeni | 0 |
| d__Bacteria;p__Bdellovibrionota;c__Oligoflexia;o__0319-6G20;f__0319-6G20;g__0319-6G20;s__metagenome | 0 |
| d__Bacteria;p__Patescibacteria;c__Saccharimonadia;o__Saccharimonadales;f__Saccharimonadales;g__Saccharimonadales;s__uncultured_Candidatus | 0 |
| d__Bacteria;p__Patescibacteria;c__Parcubacteria;o__Candidatus_Kaiserbacteria;f__Candidatus_Kaiserbacteria;g__Candidatus_Kaiserbacteria;__ | 0 |
| d__Bacteria;p__Firmicutes;c__Clostridia;o__Peptostreptococcales-Tissierellales;f__Peptostreptococcaceae;g__Paeniclostridium;s__Clostridium_baratii | 0 |
| d__Bacteria;p__Proteobacteria;c__Gammaproteobacteria;o__Enterobacterales;f__Enterobacteriaceae;g__Escherichia-Shigella;s__Shigella_dysenteriae | 0 |
| d__Bacteria;p__Firmicutes;c__Clostridia;o__Lachnospirales;f__Lachnospiraceae;g__Stomatobaculum;s__uncultured_bacterium | 0 |
| d__Bacteria;p__Actinobacteriota;c__Actinobacteria;o__Micrococcales;f__Microbacteriaceae;g__Yonghaparkia;s__uncultured_bacterium | 0 |
| d__Bacteria;p__Actinobacteriota;c__Actinobacteria;o__Propionibacteriales;f__Nocardioidaceae;g__Nocardioides;s__Nocardioides_phosphati | 0 |
| d__Bacteria;p__Firmicutes;c__Bacilli;o__Lactobacillales;f__Lactobacillaceae;g__Limosilactobacillus;s__Lactobacillus_reuteri | 0 |
| d__Bacteria;p__Actinobacteriota;c__Actinobacteria;o__Propionibacteriales;f__Nocardioidaceae;g__Kribbella;__ | 0 |
| d__Bacteria;p__Proteobacteria;c__Alphaproteobacteria;o__Rhizobiales;f__Rhizobiaceae;g__Allorhizobium-Neorhizobium-Pararhizobium-Rhizobium;s__Allorhizobium_oryzae | 0 |
| d__Bacteria;p__Proteobacteria;c__Gammaproteobacteria;o__Burkholderiales;f__Oxalobacteraceae;g__Massilia;s__Massilia_sp. | 0 |
| d__Bacteria;p__Firmicutes;c__Bacilli;o__Alicyclobacillales;f__Alicyclobacillaceae;g__Tumebacillus;s__Tumebacillus_sp. | 0 |
| d__Bacteria;p__Firmicutes;c__Clostridia;o__Oscillospirales;f__Ruminococcaceae;g__Anaerotruncus;s__uncultured_Ruminococcaceae | 0 |
| d__Bacteria;p__Proteobacteria;c__Alphaproteobacteria;o__Rhizobiales;f__Beijerinckiaceae;g__alphaI_cluster;s__metagenome | 0 |
| d__Bacteria;p__Proteobacteria;c__Alphaproteobacteria;o__Rhizobiales;f__Rhizobiaceae;g__Ensifer;s__Sinorhizobium_meliloti | 0 |
| d__Bacteria;p__Firmicutes;c__Bacilli;o__Lactobacillales;f__Aerococcaceae;g__Abiotrophia;s__uncultured_bacterium | 0 |
| d__Bacteria;p__Actinobacteriota;c__Actinobacteria;o__Propionibacteriales;f__Nocardioidaceae;g__Nocardioides;s__metagenome | 0 |
| d__Bacteria;p__Actinobacteriota;c__Actinobacteria;o__Micrococcales;f__Microbacteriaceae;g__Yonghaparkia;__ | 0 |
| d__Bacteria;p__Proteobacteria;c__Gammaproteobacteria;o__Xanthomonadales;f__Xanthomonadaceae;g__Stenotrophomonas;__ | 0 |
| d__Bacteria;p__Firmicutes;c__Limnochordia;o__B55-F-B-G02;f__B55-F-B-G02;g__B55-F-B-G02;__ | 0 |
| d__Bacteria;p__Cyanobacteria;c__Cyanobacteriia;o__Chloroplast;f__Chloroplast;g__Chloroplast;s__Dactylorhiza_fuchsii | 0 |
| d__Bacteria;p__Gemmatimonadota;c__Longimicrobia;o__Longimicrobiales;f__Longimicrobiaceae;g__Longimicrobiaceae;s__uncultured_Gemmatimonadetes | 0 |
| d__Bacteria;p__Proteobacteria;c__Gammaproteobacteria;o__Enterobacterales;f__Enterobacteriaceae;g__Citrobacter;__ | 0 |
| d__Bacteria;p__Proteobacteria;c__Alphaproteobacteria;o__Caulobacterales;f__Caulobacteraceae;g__Brevundimonas;s__Brevundimonas_basaltis | 0 |
| d__Bacteria;p__Actinobacteriota;c__Actinobacteria;o__Propionibacteriales;f__Nocardioidaceae;g__Aeromicrobium;s__uncultured_Aeromicrobium | 0 |
| d__Bacteria;p__Firmicutes;c__Bacilli;o__Lactobacillales;f__Streptococcaceae;g__Streptococcus;s__Streptococcus_australis | 0 |
| d__Bacteria;p__Proteobacteria;c__Gammaproteobacteria;o__Xanthomonadales;f__Xanthomonadaceae;g__Lysobacter;s__Lysobacter_panacisoli | 0 |
| d__Bacteria;p__Actinobacteriota;c__Thermoleophilia;o__Solirubrobacterales;f__Solirubrobacteraceae;g__Patulibacter;__ | 0 |
| d__Bacteria;p__Actinobacteriota;c__Actinobacteria;o__Micrococcales;f__Promicromonosporaceae;g__Promicromonospora;__ | 0 |
| d__Bacteria;p__Proteobacteria;c__Gammaproteobacteria;o__Burkholderiales;f__Alcaligenaceae;g__Pusillimonas;__ | 0 |
| d__Bacteria;p__Proteobacteria;c__Gammaproteobacteria;o__Diplorickettsiales;f__Diplorickettsiaceae;g__Aquicella;s__uncultured_gamma | 0 |
| d__Bacteria;p__Firmicutes;c__Bacilli;o__Staphylococcales;f__Staphylococcaceae;g__Staphylococcus;s__Staphylococcus_caprae | 0 |
| d__Bacteria;p__Proteobacteria;c__Gammaproteobacteria;o__Burkholderiales;f__Alcaligenaceae;g__Castellaniella;s__Castellaniella_defragrans | 0 |
| d__Bacteria;p__Firmicutes;c__Desulfitobacteriia;o__Desulfitobacteriales;f__Desulfitobacteriaceae;g__Desulfosporosinus;s__Desulfosporosinus_orientis | 0 |
| d__Bacteria;p__Firmicutes;c__Clostridia;o__Clostridiales;f__Clostridiaceae;g__Proteiniclasticum;s__uncultured_bacterium | 0 |
| d__Bacteria;p__Firmicutes;c__Clostridia;o__Oscillospirales;f__Oscillospiraceae;g__Colidextribacter;s__uncultured_bacterium | 0 |
| d__Bacteria;p__Actinobacteriota;c__Actinobacteria;o__Catenulisporales;f__Actinospicaceae;g__Actinospica;s__bacterium_Ellin5012 | 0 |
| d__Bacteria;p__Proteobacteria;c__Gammaproteobacteria;o__Burkholderiales;f__Burkholderiaceae;g__Burkholderia-Caballeronia-Paraburkholderia;s__Burkholderia_territorii | 0 |
| d__Bacteria;p__Firmicutes;c__Bacilli;o__Thermoactinomycetales;f__Thermoactinomycetaceae;g__Planifilum;s__uncultured_compost | 0 |
| d__Bacteria;p__Actinobacteriota;c__Actinobacteria;o__Micrococcales;f__Microbacteriaceae;g__Clavibacter;__ | 0 |
| d__Bacteria;p__Proteobacteria;c__Gammaproteobacteria;o__Burkholderiales;f__Burkholderiaceae;g__Polynucleobacter;s__uncultured_bacterium | 0 |
| d__Bacteria;p__Firmicutes;c__Clostridia;o__Oscillospirales;f__Ruminococcaceae;g__Subdoligranulum;s__uncultured_bacterium | 0 |
| d__Bacteria;p__Actinobacteriota;c__Actinobacteria;o__Pseudonocardiales;f__Pseudonocardiaceae;g__Actinomycetospora;__ | 0 |
| d__Bacteria;p__Proteobacteria;c__Gammaproteobacteria;o__Enterobacterales;f__Enterobacteriaceae;g__Enterobacter;s__Enterobacter_sp. | 0 |
| d__Bacteria;p__Proteobacteria;c__Gammaproteobacteria;o__Burkholderiales;f__Rhodocyclaceae;g__Methyloversatilis;s__iron-reducing_bacterium | 0 |
| d__Bacteria;p__Cyanobacteria;c__Cyanobacteriia;o__Chloroplast;f__Chloroplast;g__Chloroplast;s__Arachis_hypogaea | 0 |
| d__Bacteria;p__Firmicutes;c__TSAC18;o__TSAC18;f__TSAC18;g__TSAC18;__ | 0 |
| d__Bacteria;p__Actinobacteriota;c__Actinobacteria;o__Streptosporangiales;f__Thermomonosporaceae;__;__ | 0 |
| d__Bacteria;p__Firmicutes;c__Bacilli;o__Paenibacillales;f__Paenibacillaceae;g__Cohnella;s__Cohnella_cellulosilytica | 0 |
| d__Bacteria;p__Actinobacteriota;c__Actinobacteria;o__Propionibacteriales;f__Nocardioidaceae;g__Nocardioides;s__Nocardioides_iriomotensis | 0 |
| d__Bacteria;p__Proteobacteria;c__Alphaproteobacteria;o__Rhizobiales;f__Devosiaceae;g__Devosia;s__Devosia_albogilva | 0 |
| d__Bacteria;p__Proteobacteria;c__Gammaproteobacteria;o__Enterobacterales;f__Enterobacteriaceae;g__Escherichia-Shigella;s__uncultured_Escherichia | 0 |
| d__Bacteria;p__Proteobacteria;c__Alphaproteobacteria;o__Rhizobiales;f__Xanthobacteraceae;g__Rhodoplanes;s__uncultured_Alphaproteobacteria | 0 |
| d__Bacteria;p__Firmicutes;c__Bacilli;o__Bacillales;f__Planococcaceae;g__Rummeliibacillus;s__Rummeliibacillus_stabekisii | 0 |
| d__Bacteria;p__Chloroflexi;c__Anaerolineae;o__SBR1031;f__A4b;g__A4b;s__metagenome | 0 |
| d__Bacteria;p__Actinobacteriota;c__Actinobacteria;o__Micrococcales;f__Microbacteriaceae;g__Cryobacterium;s__Cryobacterium_sp. | 0 |
| d__Bacteria;p__Proteobacteria;c__Gammaproteobacteria;o__Burkholderiales;f__Comamonadaceae;g__Leptothrix;__ | 0 |
| d__Bacteria;p__Myxococcota;c__Polyangia;o__Polyangiales;f__Polyangiaceae;g__Polyangium;__ | 0 |
| d__Bacteria;p__Proteobacteria;c__Gammaproteobacteria;o__Methylococcales;f__Methylococcaceae;g__Methylococcus;s__uncultured_bacterium | 0 |
| d__Bacteria;p__Actinobacteriota;c__Actinobacteria;o__Streptosporangiales;f__Streptosporangiaceae;g__Acrocarpospora;__ | 0 |
| d__Bacteria;p__Firmicutes;c__Bacilli;o__Bacillales;f__Bacillaceae;g__Bacillus;s__Bacillus_glycinifermentans | 0 |
| d__Bacteria;p__Firmicutes;c__Clostridia;o__Clostridiales;f__Clostridiaceae;g__Clostridium_sensu_stricto_3;s__uncultured_bacterium | 0 |
| d__Bacteria;p__Proteobacteria;c__Gammaproteobacteria;o__Burkholderiales;f__Comamonadaceae;g__Rhodoferax;__ | 0 |
| d__Bacteria;p__Bacteroidota;c__Ignavibacteria;o__Ignavibacteriales;f__Ignavibacteriaceae;g__Ignavibacterium;__ | 0 |
| d__Bacteria;p__Patescibacteria;c__Microgenomatia;o__Candidatus_Gottesmanbacteria;f__Candidatus_Gottesmanbacteria;g__Candidatus_Gottesmanbacteria;s__uncultured_bacterium | 0 |
| d__Bacteria;p__Proteobacteria;c__Gammaproteobacteria;o__Methylococcales;f__Methylomonadaceae;g__Methylobacter;__ | 0 |
| d__Bacteria;p__Firmicutes;c__Bacilli;o__Bacillales;f__Bacillaceae;g__Anoxybacillus;s__Anoxybacillus_amylolyticus | 0 |
| d__Bacteria;p__Firmicutes;c__Thermoanaerobacteria;o__Thermoanaerobacterales;f__Thermoanaerobacteraceae;__;__ | 0 |
| d__Bacteria;p__Chloroflexi;c__Anaerolineae;o__Anaerolineales;f__Anaerolineaceae;g__Anaerolinea;__ | 0 |
| d__Bacteria;p__Firmicutes;c__Bacilli;o__Acholeplasmatales;f__Acholeplasmataceae;g__Acholeplasma;__ | 0 |
| d__Bacteria;p__Firmicutes;c__Dethiobacteria;o__Dethiobacterales;f__Dethiobacteraceae;g__uncultured;s__uncultured_prokaryote | 0 |
| d__Bacteria;p__Proteobacteria;c__Gammaproteobacteria;o__Burkholderiales;f__Rhodocyclaceae;g__Propionivibrio;__ | 0 |
| d__Bacteria;p__Bacteroidota;c__Bacteroidia;o__Bacteroidales;f__Bacteroidetes_BD2-2;g__Bacteroidetes_BD2-2;s__uncultured_bacterium | 0 |
| d__Bacteria;p__Proteobacteria;c__Gammaproteobacteria;o__Burkholderiales;f__Comamonadaceae;g__Ottowia;__ | 0 |
| d__Bacteria;p__Firmicutes;c__Clostridia;o__Caldicoprobacterales;f__Caldicoprobacteraceae;g__Caldicoprobacter;s__uncultured_Firmicutes | 0 |
| d__Bacteria;p__Proteobacteria;c__Gammaproteobacteria;o__Burkholderiales;f__Oxalobacteraceae;g__Herminiimonas;__ | 0 |
| d__Bacteria;p__Bacteroidota;c__Bacteroidia;o__Chitinophagales;f__Saprospiraceae;g__Lewinella;__ | 0 |
| d__Bacteria;p__Acidobacteriota;__;__;__;__;__ | 0 |
| d__Bacteria;p__Firmicutes;c__Bacilli;o__Thermoactinomycetales;f__Thermoactinomycetaceae;g__Laceyella;__ | 0 |
| d__Bacteria;p__Acidobacteriota;c__Acidobacteriae;o__Solibacterales;f__Solibacteraceae;g__Candidatus_Solibacter;s__metagenome | 0 |
| d__Bacteria;p__Proteobacteria;c__Gammaproteobacteria;o__Pseudomonadales;f__Pseudomonadaceae;g__Azotobacter;s__Azotobacter_vinelandii | 0 |
| d__Bacteria;p__Verrucomicrobiota;c__Omnitrophia;o__Omnitrophales;f__Omnitrophaceae;g__Candidatus_Omnitrophus;__ | 0 |
| d__Bacteria;p__Sumerlaeota;c__Sumerlaeia;o__Sumerlaeales;f__Sumerlaeaceae;g__Sumerlaea;s__uncultured_organism | 0 |
| d__Bacteria;p__Firmicutes;c__Thermoanaerobacteria;o__Caldicellulosiruptorales;f__Caldicellulosiraptoraceae;g__Caldicellulosiruptor;__ | 0 |
| d__Bacteria;p__Proteobacteria;c__Gammaproteobacteria;o__Burkholderiales;f__Comamonadaceae;g__Caenimonas;s__uncultured_bacterium | 0 |
| d__Bacteria;p__Proteobacteria;c__Alphaproteobacteria;o__Rhizobiales;f__Rhizobiaceae;g__Aureimonas;__ | 0 |
| d__Bacteria;p__Patescibacteria;c__ABY1;o__Candidatus_Magasanikbacteria;f__Candidatus_Magasanikbacteria;g__Candidatus_Magasanikbacteria;s__uncultured_bacterium | 0 |
| d__Bacteria;p__Proteobacteria;c__Gammaproteobacteria;o__Methylococcales;f__Methylomonadaceae;__;__ | 0 |
| d__Bacteria;p__Bacteroidota;c__Bacteroidia;o__Bacteroidales;f__Marinilabiliaceae;g__Ruminofilibacter;__ | 0 |
| d__Bacteria;p__Firmicutes;c__Bacilli;o__Bacillales;f__Bacillaceae;g__Bacillus;s__Bacillus_sonorensis | 0 |
| d__Bacteria;p__Planctomycetota;c__Planctomycetes;o__Planctomycetales;f__uncultured;g__uncultured;s__metagenome | 0 |
| d__Bacteria;p__Planctomycetota;c__Pla3_lineage;o__Pla3_lineage;f__Pla3_lineage;g__Pla3_lineage;s__uncultured_bacterium | 0 |
| d__Bacteria;p__Methylomirabilota;c__Methylomirabilia;o__Methylomirabilales;f__Methylomirabilaceae;g__Sh765B-TzT-35;s__uncultured_bacterium | 0 |
| d__Bacteria;p__Proteobacteria;c__Gammaproteobacteria;o__Salinisphaerales;f__Solimonadaceae;g__Hydrocarboniphaga;__ | 0 |
| d__Bacteria;p__Bacteroidota;c__Bacteroidia;o__Bacteroidales;__;__;__ | 0 |
| d__Bacteria;p__Firmicutes;c__Bacilli;o__Bacillales;f__Sporolactobacillaceae;g__Tuberibacillus;__ | 0 |
| d__Bacteria;p__Patescibacteria;c__Microgenomatia;o__Candidatus_Roizmanbacteria;f__Candidatus_Roizmanbacteria;g__Candidatus_Roizmanbacteria;s__uncultured_soil | 0 |
| d__Bacteria;p__Chloroflexi;c__Anaerolineae;o__Ardenticatenales;f__Ardenticatenaceae;g__uncultured;__ | 0 |
| d__Bacteria;p__Actinobacteriota;c__Actinobacteria;o__Micrococcales;f__Microbacteriaceae;g__Diaminobutyricimonas;__ | 0 |
| d__Bacteria;p__Firmicutes;c__Desulfitobacteriia;o__Desulfitobacteriales;f__Heliobacteriaceae;g__Heliobacterium;s__Heliobacterium_modesticaldum | 0 |
| d__Bacteria;p__Verrucomicrobiota;c__Verrucomicrobiae;o__Methylacidiphilales;f__Methylacidiphilaceae;g__uncultured;__ | 0 |
| d__Bacteria;p__Proteobacteria;c__Gammaproteobacteria;o__Gammaproteobacteria_Incertae_Sedis;f__Unknown_Family;g__uncultured;s__uncultured_bacterium | 0 |
| d__Bacteria;p__Verrucomicrobiota;c__Verrucomicrobiae;o__Opitutales;f__Opitutaceae;g__Lacunisphaera;s__uncultured_soil | 0 |
| d__Bacteria;p__Proteobacteria;c__Alphaproteobacteria;o__Kordiimonadales;__;__;__ | 0 |
| d__Bacteria;p__Proteobacteria;c__Gammaproteobacteria;o__B2M28;f__B2M28;g__B2M28;s__uncultured_bacterium | 0 |
| d__Bacteria;p__Chloroflexi;c__Dehalococcoidia;o__Dehalococcoidales;f__uncultured;g__uncultured;__ | 0 |
| d__Bacteria;p__Desulfobacterota;c__Desulfobulbia;o__Desulfobulbales;f__Desulfobulbaceae;g__Desulfobulbus;__ | 0 |
| d__Bacteria;p__Proteobacteria;c__Alphaproteobacteria;o__Kiloniellales;f__Kiloniellaceae;g__uncultured;__ | 0 |
| d__Bacteria;p__Proteobacteria;c__Gammaproteobacteria;o__Pseudomonadales;f__Pseudomonadaceae;g__Pseudomonas;s__Pseudomonas_thermotolerans | 0 |
| d__Bacteria;p__Proteobacteria;c__Gammaproteobacteria;o__Gammaproteobacteria_Incertae_Sedis;f__Unknown_Family;g__uncultured;__ | 0 |
| d__Bacteria;p__Verrucomicrobiota;c__Verrucomicrobiae;o__Opitutales;f__Puniceicoccaceae;g__Verruc-01;s__Opitutus_sp. | 0 |
| d__Bacteria;p__Firmicutes;c__Bacilli;o__Bacillales;f__Sporolactobacillaceae;g__Sporolactobacillus;s__uncultured_bacterium | 0 |
| d__Bacteria;p__Spirochaetota;c__Spirochaetia;o__Spirochaetales;f__Spirochaetaceae;g__Spirochaeta;s__uncultured_organism | 0 |
| d__Bacteria;p__Verrucomicrobiota;c__Verrucomicrobiae;o__Opitutales;f__Opitutaceae;g__Opitutus;s__Opitutus_terrae | 0 |
| d__Bacteria;p__Firmicutes;c__Bacilli;o__Paenibacillales;f__Paenibacillaceae;g__Paenibacillus;s__Paenibacillus_turicensis | 0 |
| d__Bacteria;p__Proteobacteria;c__Alphaproteobacteria;o__Rickettsiales;f__SM1B06;g__SM1B06;__ | 0 |
| d__Bacteria;p__Verrucomicrobiota;c__Verrucomicrobiae;o__Pedosphaerales;f__Pedosphaeraceae;g__Oikopleura;__ | 0 |
| d__Bacteria;p__Bacteroidota;c__Ignavibacteria;o__SJA-28;f__SJA-28;g__SJA-28;__ | 0 |
| d__Bacteria;p__Desulfobacterota;c__Desulfobacteria;o__Desulfatiglandales;f__Desulfatiglandaceae;g__Desulfatiglans;s__uncultured_bacterium | 0 |
| d__Bacteria;p__FW113;c__FW113;o__FW113;f__FW113;g__FW113;s__uncultured_organism | 0 |
| d__Bacteria;p__Bacteroidota;c__Bacteroidia;o__Sphingobacteriales;f__Sphingobacteriaceae;g__Olivibacter;__ | 0 |
| d__Bacteria;p__Acidobacteriota;c__Blastocatellia;o__DS-100;f__DS-100;g__DS-100;s__uncultured_Acidobacteria | 0 |
| d__Bacteria;p__Acidobacteriota;c__Blastocatellia;o__11-24;f__11-24;g__11-24;__ | 0 |
| d__Bacteria;p__Proteobacteria;c__Gammaproteobacteria;o__Pseudomonadales;f__Alcanivoracaceae;g__Ketobacter;s__uncultured_proteobacterium | 0 |
| d__Bacteria;p__Chloroflexi;c__Ktedonobacteria;o__Ktedonobacterales;f__Ktedonobacteraceae;g__Thermogemmatispora;__ | 0 |
| d__Bacteria;p__Patescibacteria;c__Microgenomatia;o__Candidatus_Woykebacteria;f__Candidatus_Woykebacteria;g__Candidatus_Woykebacteria;s__uncultured_bacterium | 0 |
| d__Bacteria;p__Acidobacteriota;c__Holophagae;o__Subgroup_7;f__Subgroup_7;g__Subgroup_7;__ | 0 |
| d__Bacteria;p__Chloroflexi;c__Anaerolineae;o__SBR1031;f__A4b;g__OLB13;s__uncultured_bacterium | 0 |
| d__Bacteria;p__Cyanobacteria;c__Vampirivibrionia;o__Obscuribacterales;f__Obscuribacteraceae;g__Obscuribacteraceae;s__uncultured_bacterium | 0 |
| d__Bacteria;p__Spirochaetota;c__Spirochaetia;o__Spirochaetales;f__Spirochaetaceae;g__uncultured;s__uncultured_bacterium | 0 |
| d__Bacteria;p__Firmicutes;c__Clostridia;o__Thermincolales;f__Thermincolaceae;g__Thermincola;s__uncultured_Firmicutes | 0 |
| d__Bacteria;p__Proteobacteria;c__Gammaproteobacteria;o__Burkholderiales;f__Comamonadaceae;g__Curvibacter;__ | 0 |
| d__Bacteria;p__Proteobacteria;c__Gammaproteobacteria;o__Burkholderiales;f__Rhodocyclaceae;g__Georgfuchsia;__ | 0 |
| d__Bacteria;p__Planctomycetota;c__Planctomycetes;o__Pirellulales;f__Pirellulaceae;g__Pir4_lineage;s__uncultured_soil | 0 |
| d__Bacteria;p__Proteobacteria;c__Gammaproteobacteria;o__Enterobacterales;f__Yersiniaceae;g__Serratia;s__Serratia_marcescens | 0 |
| d__Bacteria;p__Firmicutes;c__Clostridia;o__Oscillospirales;f__Ruminococcaceae;g__Paludicola;s__uncultured_bacterium | 0 |
| d__Bacteria;p__Spirochaetota;c__Spirochaetia;o__Spirochaetales;f__Spirochaetaceae;g__M2PT2-76_termite_group;s__uncultured_spirochete | 0 |
| d__Bacteria;p__Planctomycetota;c__Phycisphaerae;o__mle1-8;f__mle1-8;g__mle1-8;__ | 0 |
| d__Bacteria;p__Proteobacteria;c__Alphaproteobacteria;o__Caulobacterales;f__Caulobacteraceae;g__Phenylobacterium;s__Phenylobacterium_composti | 0 |
| d__Bacteria;p__Bacteroidota;c__Bacteroidia;o__Chitinophagales;f__37-13;g__37-13;s__uncultured_bacterium | 0 |
| d__Bacteria;p__Sumerlaeota;c__Sumerlaeia;o__uncultured;f__uncultured;g__uncultured;s__uncultured_bacterium | 0 |
| d__Bacteria;p__Bacteroidota;c__Ignavibacteria;o__Ignavibacteriales;f__SR-FBR-L83;g__SR-FBR-L83;s__uncultured_bacterium | 0 |
| d__Bacteria;p__Chloroflexi;c__Anaerolineae;o__Anaerolineales;f__Anaerolineaceae;__;__ | 0 |
| d__Bacteria;p__Margulisbacteria;c__Margulisbacteria;o__Margulisbacteria;f__Margulisbacteria;g__Margulisbacteria;s__uncultured_bacterium | 0 |
| d__Bacteria;p__Firmicutes;c__Clostridia;o__Oscillospirales;f__Oscillospiraceae;g__UCG-005;__ | 0 |
| d__Bacteria;p__Proteobacteria;c__Alphaproteobacteria;o__Rhizobiales;f__Rhizobiaceae;g__Neorhizobium;s__Neorhizobium_galegae | 0 |
| d__Bacteria;p__Myxococcota;c__Polyangia;o__Nannocystales;f__Nannocystaceae;g__Nannocystis;s__uncultured_bacterium | 0 |
| d__Bacteria;p__Proteobacteria;c__Gammaproteobacteria;o__Burkholderiales;f__Comamonadaceae;g__Polaromonas;__ | 0 |
| d__Bacteria;p__Firmicutes;c__Clostridia;o__Clostridiales;f__Clostridiaceae;g__Clostridium_sensu_stricto_10;s__unidentified_marine | 0 |
| d__Bacteria;p__Proteobacteria;c__Alphaproteobacteria;o__Sphingomonadales;f__Sphingomonadaceae;g__Sphingomonas;s__Sphingomonas_leidyi | 0 |
| d__Bacteria;p__Patescibacteria;c__Parcubacteria;o__Parcubacteria;f__Parcubacteria;g__Parcubacteria;s__uncultured_bacterium | 0 |
| d__Bacteria;p__Patescibacteria;c__Parcubacteria;o__Candidatus_Spechtbacteria;f__Candidatus_Spechtbacteria;g__Candidatus_Spechtbacteria;s__uncultured_bacterium | 0 |
| d__Bacteria;p__Actinobacteriota;c__Actinobacteria;o__Pseudonocardiales;f__Pseudonocardiaceae;g__Crossiella;__ | 0 |
| d__Bacteria;p__Firmicutes;c__Bacilli;o__Alicyclobacillales;f__Alicyclobacillaceae;g__Alicyclobacillus;s__Alicyclobacillus_sp. | 0 |
| d__Bacteria;p__Firmicutes;c__Thermoanaerobacteria;o__Thermoanaerobacterales;f__Family_III;g__Thermoanaerobacterium;s__Thermoanaerobacterium_sp. | 0 |
| d__Bacteria;p__Bacteroidota;c__Bacteroidia;o__Bacteroidales;f__Bacteroidetes_BD2-2;g__Bacteroidetes_BD2-2;__ | 0 |
| d__Bacteria;p__Firmicutes;c__Bacilli;o__Bacillales;f__Bacillaceae;g__Bacillus;s__Bacillus_pseudomycoides | 0 |
| d__Bacteria;p__Proteobacteria;c__Alphaproteobacteria;o__Parvibaculales;f__Parvibaculaceae;g__Parvibaculum;s__uncultured_bacterium | 0 |
| d__Bacteria;p__Bacteroidota;c__Bacteroidia;o__Cytophagales;f__Spirosomaceae;g__Lacihabitans;__ | 0 |
| d__Bacteria;p__Proteobacteria;c__Gammaproteobacteria;o__Pseudomonadales;f__Porticoccaceae;g__Porticoccus;__ | 0 |
| d__Bacteria;p__Proteobacteria;c__Gammaproteobacteria;o__Steroidobacterales;f__Woeseiaceae;g__JTB255_marine_benthic_group;s__uncultured_bacterium | 0 |
| d__Bacteria;p__Patescibacteria;c__Microgenomatia;o__Candidatus_Roizmanbacteria;f__Candidatus_Roizmanbacteria;g__Candidatus_Roizmanbacteria;s__Candidatus_Roizmanbacteria | 0 |
| d__Bacteria;p__Proteobacteria;c__Alphaproteobacteria;o__Sphingomonadales;f__Sphingomonadaceae;g__Altererythrobacter;s__Porphyrobacter_sp. | 0 |
| d__Bacteria;p__Firmicutes;c__Desulfotomaculia;o__Desulfotomaculales;f__Desulfurisporaceae;g__Desulfofundulus;__ | 0 |
| d__Bacteria;p__Bacteroidota;c__Bacteroidia;o__Flavobacteriales;f__Cryomorphaceae;g__uncultured;__ | 0 |
| d__Bacteria;p__Proteobacteria;c__Alphaproteobacteria;o__Azospirillales;f__Azospirillaceae;g__Azospirillum;s__Azospirillum_thiophilum | 0 |
| d__Bacteria;p__Zixibacteria;c__Zixibacteria;o__Zixibacteria;f__Zixibacteria;g__Zixibacteria;__ | 0 |
| d__Bacteria;p__Chloroflexi;c__Anaerolineae;o__SBR1031;f__A4b;g__A4b;s__uncultured_organism | 0 |
| d__Bacteria;p__Bacteroidota;c__Bacteroidia;o__Cytophagales;f__Microscillaceae;g__Hassallia;s__uncultured_bacterium | 0 |
| d__Bacteria;p__Myxococcota;c__Myxococcia;o__Myxococcales;f__Myxococcaceae;g__KD3-10;s__uncultured_bacterium | 0 |
| d__Bacteria;p__Proteobacteria;c__Alphaproteobacteria;o__Thalassobaculales;f__Thalassobaculaceae;g__Thalassobaculum;s__uncultured_bacterium | 0 |
| d__Bacteria;p__Bacteroidota;c__Bacteroidia;o__Chitinophagales;f__Saprospiraceae;g__Lewinella;s__uncultured_bacterium | 0 |
| d__Bacteria;p__Patescibacteria;c__Gracilibacteria;o__Absconditabacteriales_(SR1);f__Absconditabacteriales_(SR1);g__Absconditabacteriales_(SR1);__ | 0 |
| d__Bacteria;p__Bdellovibrionota;c__Oligoflexia;o__Oligoflexales;f__Oligoflexaceae;__;__ | 0 |
| d__Bacteria;p__Bacteroidota;c__Bacteroidia;o__Bacteroidales;f__Bacteroidaceae;g__Bacteroides;s__Bacteroides_vulgatus | 0 |
| d__Bacteria;p__Proteobacteria;c__Alphaproteobacteria;o__Rhizobiales;f__Methyloligellaceae;g__Methyloligella;__ | 0 |
| d__Bacteria;p__Cyanobacteria;c__Cyanobacteriia;o__Chloroplast;f__Chloroplast;g__Chloroplast;s__Lupinus_angustifolius | 0 |
| d__Bacteria;p__Cyanobacteria;c__Cyanobacteriia;o__Chloroplast;f__Chloroplast;g__Chloroplast;s__Lotus_corniculatus | 0 |
| d__Bacteria;p__Proteobacteria;c__Alphaproteobacteria;o__Micropepsales;f__Micropepsaceae;g__uncultured;s__Rhizobiales_bacterium | 0 |
| d__Bacteria;p__Proteobacteria;c__Gammaproteobacteria;o__Pseudomonadales;f__Alcanivoracaceae1;g__Alcanivorax;__ | 0 |
| d__Bacteria;p__Bacteroidota;c__Bacteroidia;o__Sphingobacteriales;__;__;__ | 0 |
| d__Bacteria;p__Actinobacteriota;c__Actinobacteria;o__Micrococcales;f__Microbacteriaceae;g__Humibacter;s__Humibacter_ginsengisoli | 0 |
| d__Bacteria;p__Cyanobacteria;c__Cyanobacteriia;o__Chloroplast;f__Chloroplast;g__Chloroplast;s__Phaseolus_acutifolius | 0 |
| d__Bacteria;p__Firmicutes;c__Bacilli;o__Bacillales;f__Bacillaceae;g__Oceanobacillus;s__Oceanobacillus_sp. | 0 |
| d__Bacteria;p__Proteobacteria;c__Alphaproteobacteria;o__Rhodospirillales;f__Magnetospirillaceae;g__Telmatospirillum;__ | 0 |
| d__Bacteria;p__Firmicutes;c__Clostridia;o__Peptostreptococcales-Tissierellales;f__Family_XI;g__uncultured;s__uncultured_bacterium | 0 |
| d__Bacteria;p__Firmicutes;c__Clostridia;o__Peptostreptococcales-Tissierellales;f__Thermotaleaceae;g__Geosporobacter;s__Geosporobacter_ferrireducens | 0 |
| d__Bacteria;p__Actinobacteriota;c__Actinobacteria;o__Micrococcales;f__Microbacteriaceae;g__Amnibacterium;__ | 0 |
| d__Bacteria;p__Proteobacteria;c__Alphaproteobacteria;o__Reyranellales;f__Reyranellaceae;g__Reyranella;s__uncultured_Alphaproteobacteria | 0 |
| d__Bacteria;p__Actinobacteriota;c__Actinobacteria;o__Catenulisporales;f__Actinospicaceae;g__Actinospica;s__Actinospica_robiniae | 0 |
| d__Bacteria;p__Firmicutes;c__Limnochordia;o__Limnochordia;f__Limnochordia;g__Limnochordia;__ | 0 |
| d__Bacteria;p__Firmicutes;c__Clostridia;o__Oscillospirales;f__Oscillospiraceae;g__Flavonifractor;s__Flavonifractor_plautii | 0 |
| d__Bacteria;p__Firmicutes;c__Bacilli;o__Bacillales;f__Bacillaceae;g__Piscibacillus;__ | 0 |
| d__Bacteria;p__Firmicutes;c__Clostridia;o__Clostridiales;f__Clostridiaceae;g__Sarcina;s__uncultured_bacterium | 0 |
| d__Bacteria;p__Proteobacteria;c__Alphaproteobacteria;o__Rickettsiales;f__Rickettsiaceae;g__uncultured;__ | 0 |
| d__Bacteria;p__Proteobacteria;c__Alphaproteobacteria;o__Rhizobiales;f__Xanthobacteraceae;g__Pseudolabrys;s__Pseudolabrys_taiwanensis | 0 |
| d__Bacteria;p__Actinobacteriota;c__Actinobacteria;o__Micrococcales;f__Microbacteriaceae;g__uncultured;s__Diaminobutyricimonas_sp. | 0 |
| d__Bacteria;p__Planctomycetota;c__Planctomycetes;o__Isosphaerales;f__Isosphaeraceae;g__Isosphaeraceae;s__uncultured_planctomycete | 0 |
| d__Bacteria;p__Firmicutes;c__Negativicutes;o__Veillonellales-Selenomonadales;f__Sporomusaceae;g__Pelosinus;s__uncultured_bacterium | 0 |
| d__Bacteria;p__Spirochaetota;c__Spirochaetia;o__Spirochaetales;f__Spirochaetaceae;g__Spirochaeta_2;s__uncultured_bacterium | 0 |
| d__Bacteria;p__Firmicutes;c__Bacilli;o__Bacillales;f__Planococcaceae;g__Psychrobacillus;__ | 0 |
| d__Bacteria;p__Actinobacteriota;c__Actinobacteria;o__Streptosporangiales;f__Streptosporangiaceae;g__Microbispora;__ | 0 |
| d__Bacteria;p__Firmicutes;c__Clostridia;o__Lachnospirales;f__Lachnospiraceae;g__[Eubacterium]_hallii_group;__ | 0 |
| d__Bacteria;p__Patescibacteria;c__Microgenomatia;o__Candidatus_Pacebacteria;f__Candidatus_Pacebacteria;g__Candidatus_Pacebacteria;__ | 0 |
| d__Bacteria;p__Firmicutes;c__Clostridia;o__Oscillospirales;f__Ruminococcaceae;g__[Eubacterium]_siraeum_group;__ | 0 |
| d__Bacteria;p__Acidobacteriota;c__Acidobacteriae;o__Acidobacteriales;f__Acidobacteriaceae_(Subgroup_1);g__Edaphobacter;s__uncultured_bacterium | 0 |
| d__Bacteria;p__Planctomycetota;c__Planctomycetes;o__Gemmatales;f__Gemmataceae;g__Gemmata;s__uncultured_Planctomycetales | 0 |
| d__Bacteria;p__Firmicutes;c__Clostridia;o__Clostridiales;f__Caloramatoraceae;g__Caloramator;__ | 0 |
| d__Bacteria;p__Proteobacteria;c__Alphaproteobacteria;o__Ferrovibrionales;f__Ferrovibrionales;g__Taonella;s__uncultured_bacterium | 0 |
| d__Bacteria;p__Desulfobacterota;c__Desulfuromonadia;o__Geobacterales;f__Geobacteraceae;__;__ | 0 |
| d__Bacteria;p__Proteobacteria;c__Alphaproteobacteria;o__Thalassobaculales;f__uncultured;g__uncultured;s__uncultured_proteobacterium | 0 |
| d__Bacteria;p__Bacteroidota;c__Bacteroidia;o__Bacteroidales;f__Marinilabiliaceae;g__[Cytophaga]_xylanolytica_group;s__uncultured_Bacteroidetes | 0 |
| d__Bacteria;p__Fibrobacterota;c__Fibrobacteria;o__Fibrobacterales;f__Fibrobacterales;g__BBMC-4;s__uncultured_microorganism | 0 |
| d__Bacteria;p__Verrucomicrobiota;c__Verrucomicrobiae;o__Chthoniobacterales;f__01D2Z36;g__01D2Z36;__ | 0 |
| d__Bacteria;p__Proteobacteria;c__Gammaproteobacteria;o__Enterobacterales;f__Morganellaceae;g__Morganella;s__Morganella_morganii | 0 |
| d__Bacteria;p__RCP2-54;c__RCP2-54;o__RCP2-54;f__RCP2-54;g__RCP2-54;__ | 0 |
| d__Bacteria;p__Bacteroidota;c__Bacteroidia;o__Sphingobacteriales;f__Lentimicrobiaceae;g__Lentimicrobiaceae;s__uncultured_bacterium | 0 |
| d__Bacteria;p__Actinobacteriota;c__Actinobacteria;o__Streptomycetales;f__Streptomycetaceae;g__Streptomyces;s__Streptomyces_griseorubiginosus | 0 |
| d__Bacteria;p__Actinobacteriota;c__Acidimicrobiia;o__Microtrichales;f__Iamiaceae;g__Iamia;s__metagenome | 0 |
| d__Bacteria;p__Proteobacteria;c__Gammaproteobacteria;o__R7C24;f__R7C24;g__R7C24;s__uncultured_compost | 0 |
| d__Bacteria;p__Proteobacteria;c__Alphaproteobacteria;o__Rhizobiales;f__Devosiaceae;g__uncultured;s__uncultured_bacterium | 0 |
| d__Bacteria;p__Proteobacteria;c__Gammaproteobacteria;o__Pseudomonadales;f__Spongiibacteraceae;g__BD1-7_clade;s__uncultured_bacterium | 0 |
| d__Bacteria;p__Proteobacteria;c__Gammaproteobacteria;o__Burkholderiales;f__Rhodocyclaceae;g__Sulfuritalea;s__uncultured_bacterium | 0 |
| d__Bacteria;p__Proteobacteria;c__Gammaproteobacteria;o__Methylococcales;f__Methylococcaceae;g__uncultured;s__uncultured_compost | 0 |
| d__Bacteria;p__Firmicutes;c__Symbiobacteriia;o__Symbiobacteriales;f__Symbiobacteriales;g__Caldinitratiruptor;__ | 0 |
| d__Bacteria;p__Firmicutes;c__Bacilli;o__Lactobacillales;f__Lactobacillaceae;g__Loigolactobacillus;s__Lactobacillus_coryniformis | 0 |
| d__Bacteria;p__Proteobacteria;c__Gammaproteobacteria;o__Ectothiorhodospirales;__;__;__ | 0 |
| d__Bacteria;p__Planctomycetota;c__Phycisphaerae;o__MSBL9;f__GWC2-45-44;g__GWC2-45-44;s__Planctomycetes_bacterium | 0 |
| d__Bacteria;p__Bacteroidota;c__Bacteroidia;o__Chitinophagales;f__Saprospiraceae;__;__ | 0 |
| d__Bacteria;p__Planctomycetota;c__Planctomycetes;o__uncultured;f__uncultured;g__uncultured;s__uncultured_planctomycete | 0 |
| d__Bacteria;p__Proteobacteria;c__Alphaproteobacteria;o__Ferrovibrionales;f__Ferrovibrionaceae;g__Ferrovibrio;s__Ferrovibrio_denitrificans | 0 |
| d__Bacteria;p__Proteobacteria;c__Alphaproteobacteria;o__Defluviicoccales;f__uncultured;g__uncultured;s__uncultured_bacterium | 0 |
| d__Bacteria;p__Patescibacteria;c__Parcubacteria;o__Candidatus_Yanofskybacteria;f__Candidatus_Yanofskybacteria;g__Candidatus_Yanofskybacteria;s__uncultured_bacterium | 0 |
| d__Bacteria;p__Proteobacteria;c__Alphaproteobacteria;o__Rhizobiales;f__Beijerinckiaceae;g__uncultured;s__metagenome | 0 |
| d__Bacteria;p__Proteobacteria;c__Alphaproteobacteria;o__Rickettsiales;f__Mitochondria;g__Mitochondria;s__Glycine_max | 0 |
| d__Bacteria;p__Myxococcota;c__Polyangia;o__PS-B29;f__PS-B29;g__PS-B29;s__uncultured_organism | 0 |
| d__Bacteria;p__Verrucomicrobiota;c__Verrucomicrobiae;o__Pedosphaerales;f__Pedosphaeraceae;g__DEV008;__ | 0 |
| d__Bacteria;p__Proteobacteria;c__Alphaproteobacteria;o__Sphingomonadales;f__Sphingomonadaceae;g__Sphingobium;s__Sphingobium_sp. | 0 |
| d__Bacteria;p__Proteobacteria;c__Alphaproteobacteria;o__Rhizobiales;f__Rhizobiaceae;g__Phyllobacterium;__ | 0 |
| d__Bacteria;p__Spirochaetota;__;__;__;__;__ | 0 |
| d__Bacteria;p__Verrucomicrobiota;c__Verrucomicrobiae;o__Pedosphaerales;f__Pedosphaeraceae;g__ADurb.Bin063-1;s__uncultured_bacterium | 0 |
| d__Bacteria;p__Actinobacteriota;c__Actinobacteria;o__Streptomycetales;f__Streptomycetaceae;g__Streptomyces;s__Streptomyces_leeuwenhoekii | 0 |
| d__Bacteria;p__Firmicutes;c__Negativicutes;o__Veillonellales-Selenomonadales;f__Sporomusaceae;g__Anaerosporomusa;__ | 0 |
| d__Bacteria;p__Proteobacteria;c__Gammaproteobacteria;o__Methylococcales;f__Methylococcaceae;g__Methylocaldum;s__Methylocaldum_tepidum | 0 |
| d__Bacteria;p__Elusimicrobiota;c__Lineage_IIb;o__Lineage_IIb;f__Lineage_IIb;g__Lineage_IIb;s__uncultured_soil | 0 |
| d__Bacteria;p__Proteobacteria;c__Alphaproteobacteria;o__Rhizobiales;f__Beijerinckiaceae;g__Methylosinus;s__Methylosinus_trichosporium | 0 |
| d__Bacteria;p__Planctomycetota;c__Phycisphaerae;o__Pla1_lineage;f__Pla1_lineage;g__Pla1_lineage;s__uncultured_organism | 0 |
| d__Bacteria;p__Caldatribacteriota;c__Caldatribacteriia;o__Caldatribacteriales;f__Caldatribacteriaceae;g__Candidatus_Caldatribacterium;s__uncultured_bacterium | 0 |
| d__Bacteria;p__Proteobacteria;c__Alphaproteobacteria;o__Rickettsiales;f__uncultured;g__uncultured;__ | 0 |
| d__Bacteria;p__Proteobacteria;c__Alphaproteobacteria;o__Rhizobiales;f__Rhizobiaceae;g__Mesorhizobium;s__Mesorhizobium_plurifarium | 0 |
| d__Bacteria;p__Planctomycetota;c__Phycisphaerae;o__Phycisphaerales;f__Phycisphaeraceae;g__SM1A02;s__uncultured_planctomycete | 0 |
| d__Bacteria;p__Proteobacteria;c__Gammaproteobacteria;o__Burkholderiales;f__Alcaligenaceae;g__Derxia;s__uncultured_Derxia | 0 |
| d__Bacteria;p__Planctomycetota;c__Planctomycetes;o__Pirellulales;f__Pirellulaceae;g__Rhodopirellula;s__uncultured_hydrocarbon | 0 |
| d__Bacteria;p__Proteobacteria;c__Alphaproteobacteria;o__Rhodospirillales;__;__;__ | 0 |
| d__Bacteria;p__Firmicutes;c__Bacilli;o__Paenibacillales;f__Paenibacillaceae;g__Thermobacillus;s__Firmicutes_bacterium | 0 |
| d__Bacteria;p__Proteobacteria;c__Gammaproteobacteria;o__Pseudomonadales;f__KI89A_clade;g__KI89A_clade;__ | 0 |
| d__Bacteria;p__Proteobacteria;c__Gammaproteobacteria;o__Methylococcales;f__Methylococcaceae;g__Methylocaldum;s__uncultured_bacterium | 0 |
| d__Bacteria;p__Myxococcota;c__Myxococcia;o__Myxococcales;f__Myxococcaceae;g__P3OB-42;s__uncultured_organism | 0 |
| d__Bacteria;p__MBNT15;c__MBNT15;o__MBNT15;f__MBNT15;g__MBNT15;s__uncultured_Desulfuromonadales | 0 |
| d__Bacteria;p__Proteobacteria;c__Alphaproteobacteria;o__Caulobacterales;f__Hyphomonadaceae;g__uncultured;s__uncultured_Alphaproteobacteria | 0 |
| d__Bacteria;p__Verrucomicrobiota;c__Verrucomicrobiae;o__Chthoniobacterales;f__Terrimicrobiaceae;g__Terrimicrobium;s__uncultured_bacterium | 0 |
| d__Bacteria;p__Proteobacteria;c__Alphaproteobacteria;o__Rhizobiales;f__Rhizobiaceae;g__Chelativorans;s__Chelativorans_composti | 0 |
| d__Bacteria;p__Firmicutes;c__Clostridia;o__Oscillospirales;f__Ethanoligenenaceae;g__Acetanaerobacterium;__ | 0 |
| d__Bacteria;p__Myxococcota;c__Polyangia;o__Polyangiales;f__BIrii41;g__BIrii41;s__uncultured_soil | 0 |
| d__Bacteria;p__Firmicutes;c__Bacilli;o__Paenibacillales;f__Paenibacillaceae;g__Paenibacillus;s__uncultured_Paenibacillaceae | 0 |
| d__Bacteria;p__Planctomycetota;c__Planctomycetes;o__Gemmatales;f__Gemmataceae;g__Gemmataceae;s__uncultured_bacterium | 0 |
| d__Bacteria;p__Proteobacteria;c__Alphaproteobacteria;o__Sphingomonadales;f__Sphingomonadaceae;g__Sphingopyxis;s__uncultured_bacterium | 0 |
| d__Bacteria;p__Proteobacteria;c__Alphaproteobacteria;o__Rhizobiales;f__Rhizobiales_Incertae_Sedis;g__Phreatobacter;s__metagenome | 0 |
| d__Bacteria;p__Proteobacteria;c__Alphaproteobacteria;o__Elsterales;f__Elsteraceae;g__uncultured;__ | 0 |
| d__Bacteria;p__Planctomycetota;c__Phycisphaerae;o__Tepidisphaerales;__;__;__ | 0 |
| d__Bacteria;p__Chloroflexi;c__Anaerolineae;o__SBR1031;f__SBR1031;g__SBR1031;s__uncultured_Chloroflexi | 0 |
| d__Bacteria;p__Bacteroidota;c__Bacteroidia;o__Flavobacteriales;f__NS9_marine_group;g__NS9_marine_group;__ | 0 |
| d__Bacteria;p__Chloroflexi;c__NLS2-31;o__NLS2-31;f__NLS2-31;g__NLS2-31;s__uncultured_bacterium | 0 |
| d__Bacteria;p__Chloroflexi;c__Chloroflexia;o__Thermomicrobiales;f__AKYG1722;g__AKYG1722;__ | 0 |
| d__Bacteria;p__Chloroflexi;c__Anaerolineae;o__SBR1031;f__A4b;g__A4b;s__uncultured_Chloroflexi | 0 |
| d__Bacteria;p__Bacteroidota;c__Bacteroidia;o__Cytophagales;f__Microscillaceae;g__OLB12;__ | 0 |
| d__Bacteria;p__Proteobacteria;c__Alphaproteobacteria;o__Rhizobiales;f__Hyphomicrobiaceae;g__Filomicrobium;s__uncultured_bacterium | 0 |
| d__Bacteria;p__Patescibacteria;c__Berkelbacteria;o__Berkelbacteria;f__Berkelbacteria;g__Berkelbacteria;__ | 0 |
| d__Bacteria;p__Proteobacteria;c__Alphaproteobacteria;o__Kiloniellales;f__Kiloniellaceae;__;__ | 0 |
| d__Bacteria;p__Proteobacteria;c__Alphaproteobacteria;o__Rhizobiales;f__Rhizobiaceae;g__Mesorhizobium;s__Mesorhizobium_ciceri | 0 |
| d__Bacteria;p__Proteobacteria;c__Alphaproteobacteria;o__Rhizobiales;f__Xanthobacteraceae;g__Tardiphaga;__ | 0 |
| d__Bacteria;p__Bdellovibrionota;c__Oligoflexia;o__053A03-B-DI-P58;f__053A03-B-DI-P58;g__053A03-B-DI-P58;s__uncultured_bacterium | 0 |
| d__Bacteria;p__Proteobacteria;c__Alphaproteobacteria;o__Caulobacterales;f__Hyphomonadaceae;g__Euryhalocaulis;s__Euryhalocaulis_caribicus | 0 |
| d__Bacteria;p__Actinobacteriota;c__Thermoleophilia;o__uncultured;f__uncultured;g__uncultured;s__uncultured_bacterium | 0 |
| d__Bacteria;p__Proteobacteria;c__Gammaproteobacteria;o__Burkholderiales;f__Comamonadaceae;g__Comamonas;s__Comamonas_testosteroni | 0 |
| d__Bacteria;p__Planctomycetota;c__Phycisphaerae;o__Phycisphaerales;f__Phycisphaeraceae;g__SM1A02;s__metagenome | 0 |
| d__Bacteria;p__Actinobacteriota;c__Actinobacteria;o__Micromonosporales;f__Micromonosporaceae;g__Micromonospora;s__Micromonospora_aurantiaca | 0 |
| d__Bacteria;p__Proteobacteria;c__Gammaproteobacteria;o__Burkholderiales;f__Alcaligenaceae;g__Bordetella;s__Bordetella_bronchiseptica | 0 |
| d__Bacteria;p__Chloroflexi;c__Chloroflexia;o__Chloroflexales;f__Roseiflexaceae;g__Kouleothrix;__ | 0 |
| d__Bacteria;p__Bacteroidota;c__Bacteroidia;o__Flavobacteriales;f__Flavobacteriaceae;g__Flavobacterium;s__Flavobacterium_sp. | 0 |
| d__Bacteria;p__Firmicutes;c__uncultured;o__uncultured;f__uncultured;g__uncultured;__ | 0 |
| d__Bacteria;p__Gemmatimonadota;c__Gemmatimonadetes;o__Gemmatimonadales;f__Gemmatimonadaceae;g__uncultured;s__uncultured_actinobacterium | 0 |
| d__Bacteria;p__Proteobacteria;c__Gammaproteobacteria;o__Burkholderiales;f__Burkholderiaceae;g__Cupriavidus;s__Cupriavidus_taiwanensis | 0 |
| d__Bacteria;p__Planctomycetota;c__Phycisphaerae;o__Tepidisphaerales;f__WD2101_soil_group;g__WD2101_soil_group;s__uncultured_planctomycete | 0 |
| d__Bacteria;p__Planctomycetota;c__Phycisphaerae;o__Phycisphaerales;f__Phycisphaeraceae;g__SM1A02;s__uncultured_Planctomycetales | 0 |
| d__Bacteria;p__Proteobacteria;c__Gammaproteobacteria;o__KF-JG30-C25;f__KF-JG30-C25;g__KF-JG30-C25;__ | 0 |
| d__Bacteria;p__Cyanobacteria;c__Cyanobacteriia;o__Chloroplast;f__Chloroplast;g__Chloroplast;s__Eupomatia_laurina | 0 |
| d__Bacteria;p__Gemmatimonadota;c__Gemmatimonadetes;o__Gemmatimonadales;f__Gemmatimonadaceae;g__Gemmatimonas;s__uncultured_Gemmatimonadetes | 0 |
| d__Bacteria;p__Bacteroidota;c__Bacteroidia;o__Bacteroidales;f__Prolixibacteraceae;g__Meniscus;s__uncultured_Bacteroidetes | 0 |
| d__Bacteria;p__Actinobacteriota;c__Actinobacteria;o__Micrococcales;f__Microbacteriaceae;g__Curtobacterium;__ | 0 |
| d__Bacteria;p__Proteobacteria;c__Gammaproteobacteria;o__Pseudomonadales;f__Moraxellaceae;g__Acinetobacter;s__Acinetobacter_junii | 0 |
| d__Bacteria;p__Firmicutes;c__Bacilli;o__Bacillales;f__Bacillaceae;g__Bacillus;s__Bacillus_aryabhattai | 0 |
| d__Bacteria;p__Proteobacteria;c__Alphaproteobacteria;o__Rhizobiales;f__Rhizobiaceae;g__Brucella;__ | 0 |
| d__Bacteria;p__Firmicutes;c__Desulfotomaculia;o__Desulfotomaculales;f__Desulfotomaculaceae;__;__ | 0 |
| d__Bacteria;p__Chloroflexi;c__Ktedonobacteria;o__Ktedonobacterales;f__Ktedonobacteraceae;g__Ktedonobacter;__ | 0 |
| d__Bacteria;p__Actinobacteriota;c__Thermoleophilia;o__Gaiellales;f__uncultured;g__uncultured;s__uncultured_actinobacterium | 0 |
| d__Bacteria;p__Bacteroidota;c__Bacteroidia;o__Chitinophagales;f__Chitinophagaceae;g__Edaphobaculum;s__uncultured_Bacteroidetes | 0 |
| d__Bacteria;p__Firmicutes;c__Bacilli;o__Desulfuribacillales;f__Desulfuribacillaceae;g__Desulfuribacillus;__ | 0 |
| d__Bacteria;p__Proteobacteria;c__Alphaproteobacteria;o__Acetobacterales;f__Acetobacteraceae;g__Acidocella;s__uncultured_bacterium | 0 |
| d__Bacteria;p__Firmicutes;c__Bacilli;o__Bacillales;f__Bacillaceae;g__Bacillus;s__Bacillus_hwajinpoensis | 0 |
| d__Bacteria;p__Proteobacteria;c__Gammaproteobacteria;o__Burkholderiales;f__Oxalobacteraceae;g__Massilia;s__Naxibacter_sp. | 0 |
| d__Bacteria;p__Actinobacteriota;c__Actinobacteria;o__Streptosporangiales;f__Nocardiopsaceae;g__Thermobifida;s__Thermobifida_fusca | 0 |
| d__Bacteria;p__Firmicutes;c__Bacilli;o__Staphylococcales;f__Staphylococcaceae;g__Staphylococcus;s__Staphylococcus_nepalensis | 0 |
| d__Bacteria;p__Acidobacteriota;c__Acidobacteriae;o__Acidobacteriales;f__Acidobacteriaceae_(Subgroup_1);g__Granulicella;s__metagenome | 0 |
| d__Bacteria;p__Bacteroidota;c__Bacteroidia;o__Chitinophagales;f__Chitinophagaceae;g__uncultured;s__uncultured_Terrimonas | 0 |
| d__Bacteria;p__Proteobacteria;c__Alphaproteobacteria;o__Parvibaculales;f__Parvibaculaceae;g__Rhodoligotrophos;s__uncultured_bacterium | 0 |
| d__Bacteria;p__Elusimicrobiota;c__Lineage_IIa;o__Lineage_IIa;f__Lineage_IIa;g__Lineage_IIa;s__uncultured_bacterium | 0 |
| d__Bacteria;p__Verrucomicrobiota;c__Verrucomicrobiae;o__Chthoniobacterales;f__Chthoniobacteraceae;g__LD29;s__uncultured_Xiphinematobacteriaceae | 0 |
| d__Bacteria;p__Firmicutes;c__Clostridia;o__Lachnospirales;f__Lachnospiraceae;g__28-4;s__Kineothrix_alysoides | 0 |
| d__Bacteria;p__Acidobacteriota;c__Subgroup_22;o__Subgroup_22;f__Subgroup_22;g__Subgroup_22;s__uncultured_bacterium | 0 |
| d__Bacteria;p__Proteobacteria;c__Gammaproteobacteria;o__Pseudomonadales;f__Halieaceae;__;__ | 0 |
| d__Bacteria;p__Firmicutes;c__Clostridia;o__Lachnospirales;f__Lachnospiraceae;g__Lachnospiraceae;s__uncultured_Clostridiaceae | 0 |
| d__Bacteria;p__Proteobacteria;c__Alphaproteobacteria;o__Rhizobiales;f__Rhizobiaceae;g__Chelativorans;s__uncultured_bacterium | 0 |
| d__Bacteria;p__Firmicutes;c__Bacilli;o__Lactobacillales;f__Lactobacillaceae;g__Limosilactobacillus;s__Lactobacillus_fermentum | 0 |
| d__Bacteria;p__Acidobacteriota;c__Vicinamibacteria;o__Vicinamibacterales;f__Vicinamibacteraceae;g__Vicinamibacter;__ | 0 |
| d__Bacteria;p__Proteobacteria;c__Alphaproteobacteria;o__Kiloniellales;f__Fodinicurvataceae;g__uncultured;s__uncultured_bacterium | 0 |
| d__Bacteria;p__Verrucomicrobiota;c__Verrucomicrobiae;o__Pedosphaerales;f__Pedosphaeraceae;g__uncultured;s__uncultured_Verrucomicrobia | 0 |
| d__Bacteria;p__Actinobacteriota;c__Actinobacteria;o__Micrococcales;f__Intrasporangiaceae;g__Lapillicoccus;__ | 0 |
| d__Bacteria;p__Desulfobacterota;c__Desulfuromonadia;o__PB19;f__PB19;g__PB19;s__metagenome | 0 |
| d__Bacteria;p__Firmicutes;c__Thermoanaerobacteria;o__Thermoanaerobacterales;f__Thermoanaerobacteraceae;g__Thermanaeromonas;__ | 0 |
| d__Bacteria;p__Proteobacteria;c__Alphaproteobacteria;o__Rhizobiales;f__Rhizobiales_Incertae_Sedis;g__Nordella;s__bacterium_enrichment | 0 |
| d__Bacteria;p__Bacteroidota;c__Bacteroidia;o__Flavobacteriales;f__NS9_marine_group;g__NS9_marine_group;s__metagenome | 0 |
| d__Bacteria;p__Proteobacteria;c__Gammaproteobacteria;o__Enterobacterales;f__Budviciaceae;g__Pragia;__ | 0 |
| d__Bacteria;p__Proteobacteria;c__Alphaproteobacteria;o__Rhizobiales;f__Devosiaceae;g__Devosia;s__Devosia_chinhatensis | 0 |
| d__Bacteria;p__Firmicutes;c__Desulfotomaculia;o__Desulfotomaculales;f__Desulfurisporaceae;__;__ | 0 |
| d__Bacteria;p__Firmicutes;c__Limnochordia;o__Limnochordia;f__Limnochordia;g__Hydrogenispora;s__uncultured_Clostridium | 0 |
| d__Bacteria;p__Proteobacteria;c__Alphaproteobacteria;o__Acetobacterales;f__Acetobacteraceae;g__Gluconacetobacter;__ | 0 |
| d__Bacteria;p__Actinobacteriota;c__Rubrobacteria;o__Rubrobacterales;f__Rubrobacteriaceae;g__Rubrobacter;s__uncultured_bacterium | 0 |
| d__Bacteria;p__Bacteroidota;c__Bacteroidia;o__Cytophagales;f__Hymenobacteraceae;g__Adhaeribacter;__ | 0 |
| d__Bacteria;p__Firmicutes;c__Thermoanaerobacteria;o__Caldicellulosiruptorales;f__uncultured;g__uncultured;s__uncultured_bacterium | 0 |
| d__Bacteria;p__Actinobacteriota;c__Actinobacteria;o__Micrococcales;f__Cellulomonadaceae;g__Cellulomonas;s__Cellulomonas_sp. | 0 |
| d__Bacteria;p__Firmicutes;c__Bacilli;o__Bacillales;f__Planococcaceae;g__Sporosarcina;s__Sporosarcina_ureae | 0 |
| d__Bacteria;p__Actinobacteriota;c__Actinobacteria;o__Micrococcales;f__Microbacteriaceae;g__Leucobacter;__ | 0 |
| d__Bacteria;p__Firmicutes;c__Bacilli;o__uncultured;f__uncultured;g__uncultured;__ | 0 |
| d__Bacteria;p__Hydrogenedentes;c__Hydrogenedentia;o__Hydrogenedentiales;f__Hydrogenedensaceae;g__Hydrogenedensaceae;s__metagenome | 0 |
| d__Bacteria;p__Firmicutes;c__Limnochordia;o__Limnochordia;f__Limnochordia;g__Hydrogenispora;s__uncultured_Firmicutes | 0 |
| d__Bacteria;p__Firmicutes;c__Desulfotomaculia;o__Desulfotomaculales;f__Desulfurisporaceae;g__SCADC1-2-3;__ | 0 |
| d__Bacteria;p__Firmicutes;c__Clostridia;o__Oscillospirales;f__Hungateiclostridiaceae;g__Ruminiclostridium;s__uncultured_Clostridiales | 0 |
| d__Bacteria;p__Actinobacteriota;c__Actinobacteria;o__Streptosporangiales;f__Thermomonosporaceae;g__[Streptomyces]_thermoautotrophicus;s__uncultured_bacterium | 0 |
| d__Bacteria;p__Firmicutes;c__Bacilli;o__Bacillales;f__Bacillaceae;g__Bacillus;s__Bacillus_flexus | 0 |
| d__Bacteria;p__Bacteroidota;c__Bacteroidia;o__Cytophagales;f__Cyclobacteriaceae;g__Imperialibacter;s__uncultured_bacterium | 0 |
| d__Bacteria;p__Actinobacteriota;c__Actinobacteria;o__Corynebacteriales;f__Corynebacteriaceae;g__Corynebacterium;__ | 0 |
| d__Bacteria;p__Verrucomicrobiota;c__Verrucomicrobiae;o__Opitutales;f__Opitutaceae;g__IMCC26134;s__uncultured_bacterium | 0 |
| d__Bacteria;p__Chloroflexi;c__Anaerolineae;o__Caldilineales;f__Caldilineaceae;g__Litorilinea;__ | 0 |
| d__Bacteria;p__Patescibacteria;c__Microgenomatia;o__Candidatus_Levybacteria;f__Candidatus_Levybacteria;g__Candidatus_Levybacteria;s__uncultured_soil | 0 |
| d__Bacteria;p__Firmicutes;c__Clostridia;o__Oscillospirales;f__Ruminococcaceae;g__Incertae_Sedis;__ | 0 |
| d__Bacteria;p__Bacteroidota;c__Bacteroidia;o__Chitinophagales;f__Chitinophagaceae;g__Rurimicrobium;__ | 0 |
| d__Bacteria;p__Firmicutes;c__Bacilli;o__Bacillales;f__Bacillaceae;g__Bacillus;s__Bacillus_horikoshii | 0 |
| d__Bacteria;p__Proteobacteria;c__Alphaproteobacteria;o__Rhizobiales;f__Rhizobiales_Incertae_Sedis;g__Bauldia;s__metagenome | 0 |
| d__Bacteria;p__Proteobacteria;c__Gammaproteobacteria;o__Burkholderiales;f__Comamonadaceae;g__Rivibacter;__ | 0 |
| d__Bacteria;p__Firmicutes;c__Clostridia;o__Oscillospirales;f__Oscillospiraceae;g__Sporobacter;s__uncultured_Firmicutes | 0 |
| d__Bacteria;p__Proteobacteria;c__Alphaproteobacteria;o__Rhizobiales;f__Pleomorphomonadaceae;__;__ | 0 |
| d__Bacteria;p__Firmicutes;c__Bacilli;o__Paenibacillales;f__Paenibacillaceae;g__Paenibacillus;s__Paenibacillus_ihbetae | 0 |
| d__Bacteria;p__Chloroflexi;c__Dehalococcoidia;o__Dehalococcoidales;f__uncultured;g__uncultured;s__uncultured_bacterium | 0 |
| d__Bacteria;p__Fibrobacterota;c__Fibrobacteria;o__Fibrobacterales;f__Fibrobacterales;g__BBMC-4;__ | 0 |
| d__Bacteria;p__Proteobacteria;c__Gammaproteobacteria;o__Burkholderiales;f__Comamonadaceae;g__Aquabacterium;s__uncultured_bacterium | 0 |
| d__Bacteria;p__Firmicutes;c__Thermoanaerobacteria;o__Thermoanaerobacterales;f__Family_III;g__Thermoanaerobacterium;s__Thermoanaerobacterium_thermostercoris | 0 |
| d__Bacteria;p__Firmicutes;c__Clostridia;o__Clostridiales;f__Clostridiaceae;g__Clostridium_sensu_stricto_7;s__Clostridium_thermopalmarium | 0 |
| d__Bacteria;p__Firmicutes;c__Bacilli;o__Thermoactinomycetales;f__Thermoactinomycetaceae;g__uncultured;s__uncultured_compost | 0 |
| d__Bacteria;p__Firmicutes;c__Clostridia;o__Oscillospirales;f__Oscillospiraceae;g__Intestinimonas;__ | 0 |
| d__Bacteria;p__Proteobacteria;c__Alphaproteobacteria;o__Rhizobiales;f__Beijerinckiaceae;g__1174-901-12;__ | 0 |
| d__Bacteria;p__Planctomycetota;c__Phycisphaerae;o__Phycisphaerales;f__Phycisphaeraceae;g__uncultured;s__uncultured_planctomycete | 0 |
| d__Bacteria;p__Proteobacteria;c__Alphaproteobacteria;o__Azospirillales;f__Azospirillaceae;g__Azospirillum;s__Azospirillum_canadense | 0 |
| d__Bacteria;p__Firmicutes;c__Bacilli;o__Aneurinibacillales;f__Aneurinibacillaceae;g__Aneurinibacillus;s__uncultured_bacterium | 0 |
| d__Bacteria;p__Chloroflexi;c__Chloroflexia;o__Thermomicrobiales;f__AKYG1722;g__AKYG1722;s__uncultured_Chloroflexi | 0 |
| d__Bacteria;p__Proteobacteria;c__Alphaproteobacteria;o__Rhizobiales;f__Xanthobacteraceae;g__Xanthobacter;s__Xanthobacter_agilis | 0 |
| d__Bacteria;p__Patescibacteria;c__Microgenomatia;o__Candidatus_Woesebacteria;f__Candidatus_Woesebacteria;g__Candidatus_Woesebacteria;__ | 0 |
| d__Bacteria;p__Firmicutes;c__Incertae_Sedis;o__Incertae_Sedis;f__Incertae_Sedis;g__Gelria;s__uncultured_bacterium | 0 |
| d__Bacteria;p__Proteobacteria;c__Gammaproteobacteria;o__Burkholderiales;f__B1-7BS;g__B1-7BS;s__uncultured_compost | 0 |
| d__Bacteria;p__Proteobacteria;c__Gammaproteobacteria;o__Burkholderiales;f__Nitrosomonadaceae;g__IS-44;__ | 0 |
| d__Bacteria;p__Acidobacteriota;c__Thermoanaerobaculia;o__Thermoanaerobaculales;f__Thermoanaerobaculaceae;g__Subgroup_10;s__uncultured_prokaryote | 0 |
| d__Bacteria;p__Myxococcota;c__Polyangia;o__Polyangiales;f__BIrii41;g__BIrii41;s__uncultured_Sorangiineae | 0 |
| d__Bacteria;p__Verrucomicrobiota;c__Lentisphaeria;o__SS1-B-02-17;f__SS1-B-02-17;g__SS1-B-02-17;s__uncultured_bacterium | 0 |
| d__Bacteria;p__Myxococcota;c__Myxococcia;o__Myxococcales;f__Myxococcaceae;g__P3OB-42;s__metagenome | 0 |
| d__Bacteria;p__Bacteroidota;c__Bacteroidia;o__Chitinophagales;f__Saprospiraceae;g__Portibacter;__ | 0 |
| d__Bacteria;p__Proteobacteria;c__Alphaproteobacteria;o__Rickettsiales;f__SM2D12;g__SM2D12;s__uncultured_organism | 0 |
| d__Bacteria;p__Actinobacteriota;c__Actinobacteria;o__Propionibacteriales;f__Propionibacteriaceae;g__Friedmanniella;__ | 0 |
| d__Bacteria;p__Proteobacteria;c__Gammaproteobacteria;o__Pseudomonadales;f__Moraxellaceae;g__Acinetobacter;s__Acinetobacter_haemolyticus | 0 |
| d__Bacteria;p__Chloroflexi;__;__;__;__;__ | 0 |
| d__Bacteria;p__Proteobacteria;c__Gammaproteobacteria;o__Enterobacterales;f__Enterobacteriaceae;g__Cronobacter;s__Cronobacter_sakazakii | 0 |
| d__Bacteria;p__Proteobacteria;c__Gammaproteobacteria;o__Burkholderiales;f__Oxalobacteraceae;g__Oxalobacter;__ | 0 |
| d__Bacteria;p__Myxococcota;c__Polyangia;o__mle1-27;f__mle1-27;g__mle1-27;s__uncultured_delta | 0 |
| d__Bacteria;p__Firmicutes;c__Bacilli;o__Lactobacillales;f__Lactobacillaceae;g__Companilactobacillus;__ | 0 |
| d__Bacteria;p__Bacteroidota;c__Bacteroidia;o__Bacteroidales;f__Marinilabiliaceae;g__[Cytophaga]_xylanolytica_group;s__uncultured_bacterium | 0 |
| d__Bacteria;p__Proteobacteria;c__Alphaproteobacteria;o__Tistrellales;f__Geminicoccaceae;g__Geminicoccus;s__uncultured_bacterium | 0 |
| d__Bacteria;p__Actinobacteriota;c__Actinobacteria;o__Propionibacteriales;f__Propionibacteriaceae;g__Cutibacterium;__ | 0 |
| d__Bacteria;p__Bacteroidota;c__Bacteroidia;o__SM1A07;f__SM1A07;g__SM1A07;__ | 0 |
| d__Bacteria;p__Verrucomicrobiota;c__Omnitrophia;o__Omnitrophales;f__Omnitrophales;g__Omnitrophales;__ | 0 |
| d__Bacteria;p__Proteobacteria;c__Gammaproteobacteria;o__Pseudomonadales;f__Pseudomonadaceae;g__Pseudomonas;s__Pseudomonas_putida | 0 |
| d__Bacteria;p__Firmicutes;c__Clostridia;o__Lachnospirales;f__Lachnospiraceae;g__Agathobacter;s__uncultured_bacterium | 0 |
| d__Bacteria;p__Firmicutes;c__Bacilli;o__Bacillales;f__Bacillaceae;g__Pontibacillus;__ | 0 |
| d__Bacteria;p__Proteobacteria;c__Gammaproteobacteria;o__Pseudomonadales;f__Moraxellaceae;g__Acinetobacter;s__Acinetobacter_sp. | 0 |
| d__Bacteria;p__Proteobacteria;c__Gammaproteobacteria;o__Burkholderiales;f__Oxalobacteraceae;g__Oxalicibacterium;__ | 0 |
| d__Bacteria;p__Proteobacteria;c__Gammaproteobacteria;o__Burkholderiales;f__Alcaligenaceae;g__Pusillimonas;s__uncultured_bacterium | 0 |
| d__Bacteria;p__Firmicutes;c__Clostridia;o__Clostridiales;f__uncultured;g__uncultured;s__uncultured_bacterium | 0 |
| d__Bacteria;p__Firmicutes;c__Clostridia;o__Clostridiales;f__Clostridiaceae;g__Clostridium_sensu_stricto_2;__ | 0 |
| d__Bacteria;p__Dependentiae;c__Babeliae;o__Babeliales;f__UBA12409;g__UBA12409;s__uncultured_Candidatus | 0 |
| d__Bacteria;p__Proteobacteria;c__Alphaproteobacteria;o__Rhodospirillales;f__Magnetospirillaceae;g__Telmatospirillum;s__uncultured_bacterium | 0 |
| d__Bacteria;p__Firmicutes;c__Clostridia;o__Peptostreptococcales-Tissierellales;__;__;__ | 0 |
| d__Bacteria;p__Proteobacteria;c__Gammaproteobacteria;o__Pseudomonadales;f__Moraxellaceae;g__Acinetobacter;s__Acinetobacter_tandoii | 0 |
| d__Bacteria;p__Proteobacteria;c__Gammaproteobacteria;o__Burkholderiales;f__Methylophilaceae;g__Methylotenera;s__uncultured_bacterium | 0 |
| d__Bacteria;p__Proteobacteria;c__Alphaproteobacteria;o__Reyranellales;f__Reyranellaceae;g__uncultured;s__uncultured_Alphaproteobacteria | 0 |
| d__Bacteria;p__Proteobacteria;c__Gammaproteobacteria;o__Burkholderiales;f__Alcaligenaceae;g__Eoetvoesia;__ | 0 |
| d__Bacteria;p__Proteobacteria;c__Gammaproteobacteria;o__Xanthomonadales;f__Rhodanobacteraceae;g__Rhodanobacter;s__uncultured_Rhodanobacter | 0 |
| d__Bacteria;p__Proteobacteria;c__Alphaproteobacteria;o__Caulobacterales;f__Caulobacteraceae;g__Phenylobacterium;s__Phenylobacterium_hankyongense | 0 |
| d__Bacteria;p__Proteobacteria;c__Gammaproteobacteria;o__Xanthomonadales;f__Rhodanobacteraceae;g__Rhodanobacter;s__Rhodanobacter_glycinis | 0 |
| d__Bacteria;p__Proteobacteria;c__Alphaproteobacteria;o__Ferrovibrionales;f__Ferrovibrionaceae;g__Ferrovibrio;s__Ferrovibrio_xuzhouensis | 0 |
| d__Bacteria;p__Bacteroidota;c__Bacteroidia;o__Flavobacteriales;f__Flavobacteriaceae;g__Flavobacterium;s__Flavobacterium_daejeonense | 0 |
| d__Bacteria;p__Proteobacteria;c__Gammaproteobacteria;o__Burkholderiales;f__Comamonadaceae;g__Delftia;__ | 0 |
| d__Bacteria;p__Proteobacteria;c__Gammaproteobacteria;o__Pseudomonadales;f__Cellvibrionaceae;g__Cellvibrio;s__Cellvibrio_sp. | 0 |
| d__Bacteria;p__Acidobacteriota;c__Acidobacteriae;o__PAUC26f;f__PAUC26f;g__PAUC26f;s__uncultured_bacterium | 0 |
| d__Bacteria;p__Bacteroidota;c__Bacteroidia;o__Cytophagales;f__Cyclobacteriaceae;g__Aquiflexum;__ | 0 |
| d__Bacteria;p__Proteobacteria;c__Gammaproteobacteria;o__Enterobacterales;f__Idiomarinaceae;g__Aliidiomarina;__ | 0 |
| d__Bacteria;p__Bacteroidota;c__Bacteroidia;o__Cytophagales;f__Cyclobacteriaceae;g__Belliella;__ | 0 |
| d__Bacteria;p__Bacteroidota;c__Rhodothermia;o__Balneolales;f__Balneolaceae;g__uncultured;__ | 0 |
| d__Bacteria;p__Proteobacteria;c__Gammaproteobacteria;o__Xanthomonadales;f__Rhodanobacteraceae;g__Luteibacter;s__uncultured_bacterium | 0 |
| d__Bacteria;p__Bacteroidota;c__Bacteroidia;o__Cytophagales;f__Cytophagaceae;g__Cytophaga;__ | 0 |
| d__Bacteria;p__Proteobacteria;c__Gammaproteobacteria;o__Pseudomonadales;f__Pseudohongiellaceae;g__Pseudohongiella;s__uncultured_bacterium | 0 |
| d__Bacteria;p__Firmicutes;c__Clostridia;o__Clostridiales;f__Clostridiaceae;g__Clostridium_sensu_stricto_6;s__uncultured_bacterium | 0 |
| d__Bacteria;p__Firmicutes;c__Clostridia;o__Clostridiales;f__Clostridiaceae;g__Clostridium_sensu_stricto_13;s__Clostridium_argentinense | 0 |
| d__Bacteria;p__Proteobacteria;c__Gammaproteobacteria;o__Ectothiorhodospirales;f__Ectothiorhodospiraceae;g__Thioalkalivibrio;__ | 0 |
| d__Bacteria;p__Proteobacteria;c__Gammaproteobacteria;o__Enterobacterales;f__Aeromonadaceae;g__Aeromonas;__ | 0 |
| d__Bacteria;p__Proteobacteria;c__Gammaproteobacteria;o__Pseudomonadales;f__Pseudohongiellaceae;__;__ | 0 |
| d__Bacteria;p__Firmicutes;c__Clostridia;o__Oscillospirales;f__UCG-010;g__UCG-010;s__uncultured_rumen | 0 |
| d__Bacteria;p__Proteobacteria;c__Gammaproteobacteria;o__Pseudomonadales;f__Pseudohongiellaceae;g__Pseudohongiella;__ | 0 |
| d__Bacteria;p__Bacteroidota;c__Bacteroidia;o__Cytophagales;f__Cyclobacteriaceae;g__Marivirga;__ | 0 |
| d__Bacteria;p__Verrucomicrobiota;c__Verrucomicrobiae;o__Opitutales;f__Opitutaceae;g__Diplosphaera;s__uncultured_bacterium | 0 |
| d__Bacteria;p__Proteobacteria;c__Gammaproteobacteria;o__Burkholderiales;f__Nitrosomonadaceae;g__mle1-7;__ | 0 |
| d__Bacteria;p__Bacteroidota;c__Bacteroidia;o__Chitinophagales;f__37-13;g__37-13;s__uncultured_Bacteroidetes | 0 |
| d__Bacteria;p__Proteobacteria;c__Alphaproteobacteria;o__Rhizobiales;f__Beijerinckiaceae;g__Salinarimonas;__ | 0 |
| d__Bacteria;p__Proteobacteria;c__Gammaproteobacteria;o__Burkholderiales;f__Comamonadaceae;g__Acidovorax;s__Acidovorax_delafieldii | 0 |
| d__Bacteria;p__Bacteroidota;c__Bacteroidia;o__Cytophagales;f__Spirosomaceae;g__Rudanella;__ | 0 |
| d__Bacteria;p__Actinobacteriota;c__Actinobacteria;o__Nitriliruptorales;f__Nitriliruptoraceae;g__Nitriliruptoraceae;s__uncultured_bacterium | 0 |
| d__Bacteria;p__Proteobacteria;c__Gammaproteobacteria;o__Burkholderiales;f__Alcaligenaceae;g__Castellaniella;s__uncultured_bacterium | 0 |
| d__Bacteria;p__Bdellovibrionota;c__Oligoflexia;o__Oligoflexales;f__Oligoflexaceae;g__Pseudobacteriovorax;__ | 0 |
| d__Bacteria;p__Proteobacteria;c__Gammaproteobacteria;o__Pseudomonadales;f__Pseudomonadaceae;g__Pseudomonas;s__Pseudomonas_anguilliseptica | 0 |
| d__Bacteria;p__Proteobacteria;c__Gammaproteobacteria;o__Burkholderiales;f__Nitrosomonadaceae;g__oc32;s__uncultured_bacterium | 0 |
| d__Bacteria;p__Proteobacteria;c__Gammaproteobacteria;o__Burkholderiales;f__Comamonadaceae;g__Hydrogenophaga;s__Hydrogenophaga_sp. | 0 |
| d__Bacteria;p__Proteobacteria;c__Gammaproteobacteria;o__Enterobacterales;f__Aeromonadaceae;g__Aeromonas;s__Aeromonas_caviae | 0 |
| d__Bacteria;p__Bacteroidota;c__Bacteroidia;o__Flavobacteriales;f__Flavobacteriaceae;g__Arenibacter;__ | 0 |
| d__Bacteria;p__Proteobacteria;c__Alphaproteobacteria;o__Rhizobiales;f__Rhizobiaceae;g__Shinella;s__Shinella_granuli | 0 |
| d__Bacteria;p__Proteobacteria;c__Gammaproteobacteria;o__Enterobacterales;f__Erwiniaceae;g__Pantoea;s__uncultured_bacterium | 0 |
| d__Bacteria;p__Proteobacteria;c__Gammaproteobacteria;o__Burkholderiales;f__Oxalobacteraceae;g__Oxalicibacterium;s__Oxalicibacterium_solurbis | 0 |
| d__Bacteria;p__Proteobacteria;c__Gammaproteobacteria;o__Pseudomonadales;f__Pseudomonadaceae;g__Pseudomonas;s__Pseudomonas_peli | 0 |
| d__Bacteria;p__Firmicutes;c__Clostridia;o__Oscillospirales;f__Hungateiclostridiaceae;g__Ruminiclostridium;s__Clostridium_sp. | 0 |
| d__Bacteria;p__Proteobacteria;c__Gammaproteobacteria;o__Ectothiorhodospirales;f__Ectothiorhodospiraceae;__;__ | 0 |
| d__Bacteria;p__Firmicutes;c__Clostridia;o__Lachnospirales;f__Lachnospiraceae;g__Coprococcus;__ | 0 |
| d__Bacteria;p__Proteobacteria;c__Alphaproteobacteria;o__Acetobacterales;f__Acetobacteraceae;g__uncultured;s__uncultured_soil | 0 |
| d__Bacteria;p__Actinobacteriota;c__Actinobacteria;o__PeM15;f__PeM15;g__PeM15;__ | 0 |
| d__Bacteria;p__Actinobacteriota;c__Actinobacteria;o__Corynebacteriales;f__Nocardiaceae;g__Nocardia;s__Nocardia_pseudobrasiliensis | 0 |
| d__Bacteria;p__Proteobacteria;c__Alphaproteobacteria;o__Defluviicoccales;f__Defluviicoccaceae;g__Defluviicoccus;s__uncultured_Acetobacteraceae | 0 |
| d__Bacteria;p__Actinobacteriota;c__Actinobacteria;o__Streptomycetales;f__Streptomycetaceae;g__Streptomyces;s__Streptomyces_lincolnensis | 0 |
| d__Bacteria;p__Proteobacteria;c__Alphaproteobacteria;o__Azospirillales;f__Inquilinaceae;g__Inquilinus;s__Inquilinus_sp. | 0 |
| d__Bacteria;p__Firmicutes;c__Clostridia;o__Clostridiales;f__Clostridiaceae;g__Clostridium_sensu_stricto_11;__ | 0 |
| d__Bacteria;p__Proteobacteria;c__Alphaproteobacteria;o__Rhizobiales;f__Hyphomicrobiaceae;g__Hyphomicrobium;s__unidentified | 0 |
| d__Bacteria;p__Proteobacteria;c__Gammaproteobacteria;o__Xanthomonadales;f__Rhodanobacteraceae;g__Rhodanobacter;s__bacterium_enrichment | 0 |
| d__Bacteria;p__Proteobacteria;c__Gammaproteobacteria;o__Enterobacterales;f__Enterobacteriaceae;g__Salmonella;s__Salmonella_enterica | 0 |
| d__Bacteria;p__Bacteroidota;c__Bacteroidia;o__Cytophagales;f__MWH-CFBk5;g__MWH-CFBk5;__ | 0 |
| d__Bacteria;p__Proteobacteria;c__Gammaproteobacteria;o__CHAB-XI-27;f__CHAB-XI-27;g__CHAB-XI-27;__ | 0 |
| d__Bacteria;p__Firmicutes;c__Clostridia;o__Oscillospirales;f__Ruminococcaceae;g__Ruminococcaceae;s__uncultured_bacterium | 0 |
| d__Bacteria;p__Firmicutes;c__Clostridia;o__Lachnospirales;f__Lachnospiraceae;g__Coprococcus;s__uncultured_bacterium | 0 |
| d__Bacteria;p__Firmicutes;c__Clostridia;o__Lachnospirales;f__Lachnospiraceae;g__Lachnospiraceae_FCS020_group;s__uncultured_bacterium | 0 |
| d__Bacteria;p__Proteobacteria;c__Gammaproteobacteria;o__Burkholderiales;f__Burkholderiaceae;g__Burkholderia-Caballeronia-Paraburkholderia;s__Paraburkholderia_diazotrophica | 0 |
| d__Bacteria;p__Proteobacteria;c__Gammaproteobacteria;o__Xanthomonadales;f__Xanthomonadaceae;g__Pseudoxanthomonas;s__Pseudoxanthomonas_koreensis | 0 |
| d__Bacteria;p__Verrucomicrobiota;c__Verrucomicrobiae;o__Opitutales;f__Opitutaceae;g__uncultured;__ | 0 |
| d__Bacteria;p__Proteobacteria;c__Alphaproteobacteria;o__Rhizobiales;f__Rhizobiaceae;g__Allorhizobium-Neorhizobium-Pararhizobium-Rhizobium;s__Rhizobium_tropici | 0 |
| d__Bacteria;p__Firmicutes;c__Clostridia;o__Christensenellales;f__Christensenellaceae;g__Christensenellaceae_R-7_group;s__uncultured_rumen | 0 |
| d__Bacteria;p__Proteobacteria;c__Alphaproteobacteria;o__Rhizobiales;f__Xanthobacteraceae;g__Xanthobacteraceae;s__uncultured_bacterium | 0 |
| d__Bacteria;p__Proteobacteria;c__Gammaproteobacteria;o__Burkholderiales;f__Comamonadaceae;g__Pelomonas;__ | 0 |
| d__Bacteria;p__Verrucomicrobiota;c__Verrucomicrobiae;o__Chthoniobacterales;f__Terrimicrobiaceae;g__FukuN18_freshwater_group;s__uncultured_bacterium | 0 |
| d__Bacteria;p__Firmicutes;c__Clostridia;o__Oscillospirales;f__Oscillospiraceae;g__UCG-002;__ | 0 |
| d__Bacteria;p__Actinobacteriota;c__Acidimicrobiia;o__uncultured;f__uncultured;g__uncultured;s__uncultured_actinobacterium | 0 |
| d__Bacteria;p__Proteobacteria;c__Gammaproteobacteria;o__Burkholderiales;f__Comamonadaceae;g__Polaromonas;s__uncultured_bacterium | 0 |
| d__Bacteria;p__Chloroflexi;c__Ktedonobacteria;o__Ktedonobacterales;f__JG30-KF-AS9;g__JG30-KF-AS9;s__Chloroflexi_bacterium | 0 |
| d__Bacteria;p__Proteobacteria;c__Gammaproteobacteria;o__Salinisphaerales;f__Solimonadaceae;g__Polycyclovorans;s__uncultured_bacterium | 0 |
| d__Bacteria;p__Firmicutes;c__Bacilli;o__Bacillales;f__Marinococcaceae;g__Alteribacillus;__ | 0 |
| d__Bacteria;p__Proteobacteria;c__Gammaproteobacteria;o__Pseudomonadales;f__Pseudomonadaceae;g__Pseudomonas;s__Pseudomonas_mandelii | 0 |
| d__Bacteria;p__Acidobacteriota;c__Acidobacteriae;o__Acidobacteriae;f__Acidobacteriae;g__Paludibaculum;__ | 0 |
| d__Bacteria;p__Actinobacteriota;c__Thermoleophilia;o__Solirubrobacterales;f__Solirubrobacteraceae;g__Conexibacter;s__Conexibacter_sp. | 0 |
| d__Bacteria;p__Firmicutes;c__Sulfobacillia;o__Sulfobacillales;f__Sulfobacillaceae;g__uncultured;s__uncultured_Clostridia | 0 |
| d__Bacteria;p__Firmicutes;c__Bacilli;o__Erysipelotrichales;f__Erysipelotrichaceae;g__Turicibacter;s__uncultured_bacterium | 0 |
| d__Bacteria;p__Proteobacteria;c__Gammaproteobacteria;o__Enterobacterales;f__Aeromonadaceae;g__Aeromonas;s__Aeromonas_hydrophila | 0 |
| d__Bacteria;p__Campylobacterota;c__Campylobacteria;o__Campylobacterales;f__Campylobacteraceae;g__Campylobacter;s__Campylobacter_ureolyticus | 0 |
| d__Bacteria;p__Firmicutes;c__Negativicutes;o__Veillonellales-Selenomonadales;f__Sporomusaceae;g__Pelosinus;s__Pelosinus_fermentans | 0 |
| d__Bacteria;p__Proteobacteria;c__Alphaproteobacteria;o__Caulobacterales;f__Parvularculaceae;g__Amphiplicatus;__ | 0 |
| d__Bacteria;p__Proteobacteria;c__Gammaproteobacteria;o__Enterobacterales;f__Enterobacteriaceae;g__Klebsiella;s__Klebsiella_michiganensis | 0 |
| d__Bacteria;p__Firmicutes;c__Bacilli;o__Bacillales;f__Bacillaceae;g__Aeribacillus;__ | 0 |
| d__Bacteria;p__Firmicutes;c__Clostridia;o__Clostridia;f__Gracilibacteraceae;g__Lutispora;__ | 0 |
| d__Bacteria;p__Planctomycetota;c__Planctomycetes;o__Isosphaerales;f__Isosphaeraceae;g__Aquisphaera;s__uncultured_Planctomycetales | 0 |
| d__Bacteria;p__Verrucomicrobiota;c__Verrucomicrobiae;o__Pedosphaerales;f__Pedosphaeraceae;g__Ellin517;s__uncultured_bacterium | 0 |
| d__Bacteria;p__Firmicutes;c__Clostridia;o__Lachnospirales;f__Lachnospiraceae;g__Agathobacter;__ | 0 |
| d__Bacteria;p__Proteobacteria;c__Alphaproteobacteria;o__Rhizobiales;f__Xanthobacteraceae;g__Pseudolabrys;s__uncultured_Alphaproteobacteria | 0 |
| d__Bacteria;p__Firmicutes;c__Bacilli;o__Bacillales;f__Bacillaceae;g__Bacillus;s__Bacillaceae_bacterium | 0 |
| d__Bacteria;p__Actinobacteriota;c__Acidimicrobiia;o__Microtrichales;f__Ilumatobacteraceae;g__CL500-29_marine_group;s__metagenome | 0 |
| d__Bacteria;p__Firmicutes;c__Limnochordia;o__B55-F-B-G02;f__B55-F-B-G02;g__B55-F-B-G02;s__uncultured_bacterium | 0 |
| d__Bacteria;p__Proteobacteria;c__Alphaproteobacteria;o__Caulobacterales;f__Caulobacteraceae;g__Phenylobacterium;s__Phenylobacterium_sp. | 0 |
| d__Bacteria;p__Latescibacterota;c__Latescibacterota;o__Latescibacterota;f__Latescibacterota;g__Latescibacterota;__ | 0 |
| d__Bacteria;p__Proteobacteria;c__Alphaproteobacteria;o__Rhizobiales;f__Beijerinckiaceae;g__1174-901-12;s__uncultured_bacterium | 0 |
| d__Bacteria;p__Proteobacteria;c__Gammaproteobacteria;o__Pseudomonadales;f__Pseudomonadaceae;g__Pseudomonas;s__Pseudomonas_mendocina | 0 |
| d__Bacteria;p__Firmicutes;c__Negativicutes;o__Veillonellales-Selenomonadales;f__Sporomusaceae;g__Pelosinus;__ | 0 |
| d__Bacteria;p__Proteobacteria;c__Alphaproteobacteria;o__Dongiales;f__Dongiaceae;g__Dongia;s__uncultured_Rhodospirillaceae | 0 |
| d__Bacteria;p__Bacteroidota;c__Bacteroidia;o__Sphingobacteriales;f__Sphingobacteriaceae;g__uncultured;s__uncultured_bacterium | 0 |
| d__Bacteria;p__Proteobacteria;c__Gammaproteobacteria;o__Enterobacterales;f__Enterobacteriaceae;g__Raoultella;__ | 0 |
| d__Bacteria;p__Proteobacteria;c__Gammaproteobacteria;o__Pseudomonadales;f__Moraxellaceae;g__Psychrobacter;__ | 0 |
| d__Bacteria;p__Proteobacteria;c__Gammaproteobacteria;o__Pseudomonadales;f__Pseudomonadaceae;g__Pseudomonas;s__Pseudomonas_fragi | 0 |
| d__Bacteria;p__Firmicutes;c__Bacilli;o__Lactobacillales;f__Lactobacillaceae;g__Leuconostoc;s__Leuconostoc_mesenteroides | 0 |
| d__Bacteria;p__Bacteroidota;c__Bacteroidia;o__Cytophagales;f__Spirosomaceae;g__Runella;__ | 0 |
| d__Bacteria;p__Firmicutes;c__Bacilli;o__Lactobacillales;f__Carnobacteriaceae;g__Granulicatella;__ | 0 |
| d__Bacteria;p__Bacteroidota;c__Bacteroidia;o__Cytophagales;f__Spirosomaceae;g__Rudanella;s__Rudanella_lutea | 0 |
| d__Bacteria;p__Fusobacteriota;c__Fusobacteriia;o__Fusobacteriales;f__Leptotrichiaceae;g__Leptotrichia;__ | 0 |
| d__Bacteria;p__Bacteroidota;c__Bacteroidia;o__Bacteroidales;f__Prevotellaceae;g__Alloprevotella;__ | 0 |
| d__Bacteria;p__Proteobacteria;c__Gammaproteobacteria;o__Enterobacterales;f__Aeromonadaceae;g__Aeromonas;s__Aeromonas_salmonicida | 0 |
| d__Bacteria;p__Firmicutes;c__Clostridia;o__Lachnospirales;f__Lachnospiraceae;g__Blautia;s__Blautia_sp. | 0 |
| d__Bacteria;p__Bacteroidota;c__Bacteroidia;o__Bacteroidales;f__Bacteroidaceae;g__Bacteroides;s__Bacteroides_ovatus | 0 |
| d__Bacteria;p__Proteobacteria;c__Gammaproteobacteria;o__Burkholderiales;f__Alcaligenaceae;g__Alcaligenaceae;s__Achromobacter_xylosoxidans | 0 |
| d__Bacteria;p__Proteobacteria;c__Alphaproteobacteria;o__Ferrovibrionales;f__Ferrovibrionales;g__Taonella;__ | 0 |
| d__Bacteria;p__Firmicutes;c__Clostridia;o__Clostridiales;f__Clostridiaceae;g__Clostridium_sensu_stricto_18;s__Clostridium_sporogenes | 0 |
| d__Bacteria;p__Actinobacteriota;c__Actinobacteria;o__Micromonosporales;f__Micromonosporaceae;g__Micromonospora;s__Micromonospora_sp. | 0 |
| d__Bacteria;p__Proteobacteria;c__Alphaproteobacteria;o__Rhizobiales;f__Hyphomicrobiaceae;g__Hyphomicrobium;s__Hyphomicrobium_denitrificans | 0 |
| d__Bacteria;p__Firmicutes;c__Clostridia;o__Lachnospirales;f__Lachnospiraceae;g__[Eubacterium]_ventriosum_group;s__uncultured_bacterium | 0 |
| d__Bacteria;p__Proteobacteria;c__Gammaproteobacteria;o__Enterobacterales;f__Pasteurellaceae;g__Haemophilus;s__uncultured_bacterium | 0 |
| d__Bacteria;p__Proteobacteria;c__Gammaproteobacteria;o__Pseudomonadales;f__Moraxellaceae;g__Acinetobacter;s__Acinetobacter_schindleri | 0 |
| d__Bacteria;p__Firmicutes;c__Desulfitobacteriia;o__Desulfitobacteriales;f__Syntrophobotulaceae;g__Dehalobacter;s__Dehalobacter_sp. | 0 |
| d__Bacteria;p__Firmicutes;c__Clostridia;o__Eubacteriales;f__Anaerofustaceae;g__Anaerofustis;s__uncultured_bacterium | 0 |
| d__Bacteria;p__Myxococcota;c__Polyangia;o__Nannocystales;f__Nannocystaceae;g__Nannocystis;s__metagenome | 0 |
| d__Bacteria;p__Proteobacteria;c__Alphaproteobacteria;o__Caulobacterales;f__Hyphomonadaceae;g__Hyphomonas;__ | 0 |
| d__Bacteria;p__Proteobacteria;c__Alphaproteobacteria;o__Caulobacterales;f__Caulobacteraceae;g__Caulobacter;s__uncultured_Alphaproteobacteria | 0 |
| d__Bacteria;p__Firmicutes;c__Clostridia;o__Lachnospirales;f__Lachnospiraceae;g__Lachnoclostridium;s__[Clostridium]_methoxybenzovorans | 0 |
| d__Bacteria;p__Planctomycetota;c__Planctomycetes;o__Planctomycetales;f__Rubinisphaeraceae;g__Planctomicrobium;__ | 0 |
| d__Bacteria;p__Proteobacteria;c__Alphaproteobacteria;o__Sneathiellales;f__Sneathiellaceae;g__Sneathiella;s__uncultured_bacterium | 0 |
| d__Bacteria;p__Firmicutes;c__Negativicutes;o__Veillonellales-Selenomonadales;f__Sporomusaceae;g__Acetonema;__ | 0 |
| d__Bacteria;p__Proteobacteria;c__Gammaproteobacteria;o__Methylococcales;f__Methylococcaceae;g__Methylococcus;__ | 0 |
| d__Bacteria;p__Firmicutes;c__Clostridia;o__Clostridiales;f__Clostridiaceae;g__Clostridium_sensu_stricto_1;s__Clostridium_paraputrificum | 0 |
| d__Bacteria;p__Verrucomicrobiota;c__Verrucomicrobiae;o__Chthoniobacterales;f__Chthoniobacteraceae;g__Chthoniobacter;s__metagenome | 0 |
| d__Bacteria;p__Bacteroidota;c__Bacteroidia;o__Bacteroidetes_VC2.1_Bac22;f__Bacteroidetes_VC2.1_Bac22;g__Bacteroidetes_VC2.1_Bac22;__ | 0 |
| d__Bacteria;p__Firmicutes;c__Bacilli;o__Bacillales;f__Planococcaceae;g__Rummeliibacillus;s__Rummeliibacillus_pycnus | 0 |
| d__Bacteria;p__Desulfobacterota;c__Desulfarculia;o__Desulfarculales;f__Desulfarculaceae;__;__ | 0 |
| d__Bacteria;p__Acidobacteriota;c__Subgroup_25;o__Subgroup_25;f__Subgroup_25;g__Subgroup_25;s__uncultured_bacterium | 0 |
| d__Bacteria;p__Proteobacteria;c__Gammaproteobacteria;o__Burkholderiales;f__Nitrosomonadaceae;g__Ellin6067;s__beta_proteobacterium | 0 |
| d__Bacteria;p__Proteobacteria;c__Gammaproteobacteria;o__Pseudomonadales;f__Halieaceae;g__OM60(NOR5)_clade;s__uncultured_bacterium | 0 |
| d__Bacteria;p__Proteobacteria;c__Gammaproteobacteria;o__Burkholderiales;f__Chitinimonadaceae;g__Chitinimonas;__ | 0 |
| d__Bacteria;p__Myxococcota;c__Polyangia;o__Polyangiales;f__Sandaracinaceae;g__Sandaracinus;s__Sandaracinus_amylolyticus | 0 |
| d__Bacteria;p__Proteobacteria;c__Alphaproteobacteria;o__Rhizobiales;f__Rhizobiaceae;g__Allorhizobium-Neorhizobium-Pararhizobium-Rhizobium;s__Rhizobium_daejeonense | 0 |
| d__Bacteria;p__Proteobacteria;c__Gammaproteobacteria;o__Pseudomonadales;f__Moraxellaceae;g__Permianibacter;s__Permianibacter_aggregans | 0 |
| d__Bacteria;p__Proteobacteria;c__Gammaproteobacteria;o__Ectothiorhodospirales;f__Ectothiorhodospiraceae;g__Thiogranum;__ | 0 |
| d__Bacteria;p__Firmicutes;c__Clostridia;o__Oscillospirales;f__Butyricicoccaceae;g__UCG-009;__ | 0 |
| d__Bacteria;p__Verrucomicrobiota;c__Verrucomicrobiae;o__Pedosphaerales;f__Pedosphaeraceae;g__Pedosphaeraceae;s__metagenome | 0 |
| d__Bacteria;p__Firmicutes;c__Limnochordia;o__Limnochordales;f__Limnochordaceae;g__Limnochorda;s__uncultured_compost | 0 |
| d__Bacteria;p__Proteobacteria;c__Alphaproteobacteria;o__Rhizobiales;f__Devosiaceae;g__Devosia;s__uncultured_soil | 0 |
| d__Bacteria;p__Verrucomicrobiota;c__Kiritimatiellae;o__WCHB1-41;f__WCHB1-41;g__WCHB1-41;__ | 0 |
| d__Bacteria;p__Firmicutes;c__Bacilli;o__Bacillales;f__Bacillaceae;g__Bacillus;s__Bacillus_indicus | 0 |
| d__Bacteria;p__Firmicutes;c__Incertae_Sedis;o__DTU014;f__DTU014;g__DTU014;s__anaerobic_digester | 0 |
| d__Bacteria;p__Myxococcota;c__Polyangia;o__Polyangiales;f__Sandaracinaceae;g__uncultured;s__uncultured_soil | 0 |
| d__Bacteria;p__Bacteroidota;c__Bacteroidia;o__Bacteroidales;f__Porphyromonadaceae;g__Porphyromonas;__ | 0 |
| d__Bacteria;p__Firmicutes;c__Bacilli;o__Bacillales;f__Bacillaceae;g__Bacillus;s__Bacillus_soli | 0 |
| d__Bacteria;p__Proteobacteria;c__Alphaproteobacteria;o__Sphingomonadales;f__Sphingomonadaceae;g__Sphingoaurantiacus;s__uncultured_bacterium | 0 |
| d__Bacteria;p__Bacteroidota;c__Bacteroidia;o__Chitinophagales;f__Chitinophagaceae;g__Chitinophaga;s__metagenome | 0 |
| d__Bacteria;p__Proteobacteria;c__Gammaproteobacteria;o__Salinisphaerales;f__Solimonadaceae;g__Nevskia;s__Nevskia_ramosa | 0 |
| d__Bacteria;p__Bdellovibrionota;c__Bdellovibrionia;o__Bdellovibrionales;f__Bdellovibrionaceae;g__Bdellovibrio;s__uncultured_Bdellovibrio | 0 |
| d__Bacteria;p__Proteobacteria;c__Gammaproteobacteria;o__Salinisphaerales;f__Solimonadaceae;g__Nevskia;s__uncultured_bacterium | 0 |
| d__Bacteria;p__Actinobacteriota;c__Actinobacteria;o__Micromonosporales;f__Micromonosporaceae;g__Rugosimonospora;__ | 0 |
| d__Bacteria;p__Verrucomicrobiota;c__Verrucomicrobiae;o__Pedosphaerales;f__Pedosphaeraceae;g__uncultured;s__metagenome | 0 |
| d__Bacteria;p__Proteobacteria;c__Gammaproteobacteria;o__HOC36;f__HOC36;g__HOC36;s__uncultured_bacterium | 0 |
| d__Bacteria;p__Proteobacteria;c__Alphaproteobacteria;o__Acetobacterales;f__Acetobacteraceae;g__uncultured;s__uncultured_Alphaproteobacteria | 0 |
| d__Bacteria;p__Verrucomicrobiota;c__Verrucomicrobiae;o__Methylacidiphilales;f__Methylacidiphilaceae;g__uncultured;s__uncultured_bacterium | 0 |
| d__Bacteria;p__Actinobacteriota;c__Actinobacteria;o__Corynebacteriales;f__Mycobacteriaceae;g__Mycobacterium;s__uncultured_actinobacterium | 0 |
| d__Bacteria;p__Myxococcota;c__Myxococcia;o__Myxococcales;f__Myxococcaceae;g__Myxococcaceae;s__uncultured_bacterium | 0 |
| d__Bacteria;p__Myxococcota;c__Myxococcia;o__Myxococcales;f__Vulgatibacteraceae;g__Vulgatibacter;s__uncultured_delta | 0 |
| d__Bacteria;p__Bdellovibrionota;c__Bdellovibrionia;o__Bdellovibrionales;f__Bdellovibrionaceae;g__Bdellovibrio;s__metagenome | 0 |
| d__Bacteria;p__Proteobacteria;c__Gammaproteobacteria;o__Burkholderiales;f__Hydrogenophilaceae;g__Thiobacillus;s__iron-reducing_bacterium | 0 |
| d__Bacteria;p__Proteobacteria;c__Gammaproteobacteria;o__Burkholderiales;f__Comamonadaceae;g__Leptothrix;s__metagenome | 0 |
| d__Bacteria;p__Firmicutes;c__Bacilli;o__Bacillales;f__Bacillaceae;g__Anaerobacillus;__ | 0 |
| d__Bacteria;p__Actinobacteriota;c__Actinobacteria;o__Micrococcales;f__Microbacteriaceae;g__Gryllotalpicola;s__uncultured_bacterium | 0 |
| d__Bacteria;p__Proteobacteria;c__Gammaproteobacteria;o__Burkholderiales;f__Burkholderiaceae;g__Ralstonia;__ | 0 |
| d__Bacteria;p__Acidobacteriota;c__Acidobacteriae;o__Acidobacteriales;f__Acidobacteriaceae_(Subgroup_1);g__Terracidiphilus;s__uncultured_bacterium | 0 |
| d__Bacteria;p__Proteobacteria;c__Alphaproteobacteria;o__Rhizobiales;f__Hyphomicrobiaceae;g__Hyphomicrobium;s__Hyphomicrobium_methylovorum | 0 |
| d__Bacteria;p__Proteobacteria;c__Alphaproteobacteria;o__Rhizobiales;f__Rhizobiaceae;g__Mesorhizobium;s__Mesorhizobium_australicum | 0 |
| d__Bacteria;p__Firmicutes;c__Clostridia;o__Peptostreptococcales-Tissierellales;f__Peptostreptococcaceae;g__Paeniclostridium;__ | 0 |
| d__Bacteria;p__Cyanobacteria;c__Cyanobacteriia;o__Leptolyngbyales;f__Leptolyngbyaceae;g__Leptolyngbya_ANT.L52.2;__ | 0 |
| d__Bacteria;p__Proteobacteria;c__Gammaproteobacteria;o__Burkholderiales;f__Comamonadaceae;g__Alicycliphilus;__ | 0 |
| d__Bacteria;p__Proteobacteria;c__Alphaproteobacteria;o__Rhizobiales;f__Beijerinckiaceae;g__Methylocystis;s__uncultured_bacterium | 0 |
| d__Bacteria;p__Proteobacteria;c__Gammaproteobacteria;o__Burkholderiales;f__Oxalobacteraceae;g__Duganella;__ | 0 |
| d__Bacteria;p__Proteobacteria;c__Gammaproteobacteria;o__WD260;f__WD260;g__WD260;s__uncultured_bacterium | 0 |
| d__Bacteria;p__Proteobacteria;c__Gammaproteobacteria;o__Pseudomonadales;f__Halomonadaceae;g__Halomonas;s__uncultured_bacterium | 0 |
| d__Bacteria;p__Proteobacteria;c__Alphaproteobacteria;o__Rickettsiales;f__Mitochondria;g__Mitochondria;s__uncultured_bacterium | 0 |
| d__Bacteria;p__Firmicutes;c__Clostridia;o__Lachnospirales;f__Lachnospiraceae;g__Tyzzerella;__ | 0 |
| d__Bacteria;p__Armatimonadota;c__Fimbriimonadia;o__Fimbriimonadales;f__Fimbriimonadaceae;g__Fimbriimonadaceae;__ | 0 |
| d__Bacteria;p__Bacteroidota;c__Bacteroidia;o__Chitinophagales;f__Chitinophagaceae;g__Heliimonas;__ | 0 |
| d__Bacteria;p__Proteobacteria;c__Gammaproteobacteria;o__Burkholderiales;f__Comamonadaceae;g__Ottowia;s__uncultured_bacterium | 0 |
| d__Bacteria;p__Proteobacteria;c__Gammaproteobacteria;o__Burkholderiales;f__Neisseriaceae;g__uncultured;__ | 0 |
| d__Bacteria;p__Verrucomicrobiota;c__Verrucomicrobiae;o__Opitutales;f__Opitutaceae;g__IMCC26134;s__metagenome | 0 |
| d__Bacteria;p__Proteobacteria;c__Alphaproteobacteria;o__Paracaedibacterales;f__Paracaedibacteraceae;g__uncultured;__ | 0 |
| d__Bacteria;p__Acidobacteriota;c__Acidobacteriae;o__Acidobacteriales;f__Acidobacteriaceae_(Subgroup_1);g__Acidicapsa;__ | 0 |
| d__Bacteria;p__Proteobacteria;c__Gammaproteobacteria;o__JG36-GS-52;f__JG36-GS-52;g__JG36-GS-52;__ | 0 |
| d__Bacteria;p__Spirochaetota;c__Leptospirae;o__Leptospirales;f__Leptospiraceae;g__Leptospira;__ | 0 |
| d__Bacteria;p__Verrucomicrobiota;c__Verrucomicrobiae;o__Pedosphaerales;f__Pedosphaeraceae;g__DEV008;s__metagenome | 0 |
| d__Bacteria;p__Acidobacteriota;c__Acidobacteriae;o__Subgroup_13;f__Subgroup_13;g__Subgroup_13;s__uncultured_bacterium | 0 |
| d__Bacteria;p__Cyanobacteria;c__Cyanobacteriia;o__Leptolyngbyales;f__Leptolyngbyaceae;g__Phormidium_SAG_37.90;__ | 0 |
| d__Bacteria;p__Proteobacteria;c__Alphaproteobacteria;o__Micavibrionales;f__Micavibrionaceae;g__uncultured;s__uncultured_Alphaproteobacteria | 0 |
| d__Bacteria;p__Proteobacteria;c__Alphaproteobacteria;o__Kiloniellales;__;__;__ | 0 |
| d__Bacteria;p__Proteobacteria;c__Alphaproteobacteria;o__Paracaedibacterales;f__Paracaedibacteraceae;__;__ | 0 |
| d__Bacteria;p__Bacteroidota;c__Bacteroidia;o__Cytophagales;f__Cytophagaceae;g__Cytophaga;s__uncultured_bacterium | 0 |
| d__Bacteria;p__Proteobacteria;c__Gammaproteobacteria;o__Burkholderiales;f__Methylophilaceae;g__Methylobacillus;s__uncultured_bacterium | 0 |
| d__Bacteria;p__Verrucomicrobiota;c__Omnitrophia;o__Omnitrophales;__;__;__ | 0 |
| d__Bacteria;p__Proteobacteria;c__Gammaproteobacteria;o__Burkholderiales;f__Comamonadaceae;g__Variovorax;s__Variovorax_paradoxus | 0 |
| d__Bacteria;p__Verrucomicrobiota;c__Lentisphaeria;o__Victivallales;f__Arctic95B-14;g__Arctic95B-14;s__uncultured_Lentisphaerae | 0 |
| d__Bacteria;p__Planctomycetota;c__Planctomycetes;o__Isosphaerales;f__Isosphaeraceae;g__Tundrisphaera;s__Tundrisphaera_lichenicola | 0 |
| d__Bacteria;p__Proteobacteria;c__Gammaproteobacteria;o__Diplorickettsiales;f__Diplorickettsiaceae;g__uncultured;s__uncultured_gamma | 0 |
| d__Bacteria;p__Verrucomicrobiota;c__Verrucomicrobiae;o__uncultured;f__uncultured;g__uncultured;__ | 0 |
| d__Bacteria;p__Actinobacteriota;c__Actinobacteria;o__Streptomycetales;f__Streptomycetaceae;g__Streptomyces;s__Streptomyces_cattleya | 0 |
| d__Bacteria;p__Firmicutes;c__Desulfitobacteriia;o__Desulfitobacteriales;f__Desulfitobacteriaceae;g__Desulfosporosinus;s__Desulfosporosinus_sp. | 0 |
| d__Bacteria;p__Proteobacteria;c__Gammaproteobacteria;o__Burkholderiales;f__A21b;g__A21b;s__uncultured_bacterium | 0 |
| d__Bacteria;p__Planctomycetota;c__Planctomycetes;o__Pirellulales;f__Pirellulaceae;g__uncultured;s__metagenome | 0 |
| d__Bacteria;p__Chloroflexi;c__Ktedonobacteria;o__Ktedonobacterales;f__Ktedonobacteraceae;g__uncultured;s__uncultured_Clostridiales | 0 |
| d__Bacteria;p__Firmicutes;c__Bacilli;o__Bacillales;f__Bacillaceae;g__Bacillus;s__Bacillus_fumarioli | 0 |
| d__Bacteria;p__Proteobacteria;c__Alphaproteobacteria;o__Paracaedibacterales;f__Paracaedibacteraceae;g__Candidatus_Paracaedibacter;s__uncultured_bacterium | 0 |
| d__Bacteria;p__Bacteroidota;c__Bacteroidia;o__Chitinophagales;f__Chitinophagaceae;g__Puia;s__uncultured_Chitinophagaceae | 0 |
| d__Bacteria;p__Proteobacteria;c__Gammaproteobacteria;o__Pseudomonadales;f__Hahellaceae;g__Hahella;__ | 0 |
| d__Bacteria;p__Proteobacteria;c__Gammaproteobacteria;o__Burkholderiales;f__Comamonadaceae;g__Diaphorobacter;s__uncultured_bacterium | 0 |
| d__Bacteria;p__Acidobacteriota;c__Acidobacteriae;o__Subgroup_2;f__Subgroup_2;g__Subgroup_2;__ | 0 |
| d__Bacteria;p__Proteobacteria;c__Gammaproteobacteria;o__Burkholderiales;f__Burkholderiaceae;g__Burkholderia-Caballeronia-Paraburkholderia;s__Trinickia_soli | 0 |
| d__Bacteria;p__Bacteroidota;c__Bacteroidia;o__Chitinophagales;f__Chitinophagaceae;g__Filimonas;__ | 0 |
| d__Bacteria;p__Bdellovibrionota;c__Oligoflexia;o__Oligoflexales;f__Oligoflexaceae;g__Oligoflexus;s__uncultured_bacterium | 0 |
| d__Bacteria;p__Proteobacteria;c__Gammaproteobacteria;o__Burkholderiales;f__Oxalobacteraceae;g__Duganella;s__Duganella_sp. | 0 |
| d__Bacteria;p__Acidobacteriota;c__Holophagae;o__Holophagales;f__Holophagaceae;g__Holophaga;__ | 0 |
| d__Bacteria;p__Proteobacteria;c__Gammaproteobacteria;o__Xanthomonadales;f__Xanthomonadaceae;g__Xanthomonas;s__Xanthomonas_oryzae | 0 |
| d__Bacteria;p__Proteobacteria;c__Gammaproteobacteria;o__Xanthomonadales;f__Rhodanobacteraceae;g__Chiayiivirga;__ | 0 |
| d__Bacteria;p__Proteobacteria;c__Gammaproteobacteria;o__Burkholderiales;f__Rhodocyclaceae;g__Azospira;__ | 0 |
| d__Bacteria;p__Proteobacteria;c__Alphaproteobacteria;o__Acetobacterales;f__Acetobacteraceae;g__Rhodovastum;s__metagenome | 0 |
| d__Bacteria;p__Acidobacteriota;c__Acidobacteriae;o__Acidobacteriales;f__Acidobacteriaceae_(Subgroup_1);g__Bryocella;s__uncultured_bacterium | 0 |
| d__Bacteria;p__Proteobacteria;c__Gammaproteobacteria;o__Xanthomonadales;f__Xanthomonadaceae;g__Luteimonas;s__Lysobacter_pocheonensis | 0 |
| d__Bacteria;p__Firmicutes;c__Bacilli;o__Bacillales;f__Planococcaceae;g__Rummeliibacillus;s__uncultured_compost | 0 |
| d__Bacteria;p__Cyanobacteria;c__Cyanobacteriia;o__Cyanobacteriales;f__Phormidiaceae;g__Tychonema_CCAP_1459-11B;__ | 0 |
| d__Bacteria;p__Actinobacteriota;c__Actinobacteria;o__Micrococcales;f__Microbacteriaceae;g__Curtobacterium;s__Curtobacterium_sp. | 0 |
| d__Bacteria;p__Actinobacteriota;c__Actinobacteria;o__Micrococcales;f__Demequinaceae;g__Demequina;s__uncultured_bacterium | 0 |
| d__Bacteria;p__Verrucomicrobiota;c__Lentisphaeria;o__SS1-B-02-17;f__SS1-B-02-17;g__SS1-B-02-17;s__uncultured_organism | 0 |
| d__Bacteria;p__Bacteroidota;c__Bacteroidia;o__Sphingobacteriales;f__KD3-93;g__KD3-93;s__uncultured_bacterium | 0 |
| d__Bacteria;p__Armatimonadota;c__Fimbriimonadia;o__Fimbriimonadales;f__Fimbriimonadaceae;g__Fimbriimonadaceae;s__metagenome | 0 |
| d__Bacteria;p__Firmicutes;c__Clostridia;o__Lachnospirales;f__Lachnospiraceae;g__Sellimonas;__ | 0 |
| d__Bacteria;p__Proteobacteria;c__Alphaproteobacteria;o__Rhodobacterales;f__Rhodobacteraceae;g__Falsirhodobacter;s__uncultured_bacterium | 0 |
| d__Bacteria;p__Firmicutes;c__Limnochordia;o__Limnochordales;f__Limnochordaceae;g__Limnochordaceae;s__uncultured_Clostridia | 0 |
| d__Bacteria;p__Proteobacteria;c__Alphaproteobacteria;o__Micavibrionales;__;__;__ | 0 |
| d__Bacteria;p__Spirochaetota;c__Spirochaetia;o__Spirochaetales;f__Spirochaetaceae;g__Spirochaeta_2;s__uncultured_organism | 0 |
| d__Bacteria;p__Firmicutes;c__Clostridia;o__Clostridia;f__Gracilibacteraceae;g__Gracilibacter;s__Gracilibacter_thermotolerans | 0 |
| d__Bacteria;p__Proteobacteria;c__Alphaproteobacteria;o__Micavibrionales;f__Micavibrionaceae;__;__ | 0 |
| d__Bacteria;p__Proteobacteria;c__Gammaproteobacteria;o__Piscirickettsiales;f__Piscirickettsiaceae;g__Candidatus_Endoecteinascidia;s__metagenome | 0 |
| d__Bacteria;p__Proteobacteria;c__Alphaproteobacteria;o__Ferrovibrionales;__;__;__ | 0 |
| d__Bacteria;p__Verrucomicrobiota;c__Verrucomicrobiae;o__Opitutales;f__Puniceicoccaceae;g__Cerasicoccus;s__Ruficoccus_amylovorans | 0 |
| d__Bacteria;p__Firmicutes;c__Clostridia;o__Oscillospirales;f__Oscillospiraceae;g__uncultured;s__uncultured_prokaryote | 0 |
| d__Bacteria;p__Planctomycetota;c__Planctomycetes;o__Isosphaerales;f__Isosphaeraceae;g__uncultured;s__planctomycete_WY108 | 0 |
| d__Bacteria;p__Proteobacteria;c__Gammaproteobacteria;o__Burkholderiales;f__Comamonadaceae;g__Pseudacidovorax;s__Acidovorax_sp. | 0 |
| d__Bacteria;p__Firmicutes;c__Clostridia;o__Lachnospirales;f__Lachnospiraceae;g__Herbinix;s__Herbinix_luporum | 0 |
| d__Bacteria;p__Proteobacteria;c__Gammaproteobacteria;o__Burkholderiales;f__Comamonadaceae;g__uncultured;s__Acidovorax_sp. | 0 |
| d__Bacteria;p__Proteobacteria;c__Gammaproteobacteria;o__Burkholderiales;f__Comamonadaceae;g__Pseudorhodoferax;__ | 0 |
| d__Bacteria;p__Acidobacteriota;c__Blastocatellia;o__Blastocatellales;f__Blastocatellaceae;g__OLB17;__ | 0 |
| d__Bacteria;p__Proteobacteria;c__Gammaproteobacteria;o__Burkholderiales;f__Burkholderiaceae;g__Cupriavidus;s__Cupriavidus_pinatubonensis | 0 |
| d__Bacteria;p__Verrucomicrobiota;c__Verrucomicrobiae;o__Pedosphaerales;f__Pedosphaeraceae;g__Pedosphaera;__ | 0 |
| d__Bacteria;p__Proteobacteria;c__Gammaproteobacteria;o__Xanthomonadales;f__Rhodanobacteraceae;g__Dokdonella;s__uncultured_gamma | 0 |
| d__Bacteria;p__Cyanobacteria;c__Cyanobacteriia;o__Cyanobacteriales;f__Phormidiaceae;g__Cephalothrix_SAG_75.79;s__uncultured_bacterium | 0 |
| d__Bacteria;p__Spirochaetota;c__V2072-189E03;o__V2072-189E03;f__V2072-189E03;g__V2072-189E03;__ | 0 |
| d__Bacteria;p__Verrucomicrobiota;c__Verrucomicrobiae;o__Verrucomicrobiales;f__DEV007;g__DEV007;s__uncultured_bacterium | 0 |
| d__Bacteria;p__Proteobacteria;c__Alphaproteobacteria;o__Rhodospirillales;f__Rhodospirillaceae;__;__ | 0 |
| d__Bacteria;p__Bacteroidota;c__Bacteroidia;o__Bacteroidales;f__Prolixibacteraceae;g__WCHB1-32;s__uncultured_bacterium | 0 |
| d__Bacteria;p__Proteobacteria;c__Gammaproteobacteria;o__Burkholderiales;f__Comamonadaceae;g__Ideonella;s__uncultured_bacterium | 0 |
| d__Bacteria;p__Chloroflexi;c__Chloroflexia;o__Chloroflexales;f__Roseiflexaceae;g__uncultured;s__uncultured_Chloroflexi | 0 |
| d__Bacteria;p__Proteobacteria;c__Gammaproteobacteria;o__Burkholderiales;f__Gallionellaceae;g__Sideroxydans;s__uncultured_bacterium | 0 |
| d__Bacteria;p__Spirochaetota;c__Spirochaetia;o__Spirochaetales;f__Spirochaetaceae;g__M2PT2-76_termite_group;__ | 0 |
| d__Bacteria;p__Actinobacteriota;c__Thermoleophilia;o__Solirubrobacterales;f__Solirubrobacteraceae;g__Conexibacter;s__metagenome | 0 |
| d__Bacteria;p__Planctomycetota;c__Planctomycetes;o__Planctomycetales;f__Rubinisphaeraceae;g__uncultured;s__uncultured_bacterium | 0 |
| d__Bacteria;p__Fibrobacterota;c__Fibrobacteria;o__Fibrobacterales;f__uncultured;g__uncultured;s__uncultured_Fibrobacteres | 0 |
| d__Bacteria;p__Proteobacteria;c__Gammaproteobacteria;o__Ectothiorhodospirales;f__Thioalkalispiraceae;g__uncultured;s__metagenome | 0 |
| d__Bacteria;p__Verrucomicrobiota;c__Lentisphaeria;o__Victivallales;f__Arctic95B-14;g__Arctic95B-14;s__uncultured_deep-sea | 0 |
| d__Bacteria;p__Proteobacteria;c__Gammaproteobacteria;o__Burkholderiales;f__TRA3-20;g__TRA3-20;s__uncultured_beta | 0 |
| d__Bacteria;p__Firmicutes;c__Clostridia;o__Oscillospirales;f__Oscillospiraceae;g__uncultured;s__uncultured_Firmicutes | 0 |
| d__Bacteria;p__Proteobacteria;c__Alphaproteobacteria;o__Rhodospirillales;f__Rhodospirillaceae;g__uncultured;s__uncultured_bacterium | 0 |
| d__Bacteria;p__Fibrobacterota;c__Fibrobacteria;o__Fibrobacterales;f__B122;g__B122;s__uncultured_bacterium | 0 |
| d__Bacteria;p__Proteobacteria;c__Gammaproteobacteria;o__Burkholderiales;f__Comamonadaceae;g__Acidovorax;s__Acidovorax_sp. | 0 |
| d__Bacteria;p__Bacteroidota;c__Bacteroidia;o__SM1A07;f__SM1A07;g__SM1A07;s__uncultured_organism | 0 |
| d__Bacteria;p__Firmicutes;c__Clostridia;o__Peptostreptococcales-Tissierellales;f__Family_XI;g__Family_XI;s__uncultured_Sporanaerobacter | 0 |
| d__Bacteria;p__Desulfobacterota;c__Desulfuromonadia;o__Geobacterales;f__Geobacteraceae;g__Geotalea;__ | 0 |
| d__Bacteria;p__Proteobacteria;c__Alphaproteobacteria;o__Acetobacterales;f__Acetobacteraceae;g__Craurococcus-Caldovatus;s__Caldovatus_sediminis | 0 |
| d__Bacteria;p__Proteobacteria;c__Gammaproteobacteria;o__Arenicellales;f__Arenicellaceae;g__uncultured;s__uncultured_bacterium | 0 |
| d__Bacteria;p__Verrucomicrobiota;c__Verrucomicrobiae;o__Verrucomicrobiales;f__DEV007;g__DEV007;__ | 0 |
| d__Bacteria;p__Firmicutes;c__Bacilli;o__Paenibacillales;f__Paenibacillaceae;g__Cohnella;s__Cohnella_hongkongensis | 0 |
| d__Bacteria;p__Bacteroidota;c__Bacteroidia;o__Sphingobacteriales;f__LiUU-11-161;g__LiUU-11-161;__ | 0 |
| d__Bacteria;p__Desulfobacterota;c__Desulfobacteria;o__Desulfobacterales;f__Desulfosarcinaceae;g__SEEP-SRB1;__ | 0 |
| d__Bacteria;p__Myxococcota;c__Polyangia;o__Polyangiales;f__Polyangiaceae;g__Aetherobacter;__ | 0 |
| d__Bacteria;p__Spirochaetota;c__V2072-189E03;o__V2072-189E03;f__V2072-189E03;g__V2072-189E03;s__uncultured_Spirochaetales | 0 |
| d__Bacteria;p__Bacteroidota;c__Bacteroidia;o__SM1A07;f__SM1A07;g__SM1A07;s__metagenome | 0 |
| d__Bacteria;p__Proteobacteria;c__Gammaproteobacteria;o__Burkholderiales;f__A21b;g__A21b;__ | 0 |
| d__Bacteria;p__Firmicutes;c__Clostridia;o__Oscillospirales;f__Hungateiclostridiaceae;g__HN-HF0106;s__uncultured_prokaryote | 0 |
| d__Bacteria;p__Bacteroidota;c__Bacteroidia;o__Chitinophagales;__;__;__ | 0 |
| d__Bacteria;p__Acidobacteriota;c__Subgroup_11;o__Subgroup_11;f__Subgroup_11;g__Subgroup_11;__ | 0 |
| d__Bacteria;p__Proteobacteria;c__Gammaproteobacteria;o__Burkholderiales;f__Alcaligenaceae;g__Derxia;__ | 0 |
| d__Bacteria;p__Planctomycetota;c__vadinHA49;o__vadinHA49;f__vadinHA49;g__vadinHA49;__ | 0 |
| d__Bacteria;p__Bacteroidota;c__Kapabacteria;o__Kapabacteriales;f__Kapabacteriales;g__Kapabacteriales;__ | 0 |
| d__Bacteria;p__Acidobacteriota;c__Subgroup_22;o__Subgroup_22;f__Subgroup_22;g__Subgroup_22;__ | 0 |
| d__Bacteria;p__Planctomycetota;c__Phycisphaerae;o__MSBL9;f__SG8-4;g__SG8-4;s__uncultured_organism | 0 |
| d__Bacteria;p__Firmicutes;c__Clostridia;o__Oscillospirales;f__Ruminococcaceae;g__Negativibacillus;s__uncultured_bacterium | 0 |
| d__Bacteria;p__Firmicutes;c__TSAC18;o__TSAC18;f__TSAC18;g__TSAC18;s__uncultured_bacterium | 0 |
| d__Bacteria;p__Spirochaetota;c__Spirochaetia;o__Spirochaetales;f__Spirochaetaceae;g__Treponema;s__uncultured_bacterium | 0 |
| d__Bacteria;p__Verrucomicrobiota;c__Verrucomicrobiae;o__Pedosphaerales;f__Pedosphaeraceae;g__DEV008;s__uncultured_bacterium | 0 |
| d__Bacteria;p__Proteobacteria;c__Gammaproteobacteria;o__Burkholderiales;f__Oxalobacteraceae;g__Undibacterium;s__uncultured_bacterium | 0 |
| d__Bacteria;p__Proteobacteria;c__Alphaproteobacteria;o__Caulobacterales;f__Hyphomonadaceae;g__uncultured;s__uncultured_bacterium | 0 |
| d__Bacteria;p__Acidobacteriota;c__Blastocatellia;o__Pyrinomonadales;f__Pyrinomonadaceae;g__Pyrinomonas;__ | 0 |
| d__Bacteria;p__Proteobacteria;c__Gammaproteobacteria;o__Burkholderiales;f__Rhodocyclaceae;g__Propionivibrio;s__uncultured_bacterium | 0 |
| d__Bacteria;p__Chloroflexi;c__Gitt-GS-136;o__Gitt-GS-136;f__Gitt-GS-136;g__Gitt-GS-136;__ | 0 |
| d__Bacteria;p__Bacteroidota;c__Bacteroidia;o__Bacteroidales;f__SB-5;g__SB-5;s__uncultured_Bacteroidetes | 0 |
| d__Bacteria;p__Proteobacteria;c__Gammaproteobacteria;o__Steroidobacterales;f__Steroidobacteraceae;g__uncultured;s__Steroidobacter_sp. | 0 |
| d__Bacteria;p__Proteobacteria;c__Gammaproteobacteria;o__EV818SWSAP88;f__EV818SWSAP88;g__EV818SWSAP88;__ | 0 |
| d__Bacteria;p__Planctomycetota;c__Phycisphaerae;o__Tepidisphaerales;f__Tepidisphaeraceae;g__Tepidisphaera;s__uncultured_bacterium | 0 |
| d__Bacteria;p__Proteobacteria;c__Gammaproteobacteria;o__Burkholderiales;f__Rhodocyclaceae;g__Azovibrio;s__uncultured_bacterium | 0 |
| d__Bacteria;p__Planctomycetota;c__Planctomycetes;o__Pirellulales;f__Pirellulaceae;g__uncultured;s__uncultured_Planctomycetales | 0 |
| d__Bacteria;p__Patescibacteria;c__Gracilibacteria;o__Candidatus_Abawacabacteria;f__Candidatus_Abawacabacteria;g__Candidatus_Abawacabacteria;s__uncultured_bacterium | 0 |
| d__Bacteria;p__Desulfobacterota;c__Desulfuromonadia;o__Geobacterales;f__Geobacteraceae;g__uncultured;__ | 0 |
| d__Bacteria;p__Patescibacteria;c__Parcubacteria;o__Candidatus_Moranbacteria;f__Candidatus_Moranbacteria;g__Candidatus_Moranbacteria;s__uncultured_bacterium | 0 |
| d__Bacteria;p__Patescibacteria;c__Microgenomatia;o__Candidatus_Woesebacteria;f__Candidatus_Woesebacteria;g__Candidatus_Woesebacteria;s__uncultured_Microgenomates | 0 |
| d__Bacteria;p__Spirochaetota;c__Spirochaetia;o__Spirochaetales;f__Spirochaetaceae;g__Treponema;s__Treponema_stenostreptum | 0 |
| d__Bacteria;p__Planctomycetota;c__Planctomycetes;o__Planctomycetales;f__Rubinisphaeraceae;__;__ | 0 |
| d__Bacteria;p__Firmicutes;c__Bacilli;o__Bacillales;f__Bacillaceae;g__Bacillus;s__Bacillus_acidiceler | 0 |
| d__Bacteria;p__Chloroflexi;c__Dehalococcoidia;o__Dehalococcoidales;__;__;__ | 0 |
| d__Bacteria;p__Acidobacteriota;c__Acidobacteriae;o__Acidobacteriae;f__Acidobacteriae;g__Paludibaculum;s__uncultured_Acidobacteria | 0 |
| d__Bacteria;p__Spirochaetota;c__Leptospirae;o__Leptospirales;f__Leptospiraceae;g__RBG-16-49-21;s__uncultured_bacterium | 0 |
| d__Bacteria;p__Bacteroidota;c__Bacteroidia;o__Bacteroidales;f__Paludibacteraceae;g__uncultured;s__uncultured_soil | 0 |
| d__Bacteria;p__Verrucomicrobiota;c__Verrucomicrobiae;o__Opitutales;f__Opitutaceae;g__Opitutus;s__unidentified_eubacterium | 0 |
| d__Bacteria;p__Chloroflexi;c__Anaerolineae;o__Ardenticatenales;f__uncultured;g__uncultured;s__uncultured_Chloroflexi | 0 |
| d__Bacteria;p__Proteobacteria;c__Alphaproteobacteria;o__Micavibrionales;f__Micavibrionaceae;g__uncultured;s__uncultured_organism | 0 |
| d__Bacteria;p__Proteobacteria;c__Alphaproteobacteria;o__Rickettsiales;f__SM1B06;g__SM1B06;s__uncultured_bacterium | 0 |
| d__Bacteria;p__Patescibacteria;c__Microgenomatia;o__Candidatus_Shapirobacteria;f__Candidatus_Shapirobacteria;g__Candidatus_Shapirobacteria;s__uncultured_bacterium | 0 |
| d__Bacteria;p__Planctomycetota;c__Phycisphaerae;o__S-70;f__S-70;g__S-70;__ | 0 |
| d__Bacteria;p__PAUC34f;c__PAUC34f;o__PAUC34f;f__PAUC34f;g__PAUC34f;s__uncultured_bacterium | 0 |
| d__Bacteria;p__Proteobacteria;c__Gammaproteobacteria;o__Burkholderiales;f__Nitrosomonadaceae;g__IS-44;s__metagenome | 0 |
| d__Bacteria;p__Planctomycetota;c__BD7-11;o__BD7-11;f__BD7-11;g__BD7-11;s__uncultured_planctomycete | 0 |
| d__Bacteria;p__Planctomycetota;c__Phycisphaerae;o__mle1-8;f__mle1-8;g__mle1-8;s__uncultured_organism | 0 |
| d__Bacteria;p__Firmicutes;c__Desulfotomaculia;o__Desulfotomaculales;f__Desulfotomaculaceae;g__Desulfotomaculum;s__uncultured_bacterium | 0 |
| d__Bacteria;p__Bacteroidota;c__Kryptonia;o__Kryptoniales;f__MSB-3C8;g__MSB-3C8;s__uncultured_bacterium | 0 |
| d__Bacteria;p__Actinobacteriota;c__Acidimicrobiia;o__Microtrichales;f__Microtrichaceae;g__uncultured;s__metagenome | 0 |
| d__Bacteria;p__Fibrobacterota;c__Fibrobacteria;o__Fibrobacterales;f__09D2Z48;g__09D2Z48;s__uncultured_organism | 0 |
| d__Bacteria;p__Firmicutes;c__Bacilli;o__Bacillales;f__Bacillaceae;g__Geobacillus;s__Parageobacillus_thermoglucosidasius | 0 |
| d__Bacteria;p__Cyanobacteria;c__Vampirivibrionia;o__Vampirovibrionales;__;__;__ | 0 |
| d__Bacteria;p__Patescibacteria;c__CPR2;o__CPR2;f__CPR2;g__CPR2;s__uncultured_Clostridium | 0 |
| d__Bacteria;p__Planctomycetota;c__Planctomycetes;o__Pirellulales;f__Pirellulaceae;g__Candidatus_Anammoximicrobium;__ | 0 |
| d__Bacteria;p__Firmicutes;c__Bacilli;o__Paenibacillales;f__Paenibacillaceae;g__Paenibacillus;s__Paenibacillus_macerans | 0 |
| d__Bacteria;p__Spirochaetota;c__V2072-189E03;o__V2072-189E03;f__V2072-189E03;g__V2072-189E03;s__uncultured_bacterium | 0 |
| d__Bacteria;p__Planctomycetota;c__Phycisphaerae;o__Pla1_lineage;f__Pla1_lineage;g__Pla1_lineage;__ | 0 |
| d__Bacteria;p__Patescibacteria;c__ABY1;o__Candidatus_Kerfeldbacteria;f__Candidatus_Kerfeldbacteria;g__Candidatus_Kerfeldbacteria;s__uncultured_bacterium | 0 |
| d__Bacteria;p__Cyanobacteria;c__Cyanobacteriia;o__Chloroplast;f__Chloroplast;g__Chloroplast;s__Gossypium_arboreum | 0 |
| d__Bacteria;p__Proteobacteria;c__Alphaproteobacteria;o__Rickettsiales;f__Mitochondria;g__Mitochondria;s__Histiona_aroides | 0 |
| d__Bacteria;p__Gemmatimonadota;c__MD2902-B12;o__MD2902-B12;f__MD2902-B12;g__MD2902-B12;s__uncultured_bacterium | 0 |
| d__Bacteria;p__Cyanobacteria;c__Vampirivibrionia;o__Vampirovibrionales;f__Vampirovibrionaceae;g__Vampirovibrio;s__metagenome | 0 |
| d__Bacteria;p__Firmicutes;c__Clostridia;o__Clostridiales;f__Clostridiaceae;g__Proteiniclasticum;__ | 0 |
| d__Bacteria;p__Proteobacteria;c__Gammaproteobacteria;o__Burkholderiales;f__Comamonadaceae;g__Hylemonella;s__Hylemonella_gracilis | 0 |
| d__Bacteria;p__Desulfobacterota;c__Syntrophobacteria;o__Syntrophobacterales;f__Syntrophobacteraceae;g__uncultured;__ | 0 |
| d__Bacteria;p__Proteobacteria;c__Alphaproteobacteria;o__Rhizobiales;f__Beijerinckiaceae;g__alphaI_cluster;s__uncultured_bacterium | 0 |
| d__Bacteria;p__Proteobacteria;c__Alphaproteobacteria;o__Acetobacterales;__;__;__ | 0 |
| d__Bacteria;p__Bacteroidota;c__OC31;o__OC31;f__OC31;g__OC31;s__uncultured_bacterium | 0 |
| d__Bacteria;p__Proteobacteria;c__Gammaproteobacteria;o__Pseudomonadales;f__Porticoccaceae;g__Porticoccus;s__metagenome | 0 |
| d__Bacteria;p__Myxococcota;c__Polyangia;o__Polyangiales;f__BIrii41;g__BIrii41;s__uncultured_organism | 0 |
| d__Bacteria;p__Patescibacteria;c__CPR2;o__CPR2;f__CPR2;g__CPR2;__ | 0 |
| d__Bacteria;p__Proteobacteria;c__Gammaproteobacteria;o__Burkholderiales;f__Comamonadaceae;g__Hydrogenophaga;s__Hydrogenophaga_palleronii | 0 |
| d__Bacteria;p__Actinobacteriota;c__Acidimicrobiia;o__Microtrichales;f__uncultured;g__uncultured;s__uncultured_Acidimicrobiia | 0 |
| d__Bacteria;p__Proteobacteria;c__Gammaproteobacteria;o__Burkholderiales;f__Comamonadaceae;g__Comamonas;s__Comamonas_terrigena | 0 |
| d__Bacteria;p__Firmicutes;c__Bacilli;o__Bacillales;f__Sporolactobacillaceae;g__Sporolactobacillus;s__Sporolactobacillus_inulinus | 0 |
| d__Bacteria;p__Patescibacteria;c__ABY1;o__Candidatus_Komeilibacteria;f__Candidatus_Komeilibacteria;g__Candidatus_Komeilibacteria;s__uncultured_bacterium | 0 |
| d__Bacteria;p__Proteobacteria;c__Gammaproteobacteria;o__Pseudomonadales;f__Pseudohongiellaceae;g__BIyi10;s__uncultured_bacterium | 0 |
| d__Bacteria;p__Proteobacteria;c__Gammaproteobacteria;o__Burkholderiales;f__Comamonadaceae;g__Piscinibacter;s__Piscinibacter_aquaticus | 0 |
| d__Bacteria;p__Proteobacteria;c__Alphaproteobacteria;o__Rhizobiales;f__Rhizobiaceae;g__Allorhizobium-Neorhizobium-Pararhizobium-Rhizobium;s__Rhizobium_altiplani | 0 |
| d__Bacteria;p__Proteobacteria;c__Gammaproteobacteria;o__Burkholderiales;f__Rhodocyclaceae;g__Zoogloea;s__uncultured_beta | 0 |
| d__Bacteria;p__Armatimonadota;c__Armatimonadia;o__Armatimonadales;__;__;__ | 0 |
| d__Bacteria;p__Proteobacteria;c__Gammaproteobacteria;o__Pseudomonadales;f__Moraxellaceae;g__Fluviicoccus;__ | 0 |
| d__Bacteria;p__Proteobacteria;c__Gammaproteobacteria;o__Pseudomonadales;f__Pseudomonadaceae;g__Pseudomonas;s__Pseudomonas_alcaligenes | 0 |
| d__Bacteria;p__Proteobacteria;c__Alphaproteobacteria;o__Rhodobacterales;f__Rhodobacteraceae;g__Pseudorhodobacter;s__uncultured_bacterium | 0 |
| d__Bacteria;p__Proteobacteria;c__Gammaproteobacteria;o__uncultured;f__uncultured;g__uncultured;s__uncultured_organism | 0 |
| d__Bacteria;p__Proteobacteria;c__Alphaproteobacteria;o__Azospirillales;f__Azospirillaceae;g__Niveispirillum;__ | 0 |
| d__Bacteria;p__Verrucomicrobiota;c__Verrucomicrobiae;o__Opitutales;f__Puniceicoccaceae;g__Cerasicoccus;__ | 0 |
| d__Bacteria;p__Cyanobacteria;c__Cyanobacteriia;o__Chloroplast;f__Chloroplast;g__Chloroplast;s__metagenome | 0 |
| d__Bacteria;p__Proteobacteria;c__Alphaproteobacteria;o__Caulobacterales;f__Hyphomonadaceae;g__SWB02;s__metagenome | 0 |
| d__Bacteria;p__Actinobacteriota;c__Actinobacteria;o__Streptomycetales;f__Streptomycetaceae;g__Streptomyces;s__Streptomyces_nodosus | 0 |
| d__Bacteria;p__Proteobacteria;c__Gammaproteobacteria;o__Burkholderiales;f__Oxalobacteraceae;g__Herbaspirillum;s__Herbaspirillum_huttiense | 0 |
| d__Bacteria;p__Proteobacteria;c__Gammaproteobacteria;o__Burkholderiales;f__Burkholderiaceae;g__Ralstonia;s__Ralstonia_solanacearum | 0 |
| d__Bacteria;p__Bacteroidota;c__Bacteroidia;o__Cytophagales;f__Cyclobacteriaceae;g__Marinoscillum;__ | 0 |
| d__Bacteria;p__Bacteroidota;c__Bacteroidia;o__Cytophagales;f__Microscillaceae;g__Chryseolinea;s__metagenome | 0 |
| d__Bacteria;p__Bacteroidota;c__Bacteroidia;o__Chitinophagales;f__Chitinophagaceae;g__Flavisolibacter;s__uncultured_Bacteroidetes | 0 |
| d__Bacteria;p__Proteobacteria;c__Gammaproteobacteria;o__Legionellales;f__Legionellaceae;g__uncultured;s__uncultured_gamma | 0 |
| d__Bacteria;p__Firmicutes;c__Bacilli;o__Bacillales;f__Bacillaceae;g__Bacillus;s__Bacillus_pseudofirmus | 0 |
| d__Bacteria;p__Cyanobacteria;c__Cyanobacteriia;o__Chloroplast;f__Chloroplast;g__Chloroplast;s__Punica_granatum | 0 |
| d__Bacteria;p__Proteobacteria;c__Gammaproteobacteria;o__Burkholderiales;f__Burkholderiaceae;g__Cupriavidus;s__Cupriavidus_numazuensis | 0 |
| d__Bacteria;p__Proteobacteria;c__Alphaproteobacteria;o__Rickettsiales;f__AB1;g__AB1;s__uncultured_proteobacterium | 0 |
| d__Bacteria;p__Proteobacteria;c__Gammaproteobacteria;o__Burkholderiales;f__Burkholderiaceae;g__Cupriavidus;s__Cupriavidus_metallidurans | 0 |
| d__Bacteria;p__Bacteroidota;c__Bacteroidia;o__Flavobacteriales;f__Flavobacteriaceae;g__Flavobacterium;s__Flavobacterium_johnsoniae | 0 |
| d__Bacteria;p__Proteobacteria;c__Gammaproteobacteria;o__Legionellales;f__Legionellaceae;g__uncultured;__ | 0 |
| d__Bacteria;p__Proteobacteria;c__Gammaproteobacteria;o__Burkholderiales;f__Comamonadaceae;g__Ottowia;s__Ottowia_pentelensis | 0 |
| d__Bacteria;p__Patescibacteria;c__Gracilibacteria;o__Candidatus_Peribacteria;f__Candidatus_Peribacteria;g__Candidatus_Peribacteria;s__metagenome | 0 |
| d__Bacteria;p__Bacteroidota;c__Bacteroidia;o__Chitinophagales;f__Chitinophagaceae;g__Niastella;s__Niastella_koreensis | 0 |
| d__Bacteria;p__Proteobacteria;c__Alphaproteobacteria;o__Rhizobiales;f__Rhizobiaceae;g__Mesorhizobium;s__Mesorhizobium_loti | 0 |
| d__Bacteria;p__Verrucomicrobiota;c__Omnitrophia;o__Omnitrophales;f__Omnitrophales;g__Omnitrophales;s__uncultured_bacterium | 0 |
| d__Bacteria;p__Firmicutes;c__Sulfobacillia;o__Sulfobacillales;f__Sulfobacillaceae;g__Sulfobacillus;s__uncultured_Firmicutes | 0 |
| d__Bacteria;p__Cyanobacteria;c__Cyanobacteriia;o__Chloroplast;f__Chloroplast;g__Chloroplast;s__Alisma_plantago-aquatica | 0 |
| d__Bacteria;p__Proteobacteria;c__Gammaproteobacteria;o__Burkholderiales;f__Comamonadaceae;g__Aquincola;s__Aquincola_tertiaricarbonis | 0 |
| d__Bacteria;p__Proteobacteria;c__Gammaproteobacteria;o__Xanthomonadales;f__Rhodanobacteraceae;g__Rhodanobacter;s__uncultured_Xanthomonadaceae | 0 |
| d__Bacteria;p__Bacteroidota;c__Kapabacteria;o__Kapabacteriales;f__Kapabacteriales;g__Kapabacteriales;s__metagenome | 0 |
| d__Bacteria;p__Proteobacteria;c__Gammaproteobacteria;o__Burkholderiales;f__Comamonadaceae;g__Comamonadaceae;s__uncultured_bacterium | 0 |
| d__Bacteria;p__Proteobacteria;c__Alphaproteobacteria;o__Paracaedibacterales;f__Paracaedibacteraceae;g__Candidatus_Captivus;__ | 0 |
| d__Bacteria;p__Proteobacteria;c__Gammaproteobacteria;o__Burkholderiales;f__Chromobacteriaceae;g__Pseudogulbenkiania;__ | 0 |
| d__Bacteria;p__Bacteroidota;c__Bacteroidia;o__Flavobacteriales;f__Weeksellaceae;g__Chryseobacterium;s__Chryseobacterium_sp. | 0 |
| d__Bacteria;p__Cyanobacteria;c__Cyanobacteriia;o__Leptolyngbyales;f__Leptolyngbyaceae;g__Leptolyngbya_PCC-6306;__ | 0 |
| d__Bacteria;p__Bdellovibrionota;c__Bdellovibrionia;o__Bacteriovoracales;f__Bacteriovoracaceae;g__Bacteriovorax;__ | 0 |
| d__Bacteria;p__Patescibacteria;c__ABY1;o__Candidatus_Magasanikbacteria;f__Candidatus_Magasanikbacteria;g__Candidatus_Magasanikbacteria;__ | 0 |
| d__Bacteria;p__Proteobacteria;c__Gammaproteobacteria;o__Burkholderiales;f__Methylophilaceae;g__Methylophilus;s__uncultured_Methylophilus | 0 |
| d__Bacteria;p__Cyanobacteria;c__Vampirivibrionia;o__Caenarcaniphilales;f__Caenarcaniphilales;g__Caenarcaniphilales;s__uncultured_cyanobacterium | 0 |
| d__Bacteria;p__Bacteroidota;c__Bacteroidia;o__Flavobacteriales;f__Weeksellaceae;g__Moheibacter;s__uncultured_bacterium | 0 |
| d__Bacteria;p__Actinobacteriota;c__Acidimicrobiia;o__Microtrichales;f__Microtrichaceae;__;__ | 0 |
| d__Bacteria;p__Cyanobacteria;c__Cyanobacteriia;o__Cyanobacteriales;f__Phormidiaceae;g__Phormidium_IAM_M-71;__ | 0 |
| d__Bacteria;p__Patescibacteria;c__Microgenomatia;o__Microgenomatia;f__Microgenomatia;g__Microgenomatia;__ | 0 |
| d__Bacteria;p__Actinobacteriota;c__Actinobacteria;o__Micrococcales;f__Microbacteriaceae;g__Parafrigoribacterium;__ | 0 |
| d__Bacteria;p__Proteobacteria;c__Gammaproteobacteria;o__Burkholderiales;f__Alcaligenaceae;g__Alcaligenaceae;s__Achromobacter_spanius | 0 |
| d__Bacteria;p__Proteobacteria;c__Alphaproteobacteria;o__Rhodobacterales;f__Rhodobacteraceae;g__Pseudorhodobacter;__ | 0 |
| d__Bacteria;p__Sumerlaeota;c__Sumerlaeia;o__Sumerlaeales;f__Sumerlaeaceae;g__Sumerlaea;s__metagenome | 0 |
| d__Bacteria;p__Proteobacteria;c__Gammaproteobacteria;o__Xanthomonadales;f__Xanthomonadaceae;g__Luteimonas;s__Lysobacter_lycopersici | 0 |
| d__Bacteria;p__Proteobacteria;c__Gammaproteobacteria;o__Pseudomonadales;f__Cellvibrionaceae;g__Cellvibrio;s__uncultured_bacterium | 0 |
| d__Bacteria;p__Cyanobacteria;c__Cyanobacteriia;o__Leptolyngbyales;f__Leptolyngbyaceae;g__Leptolyngbya_VRUC_135;__ | 0 |
| d__Bacteria;p__Cyanobacteria;c__Cyanobacteriia;__;__;__;__ | 0 |
| d__Bacteria;p__Proteobacteria;c__Gammaproteobacteria;o__Xanthomonadales;f__Xanthomonadaceae;g__Luteimonas;s__Lysobacter_sp. | 0 |
| d__Bacteria;p__Proteobacteria;c__Gammaproteobacteria;o__Pseudomonadales;f__Pseudomonadaceae;g__Pseudomonas;s__Pseudomonas_protegens | 0 |
| d__Bacteria;p__Proteobacteria;c__Gammaproteobacteria;o__Xanthomonadales;f__Xanthomonadaceae;g__Thermomonas;s__Thermomonas_sp. | 0 |
| d__Bacteria;p__Proteobacteria;c__Gammaproteobacteria;o__Enterobacterales;f__Enterobacteriaceae;g__Enterobacter;s__Enterobacter_asburiae | 0 |
| d__Bacteria;p__Patescibacteria;c__Gracilibacteria;o__Gracilibacteria;f__Gracilibacteria;g__Gracilibacteria;__ | 0 |
| d__Bacteria;p__Proteobacteria;c__Alphaproteobacteria;o__Rhizobiales;f__Beijerinckiaceae;g__Methylobacterium-Methylorubrum;s__Methylobacterium_currus | 0 |
| d__Bacteria;p__Proteobacteria;c__Alphaproteobacteria;o__Caulobacterales;f__Caulobacteraceae;g__Brevundimonas;s__Brevundimonas_viscosa | 0 |
| d__Bacteria;p__Proteobacteria;c__Gammaproteobacteria;o__Burkholderiales;f__Comamonadaceae;g__Variovorax;s__Variovorax_sp. | 0 |
| d__Bacteria;p__TX1A-33;c__TX1A-33;o__TX1A-33;f__TX1A-33;g__TX1A-33;s__uncultured_bacterium | 0 |
| d__Bacteria;p__Proteobacteria;c__Alphaproteobacteria;o__Thalassobaculales;__;__;__ | 0 |
| d__Bacteria;p__Proteobacteria;c__Gammaproteobacteria;o__Gammaproteobacteria_Incertae_Sedis;f__Unknown_Family;g__Acidibacter;s__uncultured_soil | 0 |
| d__Bacteria;p__Proteobacteria;c__Gammaproteobacteria;o__Xanthomonadales;f__Xanthomonadaceae;g__Luteimonas;s__uncultured_gamma | 0 |
| d__Bacteria;p__Proteobacteria;c__Gammaproteobacteria;o__Burkholderiales;f__Comamonadaceae;g__Alicycliphilus;s__uncultured_bacterium | 0 |
| d__Bacteria;p__Verrucomicrobiota;c__Lentisphaeria;o__Victivallales;f__Arctic95B-14;g__Arctic95B-14;__ | 0 |
| d__Bacteria;p__Verrucomicrobiota;c__Verrucomicrobiae;o__Opitutales;f__Opitutaceae;g__Opitutus;s__Verrucomicrobia_bacterium | 0 |
| d__Bacteria;p__Proteobacteria;c__Gammaproteobacteria;o__Burkholderiales;f__Comamonadaceae;g__Xylophilus;__ | 0 |
| d__Bacteria;p__Proteobacteria;c__Alphaproteobacteria;o__Rhizobiales;f__Rhizobiaceae;g__Shinella;s__Shinella_fusca | 0 |
| d__Bacteria;p__Acidobacteriota;c__Acidobacteriae;o__Acidobacteriales;f__Acidobacteriaceae_(Subgroup_1);g__Terracidiphilus;s__uncultured_Acidobacteriaceae | 0 |
| d__Bacteria;p__Actinobacteriota;c__Actinobacteria;o__Corynebacteriales;f__Nocardiaceae;g__Gordonia;s__Gordonia_polyisoprenivorans | 0 |
| d__Bacteria;p__Proteobacteria;c__Gammaproteobacteria;o__Burkholderiales;f__Comamonadaceae;g__Rhodoferax;s__uncultured_bacterium | 0 |
| d__Bacteria;p__Proteobacteria;c__Alphaproteobacteria;o__Tistrellales;f__Tistrellaceae;g__Tistrella;s__Rhodobium_sp. | 0 |
| d__Bacteria;p__Actinobacteriota;c__Actinobacteria;o__Micrococcales;f__Intrasporangiaceae;g__Pedococcus-Phycicoccus;s__Phycicoccus_jejuensis | 0 |
| d__Bacteria;p__Proteobacteria;c__Alphaproteobacteria;o__Rhizobiales;f__Beijerinckiaceae;g__Methylobacterium-Methylorubrum;s__Methylobacterium_sp. | 0 |
| d__Bacteria;p__Proteobacteria;c__Gammaproteobacteria;o__Burkholderiales;f__Comamonadaceae;g__Hydrogenophaga;s__uncultured_beta | 0 |
| d__Bacteria;p__Proteobacteria;c__Alphaproteobacteria;o__Rhizobiales;f__Rhizobiaceae;g__Allorhizobium-Neorhizobium-Pararhizobium-Rhizobium;s__Rhizobium_rhizoryzae | 0 |
| d__Bacteria;p__Proteobacteria;c__Alphaproteobacteria;o__Sphingomonadales;f__Sphingomonadaceae;g__Novosphingobium;s__uncultured_soil | 0 |
| d__Bacteria;p__Proteobacteria;c__Alphaproteobacteria;o__Defluviicoccales;f__uncultured;g__uncultured;s__metagenome | 0 |
| d__Bacteria;p__Proteobacteria;c__Gammaproteobacteria;o__Burkholderiales;f__Comamonadaceae;g__Rhizobacter;s__metagenome | 0 |
| d__Bacteria;p__Proteobacteria;c__Alphaproteobacteria;o__Rhizobiales;f__Rhizobiaceae;g__Ensifer;s__Sinorhizobium_sp. | 0 |
| d__Bacteria;p__Proteobacteria;c__Gammaproteobacteria;o__Xanthomonadales;f__Rhodanobacteraceae;g__Dokdonella;s__Dokdonella_immobilis | 0 |
| d__Bacteria;p__Actinobacteriota;c__Actinobacteria;o__Frankiales;f__Geodermatophilaceae;g__Antricoccus;__ | 0 |
| d__Bacteria;p__Abditibacteriota;c__Abditibacteria;o__Abditibacteriales;f__Abditibacteriaceae;g__Abditibacterium;__ | 0 |
| d__Bacteria;p__Proteobacteria;c__Gammaproteobacteria;o__Burkholderiales;f__Methylophilaceae;g__uncultured;__ | 0 |
| d__Bacteria;p__Firmicutes;c__Clostridia;o__Lachnospirales;f__Lachnospiraceae;g__Oribacterium;s__uncultured_bacterium | 0 |
| d__Bacteria;p__Proteobacteria;c__Gammaproteobacteria;o__CCM19a;f__CCM19a;g__CCM19a;s__uncultured_bacterium | 0 |
| d__Bacteria;p__Proteobacteria;c__Alphaproteobacteria;o__Dongiales;f__Dongiaceae;g__Dongia;s__uncultured_Alphaproteobacteria | 0 |
| d__Bacteria;p__Bdellovibrionota;c__Oligoflexia;o__053A03-B-DI-P58;f__053A03-B-DI-P58;g__053A03-B-DI-P58;__ | 0 |
| d__Bacteria;p__Proteobacteria;c__Gammaproteobacteria;o__Burkholderiales;f__Comamonadaceae;g__Pelomonas;s__Pelomonas_puraquae | 0 |
| d__Bacteria;p__Proteobacteria;c__Alphaproteobacteria;o__Paracaedibacterales;f__Paracaedibacteraceae;g__Candidatus_Finniella;s__uncultured_bacterium | 0 |
| d__Bacteria;p__Planctomycetota;c__Phycisphaerae;o__Phycisphaerales;f__Phycisphaeraceae;g__Phycisphaera;s__uncultured_bacterium | 0 |
| d__Bacteria;p__Bdellovibrionota;c__Oligoflexia;o__Silvanigrellales;f__Silvanigrellaceae;g__Silvanigrella;s__uncultured_bacterium | 0 |
| d__Bacteria;p__Proteobacteria;c__Gammaproteobacteria;o__Pseudomonadales;f__Pseudomonadaceae;g__Pseudomonas;s__Pseudomonas_mosselii | 0 |
| d__Bacteria;p__Proteobacteria;c__Alphaproteobacteria;o__Holosporales;f__Holosporaceae;g__uncultured;s__uncultured_Alphaproteobacteria | 0 |
| d__Bacteria;p__Proteobacteria;c__Gammaproteobacteria;o__Pseudomonadales;f__Halieaceae;g__OM60(NOR5)_clade;__ | 0 |
| d__Bacteria;p__Chloroflexi;c__Ktedonobacteria;o__Ktedonobacterales;f__Ktedonobacteraceae;g__uncultured;s__uncultured_Ktedonobacterales | 0 |
| d__Bacteria;p__Acidobacteriota;c__Subgroup_11;o__Subgroup_11;f__Subgroup_11;g__Subgroup_11;s__uncultured_bacterium | 0 |
| d__Bacteria;p__Bdellovibrionota;c__Oligoflexia;o__Silvanigrellales;f__Silvanigrellaceae;g__Silvanigrella;s__Spirobacillus_sp. | 0 |
| d__Bacteria;p__Cyanobacteria;c__Vampirivibrionia;o__Obscuribacterales;f__Obscuribacteraceae;g__Obscuribacteraceae;__ | 0 |
| d__Bacteria;p__Proteobacteria;c__Gammaproteobacteria;o__Burkholderiales;f__Alcaligenaceae;g__Bordetella;s__uncultured_bacterium | 0 |
| d__Bacteria;p__Firmicutes;c__Bacilli;o__Bacillales;f__Bacillaceae;g__Bacillus;s__Bacillus_infernus | 0 |
| d__Bacteria;p__Proteobacteria;c__Gammaproteobacteria;o__Burkholderiales;f__Methylophilaceae;g__Methylobacillus;s__Methylobacillus_flagellatus | 0 |
| d__Bacteria;p__Planctomycetota;c__Phycisphaerae;o__Phycisphaerales;f__Phycisphaeraceae;g__uncultured;s__metagenome | 0 |
| d__Bacteria;p__Proteobacteria;c__Alphaproteobacteria;o__Acetobacterales;f__Acetobacteraceae;g__uncultured;s__uncultured_eubacterium | 0 |
| d__Bacteria;p__Proteobacteria;c__Gammaproteobacteria;o__Burkholderiales;f__Comamonadaceae;g__Thiomonas;s__uncultured_bacterium | 0 |
| d__Bacteria;p__Proteobacteria;c__Gammaproteobacteria;o__Burkholderiales;f__Comamonadaceae;g__Variovorax;s__metagenome | 0 |
| d__Bacteria;p__Proteobacteria;c__Gammaproteobacteria;o__Burkholderiales;f__Burkholderiaceae;g__Burkholderia-Caballeronia-Paraburkholderia;s__Paraburkholderia_tropica | 0 |
| d__Bacteria;p__Proteobacteria;c__Gammaproteobacteria;o__Burkholderiales;f__Chromobacteriaceae;g__Pseudogulbenkiania;s__Pseudogulbenkiania_sp. | 0 |
| d__Bacteria;p__Cyanobacteria;c__Cyanobacteriia;o__Oxyphotobacteria_Incertae_Sedis;f__Unknown_Family;__;__ | 0 |
| d__Bacteria;p__Firmicutes;c__Negativicutes;o__Veillonellales-Selenomonadales;f__Sporomusaceae;g__Pelosinus;s__Pelosinus_sp. | 0 |
| d__Bacteria;p__Verrucomicrobiota;c__Lentisphaeria;o__Oligosphaerales;__;__;__ | 0 |
| d__Bacteria;p__Proteobacteria;c__Gammaproteobacteria;o__Pseudomonadales;f__Alcanivoracaceae1;g__Alcanivorax;s__uncultured_bacterium | 0 |
| d__Bacteria;p__Bacteroidota;c__Bacteroidia;o__Sphingobacteriales;f__env.OPS_17;g__env.OPS_17;s__uncultured_Bacteroidetes | 0 |
| d__Bacteria;p__Proteobacteria;c__Alphaproteobacteria;o__Rhizobiales;f__Xanthobacteraceae;g__Bradyrhizobium;s__uncultured_Bradyrhizobium | 0 |
| d__Bacteria;p__Planctomycetota;c__Phycisphaerae;o__Phycisphaerales;f__Phycisphaeraceae;g__Phycisphaera;__ | 0 |
| d__Bacteria;p__Actinobacteriota;c__Actinobacteria;o__Micromonosporales;f__Micromonosporaceae;g__Dactylosporangium;__ | 0 |
| d__Bacteria;p__Proteobacteria;c__Gammaproteobacteria;o__Burkholderiales;f__SC-I-84;g__SC-I-84;s__uncultured_beta | 0 |
| d__Bacteria;p__Bacteroidota;c__Bacteroidia;o__Sphingobacteriales;f__Sphingobacteriaceae;g__Mucilaginibacter;s__Sphingobacteriaceae_bacterium | 0 |
| d__Bacteria;p__Proteobacteria;c__Gammaproteobacteria;o__Xanthomonadales;f__Xanthomonadaceae;g__Lysobacter;s__Lysobacter_sediminicola | 0 |
| d__Bacteria;p__Proteobacteria;c__Alphaproteobacteria;o__Micropepsales;f__Micropepsaceae;g__uncultured;s__bacterium_Ellin335 | 0 |
| d__Bacteria;p__Proteobacteria;c__Alphaproteobacteria;o__Rickettsiales;f__Midichloriaceae;g__Candidatus_Midichloria;__ | 0 |
| d__Bacteria;p__Proteobacteria;c__Gammaproteobacteria;o__Nitrococcales;f__Nitrococcaceae;g__Nitrococcus;__ | 0 |
| d__Bacteria;p__Bacteroidota;c__Bacteroidia;o__Sphingobacteriales;f__Sphingobacteriaceae;g__Solitalea;__ | 0 |
| d__Bacteria;p__Proteobacteria;c__Alphaproteobacteria;o__Paracaedibacterales;f__Paracaedibacteraceae;g__Candidatus_Captivus;s__uncultured_bacterium | 0 |
| d__Bacteria;p__Proteobacteria;c__Gammaproteobacteria;o__Burkholderiales;f__Rhodocyclaceae;g__Uliginosibacterium;s__Uliginosibacterium_sangjuense | 0 |
| d__Bacteria;p__WPS-2;c__WPS-2;o__WPS-2;f__WPS-2;g__WPS-2;s__bacterium_enrichment | 0 |
| d__Bacteria;p__Actinobacteriota;c__Actinobacteria;o__Frankiales;f__Sporichthyaceae;g__uncultured;__ | 0 |
| d__Bacteria;p__Myxococcota;c__Polyangia;o__VHS-B3-70;f__VHS-B3-70;g__VHS-B3-70;s__uncultured_bacterium | 0 |
| d__Bacteria;p__Actinobacteriota;c__Actinobacteria;o__Corynebacteriales;f__Nocardiaceae;g__Williamsia;__ | 0 |
| d__Bacteria;p__Proteobacteria;c__Gammaproteobacteria;o__Burkholderiales;f__Burkholderiaceae;g__Cupriavidus;s__Cupriavidus_basilensis | 0 |
| d__Bacteria;p__Firmicutes;c__Clostridia;o__Lachnospirales;f__Lachnospiraceae;g__[Eubacterium]_fissicatena_group;__ | 0 |
| d__Bacteria;p__Firmicutes;c__Bacilli;o__Alicyclobacillales;f__Alicyclobacillaceae;g__Alicyclobacillus;s__Alicyclobacillus_cycloheptanicus | 0 |
| d__Bacteria;p__Proteobacteria;c__Gammaproteobacteria;o__Burkholderiales;f__Comamonadaceae;g__Simplicispira;s__uncultured_bacterium | 0 |
| d__Bacteria;p__Proteobacteria;c__Gammaproteobacteria;o__Xanthomonadales;f__Xanthomonadaceae;g__Xanthomonas;s__Xanthomonas_maliensis | 0 |
| d__Bacteria;p__Verrucomicrobiota;c__Verrucomicrobiae;o__Pedosphaerales;f__Pedosphaeraceae;g__Ellin517;s__metagenome | 0 |
| d__Bacteria;p__Gemmatimonadota;c__Gemmatimonadetes;o__Gemmatimonadales;f__Gemmatimonadaceae;g__uncultured;s__metagenome | 0 |
| d__Bacteria;p__Firmicutes;c__Bacilli;o__Bacillales;f__Bacillaceae;g__Bacillus;s__Bacillus_filamentosus | 0 |
| d__Bacteria;p__Firmicutes;c__Clostridia;o__Clostridia_UCG-014;f__Clostridia_UCG-014;g__Clostridia_UCG-014;__ | 0 |
| d__Bacteria;p__SAR324_clade(Marine_group_B);c__SAR324_clade(Marine_group_B);o__SAR324_clade(Marine_group_B);f__SAR324_clade(Marine_group_B);g__SAR324_clade(Marine_group_B);s__uncultured_delta | 0 |
| d__Bacteria;p__Firmicutes;c__Desulfitobacteriia;o__Desulfitobacteriales;f__uncultured;g__uncultured;__ | 0 |
| d__Bacteria;p__Cyanobacteria;c__Cyanobacteriia;o__Chloroplast;f__Chloroplast;g__Chloroplast;s__Gossypium_barbadense | 0 |
| d__Bacteria;p__Bacteroidota;c__Kapabacteria;o__Kapabacteriales;f__Kapabacteriales;g__Kapabacteriales;s__uncultured_soil | 0 |
| d__Bacteria;p__Actinobacteriota;c__Actinobacteria;o__Micromonosporales;f__Micromonosporaceae;g__Hamadaea;__ | 0 |
| d__Bacteria;p__Verrucomicrobiota;c__Verrucomicrobiae;o__Opitutales;f__Puniceicoccaceae;g__Verruc-01;s__uncultured_Puniceicoccaceae | 0 |
| d__Bacteria;p__Myxococcota;c__Polyangia;o__Polyangiales;f__Sandaracinaceae;__;__ | 0 |
| d__Bacteria;p__Cyanobacteria;c__Cyanobacteriia;o__Chloroplast;f__Chloroplast;g__Chloroplast;s__uncultured_Streptophyta | 0 |
| d__Bacteria;p__Chloroflexi;c__Ktedonobacteria;o__Ktedonobacterales;f__Ktedonobacteraceae;g__Ktedonobacter;s__Ktedonobacter_racemifer | 0 |
| d__Bacteria;p__Bacteroidota;c__Bacteroidia;o__Cytophagales;f__Microscillaceae;g__uncultured;s__metagenome | 0 |
| d__Bacteria;p__Planctomycetota;c__Planctomycetes;o__Gemmatales;f__Gemmataceae;g__Zavarzinella;__ | 0 |
| d__Bacteria;p__Cyanobacteria;c__Cyanobacteriia;o__Chloroplast;f__Chloroplast;g__Chloroplast;s__Swertia_japonica | 0 |
| d__Bacteria;p__Proteobacteria;c__Gammaproteobacteria;o__Xanthomonadales;f__Xanthomonadaceae;g__Lysobacter;s__uncultured_Lysobacter | 0 |
| d__Bacteria;p__Verrucomicrobiota;c__Verrucomicrobiae;o__Verrucomicrobiales;f__Verrucomicrobiaceae;g__Roseimicrobium;s__metagenome | 0 |
| d__Bacteria;p__Verrucomicrobiota;c__Verrucomicrobiae;o__S-BQ2-57_soil_group;f__S-BQ2-57_soil_group;g__S-BQ2-57_soil_group;s__uncultured_bacterium | 0 |
| d__Bacteria;p__Firmicutes;c__Bacilli;o__Paenibacillales;f__Paenibacillaceae;g__Paenibacillus;s__Paenibacillus_lautus | 0 |
| d__Bacteria;p__Firmicutes;c__Bacilli;o__Bacillales;f__Bacillaceae;g__Bacillus;s__Bacillus_sporothermodurans | 0 |
| d__Bacteria;p__Verrucomicrobiota;c__Verrucomicrobiae;o__Verrucomicrobiales;f__Verrucomicrobiaceae;__;__ | 0 |
| d__Bacteria;p__Proteobacteria;c__Alphaproteobacteria;o__Micropepsales;f__Micropepsaceae;g__Rhizomicrobium;s__uncultured_bacterium | 0 |
| d__Bacteria;p__Gemmatimonadota;c__Gemmatimonadetes;o__Gemmatimonadales;f__Gemmatimonadaceae;g__Roseisolibacter;__ | 0 |
| d__Bacteria;p__Proteobacteria;c__Alphaproteobacteria;o__uncultured;f__uncultured;g__uncultured;s__Lacibacterium_aquatile | 0 |
| d__Bacteria;p__Bacteroidota;c__Bacteroidia;o__Chitinophagales;f__Chitinophagaceae;g__Niastella;s__metagenome | 0 |
| d__Bacteria;p__Verrucomicrobiota;c__Verrucomicrobiae;o__Opitutales;f__Puniceicoccaceae;g__Cerasicoccus;s__uncultured_bacterium | 0 |
| d__Bacteria;p__Bacteroidota;c__Ignavibacteria;o__SJA-28;f__SJA-28;g__SJA-28;s__metagenome | 0 |
| d__Bacteria;p__Dependentiae;c__Babeliae;o__Babeliales;f__Vermiphilaceae;g__Vermiphilaceae;s__uncultured_Candidatus | 0 |
| d__Bacteria;p__Elusimicrobiota;c__Lineage_IIb;o__Lineage_IIb;f__Lineage_IIb;g__Lineage_IIb;__ | 0 |
| d__Bacteria;p__Bacteroidota;c__Bacteroidia;o__Chitinophagales;f__Chitinophagaceae;g__UTBCD1;__ | 0 |
| d__Bacteria;p__Cyanobacteria;c__Cyanobacteriia;o__Chloroplast;f__Chloroplast;g__Chloroplast;s__Persicaria_minor | 0 |
| d__Bacteria;p__Proteobacteria;c__Alphaproteobacteria;o__Rhizobiales;f__Devosiaceae;g__Devosia;s__Devosia_sp. | 0 |
| d__Bacteria;p__Firmicutes;c__Bacilli;o__Lactobacillales;f__Streptococcaceae;g__Streptococcus;s__Streptococcus_oralis | 0 |
| d__Bacteria;p__Firmicutes;c__Bacilli;o__Lactobacillales;f__Carnobacteriaceae;g__Granulicatella;s__uncultured_bacterium | 0 |
| d__Bacteria;p__Bacteroidota;c__Bacteroidia;o__Flavobacteriales;f__Flavobacteriaceae;g__Flavobacterium;s__metagenome | 0 |
| d__Bacteria;p__Verrucomicrobiota;c__Verrucomicrobiae;o__Opitutales;f__Opitutaceae;g__Cephaloticoccus;s__uncultured_bacterium | 0 |
| d__Bacteria;p__Firmicutes;c__Limnochordia;o__Limnochordia;f__Limnochordia;g__Hydrogenispora;s__uncultured_prokaryote | 0 |
| d__Bacteria;p__Actinobacteriota;c__Actinobacteria;o__Streptosporangiales;f__Thermomonosporaceae;g__Actinomadura;s__Actinomadura_nitritigenes | 0 |
| d__Bacteria;p__Planctomycetota;c__Planctomycetes;o__Planctomycetales;f__Rubinisphaeraceae;g__SH-PL14;s__Planctomyces_sp. | 0 |
| d__Bacteria;p__Proteobacteria;c__Gammaproteobacteria;o__Legionellales;f__Legionellaceae;g__uncultured;s__uncultured_bacterium | 0 |
| d__Bacteria;p__Verrucomicrobiota;c__Verrucomicrobiae;o__Pedosphaerales;f__Pedosphaeraceae;g__Ellin517;__ | 0 |
| d__Bacteria;p__Proteobacteria;c__Gammaproteobacteria;o__Enterobacterales;f__Alteromonadaceae;g__Rheinheimera;s__uncultured_bacterium | 0 |
| d__Bacteria;p__Bacteroidota;c__Bacteroidia;o__Chitinophagales;f__Chitinophagaceae;g__uncultured;s__uncultured_Bacteroidetes | 0 |
| d__Bacteria;p__Proteobacteria;c__Gammaproteobacteria;o__Burkholderiales;f__Oxalobacteraceae;g__Duganella;s__Massilia_sp. | 0 |
| d__Bacteria;p__Patescibacteria;c__Gracilibacteria;o__JGI_0000069-P22;f__JGI_0000069-P22;g__JGI_0000069-P22;s__uncultured_bacterium | 0 |
| d__Bacteria;p__Proteobacteria;c__Gammaproteobacteria;o__Xanthomonadales;f__Xanthomonadaceae;g__Lysobacter;s__Lysobacter_ximonensis | 0 |
| d__Bacteria;p__Proteobacteria;c__Alphaproteobacteria;o__NRL2;f__NRL2;g__NRL2;__ | 0 |
| d__Bacteria;p__Spirochaetota;c__Spirochaetia;o__Spirochaetales;f__Spirochaetaceae;g__Salinispira;__ | 0 |
| d__Bacteria;p__Proteobacteria;c__Gammaproteobacteria;o__Xanthomonadales;f__Rhodanobacteraceae;g__Oleiagrimonas;s__uncultured_bacterium | 0 |
| d__Bacteria;p__Bacteroidota;c__Bacteroidia;o__Chitinophagales;f__Chitinophagaceae;g__Niabella;s__uncultured_bacterium | 0 |
| d__Bacteria;p__Firmicutes;c__Desulfotomaculia;o__Desulfotomaculales;f__Desulfotomaculaceae;g__Desulfofarcimen;s__uncultured_bacterium | 0 |
| d__Bacteria;p__Gemmatimonadota;c__Gemmatimonadetes;o__Gemmatimonadales;f__Gemmatimonadaceae;g__uncultured;s__Gemmatimonadetes_bacterium | 0 |
| d__Bacteria;p__Dependentiae;c__Babeliae;o__Babeliales;f__UBA12411;g__UBA12411;__ | 0 |
| d__Bacteria;p__Proteobacteria;c__Alphaproteobacteria;o__Paracaedibacterales;f__Paracaedibacteraceae;g__Candidatus_Captivus;s__Candidatus_Captivus | 0 |
| d__Bacteria;p__Cyanobacteria;c__Cyanobacteriia;o__Chloroplast;f__Chloroplast;g__Chloroplast;s__uncultured_cyanobacterium | 0 |
| d__Bacteria;p__Proteobacteria;c__Gammaproteobacteria;o__Burkholderiales;f__Rhodocyclaceae;g__Uliginosibacterium;s__metagenome | 0 |
| d__Bacteria;p__Verrucomicrobiota;c__Verrucomicrobiae;o__Chthoniobacterales;f__Terrimicrobiaceae;g__FukuN18_freshwater_group;__ | 0 |
| d__Bacteria;p__Patescibacteria;c__Parcubacteria;o__Parcubacteria;f__Parcubacteria;g__Parcubacteria;s__uncultured_Parcubacteria | 0 |
| d__Bacteria;p__Bacteroidota;c__Bacteroidia;o__Sphingobacteriales;f__Sphingobacteriaceae;g__Nubsella;__ | 0 |
| d__Bacteria;p__Actinobacteriota;c__Actinobacteria;o__Streptosporangiales;__;__;__ | 0 |
| d__Bacteria;p__Verrucomicrobiota;c__Verrucomicrobiae;o__Chthoniobacterales;f__Xiphinematobacteraceae;g__Candidatus_Xiphinematobacter;s__uncultured_bacterium | 0 |
| d__Bacteria;p__Spirochaetota;c__Leptospirae;o__Leptospirales;f__Leptospiraceae;g__Turneriella;__ | 0 |
| d__Bacteria;p__Proteobacteria;c__Alphaproteobacteria;o__Rhizobiales;f__Hyphomicrobiaceae;g__Hyphomicrobium;s__uncultured_Alphaproteobacteria | 0 |
| d__Bacteria;p__Proteobacteria;c__Gammaproteobacteria;o__Gammaproteobacteria_Incertae_Sedis;f__Unknown_Family;g__Unknown_Family;__ | 0 |
| d__Bacteria;p__NB1-j;c__NB1-j;o__NB1-j;f__NB1-j;g__NB1-j;s__uncultured_delta | 0 |
| d__Bacteria;p__Firmicutes;c__Bacilli;o__Bacillales;f__Planococcaceae;g__Planococcus;s__Planococcus_maritimus | 0 |
| d__Bacteria;p__Proteobacteria;c__Alphaproteobacteria;o__Sphingomonadales;f__Sphingomonadaceae;g__Novosphingobium;s__Novosphingobium_resinovorum | 0 |
| d__Bacteria;p__Firmicutes;c__Clostridia;o__Peptostreptococcales-Tissierellales;f__Family_XI;g__Sporanaerobacter;__ | 0 |
| d__Bacteria;p__Firmicutes;c__Desulfotomaculia;o__Desulfotomaculales;f__Desulfotomaculaceae;g__Desulfohalotomaculum;s__Desulfotomaculum_sp. | 0 |
| d__Bacteria;p__Bacteroidota;c__Bacteroidia;o__Chitinophagales;f__Chitinophagaceae;g__Flavihumibacter;s__Flavihumibacter_sediminis | 0 |
| d__Bacteria;p__Bacteroidota;c__Bacteroidia;o__Flavobacteriales;f__Flavobacteriaceae;g__Flavobacterium;s__uncultured_Flavobacterium | 0 |
| d__Bacteria;p__Firmicutes;c__Bacilli;o__Paenibacillales;f__Paenibacillaceae;g__Paenibacillus;s__Paenibacillus_alvei | 0 |
| d__Bacteria;p__Proteobacteria;c__Alphaproteobacteria;o__Rickettsiales;f__Mitochondria;g__Mitochondria;s__Persicaria_minor | 0 |
| d__Bacteria;p__Proteobacteria;c__Gammaproteobacteria;o__Xanthomonadales;f__Xanthomonadaceae;g__Xanthomonas;s__Xanthomonas_sp. | 0 |
| d__Bacteria;p__Proteobacteria;c__Gammaproteobacteria;o__Burkholderiales;f__Methylophilaceae;g__Methylophilus;s__Methylophilus_luteus | 0 |
| d__Bacteria;p__Firmicutes;c__Bacilli;o__Bacillales;f__Planococcaceae;g__uncultured;s__uncultured_bacterium | 0 |
| d__Bacteria;p__Proteobacteria;c__Alphaproteobacteria;o__Caulobacterales;f__Caulobacteraceae;g__Brevundimonas;s__Brevundimonas_subvibrioides | 0 |
| d__Bacteria;p__Acidobacteriota;c__Acidobacteriae;o__Acidobacteriae;f__Acidobacteriae;g__Paludibaculum;s__metagenome | 0 |
| d__Bacteria;p__Actinobacteriota;c__Actinobacteria;o__Micromonosporales;f__Micromonosporaceae;g__uncultured;s__uncultured_bacterium | 0 |
| d__Bacteria;p__Bacteroidota;c__Bacteroidia;o__Cytophagales;f__Spirosomaceae;g__Flectobacillus;__ | 0 |
| d__Bacteria;p__Firmicutes;c__Clostridia;o__Peptococcales;f__Peptococcaceae;g__Peptococcaceae;s__uncultured_bacterium | 0 |
| d__Bacteria;p__Proteobacteria;c__Gammaproteobacteria;o__Burkholderiales;f__Comamonadaceae;g__Ramlibacter;s__Ramlibacter_sp. | 0 |
| d__Bacteria;p__Bdellovibrionota;c__Oligoflexia;o__053A03-B-DI-P58;f__053A03-B-DI-P58;g__053A03-B-DI-P58;s__uncultured_delta | 0 |
| d__Bacteria;p__Proteobacteria;c__Gammaproteobacteria;o__Burkholderiales;f__Burkholderiaceae;g__Ralstonia;s__Ralstonia_pickettii | 0 |
| d__Bacteria;p__Actinobacteriota;c__Actinobacteria;o__Micrococcales;f__Microbacteriaceae;g__Lysinimonas;s__Lysinimonas_kribbensis | 0 |
| d__Bacteria;p__Verrucomicrobiota;c__Verrucomicrobiae;o__Verrucomicrobiales;f__Rubritaleaceae;g__Luteolibacter;s__uncultured_Verrucomicrobiales | 0 |
| d__Bacteria;p__Firmicutes;c__Desulfotomaculia;o__Desulfotomaculales;f__Desulfotomaculales_Incertae_Sedis;g__Desulfallas-Sporotomaculum;s__Desulfotomaculum_sp. | 0 |
| d__Bacteria;p__Planctomycetota;c__Planctomycetes;o__Planctomycetales;f__Schlesneriaceae;g__Schlesneria;s__uncultured_planctomycete | 0 |
| d__Bacteria;p__Firmicutes;c__Clostridia;o__Peptostreptococcales-Tissierellales;f__Peptostreptococcaceae;g__Terrisporobacter;s__Terrisporobacter_glycolicus | 0 |
| d__Bacteria;p__Firmicutes;c__Bacilli;o__Bacillales;f__Bacillaceae;g__Bacillus;s__Bacillus_anthracis | 0 |
| d__Bacteria;p__Actinobacteriota;c__Actinobacteria;o__Micrococcales;f__Microbacteriaceae;g__uncultured;s__uncultured_soil | 0 |
| d__Bacteria;p__Cyanobacteria;c__Cyanobacteriia;o__Chloroplast;f__Chloroplast;g__Chloroplast;s__Helianthus_annuus | 0 |
| d__Bacteria;p__Cyanobacteria;c__Cyanobacteriia;o__Chloroplast;f__Chloroplast;g__Chloroplast;s__uncultured_phototrophic | 0 |
| d__Bacteria;p__Firmicutes;c__Clostridia;o__Eubacteriales;__;__;__ | 0 |
| d__Bacteria;p__Firmicutes;c__Bacilli;o__Alicyclobacillales;f__Alicyclobacillaceae;g__Tumebacillus;s__Tumebacillus_flagellatus | 0 |
| d__Bacteria;p__Firmicutes;c__Clostridia;o__Oscillospirales;f__Oscillospiraceae;g__UCG-002;s__uncultured_bacterium | 0 |
| d__Bacteria;p__Firmicutes;c__Clostridia;o__Clostridiales;f__Clostridiaceae;g__Clostridium_sensu_stricto_1;s__Clostridium_saudiense | 0 |
| d__Bacteria;p__Actinobacteriota;c__Actinobacteria;o__Micrococcales;f__Intrasporangiaceae;g__Janibacter;s__Janibacter_terrae | 0 |
| d__Bacteria;p__Proteobacteria;c__Gammaproteobacteria;o__Burkholderiales;f__Comamonadaceae;g__Methylibium;s__beta_proteobacterium | 0 |
| d__Bacteria;p__Actinobacteriota;c__Actinobacteria;o__Micrococcales;f__Micrococcaceae;g__Paeniglutamicibacter;s__Arthrobacter_sp. | 0 |
| d__Bacteria;p__Bacteroidota;c__Bacteroidia;o__Cytophagales;f__Amoebophilaceae;g__uncultured;s__uncultured_bacterium | 0 |
| d__Bacteria;p__Firmicutes;c__Bacilli;o__Entomoplasmatales;f__Entomoplasmataceae;__;__ | 0 |
| d__Bacteria;p__Proteobacteria;c__Gammaproteobacteria;o__Enterobacterales;f__Enterobacteriaceae;g__Kosakonia;s__Kosakonia_cowanii | 0 |
| d__Bacteria;p__Proteobacteria;c__Alphaproteobacteria;o__Rhizobiales;f__Hyphomicrobiaceae;g__Hyphomicrobium;s__uncultured_Hyphomicrobiaceae | 0 |
| d__Bacteria;p__Proteobacteria;c__Gammaproteobacteria;o__Legionellales;f__Legionellaceae;g__Legionella;s__Legionella_adelaidensis | 0 |
| d__Bacteria;p__Proteobacteria;c__Alphaproteobacteria;o__Rhizobiales;f__Devosiaceae;g__Devosia;s__uncultured_Devosia | 0 |
| d__Bacteria;p__Proteobacteria;c__Gammaproteobacteria;o__Burkholderiales;f__Comamonadaceae;g__Methylibium;s__uncultured_bacterium | 0 |
| d__Bacteria;p__Proteobacteria;c__Gammaproteobacteria;o__Burkholderiales;f__Rhodocyclaceae;g__Denitratisoma;__ | 0 |
| d__Bacteria;p__Proteobacteria;c__Alphaproteobacteria;o__Rhodobacterales;f__Rhodobacteraceae;g__Defluviimonas;s__uncultured_bacterium | 0 |
| d__Bacteria;p__Proteobacteria;c__Gammaproteobacteria;o__Burkholderiales;f__Rhodocyclaceae;g__Rhodocyclaceae;s__Azoarcus_olearius | 0 |
| d__Bacteria;p__Proteobacteria;c__Alphaproteobacteria;o__Micropepsales;f__Micropepsaceae;g__uncultured;s__uncultured_proteobacterium | 0 |
| d__Bacteria;p__Proteobacteria;c__Alphaproteobacteria;o__Rhizobiales;f__Xanthobacteraceae;g__Xanthobacteraceae;__ | 0 |
| d__Bacteria;p__Firmicutes;c__Bacilli;o__Bacillales;f__Bacillaceae;g__uncultured;s__uncultured_Halobacillus | 0 |
| d__Bacteria;p__Firmicutes;c__Bacilli;o__Alicyclobacillales;f__Alicyclobacillaceae;g__Alicyclobacillus;s__Alicyclobacillus_fastidiosus | 0 |
| d__Bacteria;p__Proteobacteria;c__Alphaproteobacteria;o__Rhodobacterales;f__Rhodobacteraceae;g__Rubellimicrobium;__ | 0 |
| d__Bacteria;p__Proteobacteria;c__Gammaproteobacteria;o__Gammaproteobacteria_Incertae_Sedis;f__Unknown_Family;g__Acidibacter;s__metagenome | 0 |
| d__Bacteria;p__Proteobacteria;c__Gammaproteobacteria;o__Salinisphaerales;f__Solimonadaceae;g__Fontimonas;s__uncultured_Xanthomonadaceae | 0 |
| d__Bacteria;p__Planctomycetota;c__Planctomycetes;o__Planctomycetales;f__Schlesneriaceae;g__Planctopirus;s__uncultured_planctomycete | 0 |
| d__Bacteria;p__Proteobacteria;c__Gammaproteobacteria;o__Burkholderiales;f__Rhodocyclaceae;g__Azonexus;s__Azonexus_caeni | 0 |
| d__Bacteria;p__Proteobacteria;c__Gammaproteobacteria;o__Burkholderiales;f__Rhodocyclaceae;g__Azonexus;s__uncultured_bacterium | 0 |
| d__Bacteria;p__Myxococcota;c__Myxococcia;o__Myxococcales;f__Myxococcaceae;g__Myxococcus;s__Myxococcus_fulvus | 0 |
| d__Bacteria;p__Proteobacteria;c__Gammaproteobacteria;o__Burkholderiales;f__Rhodocyclaceae;g__Dechlorosoma;__ | 0 |
| d__Bacteria;p__Bacteroidota;c__Kapabacteria;o__Kapabacteriales;f__Kapabacteriales;g__Kapabacteriales;s__uncultured_organism | 0 |
| d__Bacteria;p__Proteobacteria;c__Gammaproteobacteria;o__Burkholderiales;f__SC-I-84;g__SC-I-84;s__uncultured_soil | 0 |
| d__Bacteria;p__Bacteroidota;c__Bacteroidia;o__Cytophagales;f__Spirosomaceae;g__Arcicella;__ | 0 |
| d__Bacteria;p__Cyanobacteria;c__Cyanobacteriia;o__Leptolyngbyales;f__Leptolyngbyaceae;g__Oscillatoria_SAG_1459-8;__ | 0 |
| d__Bacteria;p__Proteobacteria;c__Gammaproteobacteria;o__Xanthomonadales;f__Xanthomonadaceae;g__Luteimonas;s__uncultured_Acidobacterium | 0 |
| d__Bacteria;p__Chloroflexi;c__Ktedonobacteria;o__Ktedonobacterales;f__Ktedonobacteraceae;g__uncultured;s__uncultured_Ktedonobacter | 0 |
| d__Bacteria;p__Proteobacteria;c__Alphaproteobacteria;o__Rhodospirillales;f__Magnetospirillaceae;g__Magnetospirillum;s__Magnetospirillum_gryphiswaldense | 0 |
| d__Bacteria;p__Cyanobacteria;c__Cyanobacteriia;o__Cyanobacteriales;f__Phormidiaceae;g__Cephalothrix_SAG_75.79;__ | 0 |
| d__Bacteria;p__Firmicutes;c__Clostridia;o__Peptostreptococcales-Tissierellales;f__Caminicellaceae;g__Caminicella;__ | 0 |
| d__Bacteria;p__Verrucomicrobiota;c__Verrucomicrobiae;o__Opitutales;__;__;__ | 0 |
| d__Bacteria;p__Bacteroidota;c__Bacteroidia;o__Sphingobacteriales;f__Sphingobacteriaceae;g__Sphingobacterium;s__Sphingobacterium_sp. | 0 |
| d__Bacteria;p__Proteobacteria;c__Alphaproteobacteria;o__Paracaedibacterales;f__Paracaedibacteraceae;g__Candidatus_Paracaedibacter;s__metagenome | 0 |
| d__Bacteria;p__Patescibacteria;c__Gracilibacteria;o__Candidatus_Peribacteria;f__Candidatus_Peribacteria;g__Candidatus_Peribacteria;s__uncultured_organism | 0 |
| d__Bacteria;p__Proteobacteria;c__Alphaproteobacteria;o__Rhizobiales;f__Xanthobacteraceae;g__Blastochloris;s__Blastochloris_viridis | 0 |
| d__Bacteria;p__Proteobacteria;c__Gammaproteobacteria;o__Burkholderiales;f__Comamonadaceae;g__Roseateles;s__Roseateles_depolymerans | 0 |
| d__Bacteria;p__Actinobacteriota;c__Actinobacteria;o__Micrococcales;f__Micrococcaceae;g__Glutamicibacter;__ | 0 |
| d__Bacteria;p__Cyanobacteria;c__Cyanobacteriia;o__Leptolyngbyales;f__Leptolyngbyaceae;g__Leptolyngbya_ANT.L52.2;s__uncultured_bacterium | 0 |
| d__Bacteria;p__Proteobacteria;c__Gammaproteobacteria;o__Burkholderiales;f__Rhodocyclaceae;g__Dechlorobacter;__ | 0 |
| d__Bacteria;p__Acidobacteriota;c__Acidobacteriae;o__Subgroup_2;f__Subgroup_2;g__Subgroup_2;s__uncultured_Acidobacteriales | 0 |
| d__Bacteria;p__Cyanobacteria;c__Vampirivibrionia;o__Caenarcaniphilales;f__Caenarcaniphilales;g__Caenarcaniphilales;__ | 0 |
| d__Bacteria;p__Proteobacteria;c__Gammaproteobacteria;o__Xanthomonadales;f__Rhodanobacteraceae;g__Rudaea;__ | 0 |
| d__Bacteria;p__Firmicutes;c__Clostridia;o__Peptococcales;f__Peptococcaceae;g__Desulfitispora;__ | 0 |
| d__Bacteria;p__Verrucomicrobiota;c__Verrucomicrobiae;o__Opitutales;f__Puniceicoccaceae;g__Verruc-01;s__uncultured_bacterium | 0 |
| d__Bacteria;p__Proteobacteria;c__Alphaproteobacteria;o__Rhizobiales;f__Beijerinckiaceae;g__Bosea;s__bacterium_KR | 0 |
| d__Bacteria;p__Firmicutes;c__Clostridia;o__Lachnospirales;f__Lachnospiraceae;g__Epulopiscium;s__uncultured_bacterium | 0 |
| d__Bacteria;p__Gemmatimonadota;c__Gemmatimonadetes;o__Gemmatimonadales;f__Gemmatimonadaceae;g__uncultured;s__uncultured_Gemmatimonas | 0 |
| d__Bacteria;p__Proteobacteria;c__Alphaproteobacteria;o__Rhodobacterales;f__Rhodobacteraceae;g__Paracoccus;s__Paracoccus_aminovorans | 0 |
| d__Bacteria;p__Firmicutes;c__Clostridia;o__Clostridiales;f__Caloramatoraceae;g__uncultured;s__Clostridium_sp. | 0 |
| d__Bacteria;p__Proteobacteria;c__Gammaproteobacteria;o__Chromatiales;f__Chromatiaceae;__;__ | 0 |
| d__Bacteria;p__Proteobacteria;c__Alphaproteobacteria;o__Rhodospirillales;f__Magnetospirillaceae;g__Magnetospirillum;s__Magnetospirillum_magnetotacticum | 0 |
| d__Bacteria;p__Bacteroidota;c__Bacteroidia;o__Chitinophagales;f__Chitinophagaceae;g__Chitinophaga;s__uncultured_Chitinophaga | 0 |
| d__Bacteria;p__Desulfobacterota;c__Desulfuromonadia;o__Geobacterales;f__Geobacteraceae;g__Citrifermentans;s__uncultured_delta | 0 |
| d__Bacteria;p__Proteobacteria;c__Alphaproteobacteria;o__Rhizobiales;f__Amb-16S-1323;g__Amb-16S-1323;__ | 0 |
| d__Bacteria;p__Proteobacteria;c__Alphaproteobacteria;o__Rhizobiales;f__Xanthobacteraceae;g__Starkeya;s__Starkeya_koreensis | 0 |
| d__Bacteria;p__Proteobacteria;c__Gammaproteobacteria;o__Burkholderiales;f__Comamonadaceae;g__Diaphorobacter;s__uncultured_Acidovorax | 0 |
| d__Bacteria;p__Proteobacteria;c__Gammaproteobacteria;o__F9P41300-M23;f__F9P41300-M23;g__F9P41300-M23;s__uncultured_bacterium | 0 |
| d__Bacteria;p__Proteobacteria;c__Alphaproteobacteria;o__Holosporales;f__Holosporaceae;g__Candidatus_Bealeia;s__uncultured_bacterium | 0 |
| d__Bacteria;p__Planctomycetota;c__Phycisphaerae;o__Tepidisphaerales;f__WD2101_soil_group;g__WD2101_soil_group;s__uncultured_Planctomycetales | 0 |
| d__Bacteria;p__Firmicutes;c__Clostridia;o__Peptostreptococcales-Tissierellales;f__Anaerovoracaceae;g__uncultured;__ | 0 |
| d__Bacteria;p__Proteobacteria;c__Alphaproteobacteria;o__Rhodobacterales;f__Rhodobacteraceae;g__Rhodobacter;s__Rhodobacter_sphaeroides | 0 |
| d__Bacteria;p__Firmicutes;c__Bacilli;o__Bacillales;f__Bacillaceae;g__Anoxybacillus;s__Anoxybacillus_kamchatkensis | 0 |
| d__Bacteria;p__Proteobacteria;c__Alphaproteobacteria;o__Rhizobiales;f__Beijerinckiaceae;g__Methylocella;__ | 0 |
| d__Bacteria;p__Bdellovibrionota;c__Bdellovibrionia;o__Bdellovibrionales;f__Bdellovibrionaceae;g__Bdellovibrio;s__Bdellovibrio_sp. | 0 |
| d__Bacteria;p__Firmicutes;c__Clostridia;o__Clostridiales;f__Clostridiaceae;g__Clostridium_sensu_stricto_1;s__Clostridium_sp. | 0 |
| d__Bacteria;p__Proteobacteria;c__Gammaproteobacteria;o__Burkholderiales;f__Comamonadaceae;g__Simplicispira;__ | 0 |
| d__Bacteria;p__Firmicutes;c__Negativicutes;o__Veillonellales-Selenomonadales;f__Sporomusaceae;g__Sporomusa;s__uncultured_bacterium | 0 |
| d__Bacteria;p__Proteobacteria;c__Gammaproteobacteria;o__Burkholderiales;f__Rhodocyclaceae;g__Sulfuritalea;s__uncultured_beta | 0 |
| d__Bacteria;p__Spirochaetota;c__Spirochaetia;o__Spirochaetales;f__Spirochaetaceae;g__Salinispira;s__metagenome | 0 |
| d__Bacteria;p__Proteobacteria;c__Gammaproteobacteria;o__Pseudomonadales;f__Alcanivoracaceae1;g__Alcanivorax;s__Alcanivorax_sp. | 0 |
| d__Bacteria;p__Proteobacteria;c__Alphaproteobacteria;o__Rhizobiales;f__Rhizobiaceae;g__Bartonella;__ | 0 |
| d__Bacteria;p__Proteobacteria;c__Gammaproteobacteria;o__Xanthomonadales;f__Xanthomonadaceae;g__Xanthomonas;s__Xanthomonas_citri | 0 |
| d__Bacteria;p__Bacteroidota;c__Bacteroidia;o__Sphingobacteriales;f__Sphingobacteriaceae;g__Mucilaginibacter;s__uncultured_Bacteroidetes | 0 |
| d__Bacteria;p__Cyanobacteria;c__Cyanobacteriia;o__Chloroplast;f__Chloroplast;g__Chloroplast;s__Illicium_verum | 0 |
| d__Bacteria;p__Cyanobacteria;c__Cyanobacteriia;o__Chloroplast;f__Chloroplast;g__Chloroplast;s__Nasturtium_officinale | 0 |
| d__Bacteria;p__Proteobacteria;c__Gammaproteobacteria;o__Pseudomonadales;f__Pseudomonadaceae;g__Pseudomonas;s__Pseudomonas_fulva | 0 |
| d__Bacteria;p__Verrucomicrobiota;c__Verrucomicrobiae;o__Opitutales;f__Puniceicoccaceae;g__Cerasicoccus;s__uncultured_organism | 0 |
| d__Bacteria;p__Bacteroidota;c__Bacteroidia;o__Cytophagales;f__Spirosomaceae;g__Larkinella;__ | 0 |
| d__Bacteria;p__Firmicutes;c__Bacilli;o__Bacillales;f__Bacillaceae;g__Bacillus;s__Bacillus_muralis | 0 |
| d__Bacteria;p__Proteobacteria;c__Alphaproteobacteria;o__Rhizobiales;f__Rhizobiaceae;g__Mesorhizobium;s__Rhizobium_sp. | 0 |
| d__Bacteria;p__Proteobacteria;c__Alphaproteobacteria;o__Kordiimonadales;f__Kordiimonadaceae;g__Kordiimonas;__ | 0 |
| d__Bacteria;p__Cyanobacteria;c__Cyanobacteriia;o__Chloroplast;f__Chloroplast;g__Chloroplast;s__Citrus_maxima | 0 |
| d__Bacteria;p__Bacteroidota;c__Bacteroidia;o__Sphingobacteriales;f__env.OPS_17;g__env.OPS_17;s__metagenome | 0 |
| d__Bacteria;p__Proteobacteria;c__Alphaproteobacteria;o__Rhizobiales;f__Rhizobiaceae;g__Aminobacter;s__uncultured_bacterium | 0 |
| d__Bacteria;p__Proteobacteria;c__Gammaproteobacteria;o__Enterobacterales;f__Enterobacteriaceae;g__Raoultella;s__Raoultella_planticola | 0 |
| d__Bacteria;p__Proteobacteria;c__Gammaproteobacteria;o__Gammaproteobacteria_Incertae_Sedis;f__Unknown_Family;g__Unknown_Family;s__metagenome | 0 |
| d__Bacteria;p__Proteobacteria;c__Alphaproteobacteria;o__Paracaedibacterales;f__Paracaedibacteraceae;g__uncultured;s__uncultured_Alphaproteobacteria | 0 |
| d__Bacteria;p__Actinobacteriota;c__Actinobacteria;o__Streptomycetales;f__Streptomycetaceae;g__Streptomyces;s__Streptomyces_antibioticus | 0 |
| d__Bacteria;p__Cyanobacteria;c__Vampirivibrionia;o__Caenarcaniphilales;f__Caenarcaniphilales;g__Caenarcaniphilales;s__uncultured_bacterium | 0 |
| d__Bacteria;p__Actinobacteriota;c__Actinobacteria;o__Corynebacteriales;f__Nocardiaceae;g__Gordonia;s__Gordonia_terrae | 0 |
| d__Bacteria;p__Cyanobacteria;c__Cyanobacteriia;o__Chloroplast;f__Chloroplast;g__Chloroplast;s__Petunia_exserta | 0 |
| d__Bacteria;p__Cyanobacteria;c__Cyanobacteriia;o__Chloroplast;f__Chloroplast;g__Chloroplast;s__uncultured_eukaryote | 0 |
| d__Bacteria;p__Patescibacteria;c__Parcubacteria;o__Candidatus_Vogelbacteria;f__Candidatus_Vogelbacteria;g__Candidatus_Vogelbacteria;s__uncultured_bacterium | 0 |
| d__Bacteria;p__Verrucomicrobiota;c__Verrucomicrobiae;o__Methylacidiphilales;f__Methylacidiphilaceae;g__uncultured;s__metagenome | 0 |
| d__Bacteria;p__Proteobacteria;c__Alphaproteobacteria;o__Rhizobiales;f__Xanthobacteraceae;g__Blastochloris;__ | 0 |
| d__Bacteria;p__Cyanobacteria;c__Cyanobacteriia;o__Cyanobacteriales;f__Nostocaceae;g__Nostoc_PCC-7107;s__Nostoc_sp. | 0 |
| d__Bacteria;p__Cyanobacteria;c__Cyanobacteriia;o__Chloroplast;f__Chloroplast;g__Chloroplast;s__Ammopiptanthus_mongolicus | 0 |
| d__Bacteria;p__Cyanobacteria;c__Cyanobacteriia;o__Chloroplast;f__Chloroplast;g__Chloroplast;s__Brassica_napus | 0 |
| d__Bacteria;p__Bdellovibrionota;c__Bdellovibrionia;o__Bdellovibrionales;f__Bdellovibrionaceae;g__OM27_clade;s__uncultured_delta | 0 |
| d__Bacteria;p__Bdellovibrionota;c__Bdellovibrionia;o__Bdellovibrionales;f__Bdellovibrionaceae;g__Bdellovibrio;s__uncultured_organism | 0 |
| d__Bacteria;p__Firmicutes;c__Bacilli;o__Bacillales;f__Bacillaceae;g__Vulcanibacillus;__ | 0 |
| d__Bacteria;p__Myxococcota;c__Myxococcia;o__Myxococcales;f__Anaeromyxobacteraceae;g__Anaeromyxobacter;s__bacterium_enrichment | 0 |
| d__Bacteria;p__Bacteroidota;c__Bacteroidia;o__Flavobacteriales;f__Cryomorphaceae;g__NS10_marine_group;__ | 0 |
| d__Bacteria;p__Verrucomicrobiota;c__Verrucomicrobiae;__;__;__;__ | 0 |
| d__Bacteria;p__Proteobacteria;c__Alphaproteobacteria;o__Rickettsiales;f__Rickettsiaceae;g__uncultured;s__metagenome | 0 |
| d__Bacteria;p__Proteobacteria;c__Alphaproteobacteria;o__Rhodobacterales;f__Rhodobacteraceae;g__Falsirhodobacter;__ | 0 |
| d__Bacteria;p__Planctomycetota;c__Planctomycetes;o__Gemmatales;f__Gemmataceae;g__Gemmata;s__Gemmata_sp. | 0 |
| d__Bacteria;p__Proteobacteria;c__Gammaproteobacteria;o__Diplorickettsiales;f__Diplorickettsiaceae;g__Aquicella;s__uncultured_soil | 0 |
| d__Bacteria;p__Cyanobacteria;c__Cyanobacteriia;o__Chloroplast;f__Chloroplast;g__Chloroplast;s__Chloroplast_transformation | 0 |
| d__Bacteria;p__Firmicutes;c__Limnochordia;o__M55-D21;f__M55-D21;g__M55-D21;s__uncultured_bacterium | 0 |
| d__Bacteria;p__Proteobacteria;c__Alphaproteobacteria;o__Zavarziniales;f__Zavarziniaceae;__;__ | 0 |
| d__Bacteria;p__Actinobacteriota;c__Actinobacteria;o__Corynebacteriales;f__Mycobacteriaceae;g__Mycobacterium;s__Mycobacterium_phlei | 0 |
| d__Bacteria;p__Bacteroidota;c__Bacteroidia;o__Bacteroidales;f__Prolixibacteraceae;__;__ | 0 |
| d__Bacteria;p__Proteobacteria;c__Alphaproteobacteria;o__Rhizobiales;f__Rhizobiales_Incertae_Sedis;g__Bauldia;s__uncultured_Alphaproteobacteria | 0 |
| d__Bacteria;p__Actinobacteriota;c__Acidimicrobiia;o__Microtrichales;f__uncultured;g__uncultured;s__uncultured_compost | 0 |
| d__Bacteria;p__Patescibacteria;c__ABY1;o__Candidatus_Uhrbacteria;f__Candidatus_Uhrbacteria;g__Candidatus_Uhrbacteria;s__Candidatus_Uhrbacteria | 0 |
| d__Bacteria;p__Acidobacteriota;c__Thermoanaerobaculia;o__Thermoanaerobaculales;f__Thermoanaerobaculaceae;g__Thermoanaerobaculum;__ | 0 |
| d__Bacteria;p__Desulfobacterota;c__Desulfuromonadia;o__Geobacterales;f__Geobacteraceae;g__Trichlorobacter;s__uncultured_bacterium | 0 |
| d__Bacteria;p__Desulfobacterota;c__Desulfuromonadia;o__Geobacterales;f__Geobacteraceae;g__Trichlorobacter;__ | 0 |
| d__Bacteria;p__Bacteroidota;c__Bacteroidia;o__Bacteroidales;f__Marinifilaceae;g__uncultured;__ | 0 |
| d__Bacteria;p__Planctomycetota;c__Phycisphaerae;o__Phycisphaerales;f__Phycisphaeraceae;g__SM1A02;s__uncultured_Planctomycetaceae | 0 |
| d__Bacteria;p__Bacteroidota;c__Bacteroidia;o__Bacteroidales;f__Prolixibacteraceae;g__Meniscus;s__Meniscus_glaucopis | 0 |
| d__Bacteria;p__Proteobacteria;c__Gammaproteobacteria;o__Burkholderiales;f__Rhodocyclaceae;g__Dechloromonas;s__Dechloromonas_sp. | 0 |
| d__Bacteria;p__Proteobacteria;c__Alphaproteobacteria;o__Rhizobiales;f__Pleomorphomonadaceae;g__Chthonobacter;s__uncultured_bacterium | 0 |
| d__Bacteria;p__Proteobacteria;c__Gammaproteobacteria;o__Pseudomonadales;f__Spongiibacteraceae;__;__ | 0 |
| d__Bacteria;p__Proteobacteria;c__Alphaproteobacteria;o__Sphingomonadales;f__Sphingomonadaceae;g__Altererythrobacter;s__metagenome | 0 |
| d__Bacteria;p__Bdellovibrionota;c__Bdellovibrionia;o__Bacteriovoracales;f__Bacteriovoracaceae;g__uncultured;s__uncultured_bacterium | 0 |
| d__Bacteria;p__Desulfobacterota;c__Desulfuromonadia;o__Bradymonadales;f__Bradymonadales;g__Bradymonadales;s__uncultured_bacterium | 0 |
| d__Bacteria;p__Proteobacteria;c__Gammaproteobacteria;o__Salinisphaerales;f__Solimonadaceae;g__Hydrocarboniphaga;s__uncultured_bacterium | 0 |
| d__Bacteria;p__Proteobacteria;c__Alphaproteobacteria;o__Acetobacterales;f__Acetobacteraceae;g__Roseomonas;s__Roseomonas_cervicalis | 0 |
| d__Bacteria;p__Proteobacteria;c__Alphaproteobacteria;o__Acetobacterales;f__Acetobacteraceae;g__Roseomonas;s__uncultured_bacterium | 0 |
| d__Bacteria;p__Firmicutes;c__Bacilli;o__Acholeplasmatales;f__Acholeplasmataceae;g__Acholeplasma;s__Acholeplasma_sp. | 0 |
| d__Bacteria;p__Bacteroidota;c__Bacteroidia;o__Bacteroidales;f__Bacteroidaceae;g__Bacteroides;__ | 0 |
| d__Bacteria;p__Proteobacteria;c__Alphaproteobacteria;o__Rhizobiales;f__Xanthobacteraceae;g__Pseudoxanthobacter;s__uncultured_Alphaproteobacteria | 0 |
| d__Bacteria;p__Proteobacteria;c__Gammaproteobacteria;o__Pseudomonadales;f__Alcanivoracaceae;g__Ketobacter;s__uncultured_soil | 0 |
| d__Bacteria;p__Acidobacteriota;c__Subgroup_18;o__Subgroup_18;f__Subgroup_18;g__Subgroup_18;__ | 0 |
| d__Bacteria;p__Proteobacteria;c__Gammaproteobacteria;o__Xanthomonadales;f__Xanthomonadaceae;g__Pseudoxanthomonas;s__Azotobacter_chroococcum | 0 |
| d__Bacteria;p__Bacteroidota;c__Bacteroidia;o__Bacteroidales;f__Bacteroidaceae;g__Bacteroides;s__uncultured_bacterium | 0 |
| d__Bacteria;p__Proteobacteria;c__Gammaproteobacteria;o__Burkholderiales;f__Rhodocyclaceae;g__Dechloromonas;s__metagenome | 0 |
| d__Bacteria;p__Proteobacteria;c__Gammaproteobacteria;o__Burkholderiales;f__Rhodocyclaceae;g__Thauera;s__uncultured_Thauera | 0 |
| d__Bacteria;p__Desulfobacterota;c__Desulfuromonadia;o__Geobacterales;f__Geobacteraceae;g__Geobacter;s__Geobacter_sulfurreducens | 0 |
| d__Bacteria;p__Proteobacteria;c__Gammaproteobacteria;o__Enterobacterales;f__Enterobacteriaceae;g__Enterobacter;s__uncultured_bacterium | 0 |
| d__Bacteria;p__Bacteroidota;c__Bacteroidia;o__Chitinophagales;f__uncultured;g__uncultured;s__uncultured_Saprospiraceae | 0 |
| d__Bacteria;p__Patescibacteria;c__Parcubacteria;o__Candidatus_Nomurabacteria;f__Candidatus_Nomurabacteria;g__Candidatus_Nomurabacteria;s__uncultured_bacterium | 0 |
| d__Bacteria;p__Proteobacteria;c__Alphaproteobacteria;o__Acetobacterales;f__Acetobacteraceae;g__Rhodovarius;__ | 0 |
| d__Bacteria;p__Desulfobacterota;c__Desulfuromonadia;o__Desulfuromonadales;f__Desulfuromonadaceae;g__Desulfuromonadaceae;__ | 0 |
| d__Bacteria;p__Bacteroidota;c__Bacteroidia;o__Bacteroidales;f__Dysgonomonadaceae;g__Fermentimonas;s__uncultured_bacterium | 0 |
| d__Bacteria;p__Hydrogenedentes;c__Hydrogenedentia;o__Hydrogenedentiales;f__Hydrogenedensaceae;g__YC-ZSS-LKJ63;__ | 0 |
| d__Bacteria;p__Gemmatimonadota;c__S0134_terrestrial_group;o__S0134_terrestrial_group;f__S0134_terrestrial_group;g__S0134_terrestrial_group;s__metagenome | 0 |
| d__Bacteria;p__Proteobacteria;c__Gammaproteobacteria;o__Enterobacterales;f__Hafniaceae;g__Edwardsiella;__ | 0 |
| d__Bacteria;p__Actinobacteriota;c__Actinobacteria;o__Micrococcales;__;__;__ | 0 |
| d__Bacteria;p__Firmicutes;c__Clostridia;o__Peptostreptococcales-Tissierellales;f__Peptostreptococcaceae;g__Romboutsia;s__uncultured_organism | 0 |
| d__Bacteria;p__Firmicutes;c__Bacilli;o__Bacillales;f__Bacillaceae;g__uncultured;__ | 0 |
| d__Bacteria;p__Firmicutes;c__Bacilli;o__Paenibacillales;f__Paenibacillaceae;g__Paenibacillaceae;s__uncultured_bacterium | 0 |
| d__Bacteria;p__Planctomycetota;c__Planctomycetes;o__Gemmatales;f__Gemmataceae;g__uncultured;s__uncultured_Planctomycetaceae | 0 |
| d__Bacteria;p__Acidobacteriota;c__Acidobacteriae;o__Acidobacteriales;f__Acidobacteriaceae_(Subgroup_1);g__Granulicella;s__Edaphobacter_acidisoli | 0 |
| d__Bacteria;p__Firmicutes;c__Desulfotomaculia;o__Desulfotomaculales;f__Desulfotomaculaceae;g__Desulfohalotomaculum;s__Desulfohalotomaculum_tongense | 0 |
| d__Bacteria;p__Firmicutes;c__Bacilli;o__Alicyclobacillales;f__Alicyclobacillaceae;g__Alicyclobacillus;s__Alicyclobacillus_pomorum | 0 |
| d__Bacteria;p__Proteobacteria;c__Alphaproteobacteria;o__Rhodospirillales;f__uncultured;g__uncultured;s__uncultured_Magnetospirillum | 0 |
| d__Bacteria;p__Proteobacteria;c__Gammaproteobacteria;o__Burkholderiales;f__Burkholderiaceae;g__Pandoraea;s__Pandoraea_sputorum | 0 |
| d__Bacteria;p__Firmicutes;c__Clostridia;o__Lachnospirales;f__Lachnospiraceae;g__Sellimonas;s__uncultured_bacterium | 0 |
| d__Bacteria;p__Proteobacteria;c__Gammaproteobacteria;o__Burkholderiales;f__Burkholderiaceae;g__Burkholderia-Caballeronia-Paraburkholderia;s__Burkholderia_ambifaria | 0 |
| d__Bacteria;p__Firmicutes;c__Clostridia;o__Lachnospirales;f__Lachnospiraceae;g__[Ruminococcus]_gauvreauii_group;__ | 0 |
| d__Bacteria;p__Actinobacteriota;c__Thermoleophilia;__;__;__;__ | 0 |
| d__Bacteria;p__Planctomycetota;c__Phycisphaerae;o__MSBL9;f__GWC2-45-44;g__GWC2-45-44;s__uncultured_Planctomycetaceae | 0 |
| d__Bacteria;p__Planctomycetota;c__Phycisphaerae;o__MSBL9;f__SM23-30;g__SM23-30;s__uncultured_bacterium | 0 |
| d__Bacteria;p__Firmicutes;c__Clostridia;o__Oscillospirales;f__Hungateiclostridiaceae;g__Pseudobacteroides;s__uncultured_bacterium | 0 |
| d__Bacteria;p__Firmicutes;c__Bacilli;o__Paenibacillales;f__Paenibacillaceae;g__Paenibacillus;s__uncultured_Firmicutes | 0 |
| d__Bacteria;p__Proteobacteria;c__Gammaproteobacteria;o__Burkholderiales;f__Nitrosomonadaceae;g__GOUTA6;s__metagenome | 0 |
| d__Bacteria;p__Elusimicrobiota;c__Lineage_IIa;o__Lineage_IIa;f__Lineage_IIa;g__Lineage_IIa;s__uncultured_soil | 0 |
| d__Bacteria;p__Armatimonadota;c__Chthonomonadetes;o__Chthonomonadales;f__Chthonomonadales;g__Chthonomonadales;s__uncultured_Lutispora | 0 |
| d__Bacteria;p__Proteobacteria;c__Alphaproteobacteria;o__Sphingomonadales;f__Sphingomonadaceae;g__Sphingopyxis;s__Sphingopyxis_sp. | 0 |
| d__Bacteria;p__Acidobacteriota;c__Blastocatellia;o__Pyrinomonadales;f__Pyrinomonadaceae;g__RB41;s__uncultured_Acidobacteria | 0 |
| d__Bacteria;p__Proteobacteria;c__Gammaproteobacteria;o__Burkholderiales;f__Rhodocyclaceae;g__uncultured;s__uncultured_Georgfuchsia | 0 |
| d__Bacteria;p__Firmicutes;c__Bacilli;o__Lactobacillales;f__Lactobacillaceae;g__Weissella;s__Weissella_koreensis | 0 |
| d__Bacteria;p__Firmicutes;c__Bacilli;o__Bacillales;f__Planococcaceae;g__Domibacillus;s__uncultured_bacterium | 0 |
| d__Bacteria;p__Planctomycetota;c__Planctomycetes;o__Gemmatales;f__Gemmataceae;g__Fimbriiglobus;s__uncultured_planctomycete | 0 |
| d__Bacteria;p__Firmicutes;c__Bacilli;o__Bacillales;f__Bacillaceae;g__Bacillus;s__Bacillus_abyssalis | 0 |
| d__Bacteria;p__Proteobacteria;c__Alphaproteobacteria;o__Rhizobiales;f__Hyphomicrobiaceae;g__Pedomicrobium;s__uncultured_Pedomicrobium | 0 |
| d__Bacteria;p__Fibrobacterota;c__Fibrobacteria;o__Fibrobacterales;f__Fibrobacterales;g__BBMC-4;s__metagenome | 0 |
| d__Bacteria;p__Bacteroidota;c__Bacteroidia;o__Bacteroidales;f__Dysgonomonadaceae;g__Petrimonas;__ | 0 |
| d__Bacteria;p__Bacteroidota;c__Bacteroidia;o__Bacteroidales;f__Dysgonomonadaceae;g__Proteiniphilum;s__uncultured_bacterium | 0 |
| d__Bacteria;p__Firmicutes;c__Bacilli;o__Kyrpidiales;f__Kyrpidiaceae;g__Kyrpidia;s__uncultured_bacterium | 0 |
| d__Bacteria;p__Proteobacteria;c__Alphaproteobacteria;o__Parvibaculales;f__Parvibaculaceae;g__Parvibaculum;s__uncultured_Alphaproteobacteria | 0 |
| d__Bacteria;p__Proteobacteria;c__Alphaproteobacteria;o__uncultured;f__uncultured;g__uncultured;s__uncultured_Xanthobacteraceae | 0 |
| d__Bacteria;p__Proteobacteria;c__Gammaproteobacteria;o__Burkholderiales;f__Burkholderiaceae;g__Lautropia;s__metagenome | 0 |
| d__Bacteria;p__Proteobacteria;c__Gammaproteobacteria;o__Burkholderiales;f__Sutterellaceae;g__uncultured;__ | 0 |
| d__Bacteria;p__Bacteroidota;c__Bacteroidia;o__Bacteroidales;f__Marinilabiliaceae;g__uncultured;s__uncultured_bacterium | 0 |
| d__Bacteria;p__Proteobacteria;c__Alphaproteobacteria;o__Rhizobiales;f__Hyphomicrobiaceae;g__Hyphomicrobium;s__metagenome | 0 |
| d__Bacteria;p__Firmicutes;c__Bacilli;o__Thermoactinomycetales;f__Thermoactinomycetaceae;g__Pasteuria;s__uncultured_bacterium | 0 |
| d__Bacteria;p__Proteobacteria;c__Gammaproteobacteria;o__Burkholderiales;f__Alcaligenaceae;g__Verticiella;__ | 0 |
| d__Bacteria;p__Actinobacteriota;c__Actinobacteria;o__Corynebacteriales;f__Nocardiaceae;g__Gordonia;s__Gordonia_effusa | 0 |
| d__Bacteria;p__Planctomycetota;c__Planctomycetes;o__Gemmatales;f__Gemmataceae;g__Tuwongella;s__uncultured_bacterium | 0 |
| d__Bacteria;p__Calditrichota;c__Calditrichia;o__Calditrichales;f__Calditrichaceae;g__Calditrichaceae;s__uncultured_organism | 0 |
| d__Bacteria;p__Firmicutes;c__Bacilli;o__Thermoactinomycetales;f__Thermoactinomycetaceae;g__Shimazuella;__ | 0 |
| d__Bacteria;p__Bacteroidota;c__Bacteroidia;o__Chitinophagales;f__Chitinophagaceae;g__Segetibacter;s__uncultured_bacterium | 0 |
| d__Bacteria;p__Firmicutes;c__Bacilli;o__Paenibacillales;f__Paenibacillaceae;g__Paenibacillus;s__Paenibacillus_timonensis | 0 |
| d__Bacteria;p__Proteobacteria;c__Alphaproteobacteria;o__Caulobacterales;f__Caulobacteraceae;g__Phenylobacterium;s__uncultured_Caulobacter | 0 |
| d__Bacteria;p__Patescibacteria;c__CPR2;o__CPR2;f__CPR2;g__CPR2;s__uncultured_microorganism | 0 |
| d__Bacteria;p__Aquificota;c__Aquificae;o__Aquificales;f__Aquificaceae;g__Hydrogenobacter;__ | 0 |
| d__Bacteria;p__Chloroflexi;c__Anaerolineae;o__Caldilineales;f__Caldilineaceae;g__uncultured;s__metagenome | 0 |
| d__Bacteria;p__Firmicutes;c__Bacilli;o__Lactobacillales;f__Lactobacillaceae;g__Lactiplantibacillus;s__Lactobacillus_plantarum | 0 |
| d__Bacteria;p__Planctomycetota;c__Phycisphaerae;o__MSBL9;f__4572-13;g__4572-13;__ | 0 |
| d__Bacteria;p__Cyanobacteria;c__Cyanobacteriia;o__Cyanobacteriales;f__Chroococcidiopsaceae;__;__ | 0 |
| d__Bacteria;p__Bacteroidota;c__Bacteroidia;o__Chitinophagales;f__Saprospiraceae;g__OLB8;__ | 0 |
| d__Bacteria;p__Myxococcota;c__Polyangia;o__Haliangiales;f__Haliangiaceae;g__Haliangium;s__uncultured_Nannocystineae | 0 |
| d__Bacteria;p__Firmicutes;c__Desulfotomaculia;o__Desulfotomaculales;f__Desulfotomaculaceae;g__Desulfotomaculum;s__Desulfotomaculum_hydrothermale | 0 |
| d__Bacteria;p__Firmicutes;c__Limnochordia;o__Limnochordia;f__Limnochordia;g__Hydrogenispora;s__uncultured_low | 0 |
| d__Bacteria;p__Acidobacteriota;c__Vicinamibacteria;o__Vicinamibacterales;f__uncultured;g__uncultured;s__metagenome | 0 |
| d__Bacteria;p__Chloroflexi;c__Chloroflexia;o__Thermomicrobiales;f__JG30-KF-CM45;g__JG30-KF-CM45;s__uncultured_Chloroflexi | 0 |
| d__Bacteria;p__Acidobacteriota;c__Thermoanaerobaculia;o__Thermoanaerobaculales;f__Thermoanaerobaculaceae;g__Subgroup_10;s__uncultured_Acidobacteria | 0 |
| d__Bacteria;p__Proteobacteria;c__Alphaproteobacteria;o__Rhodobacterales;f__Rhodobacteraceae;g__Paracoccus;s__uncultured_bacterium | 0 |
| d__Bacteria;p__Proteobacteria;c__Gammaproteobacteria;o__Burkholderiales;f__Burkholderiaceae;g__Burkholderia-Caballeronia-Paraburkholderia;s__Burkholderia_pyrrocinia | 0 |
| d__Bacteria;p__Acidobacteriota;c__Acidobacteriae;o__Bryobacterales;f__Bryobacteraceae;g__Bryobacter;s__uncultured_Acidobacteriaceae | 0 |
| d__Bacteria;p__Patescibacteria;c__Microgenomatia;o__Candidatus_Collierbacteria;f__Candidatus_Collierbacteria;g__Candidatus_Collierbacteria;s__uncultured_Microgenomates | 0 |
| d__Bacteria;p__Verrucomicrobiota;c__Verrucomicrobiae;o__Verrucomicrobiales;f__Rubritaleaceae;g__Luteolibacter;s__Verrucomicrobiaceae_bacterium | 0 |
| d__Bacteria;p__Proteobacteria;c__Gammaproteobacteria;o__Burkholderiales;f__Nitrosomonadaceae;g__Ellin6067;s__uncultured_delta | 0 |
| d__Bacteria;p__Proteobacteria;c__Alphaproteobacteria;o__Sphingomonadales;f__Sphingomonadaceae;g__Sphingobium;s__Sphingomonas_azotofornans | 0 |
| d__Bacteria;p__Proteobacteria;c__Alphaproteobacteria;o__Rhizobiales;f__Hyphomicrobiaceae;g__Hyphomicrobium;s__Hyphomicrobium_aestuarii | 0 |
| d__Bacteria;p__Myxococcota;c__Polyangia;o__Polyangiales;f__Polyangiaceae;g__Jahnella;s__Jahnella_thaxteri | 0 |
| d__Bacteria;p__Spirochaetota;c__Leptospirae;o__Leptospirales;f__Leptospiraceae;g__RBG-16-49-21;s__uncultured_organism | 0 |
| d__Bacteria;p__Bacteroidota;c__Ignavibacteria;o__Ignavibacteriales;f__BSV40;g__BSV40;__ | 0 |
| d__Bacteria;p__Actinobacteriota;c__Thermoleophilia;o__Solirubrobacterales;f__Solirubrobacteraceae;g__Solirubrobacter;s__uncultured_Solirubrobacter | 0 |
| d__Bacteria;p__Acidobacteriota;c__Holophagae;o__Subgroup_7;f__Subgroup_7;g__Subgroup_7;s__uncultured_Acidobacteria | 0 |
| d__Bacteria;p__Patescibacteria;c__Saccharimonadia;o__Saccharimonadales;f__Saccharimonadales;g__Saccharimonadales;s__metagenome | 0 |
| d__Bacteria;p__Planctomycetota;c__Planctomycetes;o__Isosphaerales;f__Isosphaeraceae;g__uncultured;s__metagenome | 0 |
| d__Bacteria;p__Firmicutes;c__Clostridia;o__Lachnospirales;f__Lachnospiraceae;g__Mobilitalea;__ | 0 |
| d__Bacteria;p__Firmicutes;c__Desulfotomaculia;o__Desulfotomaculales;f__Desulfurisporaceae;g__Desulfurispora;s__uncultured_Desulfotomaculum | 0 |
| d__Bacteria;p__Bacteroidota;c__Bacteroidia;o__Bacteroidales;f__Prolixibacteraceae;g__uncultured;__ | 0 |
| d__Bacteria;p__Patescibacteria;c__Dojkabacteria;o__Dojkabacteria;f__Dojkabacteria;g__Dojkabacteria;s__uncultured_Alphaproteobacteria | 0 |
| d__Bacteria;p__Bacteroidota;c__Bacteroidia;o__Cytophagales;f__Microscillaceae;g__Hassallia;s__metagenome | 0 |
| d__Bacteria;p__Firmicutes;c__Moorellia;o__Moorellales;f__Moorellaceae;g__Moorella;s__uncultured_Clostridia | 0 |
| d__Bacteria;p__Bacteroidota;c__Bacteroidia;o__Bacteroidales;f__Prevotellaceae;g__Paraprevotella;s__uncultured_bacterium | 0 |
| d__Bacteria;p__Proteobacteria;c__Gammaproteobacteria;o__Pseudomonadales;f__Pseudomonadaceae;g__Pseudomonas;s__Pseudomonas_panipatensis | 0 |
| d__Bacteria;p__Proteobacteria;c__Gammaproteobacteria;o__Xanthomonadales;f__Rhodanobacteraceae;g__Frateuria;s__Frateuria_aurantia | 0 |
| d__Bacteria;p__Verrucomicrobiota;c__Chlamydiae;o__Chlamydiales;f__Parachlamydiaceae;__;__ | 0 |
| d__Bacteria;p__Chloroflexi;c__Chloroflexia;o__Thermomicrobiales;f__Thermomicrobiaceae;__;__ | 0 |
| d__Bacteria;p__Proteobacteria;c__Alphaproteobacteria;o__Rhizobiales;f__Rhizobiaceae;g__Allorhizobium-Neorhizobium-Pararhizobium-Rhizobium;s__Rhizobium_sp.KAs5-22 | 0 |
| d__Bacteria;p__Proteobacteria;c__Gammaproteobacteria;o__Nitrosococcales;f__Nitrosococcaceae;g__Nitrosococcus;s__Nitrosococcus_oceani | 0 |
| d__Bacteria;p__Proteobacteria;c__Alphaproteobacteria;o__Reyranellales;f__Reyranellaceae;g__Reyranella;s__Enhydrobacter_aerosaccus | 0 |
| d__Bacteria;p__Bacteroidota;c__Bacteroidia;o__Chitinophagales;f__Chitinophagaceae;g__UTBCD1;s__uncultured_bacterium | 0 |
| d__Bacteria;p__Chloroflexi;c__Ktedonobacteria;o__Ktedonobacterales;f__Ktedonobacteraceae;g__uncultured;s__uncultured_soil | 0 |
| d__Bacteria;p__Proteobacteria;c__Gammaproteobacteria;o__Burkholderiales;f__Burkholderiaceae;g__Burkholderia-Caballeronia-Paraburkholderia;s__Trinickia_caryophylli | 0 |
| d__Bacteria;p__Actinobacteriota;c__Actinobacteria;o__Micrococcales;f__Intrasporangiaceae;g__Janibacter;s__Janibacter_hoylei | 0 |
| d__Bacteria;p__Proteobacteria;c__Gammaproteobacteria;o__Burkholderiales;f__Methylophilaceae;g__MM1;s__uncultured_bacterium | 0 |
| d__Bacteria;p__Actinobacteriota;c__Actinobacteria;o__Micrococcales;f__Promicromonosporaceae;__;__ | 0 |
| d__Bacteria;p__Firmicutes;c__Clostridia;o__Lachnospirales;f__Lachnospiraceae;g__Oribacterium;__ | 0 |
| d__Bacteria;p__Proteobacteria;c__Alphaproteobacteria;o__Caulobacterales;f__Caulobacteraceae;g__Asticcacaulis;s__Asticcacaulis_solisilvae | 0 |
| d__Bacteria;p__Halanaerobiaeota;c__Halanaerobiia;o__Halanaerobiales;f__Halanaerobiaceae;g__Halocella;__ | 0 |
| d__Bacteria;p__Firmicutes;c__Bacilli;o__Bacillales;f__Marinococcaceae;__;__ | 0 |
| d__Bacteria;p__Proteobacteria;c__Gammaproteobacteria;o__Enterobacterales;f__Pasteurellaceae;g__Haemophilus;s__Haemophilus_haemolyticus | 0 |
| d__Bacteria;p__Planctomycetota;c__Planctomycetes;o__Planctomycetales;f__Rubinisphaeraceae;g__Planctomicrobium;s__uncultured_Planctomyces | 0 |
| d__Bacteria;p__Proteobacteria;c__Gammaproteobacteria;o__Burkholderiales;f__Burkholderiaceae;g__Burkholderia-Caballeronia-Paraburkholderia;s__Paraburkholderia_phenoliruptrix | 0 |
| d__Bacteria;p__Chloroflexi;c__Ktedonobacteria;o__Ktedonobacterales;f__Ktedonobacteraceae;g__HSB_OF53-F07;s__uncultured_Ktedonobacter | 0 |
| d__Bacteria;p__Proteobacteria;c__Gammaproteobacteria;o__Burkholderiales;f__Comamonadaceae;g__Hydrogenophaga;s__Hydrogenophaga_temperata | 0 |
| d__Bacteria;p__Myxococcota;c__Myxococcia;o__Myxococcales;f__Vulgatibacteraceae;g__Vulgatibacter;s__uncultured_compost | 0 |
| d__Bacteria;p__Bdellovibrionota;c__Oligoflexia;o__Oligoflexales;f__Oligoflexaceae;g__uncultured;s__uncultured_delta | 0 |
| d__Bacteria;p__Proteobacteria;c__Gammaproteobacteria;o__Enterobacterales;f__Enterobacteriaceae;g__Enterobacillus;s__uncultured_Pantoea | 0 |
| d__Bacteria;p__Proteobacteria;c__Alphaproteobacteria;o__Caulobacterales;f__Caulobacteraceae;g__Caulobacter;s__Caulobacter_fusiformis | 0 |
| d__Bacteria;p__Firmicutes;c__Clostridia;o__Lachnospirales;f__Lachnospiraceae;g__Butyrivibrio;s__uncultured_bacterium | 0 |
| d__Bacteria;p__Elusimicrobiota;c__Elusimicrobia;o__MVP-88;f__MVP-88;g__MVP-88;__ | 0 |
| d__Bacteria;p__Proteobacteria;c__Gammaproteobacteria;o__Pseudomonadales;f__Nitrincolaceae;g__Nitrincola;__ | 0 |
| d__Bacteria;p__Firmicutes;c__Bacilli;o__Lactobacillales;f__Streptococcaceae;g__Streptococcus;s__Streptococcus_mutans | 0 |
| d__Bacteria;p__Dependentiae;c__Babeliae;o__Babeliales;f__Vermiphilaceae;g__Vermiphilaceae;s__uncultured_organism | 0 |
| d__Bacteria;p__Firmicutes;c__Bacilli;o__Paenibacillales;f__Paenibacillaceae;g__Paenibacillus;s__Paenibacillus_odorifer | 0 |
| d__Bacteria;p__Proteobacteria;c__Alphaproteobacteria;o__Ferrovibrionales;f__Ferrovibrionales;g__Taonella;s__metagenome | 0 |
| d__Bacteria;p__Hydrogenedentes;c__Hydrogenedentia;o__Hydrogenedentiales;f__Hydrogenedensaceae;__;__ | 0 |
| d__Bacteria;p__Actinobacteriota;c__Thermoleophilia;o__Solirubrobacterales;f__Solirubrobacteraceae;g__JCM_18997;__ | 0 |
| d__Bacteria;p__Proteobacteria;c__Alphaproteobacteria;o__Rhizobiales;f__Xanthobacteraceae;g__Pseudolabrys;s__uncultured_Rhizobiales | 0 |
| d__Bacteria;p__Actinobacteriota;c__Actinobacteria;o__Corynebacteriales;f__Mycobacteriaceae;g__Mycobacterium;s__Mycobacterium_tuberculosis | 0 |
| d__Bacteria;p__Firmicutes;c__Bacilli;o__Bacillales;f__Bacillaceae;g__Fictibacillus;s__Fictibacillus_barbaricus | 0 |
| d__Bacteria;p__Acidobacteriota;c__Acidobacteriae;o__Acidobacteriales;f__uncultured;g__uncultured;s__uncultured_soil | 0 |
| d__Bacteria;p__Actinobacteriota;c__Actinobacteria;o__Corynebacteriales;f__Nocardiaceae;g__Rhodococcus;s__Rhodococcus_ruber | 0 |
| d__Bacteria;p__Firmicutes;c__Bacilli;o__Caldalkalibacillales;f__Caldalkalibacillaceae;g__Caldalkalibacillus;s__Caldalkalibacillus_uzonensis | 0 |
| d__Bacteria;p__Firmicutes;c__Clostridia;o__Peptostreptococcales-Tissierellales;f__Family_XI;g__Peptoniphilus;s__uncultured_bacterium | 0 |
| d__Bacteria;p__Bacteroidota;c__Bacteroidia;o__Cytophagales;f__Spirosomaceae;g__uncultured;s__uncultured_bacterium | 0 |
| d__Bacteria;p__Bacteroidota;c__Bacteroidia;o__Chitinophagales;f__Chitinophagaceae;g__Chitinophaga;s__uncultured_bacterium | 0 |
| d__Bacteria;p__Actinobacteriota;c__Actinobacteria;o__Streptosporangiales;f__Thermomonosporaceae;g__Thermomonospora;__ | 0 |
| d__Bacteria;p__Myxococcota;c__Myxococcia;o__Myxococcales;f__Myxococcaceae;g__Aggregicoccus;s__Aggregicoccus_edonensis | 0 |
| d__Bacteria;p__Planctomycetota;c__Planctomycetes;o__Gemmatales;f__Gemmataceae;g__Gemmata;s__uncultured_Gemmata | 0 |
| d__Bacteria;p__Proteobacteria;c__Alphaproteobacteria;o__Rhizobiales;f__Devosiaceae;g__Devosia;s__Devosia_geojensis | 0 |
| d__Bacteria;p__Bacteroidota;c__Bacteroidia;o__Chitinophagales;f__Chitinophagaceae;g__Cnuella;s__uncultured_bacterium | 0 |
| d__Bacteria;p__Proteobacteria;c__Alphaproteobacteria;o__Sphingomonadales;f__Sphingomonadaceae;g__uncultured;s__uncultured_Sphingomonadales | 0 |
| d__Bacteria;p__Actinobacteriota;c__Actinobacteria;o__Streptomycetales;f__Streptomycetaceae;g__Kitasatospora;__ | 0 |
| d__Bacteria;p__Chloroflexi;c__Chloroflexia;o__Kallotenuales;f__AKIW781;g__AKIW781;__ | 0 |
| d__Bacteria;p__Firmicutes;c__Bacilli;o__Bacillales;f__Bacillaceae;g__Bacillus;s__Bacillus_vietnamensis | 0 |
| d__Bacteria;p__Actinobacteriota;c__Actinobacteria;o__Pseudonocardiales;f__Pseudonocardiaceae;g__Pseudonocardia;s__Pseudonocardia_kunmingensis | 0 |
| d__Bacteria;p__Proteobacteria;c__Alphaproteobacteria;o__Rhodobacterales;f__Rhodobacteraceae;g__Haematobacter;__ | 0 |
| d__Bacteria;p__Actinobacteriota;c__Actinobacteria;o__Catenulisporales;f__Catenulisporaceae;g__Catenulispora;__ | 0 |
| d__Bacteria;p__Bacteroidota;c__Bacteroidia;o__Sphingobacteriales;f__Sphingobacteriaceae;g__Pedobacter;s__Pedobacter_tournemirensis | 0 |
| d__Bacteria;p__Bacteroidota;c__Bacteroidia;o__Bacteroidales;f__Bacteroidaceae;g__Bacteroides;s__Bacteroides_fragilis | 0 |
| d__Bacteria;p__Firmicutes;c__Desulfitobacteriia;o__Desulfitobacteriales;f__Desulfitobacteriaceae;g__Desulfosporosinus;s__Desulfosporosinus_acidiphilus | 0 |
| d__Bacteria;p__Proteobacteria;c__Alphaproteobacteria;o__Rhodobacterales;f__Rhodobacteraceae;g__Gemmobacter;__ | 0 |
| d__Bacteria;p__Bacteroidota;c__Bacteroidia;o__Sphingobacteriales;f__Sphingobacteriaceae;g__Sphingobacteriaceae;s__Pedobacter_sp. | 0 |
| d__Bacteria;p__Proteobacteria;c__Gammaproteobacteria;o__Burkholderiales;f__Alcaligenaceae;g__GKS98_freshwater_group;s__uncultured_bacterium | 0 |
| d__Bacteria;p__Firmicutes;c__Bacilli;o__Paenibacillales;f__Paenibacillaceae;g__Paenibacillus;s__Paenibacillus_riograndensis | 0 |
| d__Bacteria;p__Firmicutes;c__Bacilli;o__Paenibacillales;f__Paenibacillaceae;g__Paenibacillus;s__Paenibacillus_durus | 0 |
| d__Bacteria;p__Firmicutes;c__Bacilli;o__Paenibacillales;f__Paenibacillaceae;g__Paenibacillus;s__Paenibacillus_glycanilyticus | 0 |
| d__Bacteria;p__Proteobacteria;c__Gammaproteobacteria;o__Pseudomonadales;f__211ds20;g__211ds20;s__uncultured_beta | 0 |
| d__Bacteria;p__Proteobacteria;c__Gammaproteobacteria;o__Burkholderiales;f__Comamonadaceae;g__Curvibacter;s__Curvibacter_fontanus | 0 |
| d__Bacteria;p__Chloroflexi;c__Ktedonobacteria;o__Ktedonobacterales;f__Ktedonobacteraceae;g__HSB_OF53-F07;__ | 0 |
| d__Bacteria;p__Bacteroidota;c__Bacteroidia;o__Sphingobacteriales;f__Sphingobacteriaceae;g__Pedobacter;s__Pedobacter_sp. | 0 |
| d__Bacteria;p__Firmicutes;c__Symbiobacteriia;o__Symbiobacteriales;f__uncultured;g__uncultured;s__uncultured_Clostridia | 0 |
| d__Bacteria;p__Proteobacteria;c__Alphaproteobacteria;o__Azospirillales;f__Azospirillaceae;g__Azospirillum;s__uncultured_Azospirillum | 0 |
| d__Bacteria;p__Firmicutes;c__Bacilli;o__Bacillales;f__Salisediminibacteriaceae;g__Salipaludibacillus;__ | 0 |
| d__Bacteria;p__Proteobacteria;c__Alphaproteobacteria;o__Rhizobiales;f__Rhizobiaceae;g__Allorhizobium-Neorhizobium-Pararhizobium-Rhizobium;s__Rhizobium_endolithicum | 0 |
| d__Bacteria;p__Proteobacteria;c__Alphaproteobacteria;o__Rhizobiales;f__Beijerinckiaceae;g__Psychroglaciecola;s__uncultured_bacterium | 0 |
| d__Bacteria;p__Proteobacteria;c__Gammaproteobacteria;o__Pseudomonadales;f__Pseudomonadaceae;g__Pseudomonas;s__Pseudomonas_furukawaii | 0 |
| d__Bacteria;p__Proteobacteria;c__Alphaproteobacteria;o__Azospirillales;f__Azospirillaceae;g__Azospirillum;s__Azospirillum_oryzae | 0 |
| d__Bacteria;p__Firmicutes;c__Bacilli;o__Bacillales;f__Bacillaceae;g__Bacillus;s__Bacillus_alkalinitrilicus | 0 |
| d__Bacteria;p__Proteobacteria;c__Gammaproteobacteria;o__Xanthomonadales;f__Xanthomonadaceae;g__Vulcaniibacterium;s__uncultured_gamma | 0 |
| d__Bacteria;p__Firmicutes;c__Clostridia;o__Peptostreptococcales-Tissierellales;f__Peptostreptococcaceae;g__Intestinibacter;s__uncultured_bacterium | 0 |
| d__Bacteria;p__Firmicutes;c__Clostridia;o__Clostridia_UCG-014;f__Clostridia_UCG-014;g__Clostridia_UCG-014;s__uncultured_rumen | 0 |
| d__Bacteria;p__Planctomycetota;c__Planctomycetes;o__Isosphaerales;f__Isosphaeraceae;g__uncultured;s__uncultured_Planctomycetaceae | 0 |
| d__Bacteria;p__Proteobacteria;c__Gammaproteobacteria;o__Burkholderiales;f__Rhodocyclaceae;g__Methyloversatilis;s__uncultured_Methyloversatilis | 0 |
| d__Bacteria;p__Firmicutes;c__Bacilli;o__Bacillales;f__Planococcaceae;g__Sporosarcina;s__Sporosarcina_sp. | 0 |
| d__Bacteria;p__Firmicutes;c__Bacilli;o__Paenibacillales;f__Paenibacillaceae;g__uncultured;s__uncultured_Paenibacillus | 0 |
| d__Bacteria;p__Proteobacteria;c__Gammaproteobacteria;o__Burkholderiales;f__Methylophilaceae;g__uncultured;s__metagenome | 0 |
| d__Bacteria;p__Desulfobacterota;c__Desulfuromonadia;o__Desulfuromonadales;f__Desulfuromonadaceae;g__Desulfuromonas;s__uncultured_bacterium | 0 |
| d__Bacteria;p__Firmicutes;c__Clostridia;o__Proteinivoracales;f__Proteinivoracales;g__Proteinivoracales;s__uncultured_bacterium | 0 |
| d__Bacteria;p__Proteobacteria;c__Gammaproteobacteria;o__Pseudomonadales;f__Pseudomonadaceae;g__Pseudomonas;s__Pseudomonas_linyingensis | 0 |
| d__Bacteria;p__Proteobacteria;c__Alphaproteobacteria;o__Rhizobiales;f__Rhizobiaceae;g__Allorhizobium-Neorhizobium-Pararhizobium-Rhizobium;s__Rhizobium_lemnae | 0 |
| d__Bacteria;p__Firmicutes;c__Clostridia;o__Peptostreptococcales-Tissierellales;f__Anaerovoracaceae;g__Family_XIII_UCG-001;__ | 0 |
| d__Bacteria;p__Acidobacteriota;c__Blastocatellia;o__11-24;f__11-24;g__11-24;s__uncultured_Acidobacteria | 0 |
| d__Bacteria;p__Chloroflexi;c__Anaerolineae;o__SJA-15;f__SJA-15;g__SJA-15;__ | 0 |
| d__Bacteria;p__Spirochaetota;c__Leptospirae;o__Leptospirales;f__Leptospiraceae;g__uncultured;__ | 0 |
| d__Bacteria;p__Planctomycetota;c__Planctomycetes;o__Gemmatales;f__Gemmataceae;g__uncultured;s__uncultured_soil | 0 |
| d__Bacteria;p__Actinobacteriota;c__Actinobacteria;o__Frankiales;f__Geodermatophilaceae;g__Modestobacter;__ | 0 |
| d__Bacteria;p__Firmicutes;c__Bacilli;o__Paenibacillales;f__Paenibacillaceae;g__Cohnella;s__Paenibacillus_sp. | 0 |
| d__Bacteria;p__Proteobacteria;c__Gammaproteobacteria;o__Enterobacterales;f__Erwiniaceae;g__Erwinia;__ | 0 |
| d__Bacteria;p__Firmicutes;c__Desulfitobacteriia;o__Desulfitobacteriales;f__Desulfitobacteriaceae;g__Desulfitobacterium;s__Desulfitobacterium_dehalogenans | 0 |
| d__Bacteria;p__Firmicutes;c__Desulfitobacteriia;o__Desulfitobacteriales;f__Desulfitobacteriaceae;g__Desulfitobacterium;s__uncultured_bacterium | 0 |
| d__Bacteria;p__Proteobacteria;c__Alphaproteobacteria;o__Rhizobiales;f__Beijerinckiaceae;g__Methylosinus;s__uncultured_Alphaproteobacteria | 0 |
| d__Bacteria;p__Sva0485;c__Sva0485;o__Sva0485;f__Sva0485;g__Sva0485;__ | 0 |
| d__Bacteria;p__Actinobacteriota;c__Actinobacteria;o__Streptosporangiales;f__Thermomonosporaceae;g__Actinocorallia;s__uncultured_bacterium | 0 |
| d__Bacteria;p__Proteobacteria;c__Gammaproteobacteria;o__Burkholderiales;f__Alcaligenaceae;g__GKS98_freshwater_group;__ | 0 |
| d__Bacteria;p__Firmicutes;c__TTA-B61;o__TTA-B61;f__TTA-B61;g__TTA-B61;__ | 0 |
| d__Bacteria;p__Patescibacteria;c__Parcubacteria;o__Candidatus_Colwellbacteria;f__Candidatus_Colwellbacteria;g__Candidatus_Colwellbacteria;s__uncultured_bacterium | 0 |
| d__Bacteria;p__Firmicutes;c__Clostridia;o__Lachnospirales;f__Defluviitaleaceae;g__Defluviitaleaceae_UCG-011;s__uncultured_organism | 0 |
| d__Bacteria;p__Firmicutes;c__Thermovenabulia;o__Thermovenabulales;f__Thermovenabulales;__;__ | 0 |
| d__Bacteria;p__Acidobacteriota;c__Acidobacteriae;o__Acidobacteriae;f__Acidobacteriae;g__Paludibaculum;s__uncultured_soil | 0 |
| d__Bacteria;p__Firmicutes;c__Bacilli;o__Exiguobacterales;f__Exiguobacteraceae;g__Exiguobacterium;s__Exiguobacterium_sibiricum | 0 |
| d__Bacteria;p__Firmicutes;c__Clostridia;o__Peptostreptococcales-Tissierellales;f__Peptostreptococcaceae;g__Romboutsia;s__Romboutsia_hominis | 0 |
| d__Bacteria;p__Proteobacteria;c__Alphaproteobacteria;o__Rhizobiales;f__D05-2;g__D05-2;s__Rhizobiales_bacterium | 0 |
| d__Bacteria;p__Proteobacteria;c__Gammaproteobacteria;o__Steroidobacterales;f__Steroidobacteraceae;g__Steroidobacter;s__metagenome | 0 |
| d__Bacteria;p__Proteobacteria;c__Gammaproteobacteria;o__Xanthomonadales;f__Xanthomonadaceae;g__Lysobacter;s__Lysobacter_daejeonensis | 0 |
| d__Bacteria;p__Chloroflexi;c__Chloroflexia;o__Thermomicrobiales;f__JG30-KF-CM45;g__JG30-KF-CM45;s__uncultured_soil | 0 |
| d__Bacteria;p__Firmicutes;c__Bacilli;o__Acholeplasmatales;f__Acholeplasmataceae;__;__ | 0 |
| d__Bacteria;p__Firmicutes;c__Incertae_Sedis;o__Incertae_Sedis;f__Incertae_Sedis;g__Gelria;s__Gelria_glutamica | 0 |
| d__Bacteria;p__Actinobacteriota;c__Actinobacteria;o__Propionibacteriales;f__Propionibacteriaceae;g__Micropruina;__ | 0 |
| d__Bacteria;p__Proteobacteria;c__Gammaproteobacteria;o__Ectothiorhodospirales;f__Ectothiorhodospiraceae;g__Thiogranum;s__uncultured_bacterium | 0 |
| d__Bacteria;p__Actinobacteriota;c__RBG-16-55-12;o__RBG-16-55-12;f__RBG-16-55-12;g__RBG-16-55-12;__ | 0 |
| d__Bacteria;p__Planctomycetota;c__Phycisphaerae;o__Tepidisphaerales;f__Tepidisphaeraceae;g__Tepidisphaeraceae;__ | 0 |
| d__Bacteria;p__Actinobacteriota;c__Actinobacteria;o__Micromonosporales;f__Micromonosporaceae;g__Catellatospora;__ | 0 |
| d__Bacteria;p__Acidobacteriota;c__Acidobacteriae;o__GOUTB8;f__GOUTB8;g__GOUTB8;s__uncultured_bacterium | 0 |
| d__Bacteria;p__Firmicutes;c__Bacilli;o__Bacillales;f__Planococcaceae;g__Planomicrobium;s__uncultured_Firmicutes | 0 |
| d__Bacteria;p__Firmicutes;c__Bacilli;o__Lactobacillales;f__Lactobacillaceae;g__Weissella;__ | 0 |
| d__Bacteria;p__Cyanobacteria;c__Cyanobacteriia;o__Chloroplast;f__Chloroplast;g__Chloroplast;s__Cannabis_sativa | 0 |
| d__Bacteria;p__Firmicutes;c__Clostridia;o__Clostridia;f__Gracilibacteraceae;g__Gracilibacter;s__Clostridiaceae_bacterium | 0 |
| d__Bacteria;p__Actinobacteriota;c__Actinobacteria;o__Micrococcales;f__Dermabacteraceae;g__Brachybacterium;__ | 0 |
| d__Bacteria;p__Proteobacteria;c__Alphaproteobacteria;o__Rhodobacterales;f__Rhodobacteraceae;g__Paracoccus;s__Paracoccus_sp. | 0 |
| d__Bacteria;p__Chloroflexi;c__Anaerolineae;o__RBG-13-54-9;f__RBG-13-54-9;g__RBG-13-54-9;s__uncultured_Chloroflexi | 0 |
| d__Bacteria;p__Actinobacteriota;c__Thermoleophilia;o__Solirubrobacterales;f__Solirubrobacteraceae;g__JCM_18997;s__uncultured_bacterium | 0 |
| d__Bacteria;p__Firmicutes;c__Negativicutes;o__Acidaminococcales;f__Acidaminococcaceae;g__uncultured;s__uncultured_bacterium | 0 |
| d__Bacteria;p__Acidobacteriota;c__Vicinamibacteria;o__Vicinamibacterales;f__Vicinamibacteraceae;g__Vicinamibacteraceae;s__metagenome | 0 |
| d__Bacteria;p__Proteobacteria;c__Alphaproteobacteria;o__Rhizobiales;f__Xanthobacteraceae;g__uncultured;s__uncultured_Alphaproteobacteria | 0 |
| d__Bacteria;p__Firmicutes;c__Clostridia;o__Clostridiales;f__Clostridiaceae;g__Clostridium_sensu_stricto_12;s__uncultured_Clostridium | 0 |
| d__Bacteria;p__Proteobacteria;c__Gammaproteobacteria;o__CCD24;f__CCD24;g__CCD24;s__metagenome | 0 |
| d__Bacteria;p__Myxococcota;c__Polyangia;o__Polyangiales;f__Polyangiaceae;g__uncultured;s__metagenome | 0 |
| d__Bacteria;p__Proteobacteria;c__Alphaproteobacteria;o__Alphaproteobacteria_Incertae_Sedis;f__Unknown_Family;g__Acuticoccus;__ | 0 |
| d__Bacteria;p__Proteobacteria;c__Gammaproteobacteria;o__Pseudomonadales;f__Pseudomonadaceae;g__Pseudomonas;s__Pseudomonas_rhizosphaerae | 0 |
| d__Bacteria;p__Armatimonadota;c__Armatimonadia;o__Armatimonadales;f__Armatimonadales;g__Armatimonadales;s__uncultured_organism | 0 |
| d__Bacteria;p__Acidobacteriota;c__Acidobacteriae;o__Bryobacterales;f__Bryobacteraceae;g__Bryobacter;s__uncultured_soil | 0 |
| d__Bacteria;p__Planctomycetota;c__OM190;o__OM190;f__OM190;g__OM190;s__uncultured_Planctomycetales | 0 |
| d__Bacteria;p__Actinobacteriota;c__Actinobacteria;o__Micrococcales;f__Demequinaceae;g__Lysinimicrobium;__ | 0 |
| d__Bacteria;p__Actinobacteriota;c__Actinobacteria;o__Pseudonocardiales;f__Pseudonocardiaceae;g__Actinophytocola;__ | 0 |
| d__Bacteria;p__Actinobacteriota;c__Coriobacteriia;o__Coriobacteriales;f__Eggerthellaceae;__;__ | 0 |
| d__Bacteria;p__Bacteroidota;c__Bacteroidia;o__Bacteroidales;f__Dysgonomonadaceae;g__Proteiniphilum;__ | 0 |
| d__Bacteria;p__Chloroflexi;c__Anaerolineae;__;__;__;__ | 0 |
| d__Bacteria;p__Actinobacteriota;c__Actinobacteria;o__Micrococcales;f__Microbacteriaceae;g__Agromyces;s__Agromyces_sp. | 0 |
| d__Bacteria;p__Firmicutes;c__Bacilli;o__Bacillales;f__Sporolactobacillaceae;g__Tuberibacillus;s__Caenibacillus_caldisaponilyticus | 0 |
| d__Bacteria;p__Firmicutes;c__Thermacetogenia;o__Thermacetogeniales;f__Thermacetogeniaceae;g__uncultured;__ | 0 |
| d__Bacteria;p__Proteobacteria;c__Alphaproteobacteria;o__Sphingomonadales;f__Sphingomonadaceae;g__Altererythrobacter;s__uncultured_Alphaproteobacteria | 0 |
| d__Bacteria;p__Chloroflexi;c__Dehalococcoidia;o__Dehalococcoidales;f__Dehalococcoidaceae;g__Dehalogenimonas;__ | 0 |
| d__Bacteria;p__Chloroflexi;c__OLB14;o__OLB14;f__OLB14;g__OLB14;s__metagenome | 0 |
| d__Bacteria;p__Firmicutes;c__Bacilli;o__Thermoactinomycetales;f__Thermoactinomycetaceae;g__Laceyella;s__Laceyella_sacchari | 0 |
| d__Bacteria;p__Proteobacteria;c__Gammaproteobacteria;o__Burkholderiales;f__Comamonadaceae;g__Piscinibacter;s__uncultured_soil | 0 |
| d__Bacteria;p__Proteobacteria;c__Gammaproteobacteria;o__Diplorickettsiales;f__Diplorickettsiaceae;g__Aquicella;s__Aquicella_lusitana | 0 |
| d__Bacteria;p__Firmicutes;c__Clostridia;o__Peptostreptococcales-Tissierellales;f__Anaerovoracaceae;g__Family_XIII_AD3011_group;s__uncultured_organism | 0 |
| d__Bacteria;p__Bacteroidota;c__Bacteroidia;o__Bacteroidales;f__SB-5;g__SB-5;s__uncultured_soil | 0 |
| d__Bacteria;p__Chloroflexi;c__Chloroflexia;o__Chloroflexales;f__Roseiflexaceae;g__Kouleothrix;s__uncultured_bacterium | 0 |
| d__Bacteria;p__Bacteroidota;c__Bacteroidia;o__Chitinophagales;f__Chitinophagaceae;g__Terrimonas;s__uncultured_soil | 0 |
| d__Bacteria;p__Firmicutes;c__Clostridia;o__Oscillospirales;f__Butyricicoccaceae;__;__ | 0 |
| d__Bacteria;p__Firmicutes;c__Clostridia;o__Oscillospirales;f__Butyricicoccaceae;g__UCG-009;s__uncultured_bacterium | 0 |
| d__Bacteria;p__Proteobacteria;c__Alphaproteobacteria;o__Tistrellales;f__Geminicoccaceae;__;__ | 0 |
| d__Bacteria;p__Proteobacteria;c__Gammaproteobacteria;o__Burkholderiales;f__Alcaligenaceae;g__Castellaniella;s__Castellaniella_daejeonensis | 0 |
| d__Bacteria;p__Campylobacterota;c__Campylobacteria;o__Campylobacterales;f__Arcobacteraceae;g__uncultured;s__uncultured_bacterium | 0 |
| d__Bacteria;p__Firmicutes;c__Bacilli;o__Lactobacillales;f__Aerococcaceae;g__Aerococcus;s__Aerococcus_urinaeequi | 0 |
| d__Bacteria;p__Actinobacteriota;c__Actinobacteria;o__Micrococcales;f__Microbacteriaceae;g__Humibacter;s__uncultured_bacterium | 0 |
| d__Bacteria;p__Firmicutes;c__Clostridia;o__Caldicoprobacterales;f__Caldicoprobacteraceae;g__Caldicoprobacter;s__anaerobic_digester | 0 |
| d__Bacteria;p__Acidobacteriota;c__Acidobacteriae;o__Acidobacteriales;f__Acidobacteriaceae_(Subgroup_1);g__Acidipila-Silvibacterium;s__Acidipila_sp. | 0 |
| d__Bacteria;p__Proteobacteria;c__Alphaproteobacteria;o__Defluviicoccales;f__Defluviicoccaceae;g__Defluviicoccus;s__uncultured_Alphaproteobacteria | 0 |
| d__Bacteria;p__Firmicutes;c__Clostridia;o__Peptostreptococcales-Tissierellales;f__Peptostreptococcales-Tissierellales;g__Gottschalkia;__ | 0 |
| d__Bacteria;p__Actinobacteriota;c__Actinobacteria;o__Frankiales;f__Acidothermaceae;g__Acidothermus;s__uncultured_actinobacterium | 0 |
| d__Bacteria;p__Chloroflexi;c__Ktedonobacteria;o__Ktedonobacterales;f__Ktedonobacteraceae;g__uncultured;s__uncultured_Ktedobacteria | 0 |
| d__Bacteria;p__Actinobacteriota;c__Acidimicrobiia;o__uncultured;f__uncultured;g__uncultured;s__uncultured_Actinomycetales | 0 |
| d__Bacteria;p__Actinobacteriota;c__Actinobacteria;o__Frankiales;f__uncultured;g__uncultured;s__metagenome | 0 |
| d__Bacteria;p__Proteobacteria;c__Alphaproteobacteria;o__Elsterales;f__URHD0088;g__URHD0088;s__bacterium_Ellin5299 | 0 |
| d__Bacteria;p__Actinobacteriota;c__Thermoleophilia;o__Gaiellales;f__uncultured;g__uncultured;s__uncultured_Conexibacter | 0 |
| d__Bacteria;p__Firmicutes;c__Bacilli;o__Paenibacillales;f__Paenibacillaceae;g__Paenibacillus;s__Paenibacillus_bovis | 0 |
| d__Bacteria;p__Firmicutes;c__Dethiobacteria;o__Dethiobacterales;f__Dethiobacteraceae;__;__ | 0 |
| d__Bacteria;p__Firmicutes;c__Clostridia;o__Oscillospirales;f__Hungateiclostridiaceae;g__uncultured;s__uncultured_Clostridium | 0 |
| d__Bacteria;p__Firmicutes;c__Bacilli;o__Erysipelotrichales;f__Erysipelotrichaceae;g__Turicibacter;s__Turicibacter_sp. | 0 |
| d__Bacteria;p__Actinobacteriota;c__Actinobacteria;o__Micrococcales;f__Microbacteriaceae;g__Humibacter;s__Humibacter_ginsengiterrae | 0 |
| d__Bacteria;p__Firmicutes;c__Desulfitobacteriia;o__Desulfitobacteriales;f__Desulfitobacteriaceae;g__Desulfitobacterium;s__Desulfitobacterium_dichloroeliminans | 0 |
| d__Bacteria;p__Firmicutes;c__Bacilli;o__Lactobacillales;f__Lactobacillaceae;g__Lacticaseibacillus;s__Lactobacillus_camelliae | 0 |
| d__Bacteria;p__Proteobacteria;c__Alphaproteobacteria;o__Rhizobiales;f__Hyphomicrobiaceae;g__Pedomicrobium;s__uncultured_Hyphomicrobiaceae | 0 |
| d__Bacteria;p__Actinobacteriota;c__Actinobacteria;o__Micrococcales;f__Cellulomonadaceae;g__Actinotalea;s__Actinotalea_fermentans | 0 |
| d__Bacteria;p__Spirochaetota;c__Spirochaetia;o__Spirochaetales;f__Spirochaetaceae;g__Spirochaeta;s__uncultured_Spirochaetes | 0 |
| d__Bacteria;p__Firmicutes;c__Syntrophomonadia;o__Syntrophomonadales;f__Syntrophomonadaceae;g__uncultured;s__uncultured_Syntrophomonas | 0 |
| d__Bacteria;p__Firmicutes;c__Bacilli;o__Bacillales;f__Sporolactobacillaceae;g__Tuberibacillus;s__uncultured_bacterium | 0 |
| d__Bacteria;p__Proteobacteria;c__Alphaproteobacteria;o__Rhizobiales;f__Methyloligellaceae;g__uncultured;s__uncultured_Rhizobiales | 0 |
| d__Bacteria;p__Firmicutes;c__Bacilli;o__RF39;f__RF39;g__RF39;s__uncultured_bacterium | 0 |
| d__Bacteria;p__FCPU426;c__FCPU426;o__FCPU426;f__FCPU426;g__FCPU426;s__uncultured_Syntrophaceae | 0 |
| d__Bacteria;p__Proteobacteria;c__Alphaproteobacteria;o__Acetobacterales;f__Elioraeaceae;g__Elioraea;__ | 0 |
| d__Bacteria;p__Proteobacteria;c__Alphaproteobacteria;o__Rhizobiales;f__Rhizobiaceae;g__Mesorhizobium;s__uncultured_Mesorhizobium | 0 |
| d__Bacteria;p__Proteobacteria;c__Alphaproteobacteria;o__Caulobacterales;f__Caulobacteraceae;g__Phenylobacterium;s__unidentified | 0 |
| d__Bacteria;p__Firmicutes;c__Clostridia;o__Clostridiales;f__Clostridiaceae;g__Clostridium_sensu_stricto_12;s__Clostridium_sp. | 0 |
| d__Bacteria;p__Proteobacteria;c__Gammaproteobacteria;o__Pseudomonadales;f__211ds20;g__211ds20;s__metagenome | 0 |
| d__Bacteria;p__Firmicutes;c__Desulfitobacteriia;o__Desulfitobacteriales;__;__;__ | 0 |
| d__Bacteria;p__Proteobacteria;c__Alphaproteobacteria;o__Acetobacterales;f__Acetobacteraceae;g__Belnapia;__ | 0 |
| d__Bacteria;p__Bacteroidota;c__Kryptonia;o__Kryptoniales;f__BSV26;g__BSV26;s__denitrifying_bacterium | 0 |
| d__Bacteria;p__Myxococcota;c__Polyangia;o__Polyangiales;f__Polyangiaceae;g__Sorangium;s__uncultured_bacterium | 0 |
| d__Bacteria;p__Bacteroidota;c__Rhodothermia;o__Rhodothermales;f__Rhodothermaceae;g__uncultured;s__uncultured_Bacteroidetes | 0 |
| d__Bacteria;p__Proteobacteria;c__Alphaproteobacteria;o__Acetobacterales;f__Acetobacteraceae;g__uncultured;s__Roseomonas_sp. | 0 |
| d__Bacteria;p__Desulfobacterota;c__Desulfuromonadia;o__Desulfuromonadales;f__Geothermobacteraceae;g__Geothermobacter;__ | 0 |
| d__Bacteria;p__Firmicutes;c__TTA-B61;o__TTA-B61;f__TTA-B61;g__TTA-B61;s__uncultured_bacterium | 0 |
| d__Bacteria;p__Proteobacteria;c__Alphaproteobacteria;o__Rhodospirillales;f__Rhodospirillaceae;g__Candidatus_Riegeria;s__uncultured_bacterium | 0 |
| d__Bacteria;p__Proteobacteria;c__Gammaproteobacteria;o__Enterobacterales;f__Enterobacteriaceae;g__Kosakonia;s__Kosakonia_radicincitans | 0 |
| d__Bacteria;p__Proteobacteria;c__Alphaproteobacteria;o__Caulobacterales;f__Hyphomonadaceae;g__Maricaulis;__ | 0 |
| d__Bacteria;p__Firmicutes;c__Clostridia;o__Monoglobales;f__Monoglobaceae;g__Monoglobus;s__uncultured_Firmicutes | 0 |
| d__Bacteria;p__Desulfobacterota;c__Syntrophobacteria;o__Syntrophobacterales;f__Syntrophobacteraceae;__;__ | 0 |
| d__Bacteria;p__Chloroflexi;c__Anaerolineae;o__SBR1031;f__SBR1031;g__SBR1031;s__uncultured_organism | 0 |
| d__Bacteria;p__Planctomycetota;c__Pla3_lineage;o__Pla3_lineage;f__Pla3_lineage;g__Pla3_lineage;__ | 0 |
| d__Bacteria;p__Proteobacteria;c__Gammaproteobacteria;o__Enterobacterales;f__Morganellaceae;g__Providencia;__ | 0 |
| d__Bacteria;p__Acidobacteriota;c__Subgroup_5;o__Subgroup_5;f__Subgroup_5;g__Subgroup_5;s__uncultured_Acidobacteria | 0 |
| d__Bacteria;p__Firmicutes;c__Clostridia;o__Christensenellales;f__Christensenellaceae;g__uncultured;s__uncultured_low | 0 |
| d__Bacteria;p__Firmicutes;c__Clostridia;o__Peptostreptococcales-Tissierellales;f__Anaerovoracaceae;g__Family_XIII_UCG-001;s__uncultured_bacterium | 0 |
| d__Bacteria;p__Proteobacteria;c__Gammaproteobacteria;o__Methylococcales;f__Methylohalobiaceae;g__Methylothermus;__ | 0 |
| d__Bacteria;p__Bacteroidota;c__Bacteroidia;o__Bacteroidales;f__Prolixibacteraceae;g__uncultured;s__uncultured_eubacterium | 0 |
| d__Bacteria;p__Proteobacteria;c__Alphaproteobacteria;o__Rickettsiales;f__Mitochondria;g__Mitochondria;s__Pythium_ultimum | 0 |
| d__Bacteria;p__Proteobacteria;c__Alphaproteobacteria;o__Rhizobiales;f__Rhizobiales_Incertae_Sedis;g__Bauldia;s__uncultured_soil | 0 |
| d__Bacteria;p__Firmicutes;c__Negativicutes;o__Veillonellales-Selenomonadales;f__Sporomusaceae;g__Sporomusa;s__Sporomusa_acidovorans | 0 |
| d__Bacteria;p__Planctomycetota;c__Phycisphaerae;o__MSBL9;f__SM23-30;g__SM23-30;__ | 0 |
| d__Bacteria;p__Chloroflexi;c__Chloroflexia;o__Chloroflexales;f__Chloroflexaceae;g__FFCH7168;__ | 0 |
| d__Bacteria;p__Verrucomicrobiota;c__Verrucomicrobiae;o__Opitutales;f__Opitutaceae;g__Lacunisphaera;s__uncultured_Opitutus | 0 |
| d__Bacteria;p__Actinobacteriota;c__Actinobacteria;o__Micrococcales;f__Microbacteriaceae;g__Microbacteriaceae;s__Homoserinibacter_sp. | 0 |
| d__Bacteria;p__Firmicutes;c__Bacilli;o__Bacillales;f__Planococcaceae;g__Planomicrobium;s__Planomicrobium_sp. | 0 |
| d__Bacteria;p__Firmicutes;c__Bacilli;o__Bacillales;f__Bacillaceae;g__Fictibacillus;s__uncultured_Bacillus | 0 |
| d__Bacteria;p__Firmicutes;c__Clostridia;o__Oscillospirales;f__Hungateiclostridiaceae;g__Acetivibrio;s__metagenome | 0 |
| d__Bacteria;p__Actinobacteriota;c__Acidimicrobiia;o__IMCC26256;f__IMCC26256;g__IMCC26256;s__uncultured_Actinomycetales | 0 |
| d__Bacteria;p__Chloroflexi;c__Anaerolineae;o__Anaerolineales;f__Anaerolineaceae;g__uncultured;s__uncultured_Chloroflexi | 0 |
| d__Bacteria;p__Firmicutes;c__Desulfotomaculia;o__Carboxydothermales;f__Carboxydothermaceae;g__Carboxydothermus;__ | 0 |
| d__Bacteria;p__Hydrogenedentes;c__Hydrogenedentia;o__Hydrogenedentiales;f__Hydrogenedensaceae;g__Hydrogenedensaceae;s__uncultured_organism | 0 |
| d__Bacteria;p__Verrucomicrobiota;c__Lentisphaeria;o__Victivallales;f__Victivallaceae;g__Victivallis;__ | 0 |
| d__Bacteria;p__Firmicutes;c__Bacilli;o__Paenibacillales;f__Paenibacillaceae;g__Paenibacillus;s__Paenibacillus_thailandensis | 0 |
| d__Bacteria;p__Chloroflexi;c__Anaerolineae;o__1-20;f__1-20;g__1-20;s__uncultured_bacterium | 0 |
| d__Bacteria;p__Firmicutes;c__Bacilli;o__Staphylococcales;f__Staphylococcaceae;g__Staphylococcus;s__Staphylococcus_lugdunensis | 0 |
| d__Bacteria;p__Actinobacteriota;c__Actinobacteria;o__Streptosporangiales;f__Thermomonosporaceae;g__[Streptomyces]_thermoautotrophicus;__ | 0 |
| d__Bacteria;p__Proteobacteria;c__Gammaproteobacteria;o__Methylococcales;f__Methylococcaceae;g__Candidatus_Methylospira;__ | 0 |
| d__Bacteria;p__Proteobacteria;c__Gammaproteobacteria;o__Burkholderiales;f__Comamonadaceae;g__Comamonas;s__Comamonas_aquatica | 0 |
| d__Bacteria;p__Proteobacteria;c__Alphaproteobacteria;o__uncultured;f__uncultured;g__uncultured;s__uncultured_Rhizobiales | 0 |
| d__Bacteria;p__Firmicutes;c__Bacilli;o__Paenibacillales;f__Paenibacillaceae;g__Xylanibacillus;s__uncultured_Bacilli | 0 |
| d__Bacteria;p__Planctomycetota;c__Phycisphaerae;o__DG-20;f__DG-20;g__DG-20;s__uncultured_bacterium | 0 |
| d__Bacteria;p__Firmicutes;c__Bacilli;o__Brevibacillales;f__Brevibacillaceae;g__Brevibacillus;s__uncultured_bacterium | 0 |
| d__Bacteria;p__Patescibacteria;c__Microgenomatia;o__Candidatus_Curtissbacteria;f__Candidatus_Curtissbacteria;g__Candidatus_Curtissbacteria;s__uncultured_Microgenomates | 0 |
| d__Bacteria;p__Bacteroidota;c__Bacteroidia;o__Bacteroidales;f__Marinilabiliaceae;g__uncultured;__ | 0 |
| d__Bacteria;p__Firmicutes;c__Clostridia;o__Oscillospirales;f__uncultured;g__uncultured;s__uncultured_Clostridium | 0 |
| d__Bacteria;p__Firmicutes;c__Clostridia;o__Lachnospirales;f__Lachnospiraceae;g__Lachnospiraceae;s__uncultured_bacterium | 0 |
| d__Bacteria;p__Patescibacteria;c__Dojkabacteria;o__Dojkabacteria;f__Dojkabacteria;g__Dojkabacteria;s__candidate_division | 0 |
| d__Bacteria;p__Firmicutes;c__Clostridia;o__Oscillospirales;f__[Eubacterium]_coprostanoligenes_group;g__[Eubacterium]_coprostanoligenes_group;s__uncultured_rumen | 0 |
| d__Bacteria;p__Planctomycetota;c__Phycisphaerae;o__S-70;f__S-70;g__S-70;s__uncultured_Planctomycetales | 0 |
| d__Bacteria;p__WS1;c__WS1;o__WS1;f__WS1;g__WS1;__ | 0 |
| d__Bacteria;p__Patescibacteria;c__Dojkabacteria;o__Dojkabacteria;f__Dojkabacteria;g__Dojkabacteria;s__uncultured_organism | 0 |
| d__Bacteria;p__Firmicutes;c__Clostridia;o__Lachnospirales;f__Lachnospiraceae;g__Roseburia;s__uncultured_organism | 0 |
| d__Bacteria;p__Bdellovibrionota;c__Oligoflexia;o__Oligoflexales;f__Oligoflexaceae;g__Oligoflexaceae;s__uncultured_bacterium | 0 |
| d__Bacteria;p__Proteobacteria;c__Gammaproteobacteria;o__Methylococcales;f__Methylococcaceae;g__Methyloparacoccus;s__uncultured_bacterium | 0 |
| d__Bacteria;p__Myxococcota;c__Polyangia;o__Haliangiales;f__Haliangiaceae;g__Haliangium;s__myxobacterium_AT3-03 | 0 |
| d__Bacteria;p__Firmicutes;c__Clostridia;o__Oscillospirales;f__Ruminococcaceae;g__DTU089;__ | 0 |
| d__Bacteria;p__Actinobacteriota;c__Actinobacteria;o__Pseudonocardiales;f__Pseudonocardiaceae;g__Saccharomonospora;__ | 0 |
| d__Bacteria;p__Proteobacteria;c__Alphaproteobacteria;o__Rhizobiales;f__D05-2;g__D05-2;s__metagenome | 0 |
| d__Bacteria;p__Proteobacteria;c__Gammaproteobacteria;o__Enterobacterales;f__Morganellaceae;g__Providencia;s__Providencia_stuartii | 0 |
| d__Bacteria;p__Actinobacteriota;c__Actinobacteria;o__Micrococcales;f__Intrasporangiaceae;g__Ornithinicoccus;s__uncultured_bacterium | 0 |
| d__Bacteria;p__Firmicutes;c__Clostridia;o__Eubacteriales;f__Anaerofustaceae;g__Anaerofustis;__ | 0 |
| d__Bacteria;p__Actinobacteriota;c__Acidimicrobiia;o__Actinomarinales;f__uncultured;g__uncultured;s__uncultured_compost | 0 |
| d__Bacteria;p__Proteobacteria;c__Alphaproteobacteria;o__Micavibrionales;f__Micavibrionaceae;g__uncultured;s__unidentified_marine | 0 |
| d__Bacteria;p__Chloroflexi;c__Anaerolineae;o__Anaerolineales;f__Anaerolineaceae;g__Levilinea;__ | 0 |
| d__Bacteria;p__Firmicutes;c__Clostridia;o__Lachnospirales;f__Lachnospiraceae;g__Lachnospiraceae_XPB1014_group;s__uncultured_bacterium | 0 |
| d__Bacteria;p__Verrucomicrobiota;c__Kiritimatiellae;o__WCHB1-41;f__WCHB1-41;g__WCHB1-41;s__uncultured_rumen | 0 |
| d__Bacteria;p__Firmicutes;c__Thermoanaerobacteria;o__Thermoanaerobacterales;f__Thermoanaerobacteraceae;g__Caldanaerobius;s__uncultured_Thermoanaerobacterales | 0 |
| d__Bacteria;p__Proteobacteria;c__Gammaproteobacteria;o__Burkholderiales;f__Gallionellaceae;g__Gallionella;__ | 0 |
| d__Bacteria;p__Firmicutes;c__Thermoanaerobacteria;o__Thermoanaerobacterales;f__Family_III;g__Thermoanaerobacterium;s__Thermoanaerobacterium_saccharolyticum | 0 |
| d__Bacteria;p__Firmicutes;c__Symbiobacteriia;o__Symbiobacteriales;f__Symbiobacteriales;g__Caldinitratiruptor;s__uncultured_bacterium | 0 |
| d__Bacteria;p__Firmicutes;c__Thermovenabulia;o__Thermovenabulales;f__Thermovenabulales;g__Tepidanaerobacter;s__uncultured_Thermoanaerobacterales | 0 |
| d__Bacteria;p__Proteobacteria;c__Alphaproteobacteria;o__Kiloniellales;f__Kiloniellaceae;g__Pelagibius;__ | 0 |
| d__Bacteria;p__Myxococcota;c__Myxococcia;o__Myxococcales;f__Anaeromyxobacteraceae;g__Anaeromyxobacter;s__uncultured_delta | 0 |
| d__Bacteria;p__Proteobacteria;c__Alphaproteobacteria;o__Rhizobiales;f__Rhizobiaceae;g__Mycoplana;s__Mycoplana_dimorpha | 0 |
| d__Bacteria;p__Firmicutes;c__Clostridia;o__Lachnospirales;f__Lachnospiraceae;g__Lachnotalea;s__uncultured_bacterium | 0 |
| d__Bacteria;p__Firmicutes;c__Bacilli;o__Lactobacillales;f__Carnobacteriaceae;g__Trichococcus;__ | 0 |
| d__Bacteria;p__Proteobacteria;c__Gammaproteobacteria;o__Methylococcales;f__Methylococcaceae;g__uncultured;s__uncultured_bacterium | 0 |
| d__Bacteria;p__Proteobacteria;c__Gammaproteobacteria;o__Ectothiorhodospirales;f__Thioalkalispiraceae;g__uncultured;s__uncultured_Thiorhodospira | 0 |
| d__Bacteria;p__Firmicutes;c__Bacilli;o__Paenibacillales;f__Paenibacillaceae;g__Paenibacillus;s__Paenibacillus_crassostreae | 0 |
| d__Bacteria;p__Bacteroidota;c__Bacteroidia;o__Cytophagales;f__Amoebophilaceae;g__Candidatus_Amoebophilus;s__uncultured_bacterium | 0 |
| d__Bacteria;p__Patescibacteria;c__Parcubacteria;o__GWB1-42-6;f__GWB1-42-6;g__GWB1-42-6;__ | 0 |
| d__Bacteria;p__Firmicutes;c__Negativicutes;o__Veillonellales-Selenomonadales;f__Sporomusaceae;g__Thermosinus;s__uncultured_Thermosinus | 0 |
| d__Bacteria;p__Firmicutes;c__Clostridia;o__Oscillospirales;f__Oscillospiraceae;g__NK4A214_group;s__uncultured_rumen | 0 |
| d__Bacteria;p__Proteobacteria;c__Alphaproteobacteria;o__Elsterales;f__uncultured;g__uncultured;s__Rhodospirillaceae_bacterium | 0 |
| d__Bacteria;p__Spirochaetota;c__Spirochaetia;o__Spirochaetales;f__Spirochaetaceae;g__uncultured;__ | 0 |
| d__Bacteria;p__Proteobacteria;c__Gammaproteobacteria;o__Burkholderiales;f__Hydrogenophilaceae;__;__ | 0 |
| d__Bacteria;p__Proteobacteria;c__Alphaproteobacteria;o__uncultured;f__uncultured;g__uncultured;s__uncultured_Alphaproteobacteria | 0 |
| d__Bacteria;p__Proteobacteria;c__Alphaproteobacteria;o__Zavarziniales;f__Zavarziniaceae;g__Zavarziniaceae;s__metagenome | 0 |
| d__Bacteria;p__Proteobacteria;c__Gammaproteobacteria;o__Pseudomonadales;f__Moraxellaceae;g__Perlucidibaca;__ | 0 |
| d__Bacteria;p__Proteobacteria;c__Alphaproteobacteria;o__Thalassobaculales;f__uncultured;g__uncultured;s__metagenome | 0 |
| d__Bacteria;p__Firmicutes;c__Bacilli;o__Bacillales;f__Planococcaceae;g__Lysinibacillus;s__Lysinibacillus_fusiformis | 0 |
| d__Bacteria;p__Proteobacteria;c__Gammaproteobacteria;o__Pseudomonadales;f__Moraxellaceae;g__uncultured;s__uncultured_Pseudomonadaceae | 0 |
| d__Bacteria;p__Firmicutes;c__Desulfitobacteriia;o__Desulfitobacteriales;f__Heliobacteriaceae;__;__ | 0 |
| d__Bacteria;p__Firmicutes;c__Thermoanaerobacteria;o__Thermoanaerobacterales;f__Family_III;g__Thermoanaerobacterium;s__Thermoanaerobacterium_thermosulfurigenes | 0 |
| d__Bacteria;p__Acidobacteriota;c__Subgroup_22;o__Subgroup_22;f__Subgroup_22;g__Subgroup_22;s__uncultured_Acidobacteria | 0 |
| d__Bacteria;p__Planctomycetota;c__Planctomycetes;o__Pirellulales;f__Pirellulaceae;g__uncultured;s__uncultured_organism | 0 |
| d__Bacteria;p__Firmicutes;c__Clostridia;o__Oscillospirales;f__Hungateiclostridiaceae;g__HN-HF0106;s__metagenome | 0 |
| d__Bacteria;p__Firmicutes;c__Bacilli;o__Bacillales;f__Bacillaceae;g__Terribacillus;__ | 0 |
| d__Bacteria;p__Firmicutes;c__Bacilli;o__Lactobacillales;f__Lactobacillaceae;g__Loigolactobacillus;__ | 0 |
| d__Bacteria;p__Proteobacteria;c__Gammaproteobacteria;o__Methylococcales;f__Methylococcaceae;g__Methylocaldum;s__Methylocaldum_marinum | 0 |
| d__Bacteria;p__Proteobacteria;c__Gammaproteobacteria;o__Pseudomonadales;f__Cellvibrionaceae;__;__ | 0 |
| d__Bacteria;p__Firmicutes;c__Bacilli;o__Bacillales;f__Sporolactobacillaceae;g__Sporolactobacillus;s__Sporolactobacillus_laevolacticus | 0 |
| d__Bacteria;p__Proteobacteria;c__Gammaproteobacteria;o__Burkholderiales;f__Comamonadaceae;g__Limnohabitans;__ | 0 |
| d__Bacteria;p__Verrucomicrobiota;c__Lentisphaeria;o__Victivallales;f__vadinBE97;g__vadinBE97;__ | 0 |
| d__Bacteria;p__Actinobacteriota;c__Actinobacteria;o__Pseudonocardiales;f__Pseudonocardiaceae;g__Pseudonocardia;s__uncultured_actinobacterium | 0 |
| d__Bacteria;p__Firmicutes;c__Clostridia;o__Oscillospirales;f__Hungateiclostridiaceae;g__Ruminiclostridium;s__unidentified_eubacterium | 0 |
| d__Bacteria;p__Firmicutes;c__Bacilli;o__Paenibacillales;f__Paenibacillaceae;g__Paenibacillus;s__Paenibacillus_ehimensis | 0 |
| d__Bacteria;p__Acidobacteriota;c__Blastocatellia;o__Blastocatellales;f__Blastocatellaceae;g__Blastocatella;s__uncultured_Acidobacteria | 0 |
| d__Bacteria;p__Planctomycetota;c__vadinHA49;o__vadinHA49;f__vadinHA49;g__vadinHA49;s__uncultured_organism | 0 |
| d__Bacteria;p__Proteobacteria;c__Gammaproteobacteria;o__Enterobacterales;f__Enterobacteriaceae;g__Enterobacteriaceae;s__Phytobacter_sp. | 0 |
| d__Bacteria;p__Firmicutes;c__Syntrophomonadia;o__Syntrophomonadales;f__Syntrophomonadaceae;__;__ | 0 |
| d__Bacteria;p__Firmicutes;c__Bacilli;o__Bacillales;f__Bacillaceae;g__Bacillus;s__Bacillus_gaemokensis | 0 |
| d__Bacteria;p__Chloroflexi;c__Chloroflexia;o__Thermomicrobiales;f__Thermomicrobiaceae;g__Thermomicrobium;__ | 0 |
| d__Bacteria;p__Firmicutes;c__Clostridia;o__Lachnospirales;f__Defluviitaleaceae;g__Defluviitaleaceae_UCG-011;s__uncultured_bacterium | 0 |
| d__Bacteria;p__Chloroflexi;c__Dehalococcoidia;o__DscP2;f__DscP2;g__DscP2;__ | 0 |
| d__Bacteria;p__Firmicutes;c__Bacilli;o__Lactobacillales;f__Lactobacillaceae;g__Limosilactobacillus;s__Lactobacillus_vaginalis | 0 |
| d__Bacteria;p__Proteobacteria;c__Alphaproteobacteria;o__Defluviicoccales;f__uncultured;g__uncultured;s__uncultured_Alphaproteobacteria | 0 |
| d__Bacteria;p__Planctomycetota;c__Planctomycetes;o__Isosphaerales;f__Isosphaeraceae;g__Singulisphaera;s__Isosphaera_sp. | 0 |
| d__Bacteria;p__Proteobacteria;c__Gammaproteobacteria;o__Pseudomonadales;f__Moraxellaceae;g__uncultured;s__uncultured_soil | 0 |
| d__Bacteria;p__Actinobacteriota;c__Actinobacteria;o__Propionibacteriales;f__Propionibacteriaceae;g__Propionicicella;s__uncultured_bacterium | 0 |
| d__Bacteria;p__Proteobacteria;c__Gammaproteobacteria;o__Enterobacterales;f__Yersiniaceae;g__Serratia;__ | 0 |
| d__Bacteria;p__Firmicutes;c__Clostridia;o__Lachnospirales;f__Lachnospiraceae;g__Herbinix;s__uncultured_compost | 0 |
| d__Bacteria;p__Firmicutes;c__Bacilli;o__Paenibacillales;f__Paenibacillaceae;g__Thermobacillus;s__uncultured_Bacillus | 0 |
| d__Bacteria;p__Firmicutes;c__Clostridia;o__Oscillospirales;f__Oscillospiraceae;g__Papillibacter;s__uncultured_Clostridiaceae | 0 |
| d__Bacteria;p__Planctomycetota;c__Planctomycetes;o__Pirellulales;f__Pirellulaceae;g__Pirellula;s__uncultured_planctomycete | 0 |
| d__Bacteria;p__Chloroflexi;c__Anaerolineae;o__Caldilineales;f__Caldilineaceae;__;__ | 0 |
| d__Bacteria;p__Bacteroidota;c__Bacteroidia;o__Cytophagales;f__Spirosomaceae;g__Pseudarcicella;s__uncultured_bacterium | 0 |
| d__Bacteria;p__Desulfobacterota;c__Syntrophia;o__Syntrophales;f__uncultured;g__uncultured;__ | 0 |
| d__Bacteria;p__Firmicutes;c__Clostridia;o__Monoglobales;f__Monoglobaceae;g__Monoglobus;s__Acetivibrio_sp. | 0 |
| d__Bacteria;p__Firmicutes;c__Bacilli;o__Brevibacillales;f__Brevibacillaceae;g__Brevibacillus;s__Brevibacillus_borstelensis | 0 |
| d__Bacteria;p__Firmicutes;c__Thermoanaerobacteria;o__Thermoanaerobacterales;f__Family_III;g__Thermoanaerobacterium;s__uncultured_Thermoanaerobacter | 0 |
| d__Bacteria;p__Proteobacteria;c__Alphaproteobacteria;o__Rhizobiales;f__Rhizobiaceae;g__Aminobacter;s__Nitratireductor_aestuarii | 0 |
| d__Bacteria;p__Firmicutes;c__Clostridia;o__Peptostreptococcales-Tissierellales;f__Anaerovoracaceae;g__S5-A14a;__ | 0 |
| d__Bacteria;p__Firmicutes;c__Bacilli;o__Alicyclobacillales;f__Alicyclobacillaceae;g__Alicyclobacillus;s__Alicyclobacillus_disulfidooxidans | 0 |
| d__Bacteria;p__Proteobacteria;c__Gammaproteobacteria;o__Diplorickettsiales;f__Diplorickettsiaceae;g__uncultured;s__uncultured_Coxiellaceae | 0 |
| d__Bacteria;p__Proteobacteria;c__Alphaproteobacteria;o__Rhizobiales;f__Rhizobiaceae;g__Martelella;__ | 0 |
| d__Bacteria;p__Firmicutes;c__Clostridia;o__Oscillospirales;f__Butyricicoccaceae;g__UCG-008;s__uncultured_bacterium | 0 |
| d__Bacteria;p__Firmicutes;c__Bacilli;o__Bacillales;f__Bacillaceae;g__Bacillus;s__Quasibacillus_thermotolerans | 0 |
| d__Bacteria;p__Acidobacteriota;c__Acidobacteriae;o__Acidobacteriales;f__Acidobacteriaceae_(Subgroup_1);g__Bryocella;__ | 0 |
| d__Bacteria;p__Bdellovibrionota;c__Oligoflexia;o__0319-6G20;f__0319-6G20;g__0319-6G20;s__uncultured_Roseobacter | 0 |
| d__Bacteria;p__Planctomycetota;c__Planctomycetes;o__Pirellulales;f__Pirellulaceae;g__uncultured;s__uncultured_sludge | 0 |
| d__Bacteria;p__Desulfobacterota;c__Desulfomonilia;o__Desulfomonilales;f__Desulfomonilaceae;g__Desulfomonile;s__uncultured_bacterium | 0 |
| d__Bacteria;p__Firmicutes;c__Clostridia;o__Lachnospirales;f__Lachnospiraceae;g__Marvinbryantia;s__uncultured_bacterium | 0 |
| d__Bacteria;p__Proteobacteria;c__Alphaproteobacteria;o__Rickettsiales;f__bac2nit3;g__bac2nit3;__ | 0 |
| d__Bacteria;p__Firmicutes;c__Clostridia;o__Lachnospirales;f__Lachnospiraceae;g__Marvinbryantia;__ | 0 |
| d__Bacteria;p__Proteobacteria;c__Alphaproteobacteria;o__Rickettsiales;f__Rickettsiaceae;g__Orientia;s__Orientia_tsutsugamushi | 0 |
| d__Bacteria;p__Firmicutes;c__Bacilli;o__Thermoactinomycetales;f__Thermoactinomycetaceae;g__Novibacillus;s__uncultured_bacterium | 0 |
| d__Bacteria;p__Firmicutes;c__Sulfobacillia;o__Sulfobacillales;f__Sulfobacillaceae;g__Sulfobacillus;s__uncultured_bacterium | 0 |
| d__Bacteria;p__Proteobacteria;c__Gammaproteobacteria;o__Nitrococcales;__;__;__ | 0 |
| d__Bacteria;p__Bacteroidota;c__Bacteroidia;o__Flavobacteriales;f__Cryomorphaceae;g__Vicingus;__ | 0 |
| d__Bacteria;p__Firmicutes;c__Bacilli;o__Bacillales;f__Bacillaceae;g__Bacillaceae;s__uncultured_bacterium | 0 |
| d__Bacteria;p__Proteobacteria;c__Gammaproteobacteria;o__Acidithiobacillales;f__Acidithiobacillaceae;g__KCM-B-112;s__uncultured_Chromatiaceae | 0 |
| d__Bacteria;p__Firmicutes;c__Bacilli;o__Paenibacillales;f__Paenibacillaceae;g__Xylanibacillus;s__uncultured_compost | 0 |
| d__Bacteria;p__Actinobacteriota;c__Thermoleophilia;o__Solirubrobacterales;f__67-14;g__67-14;s__metagenome | 0 |
| d__Bacteria;p__Firmicutes;c__Clostridia;o__Eubacteriales;f__Alkalibacteraceae;g__Alkalibacter;__ | 0 |
| d__Bacteria;p__Firmicutes;c__Clostridia;o__Clostridiales;f__Oxobacteraceae;g__Oxobacter;s__unidentified_eubacterium | 0 |
| d__Bacteria;p__Firmicutes;c__Bacilli;o__Paenibacillales;f__Paenibacillaceae;g__Paenibacillus;s__Paenibacillus_sabinae | 0 |
| d__Bacteria;p__Armatimonadota;c__Chthonomonadetes;o__Chthonomonadales;f__Chthonomonadaceae;g__Chthonomonas;__ | 0 |
| d__Bacteria;p__Actinobacteriota;c__Acidimicrobiia;o__uncultured;f__uncultured;g__uncultured;s__uncultured_Acidimicrobiaceae | 0 |
| d__Bacteria;p__Proteobacteria;c__Alphaproteobacteria;o__Sphingomonadales;f__Sphingomonadaceae;g__Polymorphobacter;__ | 0 |
| d__Bacteria;p__Proteobacteria;c__Gammaproteobacteria;o__Burkholderiales;f__Neisseriaceae;g__Neisseria;s__Neisseria_meningitidis | 0 |
| d__Bacteria;p__Actinobacteriota;c__Actinobacteria;o__Bifidobacteriales;f__Bifidobacteriaceae;g__Bifidobacterium;s__Bifidobacterium_breve | 0 |
| d__Bacteria;p__Firmicutes;c__Sulfobacillia;o__Sulfobacillales;f__Sulfobacillaceae;g__Sulfobacillus;__ | 0 |
| d__Bacteria;p__Proteobacteria;c__Alphaproteobacteria;o__Rhizobiales;f__Methyloligellaceae;g__uncultured;s__uncultured_Hyphomicrobiaceae | 0 |
| d__Bacteria;p__Proteobacteria;c__Gammaproteobacteria;o__1013-28-CG33;f__1013-28-CG33;g__1013-28-CG33;__ | 0 |
| d__Bacteria;p__Bacteroidota;c__Bacteroidia;o__Cytophagales;f__Cyclobacteriaceae;g__Cyclobacterium;__ | 0 |
| d__Bacteria;p__Proteobacteria;c__Gammaproteobacteria;o__Nitrosococcales;f__Nitrosococcaceae;g__Nitrosococcus;s__Nitrosococcus_halophilus | 0 |
| d__Bacteria;p__Proteobacteria;c__Gammaproteobacteria;o__Pseudomonadales;f__Halomonadaceae;g__Halomonas;s__Halomonas_sp. | 0 |
| d__Bacteria;p__Actinobacteriota;c__Actinobacteria;o__Micrococcales;f__Microbacteriaceae;g__Cryobacterium;s__Cryobacterium_psychrotolerans | 0 |
| d__Bacteria;p__Firmicutes;c__Bacilli;o__Paenibacillales;f__Paenibacillaceae;g__Cohnella;s__Cohnella_lubricantis | 0 |
| d__Bacteria;p__Proteobacteria;c__Alphaproteobacteria;o__Sphingomonadales;f__Sphingomonadaceae;g__Sphingomonas;s__Sphingomonas_oligophenolica | 0 |
| d__Bacteria;p__Patescibacteria;c__Microgenomatia;o__Candidatus_Levybacteria;f__Candidatus_Levybacteria;g__Candidatus_Levybacteria;s__uncultured_Microgenomates | 0 |
| d__Bacteria;p__Firmicutes;c__Bacilli;o__Bacillales;f__Bacillaceae;g__Sinibacillus;__ | 0 |
| d__Bacteria;p__Proteobacteria;c__Alphaproteobacteria;o__Rhizobiales;f__Devosiaceae;g__Arsenicitalea;s__uncultured_bacterium | 0 |
| d__Bacteria;p__Actinobacteriota;c__Actinobacteria;o__Micrococcales;f__Intrasporangiaceae;g__Aquipuribacter;__ | 0 |
| d__Bacteria;p__Bacteroidota;c__Bacteroidia;o__Flavobacteriales;f__Flavobacteriaceae;g__Planktosalinus;s__uncultured_bacterium | 0 |
| d__Bacteria;p__Chloroflexi;c__Ktedonobacteria;o__Ktedonobacterales;f__JG30-KF-AS9;g__JG30-KF-AS9;s__uncultured_Ktedonobacterales | 0 |
| d__Bacteria;p__Actinobacteriota;c__Actinobacteria;o__Pseudonocardiales;f__Pseudonocardiaceae;g__Lechevalieria;__ | 0 |
| d__Bacteria;p__Firmicutes;c__D8A-2;o__D8A-2;f__D8A-2;g__D8A-2;s__uncultured_Firmicutes | 0 |
| d__Bacteria;p__Proteobacteria;c__Gammaproteobacteria;o__Xanthomonadales;f__Xanthomonadaceae;g__Luteimonas;s__uncultured_Xanthomonadaceae | 0 |
| d__Bacteria;p__Firmicutes;c__Bacilli;o__Thermoactinomycetales;f__Thermoactinomycetaceae;g__Melghirimyces;s__Melghirimyces_thermohalophilus | 0 |
| d__Bacteria;p__Proteobacteria;c__Gammaproteobacteria;o__Nitrococcales;f__Nitrococcales_Incertae_Sedis;g__Methylonatrum;s__uncultured_bacterium | 0 |
| d__Bacteria;p__Firmicutes;c__Clostridia;o__Clostridiales;f__Caloramatoraceae;g__Fonticella;s__uncultured_Firmicutes | 0 |
| d__Bacteria;p__Bacteroidota;c__Bacteroidia;o__Chitinophagales;f__Chitinophagaceae;g__Haoranjiania;s__Arachidicoccus_sp. | 0 |
| d__Bacteria;p__Proteobacteria;c__Gammaproteobacteria;o__Burkholderiales;f__Nitrosomonadaceae;g__Nitrosomonas;s__uncultured_Nitrosomonas | 0 |
| d__Bacteria;p__Firmicutes;c__Bacilli;o__Staphylococcales;f__Staphylococcaceae;g__Staphylococcus;s__Staphylococcus_equorum | 0 |
| d__Bacteria;p__Actinobacteriota;c__Actinobacteria;o__Corynebacteriales;f__Nocardiaceae;g__Nocardia;s__Nocardia_asteroides | 0 |
| d__Bacteria;p__Firmicutes;c__Bacilli;o__Thermoactinomycetales;f__Thermoactinomycetaceae;g__Kroppenstedtia;s__Kroppenstedtia_guangzhouensis | 0 |
| d__Bacteria;p__Firmicutes;c__Clostridia;o__Clostridiales;f__Clostridiaceae;g__Hathewaya;__ | 0 |
| d__Bacteria;p__Firmicutes;c__Clostridia;o__Eubacteriales;f__Eubacteriaceae;g__Acetobacterium;__ | 0 |
| d__Bacteria;p__Proteobacteria;c__Gammaproteobacteria;o__Xanthomonadales;f__Rhodanobacteraceae;g__Rhodanobacter;s__Dyella_sp. | 0 |
| d__Bacteria;p__Firmicutes;c__Bacilli;o__Bacillales;f__Sporolactobacillaceae;g__Pullulanibacillus;s__uncultured_bacterium | 0 |
| d__Bacteria;p__Firmicutes;c__Thermacetogenia;o__Thermacetogeniales;f__Thermacetogeniaceae;g__Syntrophaceticus;s__Syntrophaceticus_schinkii | 0 |
| d__Bacteria;p__Proteobacteria;c__Gammaproteobacteria;o__Burkholderiales;f__Nitrosomonadaceae;g__966-1;s__uncultured_bacterium | 0 |
| d__Bacteria;p__Firmicutes;c__Clostridia;o__Lachnospirales;f__Lachnospiraceae;g__[Eubacterium]_ventriosum_group;__ | 0 |
| d__Bacteria;p__Actinobacteriota;c__Actinobacteria;o__Catenulisporales;f__Actinospicaceae;g__Actinospica;s__Actinospica_durhamensis | 0 |
| d__Bacteria;p__Actinobacteriota;c__Actinobacteria;o__Streptomycetales;f__Streptomycetaceae;g__Streptomyces;s__Streptomyces_thermocarboxydus | 0 |
| d__Bacteria;p__Actinobacteriota;c__Actinobacteria;o__Streptosporangiales;f__Streptosporangiales_Incertae_Sedis;g__Motilibacter;__ | 0 |
| d__Bacteria;p__Campylobacterota;c__Campylobacteria;o__Campylobacterales;f__Arcobacteraceae;g__uncultured;__ | 0 |
| d__Bacteria;p__Campylobacterota;c__Campylobacteria;o__Campylobacterales;f__Arcobacteraceae;__;__ | 0 |
| d__Bacteria;p__Proteobacteria;c__Gammaproteobacteria;o__Enterobacterales;f__Enterobacteriaceae;g__Enterobacter;s__Klebsiella_aerogenes | 0 |
| d__Bacteria;p__Actinobacteriota;c__Actinobacteria;o__Micrococcales;f__Micrococcaceae;g__Rothia;__ | 0 |
| d__Bacteria;p__Latescibacterota;c__Latescibacteria;o__Latescibacterales;f__Latescibacteraceae;g__Latescibacteraceae;s__uncultured_bacterium | 0 |
| d__Bacteria;p__Firmicutes;c__Bacilli;o__Bacillales;f__Marinococcaceae;g__Marinococcaceae;s__Bacillaceae_bacterium | 0 |
| d__Bacteria;p__Actinobacteriota;c__Actinobacteria;o__Corynebacteriales;f__Corynebacteriaceae;g__Corynebacterium;s__uncultured_bacterium | 0 |
| d__Bacteria;p__Bacteroidota;c__Bacteroidia;o__Bacteroidales;f__Rikenellaceae;g__DMER64;s__uncultured_bacterium | 0 |
| d__Bacteria;p__Firmicutes;c__Syntrophomonadia;o__Syntrophomonadales;f__Syntrophomonadaceae;g__Syntrophomonas;s__uncultured_Syntrophomonas | 0 |
| d__Bacteria;p__Nitrospirota;c__4-29-1;o__4-29-1;f__4-29-1;g__4-29-1;s__uncultured_bacterium | 0 |
| d__Bacteria;p__Firmicutes;c__Thermaerobacteria;o__Thermaerobacterales;f__Thermaerobacteraceae;g__Thermaerobacter;s__Thermaerobacter_litoralis | 0 |
| d__Bacteria;p__Firmicutes;c__Bacilli;o__Bacillales;f__Sporolactobacillaceae;g__Pullulanibacillus;s__Pullulanibacillus_naganoensis | 0 |
| d__Bacteria;p__Proteobacteria;c__Gammaproteobacteria;o__Cardiobacteriales;f__Cardiobacteriaceae;g__Cardiobacterium;s__uncultured_bacterium | 0 |
| d__Bacteria;p__Proteobacteria;c__Gammaproteobacteria;o__Acidiferrobacterales;f__Acidiferrobacteraceae;g__Sulfurifustis;s__uncultured_proteobacterium | 0 |
| d__Bacteria;p__Actinobacteriota;c__Actinobacteria;o__Actinomycetales;f__Actinomycetaceae;g__F0332;__ | 0 |
| d__Bacteria;p__Firmicutes;c__Clostridia;o__Peptostreptococcales-Tissierellales;f__Peptostreptococcaceae;g__[Eubacterium]_tenue_group;s__[Eubacterium]_tenue | 0 |
| d__Bacteria;p__Deinococcota;c__Deinococci;o__Thermales;f__Thermaceae;g__Meiothermus;__ | 0 |
| d__Bacteria;p__Actinobacteriota;c__Actinobacteria;o__Actinomycetales;f__Actinomycetaceae;g__Actinomyces;s__Actinomyces_oris | 0 |
| d__Bacteria;p__Actinobacteriota;c__Actinobacteria;o__Micrococcales;f__Micrococcaceae;g__Micrococcus;s__Micrococcus_luteus | 0 |
| d__Bacteria;p__Actinobacteriota;c__Actinobacteria;o__Actinomycetales;f__Actinomycetaceae;g__F0332;s__uncultured_bacterium | 0 |
| d__Bacteria;p__Actinobacteriota;c__Actinobacteria;o__Actinomycetales;f__Actinomycetaceae;g__Actinomyces;s__Actinomyces_naeslundii | 0 |
| d__Bacteria;p__Acidobacteriota;c__AT-s3-28;o__AT-s3-28;f__AT-s3-28;g__AT-s3-28;s__uncultured_bacterium | 0 |
| d__Bacteria;p__Proteobacteria;c__Alphaproteobacteria;o__Rhizobiales;f__Xanthobacteraceae;g__Xanthobacteraceae;s__uncultured_Alphaproteobacteria | 0 |
| d__Bacteria;p__Proteobacteria;c__Gammaproteobacteria;o__Xanthomonadales;f__Rhodanobacteraceae;g__Rhodanobacter;s__Rhodanobacter_humi | 0 |
| d__Bacteria;p__Firmicutes;c__Clostridia;o__Peptostreptococcales-Tissierellales;f__Family_XI;g__Tissierella;s__Tissierella_sp. | 0 |
| d__Bacteria;p__Chloroflexi;c__Anaerolineae;o__Anaerolineales;f__Anaerolineaceae;g__Levilinea;s__uncultured_bacterium | 0 |
| d__Bacteria;p__Proteobacteria;c__Gammaproteobacteria;o__Burkholderiales;f__Neisseriaceae;g__Neisseria;s__uncultured_bacterium | 0 |
| d__Bacteria;p__Firmicutes;c__Bacilli;o__Lactobacillales;f__Lactobacillaceae;g__Ligilactobacillus;s__Lactobacillus_murinus | 0 |
| d__Bacteria;p__Proteobacteria;c__Gammaproteobacteria;o__Xanthomonadales;f__Rhodanobacteraceae;g__Dyella;s__Fulvimonas_sp. | 0 |
| d__Bacteria;p__Actinobacteriota;c__Actinobacteria;o__Micrococcales;f__Microbacteriaceae;g__Naasia;s__Naasia_aerilata | 0 |
| d__Bacteria;p__Firmicutes;c__Bacilli;o__Lactobacillales;f__Enterococcaceae;g__Enterococcus;s__Enterococcus_avium | 0 |
| d__Bacteria;p__Firmicutes;c__Clostridia;o__Clostridiales;f__Clostridiaceae;g__Clostridium_sensu_stricto_1;s__Clostridium_longisporum | 0 |
| d__Bacteria;p__Fusobacteriota;c__Fusobacteriia;o__Fusobacteriales;f__Fusobacteriaceae;g__Fusobacterium;s__Fusobacterium_periodonticum | 0 |
| d__Bacteria;p__Acidobacteriota;c__Blastocatellia;o__Blastocatellales;f__Blastocatellaceae;g__Stenotrophobacter;s__uncultured_Acidobacteria | 0 |
| d__Bacteria;p__Proteobacteria;c__Alphaproteobacteria;o__Acetobacterales;f__Acetobacteraceae;g__Acidicaldus;s__uncultured_bacterium | 0 |
| d__Bacteria;p__Campylobacterota;c__Campylobacteria;o__Campylobacterales;f__Campylobacteraceae;g__Campylobacter;s__Campylobacter_concisus | 0 |
| d__Bacteria;p__Firmicutes;c__Clostridia;o__uncultured;f__uncultured;g__uncultured;s__uncultured_prokaryote | 0 |
| d__Bacteria;p__Actinobacteriota;c__Actinobacteria;o__Micrococcales;f__Micrococcaceae;g__Pseudarthrobacter;s__uncultured_bacterium | 0 |
| d__Bacteria;p__Actinobacteriota;c__Actinobacteria;o__Micrococcales;f__Dermatophilaceae;g__uncultured;s__uncultured_bacterium | 0 |
| d__Bacteria;p__Acidobacteriota;c__Acidobacteriae;o__Acidobacteriales;f__Acidobacteriaceae_(Subgroup_1);g__Occallatibacter;s__bacterium_enrichment | 0 |
| d__Bacteria;p__Firmicutes;c__Clostridia;o__Lachnospirales;f__Lachnospiraceae;g__Lachnospiraceae_ND3007_group;__ | 0 |
| d__Bacteria;p__Campylobacterota;c__Campylobacteria;o__Campylobacterales;f__Sulfurimonadaceae;g__Sulfuricurvum;s__uncultured_bacterium | 0 |
| d__Bacteria;p__Proteobacteria;c__Alphaproteobacteria;o__Rhizobiales;f__Rhizobiaceae;g__Mesorhizobium;s__Mesorhizobium_amorphae | 0 |
| d__Bacteria;p__Proteobacteria;c__Alphaproteobacteria;o__Rhizobiales;f__Rhizobiaceae;g__Rhizobiaceae;s__Mesorhizobium_thiogangeticum | 0 |
| d__Bacteria;p__Proteobacteria;c__Gammaproteobacteria;o__Burkholderiales;f__Comamonadaceae;g__Ramlibacter;s__uncultured_beta | 0 |
| d__Bacteria;p__Firmicutes;c__Clostridia;o__Oscillospirales;f__Ruminococcaceae;g__Ruminococcus;s__Ruminococcus_albus | 0 |
| d__Bacteria;p__Firmicutes;c__Clostridia;o__Thermincolales;f__Thermincolaceae;g__Thermincola;s__uncultured_Thermincola | 0 |
| d__Bacteria;p__Firmicutes;c__Bacilli;o__Bacillales;f__Bacillaceae;g__Bacillus;s__Bacillus_weihaiensis | 0 |
| d__Bacteria;p__Firmicutes;c__Bacilli;o__Paenibacillales;f__Paenibacillaceae;g__Thermobacillus;s__Thermobacillus_sp. | 0 |
| d__Bacteria;p__Proteobacteria;c__Alphaproteobacteria;o__Sphingomonadales;f__Sphingomonadaceae;g__Altererythrobacter;s__Altererythrobacter_sp. | 0 |
| d__Bacteria;p__Firmicutes;c__Desulfitobacteriia;o__Desulfitobacteriales;f__uncultured;g__uncultured;s__uncultured_organism | 0 |
| d__Bacteria;p__Proteobacteria;c__Alphaproteobacteria;o__Sphingomonadales;f__Sphingomonadaceae;g__Sphingorhabdus;__ | 0 |
| d__Bacteria;p__Firmicutes;c__Bacilli;o__Thermoactinomycetales;f__Thermoactinomycetaceae;g__Shimazuella;s__Shimazuella_sp. | 0 |
| d__Bacteria;p__Firmicutes;c__Bacilli;o__Caldalkalibacillales;f__Caldalkalibacillaceae;g__Caldalkalibacillus;s__uncultured_bacterium | 0 |
| d__Bacteria;p__Firmicutes;c__Bacilli;o__Staphylococcales;f__Staphylococcaceae;g__Staphylococcus;s__Staphylococcus_sp. | 0 |
| d__Bacteria;p__Desulfobacterota;c__Desulfovibrionia;o__Desulfovibrionales;f__Desulfovibrionaceae;g__Bilophila;s__uncultured_bacterium | 0 |
| d__Bacteria;p__Firmicutes;c__Clostridia;o__Lachnospirales;f__Lachnospiraceae;g__Lachnoclostridium;s__[Clostridium]_bolteae | 0 |
| d__Bacteria;p__Firmicutes;c__Bacilli;o__Lactobacillales;f__Streptococcaceae;g__Streptococcus;s__Streptococcus_parasanguinis | 0 |
| d__Bacteria;p__Actinobacteriota;c__Actinobacteria;o__Micrococcales;f__Micrococcaceae;g__Rothia;s__Rothia_mucilaginosa | 0 |
| d__Bacteria;p__Gemmatimonadota;c__Gemmatimonadetes;o__Gemmatimonadales;f__Gemmatimonadaceae;g__uncultured;s__uncultured_soil | 0 |
| d__Bacteria;p__Proteobacteria;c__Gammaproteobacteria;o__Xanthomonadales;f__Rhodanobacteraceae;g__Chujaibacter;s__uncultured_Xanthomonadaceae | 0 |
| d__Bacteria;p__Firmicutes;c__Bacilli;o__Bacillales;f__Bacillaceae;g__Bacillus;s__Bacillus_marisflavi | 0 |
| d__Bacteria;p__Proteobacteria;c__Gammaproteobacteria;o__Burkholderiales;f__MWH-UniP1_aquatic_group;g__MWH-UniP1_aquatic_group;s__uncultured_bacterium | 0 |
| d__Bacteria;p__Proteobacteria;c__Gammaproteobacteria;o__Burkholderiales;f__Comamonadaceae;g__Sphaerotilus;__ | 0 |
| d__Bacteria;p__Actinobacteriota;c__Actinobacteria;o__Micrococcales;f__Micrococcaceae;g__Kocuria;s__Kocuria_palustris | 0 |
| d__Bacteria;p__Actinobacteriota;c__Actinobacteria;o__Micrococcales;f__Microbacteriaceae;g__Gryllotalpicola;s__Gryllotalpicola_kribbensis | 0 |
| d__Bacteria;p__Gemmatimonadota;c__Gemmatimonadetes;o__Gemmatimonadales;f__Gemmatimonadaceae;g__uncultured;s__Acidobacteria_bacterium | 0 |
| d__Bacteria;p__Firmicutes;c__Clostridia;o__Lachnospirales;f__Lachnospiraceae;g__Catonella;__ | 0 |
| d__Bacteria;p__Actinobacteriota;c__Actinobacteria;o__Frankiales;f__Geodermatophilaceae;g__Antricoccus;s__uncultured_bacterium | 0 |
| d__Bacteria;p__Firmicutes;c__Clostridia;o__Eubacteriales;f__Garciellaceae;g__Irregularibacter;s__uncultured_bacterium | 0 |
| d__Bacteria;p__Firmicutes;c__Bacilli;o__Bacillales;f__Bacillaceae;g__Virgibacillus;s__Virgibacillus_halodenitrificans | 0 |
| d__Bacteria;p__Firmicutes;c__Limnochordia;o__B55-F-B-G02;f__B55-F-B-G02;g__B55-F-B-G02;s__uncultured_prokaryote | 0 |
| d__Bacteria;p__Firmicutes;c__Clostridia;o__Lachnospirales;f__Lachnospiraceae;g__[Eubacterium]_hallii_group;s__uncultured_bacterium | 0 |
| d__Bacteria;p__Actinobacteriota;c__Actinobacteria;o__Propionibacteriales;f__Nocardioidaceae;g__Nocardioides;s__Nocardioides_simplex | 0 |
| d__Bacteria;p__Proteobacteria;c__Gammaproteobacteria;o__Arenicellales;f__Arenicellaceae;g__uncultured;s__uncultured_gamma | 0 |
| d__Bacteria;p__Proteobacteria;c__Gammaproteobacteria;o__Enterobacterales;f__Erwiniaceae;g__Pantoea;s__Mixta_calida | 0 |
